# Using Constellation Pharmacology to Characterize a Novel α-Conotoxin from *Conus ateralbus*

**DOI:** 10.3390/md22030118

**Published:** 2024-02-29

**Authors:** Jorge L. B. Neves, Cristoval Urcino, Kevin Chase, Cheryl Dowell, Arik J. Hone, David Morgenstern, Victor M. Chua, Iris Bea L. Ramiro, Julita S. Imperial, Lee S. Leavitt, Jasmine Phan, Fernando A. Fisher, Maren Watkins, Shrinivasan Raghuraman, Jortan O. Tun, Beatrix M. Ueberheide, J. Michael McIntosh, Vitor Vasconcelos, Baldomero M. Olivera, Joanna Gajewiak

**Affiliations:** 1 Interdisciplinary Centre of Marine and Environmental Research (CIIMAR/CIMAR-LA), University of Porto, Terminal de Cruzeiros do Porto de Leixões, Avenida General Norton de Matos, S/N, 4450-208 Matosinhos, Portugal; 2 School of Biological Sciences, University of Utah, Salt Lake City, UT 84112, USA; 3 Mental Illness Research Education and Clinical Center, George E. Whalen Veterans Affairs Medical Center, Salt Lake City, UT 84148, USA; 4 Departments of Biochemistry and Molecular Pharmacology, New York University Langone Medical Center, New York, NY 10016, USA; 5 The Marine Science Institute, University of the Philippines, Quezon City 1101, Philippines; 6 Department of Psychiatry, University of Utah, Salt Lake City, UT 84108, USA; 7 Mental Health Department, George E. Whalen Veterans Affairs Medical Center, Salt Lake City, UT 84148, USA; 8 Faculty of Sciences, University of Porto, Rua do Campo Alegre, 4169-007 Porto, Portugal

**Keywords:** conotoxin, nAChRs, Constellation Pharmacology, DRG neurons

## Abstract

The venom of cone snails has been proven to be a rich source of bioactive peptides that target a variety of ion channels and receptors. α-Conotoxins (αCtx) interact with nicotinic acetylcholine receptors (nAChRs) and are powerful tools for investigating the structure and function of the various nAChR subtypes. By studying how conotoxins interact with nAChRs, we can improve our understanding of these receptors, leading to new insights into neurological diseases associated with nAChRs. Here, we describe the discovery and characterization of a novel conotoxin from *Conus ateralbus*, αCtx-AtIA, which has an amino acid sequence homologous to the well-described αCtx-PeIA, but with a different selectivity profile towards nAChRs. We tested the synthetic αCtx-AtIA using the calcium imaging-based Constellation Pharmacology assay on mouse DRG neurons and found that αCtx-AtIA significantly inhibited ACh-induced calcium influx in the presence of an α7 positive allosteric modulator, PNU-120596 (PNU). However, αCtx-AtIA did not display any activity in the absence of PNU. These findings were further validated using two-electrode voltage clamp electrophysiology performed on oocytes overexpressing mouse α3β4, α6/α3β4 and α7 nAChRs subtypes. We observed that αCtx-AtIA displayed no or low potency in blocking α3β4 and α6/α3β4 receptors, respectively, but improved potency and selectivity to block α7 nAChRs when compared with αCtx-PeIA. Through the synthesis of two additional analogs of αCtx-AtIA and subsequent characterization using Constellation Pharmacology, we were able to identify residue Trp18 as a major contributor to the activity of the peptide.

## 1. Introduction

Cone snails (genus *Conus*) are gastropods that employ venom to immobilize prey, defend against predators, and interact with other living organisms in their environment. Each species produces hundreds of peptides (conotoxins, conopeptides, Ctx) and small, bioactive molecules in their venom gland [1,2,3]. More than 40 years have passed since the characterization of the first conotoxin, αCtx-GI, a 13-amino acid peptide with two disulfide bonds from *Conus geographus* venom [4]. Since then, hundreds of bioactive molecules with pharmacological potential have been discovered, synthesized, and characterized [5,6,7]. The venom of cone snails has proven to be a rich source of bioactive peptides, targeting many different ion channels and receptors. Thus, conotoxins are useful and effective tools to investigate muscular, cardiovascular, and neuronal systems [8,9,10]. 

Cone snails are organized into three groups according to their preferred prey: piscivores (fish hunters), molluscivores (mollusk hunters), and vermivores (worm hunters). The majority of the peptides identified to date can be attributed to the study of piscivorous *Conus* [5,11]. However, there are considerably more vermivorous species, which provides an opportunity to discover bioactive molecules with novel pharmacological specificity [12,13,14]. Our laboratories have been systematically exploring bioactive peptides from different lineages of worm-hunting cone snails whenever a sufficient amount of venom becomes available. One of the least investigated lineages is *Kalloconus*, a geographically restricted group of *Conus* species found only off the coast of West Africa. The senior author (JLBN) had the opportunity to collect *Conus ateralbus*, an endemic species in the subgenus *Kalloconus* that is unique to the Cape Verde Islands. Prior characterization of one venom component revealed it to be a novel δCtx that inhibits the inactivation of Na_v_ channels [15].

**Figure 1:**
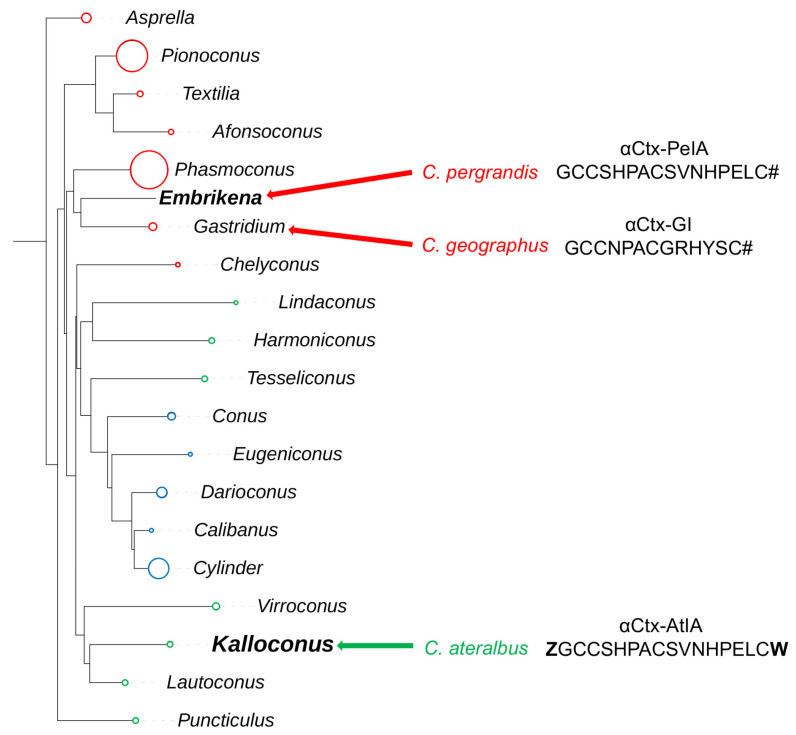
A phylogenetic tree showing the large clade of *Conus*. Lineages that hunt fish are indicated in red, those that hunt snails in blue, and those that hunt worms in green. The lineages to which the species *C. pergrandis, C. ateralbus,* and *C. geographus* belong are indicated by red and green arrows. The peptide sequences compared in this work, αCtx-PeIA and αCtx-AtIA, which were found in *C. pergrandis* and *C. ateralbus,* respectively, are listed next to the species, along with αCtx-GI from *C. geographus*, which was discovered in 1981 by Gray et al. [4].

This study focuses on another venom component that was isolated using bioactivity-guided purification and was shown to belong to the αCtx family, a well-characterized group of peptide toxins that target nicotinic acetylcholine receptors (nAChRs). When purified from venom, the amino acid sequence of this peptide was strikingly similar to a previously characterized αCtx from a fish-hunting cone snail, *Conus pergrandis* [16]. A phylogenetic tree of the genus *Conus* showing the relationship between *Embrikena* and *Kalloconus* lineages, which contain *C. pergrandis* and *C. ateralbus*, respectively, is shown in Figure 1. Given their divergent biology and phylogenetic distance from one another, it was not expected that these two species would have any highly homologous venom components.

The αCtx are one of the most widely distributed and intensively investigated of *Conus* venom peptide families. However, no αCtx from the *Kalloconus* lineage have been investigated previously. In this article, we describe the comprehensive characterization of the αCtx-AtIA peptide from *C. ateralbus* and conduct a bioactivity comparison with the previously characterized homologous αCtx-PeIA from *C. pergrandis*.

Compounds targeting nAChRs have demonstrated biomedical potential, such as uncovering new mechanisms for the relief of chronic pain and neuronal disorders, including Alzheimer’s and Parkinson’s disease [17,18,19,20]. The αCtx are among the families of toxins that have been extensively investigated due to their ability to discriminate nAChR isoforms [21,22]. By studying how αCtx interact with nAChRs, we can gain a better understanding of the structure and function of these receptors, leading to new insights into neurological diseases associated with nAChRs. 

## 2. Results

### 2.1. Discovery of αCtx-AtIA; Peptide Sequence Identification

**Figure 2:**
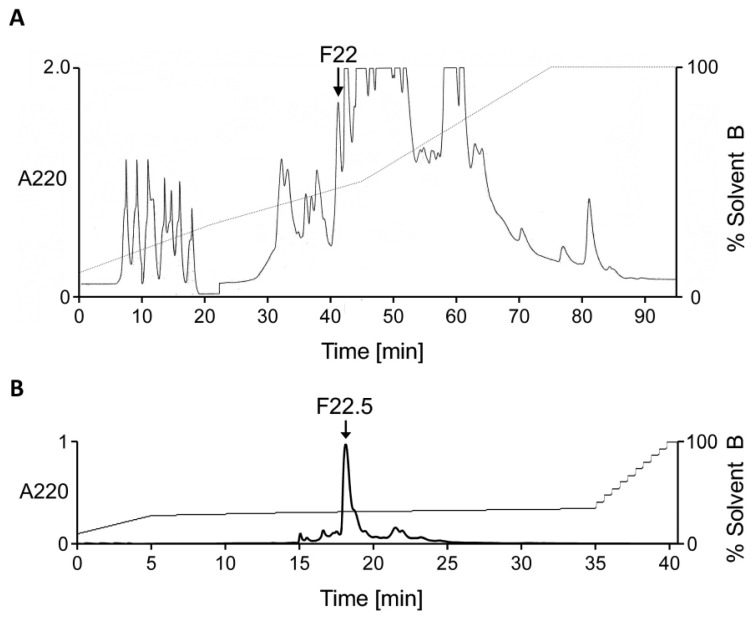
Bioassay-guided purification of fraction F22.5 from *C. ateralbus* venom. (**A**) A RP- HPLC chromatogram of *C. ateralbus* crude venom. The biological activity was first identified in fraction F22 and then sub-fraction F22.5. (**B**) Sub-fractionation of fraction F22 yielded a major peak (F22.5) containing a single peptide.

Specimens of *C. ateralbus* were collected in the shallow waters of the Calheta Funda Bay, Sal Island, and frozen at the collection site. In the laboratory, venom ducts were dissected, and the venom was pooled from a few specimens immediately after dissection and processed as described in detail in the Materials and Methods (Section 4.2). We used high-performance liquid chromatography (HPLC) to fractionate the venom into individual components and tested each fraction (Figure 2A) of the *C. ateralbus* venom extract in vivo. We detected multiple fractions that exhibited bioactivity and selected fraction F22 for further characterization, as detailed in Section 2.3 of the Results. The bioactivity of F22 (with retention time of 42 min on a C_18_ column) was also monitored in vitro in a calcium imaging-based Constellation Pharmacology platform, and it was found to modulate intracellular calcium levels in mouse dorsal root ganglion (DRG) neurons (Supplementary Figure S1). Thus, fraction F22 was further purified by C_18_-HPLC (Figure 2B), and a single peak was collected. Using Matrix-Assisted Laser Desorption/Ionization Time-of-Flight Mass Spectrometry (MALDI-TOF-MS), we obtained a molecular mass of 1954 Da (M+H)^+^. Chemical modification (reduction and alkylation) was used to estimate the number of Cys residues present. Reduction with dithiothreitol (DTT) and subsequent alkylation with 4-vinylpyridine resulted in a mass increment of 432 Da. This implied the presence of four Cys residues.

**Figure 3:**
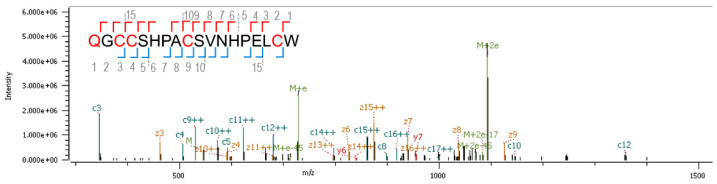
Sequence identification. MS/MS spectrum of the quadruple charge state of peptide QGCCSHPACSVNHPELCW recorded with Electron Transfer Dissociation (ETD) on an Orbitrap Elite. Observed N-terminal (c-type ions) and C-terminal (z-type ions) fragment ions are indicated in the sequence and annotated in the spectrum. Doubly charged ions are indicated with ++. M+e and M+2e indicate charge-reduced species with the number of electrons listed. The N-terminal Q is a pyroglutamate, and the cysteines were reduced and subsequently alkylated with methylaziridine (+57.0578).

The peptide sequence was identified by de novo sequencing (Figure 3). The N-terminal glutamine was observed as pyroglutamate. In position 16, the spectrum was consistent with either isoleucine or leucine. Due to the isobaric nature of these two amino acids, MS cannot differentiate between them. The leucine identity was verified after synthesis of both isoforms (see below) and co-elution on HPLC. 

### 2.2. Peptide Synthesis, Co-Elution and Cysteine Connectivity of α-Ctx AtIA

**Figure 4:**
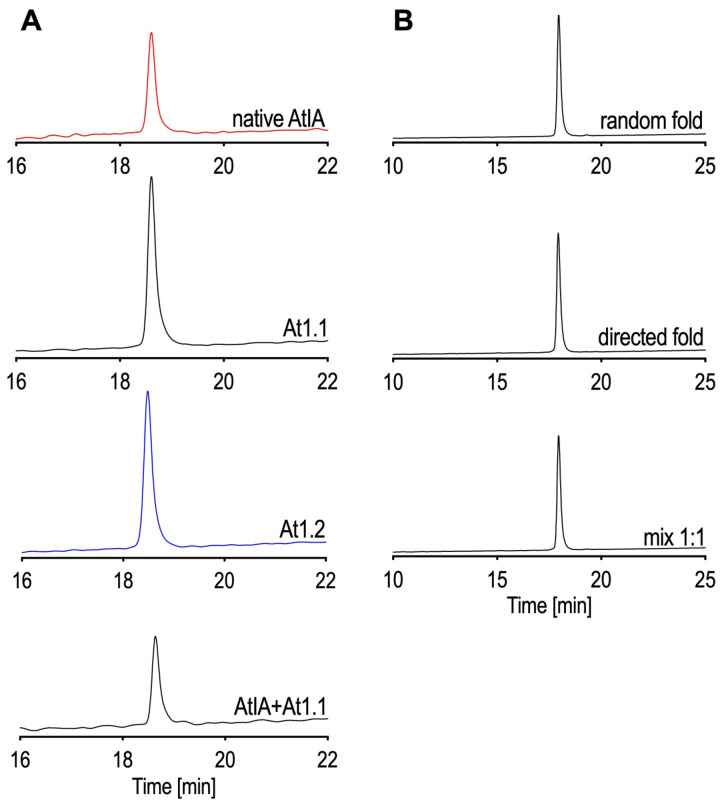
HPLC experiments with native and synthetic αCtx-AtIA. (**A**) Co-elution experiment of native αCtx-AtIA and the synthetic peptides. (**B**) Determination of the disulfide connectivity of At1.1 All experiments were performed using an analytical C18 column, a gradient of buffer B90 ranging from 20 to 50% buffer in 30 min, and a flow rate of 1 mL/min.

We used co-elution with the native material to resolve the amino acid at position 16. The peptide was synthesized in two forms: with Leu in position 16 as At1.1 (ZGCCSHPACSVNHPE**L**CW) and Ile in position 16 as At1.2 (ZGCCSHPACSVNHPE**I**CW). Both peptides were synthesized with all four Cys protected with a trityl (Trt) group and subjected to glutathione folding upon cleavage and purification of the linear forms. Each peptide folded into three different isomers, with the major product having a retention time close to that of the native peptide. Only At1.1 co-eluted with the native material (Figure 4A). To confirm that the native peptide had the globular, Cys1-Cys3 and Cys2-Cys4, connectivity, we re-synthesized At1.1 with Cys1-Cys3 Trt protected and Cys2-Cys4 S-acetamidomethyl (Acm) protected and folded it stepwise using glutathione to form the first disulfide bridge and iodine to form the second bridge. Due to the restricted amount of native material, we compared the retention time of the peptide obtained from random folding to the material obtained in the stepwise folding. Both peptides co-eluted as shown in Figure 4B. Therefore, we will refer to At1.1 as synthetic αCtx-AtIA.

### 2.3. Bioactivity of the Native and the Synthetic Peptides in Mice

Fraction F22 was injected intracranially (i.c.) in 15-day-old mice. Difficulty in walking was observed at 10 min post-injection. From 10 to 18 min, the fraction caused a consistent intense itch followed by death. The purified peptide (sub-fraction 22.5) caused similar behavioral responses as fraction F22. 

Synthetic αCtx-AtIA injected i.c. (5 nmol) in 15-day-old mice induced continuous rubbing and shaking of the head in the first 2–6 min, leading to impaired movement followed by death in the 15–35 min post-injection window (Table 1). When mice were injected with a lower dose (0.5 nmol) of αCtx-AtIA, no significant behavioral responses were observed.

### 2.4. Characterization of Synthetic αCtx-AtIA in DRG Neurons Using Constellation Pharmacology

**Figure 5:**
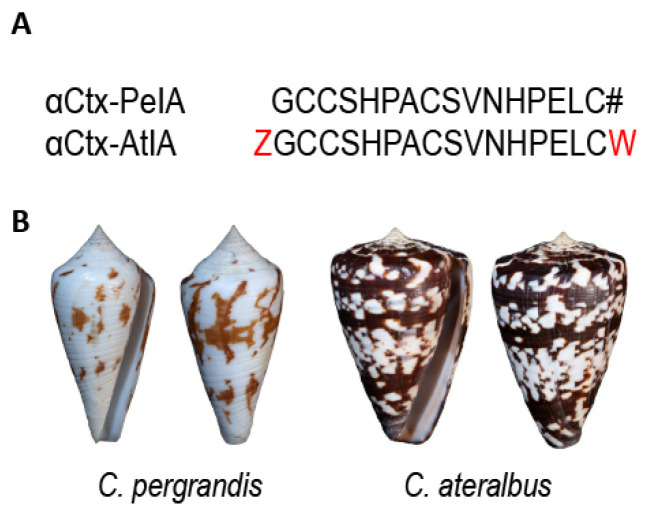
αCtx-PeIA and αCtx-AtIA sequence and shell comparison. (**A**) Residues that differ between the two peptides are indicated in red; # denotes C-terminal amidation. (**B**) Dorsal and ventral views of *C. pergrandis* and *C. ateralbus* shells.

To determine the molecular target of the peptide, we used the Constellation Pharmacology assay (see Materials and Methods Section 4.5). In this experiment, we used a calcitonin-gene-related peptide (CGRP)-GFP-labeled transgenic mouse that labels all the peptidergic nociceptive neurons in the DRGs. The application of F22.5 (purified native peptide) amplified KCl-induced calcium influx in 10% of the total DRG neurons (Supporting Figure S1). The synthetic peptide αCtx-AtIA also elicited similar phenotypic activity in ~4% of the DRG neurons when tested at 10 µM (Figure S2). Due to the pharmacological classification of this peptide as an αCtx, its further characterization was conducted on nAChRs expressed in mouse DRG neurons. However, due to the limited amount of the native peptide, comparative analysis of the native peptide with the synthetic peptide was not feasible. Thus, from then on, the synthetic version of the peptide was used for determining the ion channel target. Furthermore, because of the high sequence similarity between αCtx-AtIA and αCtx-PeIA (Figure 5), the experiments were designed to compare the activity of these two peptides on nAChR-expressing DRG neurons. 

In a primary cell culture, a subset of mouse DRG neurons responds to serial application of 1 mM acetylcholine (ACh) with elevation of intracellular calcium. This is primarily mediated by α3β4 and/or α6β4 receptor subtypes [23]. Another subset of DRG neurons responds to serial application of 1 mM ACh + 5 μM PNU-120596 (PNU; an α7 positive allosteric modulator) [24,25] mediated by the α7 subtype of nAChRs. 

To study the effect of αCtx on these neurons, two protocols were used. In protocol one, ACh pulses were applied twice, followed by the co-application of peptides with ACh to observe changes in the ACh responses. This protocol allowed the characterization of the peptide on nAChR containing α3, α6, and β4 subunits. In protocol two, ACh was applied first, followed by the application of ACh and PNU to reveal α7-nAChR mediated calcium responses. Subsequently, ACh, PNU, and the conotoxin of interest were co-applied to assess the impact of the conotoxin on α7 subunit-containing nAChRs. 

**Figure 6:**
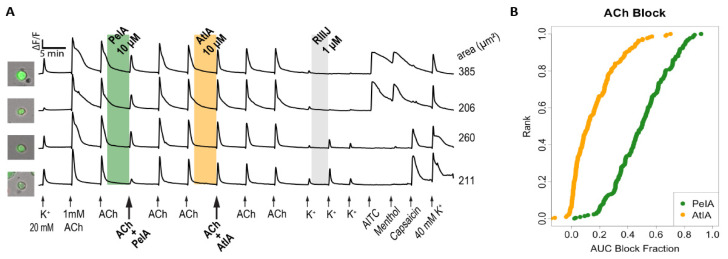
Differential effects of αCtx-AtIA and αCtx-PeIA on ACh-responsive DRG neurons. (**A**) Representative traces from four individual neurons. Shaded region indicates when the test peptide was present. (**B**) The reduction in calcium response (block) for individual cells due to αCtx-PeIA or αCtx-AtIA plotted as a cumulative distribution function (cdf) graph. Each cell is ranked on the *y*-axis according to the magnitude of block (*x*-axis).

The bioactivities of αCtx-PeIA and αCtx-AtIA were compared by monitoring the ACh-induced calcium influx signals before and after the application of αCtx-PeIA and αCtx-AtIA. Figure 6A shows traces of neurons in which 10 μM αCtx-PeIA inhibited ACh-induced calcium influx, while 10 μM αCtx-AtIA did not, suggesting that these two peptides have different selectivity for nAChR subtypes in DRG neurons. αCtx-PeIA showed potent inhibition of an α3-, α6-, and β4- containing nAChR subtypes, while αCtx-AtIA did not. The arrows in Figure 6A indicate applications of pharmacological stimuli that lasted 15 s. The column on the left shows a bright-field image of each cell overlaid with the fluorescent image obtained from green fluorescence protein expressed in the same neurons (to mark calcitonin gene-related peptide-expressing neurons). The *x*-axis indicates time, and the *y*-axis is the normalized ratio of 340/380 nm ([Ca]_i_ dual wavelength measurement). The initial 20 mM KCl application was to identify neurons, followed by 1 mM ACh applications to identify neurons expressing nicotinic receptors. The shaded area indicates the incubation of the peptides and other pharmacologic agents in the bath. Additional pharmacological stimuli such as 1 μM conotoxin kM-RIIIJ (RIIIJ), 100 μM allyl isothiocyanate (AITC), 400 μM menthol, and 40 mM KCl were applied to identify different cell types. Figure 6B displays the results from 220 ACh-responsive neurons obtained from six different experimental trials. Each neuron was ranked on the *y*-axis according to the magnitude of block by αCtx-AtIA on the *x*-axis (0 = no block, 1 = complete block). On average, αCtx-PeIA blocked 49.8% of the response magnitude while αCtx-AtIA inhibited only 11.5%. Figure S3A (Supplementary Materials) shows the count of ACh-responsive cells that were significantly blocked at an experiment-wide threshold of 0.05% (see Materials and Methods, Section 4.5). This illustrates the effects of the two peptides: 104 cells exhibited a significant block for both peptides, with a significantly lower potency for αCtx-AtIA, while 101 cells showed a significant block with αCtx-PeIA and a non-significant block with αCtx-AtIA.

**Figure 7:**
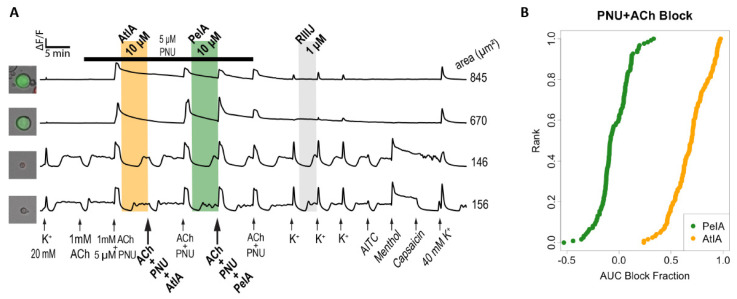
αCtx-AtIA inhibits α7-containing nAChR subtype in mouse DRG neurons. (**A**) Representative calcium imaging traces from four individual neurons. Shaded region indicates when the test peptide was present. (**B**) The reduction in calcium response (block) for all cells due to αCtx-PeIA or αCtx-AtIA plotted as a cumulative distribution function (cdf). Each cell is ranked on the *y*-axis according to the magnitude of block (*x*-axis).

Next, the activity of αCtx-AtIA on α7-subtype expressing DRGs was determined using protocol two (Figure 7). Application of ACh did not cause calcium influx in these cells; however, co-application of ACh and PNU uncovered a calcium response, suggesting the expression of α7 nAChR in these neurons (Figure 7A). PNU was applied to the bath solution for 33.5 min, as indicated by a black horizontal bar, and the cells were exposed to serial applications of 1 mM ACh+PNU at 7 min intervals. As illustrated in the figure, αCtx-PeIA did not inhibit the ACh+PNU-induced calcium influx, while αCtx-AtIA significantly blocked it. Figure 7B shows the quantitative measurements of αCtx-PeIA and αCtx-AtIA treatment on 114 ACh+PNU-responsive neurons obtained from four experimental trials. αCtx-PeIA did not inhibit the ACh+PNU-induced calcium influx, while αCtx-AtIA treatment blocked, on average, 69.5% of the response magnitude. Figure S3B (Supplementary Materials) illustrates the number of neurons that were significantly blocked at an experiment-wide threshold of 0.05%. Only 2 out of 114 neurons showed a block after applying αCtx-PeIA. In contrast, 60 out of 114 cells showed a significant block after applying αCtx-AtIA. The effect of each peptide on each cell was estimated from a multiple linear regression (see Materials and Methods).

#### 2.4.1. Synthetic αCtx-AtIA and αCtx-ArIB[V11L;V16D] Target the Same Cell Types

**Figure 8:**
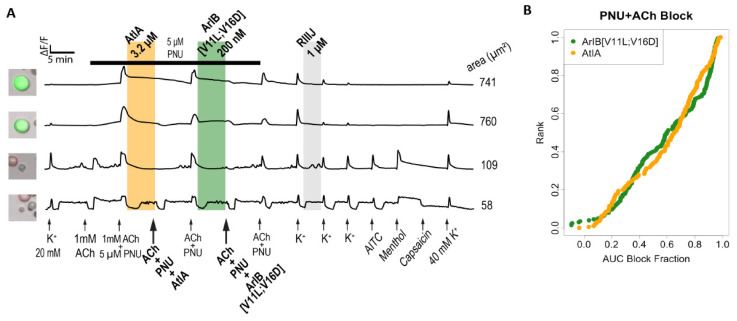
αCtx-AtIA shows a strong correlation with α7 nAChR antagonist αCtx-ArIB[V11L;V16D] in mouse DRG neurons. (**A**) Representative calcium imaging traces from four individual neurons. Shaded region indicates when the test peptide was present. (**B**) The reduction in calcium response (block) for all cells due to αCt-ArIB[V11L;V16D] or αCtx-AtIA plotted as a cumulative distribution function (cdf). Each cell is ranked on the *y*-axis according to the magnitude of block (*x*-axis).

αCtx-ArIB[V11L;V16D] is a very potent and selective blocker of α7 nAChRs [26] and has been routinely used as a pharmacological marker in calcium imaging assays to identify α7 nAChR expressing DRG neurons [23]. Following protocol two, we examined neurons that predominantly express the α7 receptor and compared the bioactivity of αCtx-AtIA and αCtx-ArIB[V11L;V16D] (Figure 8). Figure 8A shows examples of cells exhibiting a block from both αCtx-AtIA and αCtx-ArIB[V11L;V16D] while Figure 8B, represents the magnitude of the block for the 202 cells tested in this protocol. The counts of significantly blocked cells are shown in Figure S3C. The results showed that αCtx-AtIA inhibited the responses from α7 nAChRs in a similar manner to the known α7 antagonist αCtx-ArIB [V11L;V16D]. We established that at a concentration of as low as 3.2 µM, αCtx-AtIA blocked the calcium responses to the same degree as 10 µM, suggesting that 3.2 µM was a saturating concentration for peptide activity. 

### 2.5. Electrophysiology Data Confirm the Selectivity Profile of αCtx-AtIA for nAChRs

**Figure 9:**
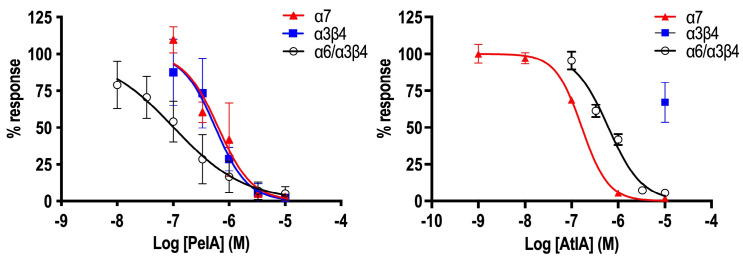
Effect of αCTx-PeIA and αCtx-AtIA on α7, α3β4 and α6/α3β4 nAChRs. Inhibition curves were obtained by the co-application of 100 μM (α3β4 and α6/α3β4) and 200 μM (α7) ACh with an increased concentration of α-CTx. A minimum of three oocytes were used for each IC_50_ determination, and the error bars indicate the SD.

Following the data obtained through Constellation Pharmacology, αCtx-PeIA and αCtx-AtIA were tested on the subtypes of mouse nAChR previously identified in DRG neurons, namely α7, α3β4, and α6β4, represented here as α6/α3β4 (a chimeric expression construct used to improve expression of an otherwise poorly expressing receptor subtype) (Table 2, Figure 9) [27]. αCtx-AtIA showed no activity on α3β4 when tested at 10 μM concentration and ~5-fold loss of potency on α6/α3β4 nAChRs when compared to αCtx-PeIA, which is consistent with the activity observed in DRGs. αCtx-PeIA blocked those subtypes with an IC_50_ = 0.57 µM and 0.1 µM, respectively. When tested on α7, αCtx-AtIA showed 4-fold higher potency than αCtx-PeIA, with an IC_50_ = 0.16 µM.

### 2.6. Contribution of the N-and C-Terminal Residues to the αCtx-AtIA Activity as Characterized by Constellation Pharmacology

**Figure 10:**
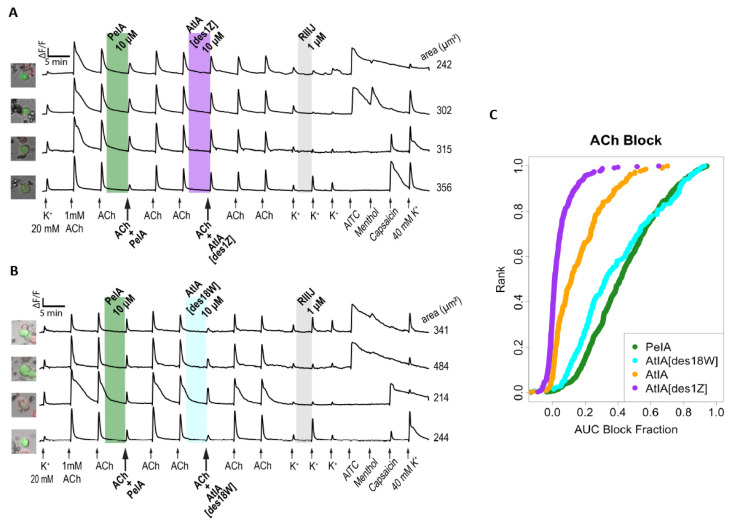
Effects of αCtx-AtIA[des1Z] and αCtx-AtIA[des18W] on ACh-responsive mouse DRG neurons. (**A**,**B**) The phenotypic effects of 10 μM αCtx-AtIA[des1Z] and αCtx-AtIA[des18W] in comparison to αCtx-PeIA and αCtx-AtIA on ACh-responsive neurons. Shaded region indicates when the test peptide was present. (**C**) The reduction in calcium response (block) for all cells due to application of indicated αCtx was plotted as a cumulative distribution function (cdf). Each cell is ranked on the *y*-axis according to the magnitude of block (*x*-axis).

**Figure 11:**
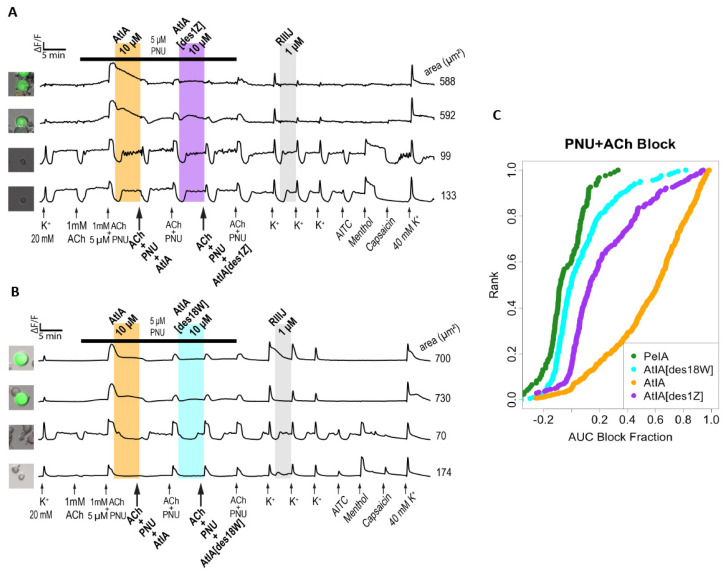
Effects of αCtx-AtIA[des1Z] and αCtx-AtIA[des18W] on ACh+PNU-responsive mouse DRG neurons. (**A**,**B**) The phenotypic effects of 10 μM αCtx-AtIA[des1Z] and αCtx-AtIA[des18W] in comparison to αCtx-AtIA on ACh+PNU-responsive neurons. (**C**) The reduction in calcium response (block) for all cells due to application of indicated αCtx was plotted as a cumulative distribution function (cdf). Each cell is ranked on the *y*-axis according to the magnitude of block (*x*-axis).

Because αCtx-AtIA and αCtx-PeIA differ by two amino acid residues, analogs with N-terminal truncation αCtx-AtIA[des1Z] and C-terminal truncation αCtx-AtIA[des18W] were synthesized and tested using Constellation Pharmacology protocol one (Figure 10) and protocol two (Figure 11). As illustrated in Figure 10A, removal of residue Pyr1 (αCtx-AtIA[des1Z]) reduced the activity of the peptide and ACh-induced calcium responses were moderately blocked, with only 28 out of 351 cells displaying a significant inhibition of the ACh response (Figure S3D). 

Deletion of the Trp18, on the other hand, yielded an analog αCtx-AtIA[des18W] that blocked the ACh responses similar to αCtx-PeIA (Figure 10B); 199 of 253 cells showed significant block from both peptides (Figure S3D) at a comparable magnitude (Figure 10B). As shown in Figure 10C, analog αCtx-AtIA[des18W] was almost as potent as αCtx-PeIA in blocking ACh induced calcium responses while αCtx-AtIA[des1Z] displayed the weakest potency with little to no blocking of the ACh response. When both analogs were tested using protocol two, the bioactivity of αCtx-AtIA[des18W] was similar to that observed for αCtx-PeIA, with minimal blocking of the ACh+PNU responses (Figure 11B). In contrast, αCtx-AtIA[des1Z] behaved more like the parent peptide but displayed weaker activity (Figure 11A). The magnitude of blocking by αCtx-AtIA[des1Z] was lower than that of the parent peptide (Figure 11C) but still highly correlated (Figure S3E). Taken together, our results suggest that the N-terminus truncation reduced the potency of the peptide and C-terminal truncation reduced the subtype selective activity of the native peptide. 

## 3. Discussion

*Conus ateralbus* is a species of cone snail within the *Kalloconus* subgenus found in the Cabo Verde archipelago off the coast of West Africa. It is known to feed primarily on marine worms. Thus far, only one conotoxin has been identified from this species: δ-AtVIA, which was shown to be a vertebrate sodium channel blocker [15]. Here, we report the discovery and characterization of the first αCtx targeting nAChRs from the venom of *C. ateralbus*. αCtx-AtIA shares a high sequence identity (16 of 18 amino acids) with αCtx-PeIA and differs only at the N- and C-terminus with the pyroglutamate and tryptophan residues, respectively, and a free carboxyl at the C-terminus. The native peptide was purified from the venom and its sequence was determined by manual de novo sequencing. Two versions of the peptide were chemically synthesized and co-eluted with the native material to resolve the Ile/Leu uncertainty in position 16. Intracranially injected native αCtx-AtIA and its synthetic version evoked similar behavioral responses in 15-day old mice. At 5 nmol, αCtx-AtIA caused seizures followed by death, a phenotype previously observed for αCtx-ImI as well as α-bungarotoxin by McIntosh et al. [28]. Both peptides were shown to target the α7 subtype of nAChRs. Therefore, we hypothesized that the effect induced by αCtx-AtIA in mice might be mediated via α7 nAChRs. 

Because of the heteromeric complexity and diversity of nAChRs, the development of either agonists or antagonists of nicotinic receptors as therapeutic drugs or diagnostic reagents has met with many challenges. Indeed, the nAChRs antagonist that had reached Phase II human clinical trials eventually failed, primarily because the subtype selectivity and affinity of the compound for nicotinic receptors had not been sufficiently defined when the clinical trials were initiated [29,30,31,32]. In this work, we introduce a novel experimental protocol for the discovery and characterization of new nAChR ligands. By customizing our Constellation Pharmacology platform, we were able to assess the bioactivity of αCtx-AtIA on nAChR-expressing DRG neurons and determine the selectivity profile for different nAChR subtypes expressed in DRGs.

Initially, fractions of the *C. ateralbus* venom were tested and their effects were monitored on KCl-induced calcium influx as an indicator of biological activity. αCtx-AtIA was found to modulate baseline calcium levels as well as amplifying KCl-induced calcium influx in a small subset of DRG neurons. These phenotypic responses were recapitulated with the synthetic peptide. However, as αCtx-AtIA belongs to the A superfamily of conotoxins that target nAChRs, we sought to characterize its effects on specific nAChRs using dedicated protocols.

In 2013, Smith et al. classified mouse and rat DRG neurons into four categories based on the functional expression of nAChRs: those that do not express nAChRs, those expressing predominantly α7 nAChRs, those expressing predominantly α3β4 and α6β4, and those expressing all three receptor types [23]. This is consistent with the observed DRG neuronal response to ACh in mice: most neurons do not respond, a subset responds repeatedly and consistently to 1 mM ACh, (α3β4 and α6β4), another subset responds repeatedly and consistently to ACh+PNU, (α7) and a fourth type responds to both ACh and ACh+PNU. A manuscript with a detailed description of the cell-type-specific functional distribution of AChR subtypes in the DRG neurons is currently being prepared by Tun et al. (unpublished data).

To confirm the data collected in DRG neurons, we mainly focused on testing the peptides on these subtypes of nAChRs using electrophysiology. The data collected in DRGs suggested a significant difference in activity between αCtx-AtIA and αCtx-PeIA. When tested in ACh+ neurons expressing α3β4 and α6β4, only αCtx-PeIA produced a substantial block at 10 μM, whereas αCtx-AtIA exhibited inhibitory characteristics at the same concentration on DRGs expressing α7 nAChRs. The same trend was observed when both peptides were tested on heterologously expressed mouse α7, α3β4 and α6/α3β4 nAChRs. αCtx-AtIA blocked mouse α7 with an IC_50_ = 0.16 µM but did not produce block at 10 µM on α3β4 and was ~six times less potent on α6/α3β4 than αCtx-PeIA. 

As the sequences of the two peptides differ only by two residues, we investigated the impact of removing residue Pyr1 or Trp18 on the activity of αCtx-AtIA. Pyroglutamate (Z, Pyr) is a cyclic amino acid identified in many biologically active peptides and proteins [33] including A-, M- and T-superfamilies of conotoxins [1]; it was found to affect the stability of the peptides, and in some cases, also their activity. In conotoxins, a pyroglutamate residue was used to improve the stability of χ-MrIA [34], a small NET inhibitor [35], without affecting its potency, advancing the peptide to clinical trials for neuropathic pain treatment [36]. When pyroglutamate was removed from conotoxin μ-SIIIA, a potent Na_v_ channel blocker, only minor changes in affinity for Na_V_1.2 and Na_V_1.4 of VGSCs were observed [37], suggesting that the residue had more of a stabilizing effect rather than contributing to the activity of the peptide. The bulky, hydrophobic tryptophan (W; Trp) residue is known to form noncovalent interactions via π-π, cation-π, and X-H-π, as well as forming hydrogen bonds. It not only stabilizes the structure of the peptide, but mostly contributes to the peptide–protein or protein–protein interaction [38]. We synthesized both analogs of the peptide and used Constellation Pharmacology to compare their activity to either parent αCtx-AtIA or αCtx-PeIA. As shown in Figure 10, in the ACh+ DRG neurons, removal of Trp18 led to an analog with an activity similar to that of αCTX-PeIA, while removal of Pyr1 did not change the activity of the analog as compared to the parent peptide. This result suggests an unfavorable interaction between Trp18 of αCtx-AtIA and residues of the α3β4 nAChRs and α6β4. It is possible that the orientation of the bulky C-terminal Trp18 interferes with the efficiency of binding to the receptor by the residues in the second inter-cysteine loop, which was previously shown to be important for the potency and selectivity of αCtx-PeIA on those subtypes [39]. When conotoxins AtIA[des1Z] and AtIA[des18W] were tested in the ACh/PNU+ DRG neurons (Figure 11), both peptides exhibited minimal inhibitory effect similar to that of αCtx-PeIA, although the peptide with the N-terminal truncation was more potent than the C-terminal truncation analog. 

In summary, based on the extensive sequence homology between αCtx-AtIA and αCtx-PeIA, we anticipated that the subtype selectivity profile of the two peptides would be comparable, if not identical. However, using Constellation Pharmacology, we demonstrated that there were significant differences in the bioactivity of the two peptides. We also characterized analogs of αCtx-AtIA to understand the molecular basis for the differences observed in the selectivity profiles. The Constellation Pharmacology assessment revealed the role of the C-terminal Trp residue in conferring differences in target specificity. The demonstration that a single amino acid can be such a powerful determinant of subtype-selectivity, essentially abolishing affinity for the α3β4 subtype, while concomitantly increasing the affinity for the α7 subtype, may be useful for future drug development programs. Through this work, we have demonstrated the potential broad applicability of Constellation Pharmacology in defining the target selectivity of nicotinic receptor ligands.

## 4. Materials and Methods

### 4.1. Phylogenetic Analysis

A dataset composed of concatenated 12S, 16S and cytochrome oxidase I (COI) gene segments from each species was used for phylogenetic analysis. These gene segments were cloned and sequenced as previously described [14,40]. Multiple sequence alignment was carried out using MAFFT version 7 [41]. Maximum likelihood tree reconstruction was performed with IQ-TREE, consisting of best-fit model TIM3+F+I+G4 using ModelFinder [42] and ultrafast bootstrap approximation [43]. The tree was visualized using iTOL version 6.4 (https://itol.embl.de, accessed on 27 February 2024) [44]. Clades were collapsed and are represented by circles with sizes proportional to the number of species in each clade. The alignment file has been included as Supplementary Data File S1.

### 4.2. Venom Preparation

*C. ateralbus* specimens (length ≥ 40 mm) were collected from shallow waters around Sal Island (Calheta Funda, Cabo Verde) by SCUBA snorkeling. The specimens were frozen at −20 °C. The specimens were carefully dissected to remove the venom gland. The venom was obtained from venom ducts by placing each duct on an ice-cold metal spatula and squeezing the contents into 40% acetonitrile (CH_3_CN) /water acidified with 0.1% trifluoroacetic acid (TFA). The solution was then lyophilized and stored at −80 °C. Crude venom extract was prepared using 40% (*v*/*v*) CH_3_CN/water acidified with 0.1% (*v*/*v*) TFA and a portion of the extract was resuspended in 15 mL of 40% CH_3_CN and 0.1% TFA and vortexed twice for 1 min each, with a pause of 3 min in between. The samples were homogenized in a Wheaton homogenizer and centrifuged in a Beckman Avanti centrifuge (F650 rotor) for 15 min at 13,650 rpm, at 4 °C. All residual particles were retained by centrifugation. 

#### 4.2.1. Peptide Isolation (RPLC Purification)

Reverse-phase high-performance liquid chromatography (RP-HPLC) was carried out using a C_18_ Vydac, 218TP101522, 50 mm × 22 mm, 10–15 µm particle size; eluted with a flow rate of 7 mL/min and a gradient ranging from 10% to 30% of buffer B in 20 min, 30% to 50% in 25 min, 50% to 100% in 30 min followed by 100% for 15 min. Buffer B was 0.1% (*v*/*v*) TFA in 90% aqueous CH_3_CN, and buffer A was 0.1% (*v*/*v*) TFA in water.

All fractionations were purified using a C_18_ Vydac Monomeric (238EV54, 250 mm × 4.6 mm, 5 µm particle size). The absorbance was monitored at 220 and 280 nm and all fractions were stored at −80 °C. 

### 4.3. Peptide Sequence Determination

The crude HPLC fractions were analyzed using Matrix-Assisted Laser Desorption/Ionization Time of Flight (MALDI-TOF, Applied Biosystems, Foster City, CA, USA) mass spectrometry at the Mass Spectrometry Core, Salk Institute for Biological Studies, La Jolla, CA, USA. To determine the number of cysteines, a DTT reduction and 4-vinylpyridine alkylation was employed on the pure peptide. To 177 µL of the pure peptide (HPLC sub-fraction 22.5), 10 µL of 0.5 M Tris base (pH 7.5) and 52 µL of 50 mM DTT were added, the reaction mixture was gently vortexed, and the solution was incubated at 65 °C for 30 min. Next, 1.6 µL of 4-vinylpyridine was added, and the reaction was mixed and kept in the dark for ~20 min at RT. Finally, the reaction mixture was diluted with at least 2 volumes of the HPLC buffer A and was subjected to LC separation with fraction collection, using the same gradient described for sub-fractionation of the venom. For comparison, a blank run was conducted using the reduction–alkylation solution but without including the peptide. 

#### 4.3.1. De Novo Sequencing

An aliquot of the HPLC sub-fraction 22.5 was dried in a SpeedVac, and subsequently reduced and alkylated in vapor using 50% CH_3_CN, 1% 2-methylaziridine and 2% trimethylphosphine in 100 mM ammonium bicarbonate pH 8.4 (*v*:*v*), for 90 min at room temperature (RT). The alkylation vapor was removed, and the sample was dissolved in 10 μL of 0.5% acetic acid. Aliquots were loaded onto a 0.2 × 25 cm Pepswift EasySpray column. The sample was eluted at a flow rate of 1μL/min with a gradient (solvent A: 0.5% acetic acid; solvent B: 90% (*v*/*v*) CH_3_CN in 0.5% acetic acid) of 0% B-45%B in 30 min, and 45–100% B in 5 min, a spray voltage of 2.5kV on an Easy nLC-1000 nanoUHPLC coupled with an Orbitrap Elite mass spectrometer. MS1 scans were acquired at 120,000 resolution (@ 400 *m*/*z*). MS2 was acquired for the top 4 precursors that carry at least 2 charges using the following settings: 4 microscans, 2 *m*/*z* isolation window, and a target value of 5 × 10^4^ ions. Each precursor was subjected to ETD and HCD fragmentation using the following conditions: 15,000 resolution (@ 400 *m*/*z*), ETD using 80 ms ion reaction time, and HCD using 27% normalized collision energy. The measured mass deviates from the theoretical mass by 4.1 ppm and is within the mass error of the instrument. The sequence was obtained by manual de novo sequencing.

### 4.4. Peptide Synthesis

The synthesis of PeIA has been previously described [16]. The novel peptides described in this study, At1.1 and At1.2 (the analog with an L16I mutation), AtIA[des1Z], and AtIA[des18W], were synthesized using solid-phase Fmoc peptide chemistry with an AAPPTec Apex 396 automated peptide synthesizer (Louisville, KY, USA). Peptides were constructed either on Fmoc-Trp(Boc)-Wang resin (substitution: 0.29 mmol/g) or Fmoc-Cys(Acm)-Wang resin (substitution: 0.62 mm/g). All standard amino acids were purchased from AAPPTec and the side-chain protection for the following amino acids was as follows: Ser: *O*-*tert*-butyl, Asn, His, Cys: trityl (Trt) and/or Cys: acetamidomethyl (Acm) (Acm), Glu: *tert*-butyl (tBu), Trp: tert-butyloxycarbonyl (Boc). Initially, At1.1 and At1.2 were synthesized with all Cys protected with Trt groups to facilitate random folding after the cleavage of the peptide from the resin. The peptides were synthesized at 50 μmol scale. Coupling activation was achieved with 1 eq of 0.4 M benzotriazol-1-yl- oxytripyrrolidinophosphonium hexafluorophosphate and 2 eq of 2 M *N*,*N*-diisopropylethyl amine in *N*-methyl-2-pyrrolidone as the solvent. For each coupling reaction, a 10-fold excess of amino acid was used, and the reaction was carried out for 60 min. Fmoc deprotection was performed for 20 min with 20% (*v*/*v*) piperidine in dimethylformamide. 

#### 4.4.1. Peptide Cleavage and Purification

The peptides were cleaved from the resin using Reagent K, consisting of trifluoroacetic acid (TFA)/phenol/H_2_O/thioanisol/ethanedithiol (82.5/5/5/5/2.5 by volume) (Fisher Scientific (Hampton, NH, USA), Millipore Sigma (Burlington, MA, USA), Millipore Sigma and Acros Organics (Geel, Belgium), respectively). Next, the cleavage mixture was filtered and precipitated with 10 mL of cold methyl-tert-butyl ether (MTBE; Fisher Scientific (Hampton, NH, USA)). The crude peptide was then precipitated by centrifugation at 7000× *g* for 7 min and washed twice with 10 mL cold MTBE. The crude peptide was diluted with 50 mL of 10% CH_3_CN in buffer A and purified by reverse-phase (RP) HPLC using a semi-preparative C18 HiChrom column (218TP510, 250 × 10 mm, 5 μm particle size) eluted with a linear gradient ranging from 20 to 50% buffer B90 in 30 min with a flow rate 4 mL/min for semi-preparative purification. The following HPLC buffers were used: 0.1% (*v*/*v*) TFA in water (buffer A) and 0.1% TFA (*v*/*v*) in 90% aqueous CH_3_CN (Fisher Scientific) (*v*/*v*) (buffer B90). The eluent was monitored at 220/280 nm. The purity of the peptides was assessed by analytical C18 Vydac RP-HPLC (218TP54, 250 × 4.6 mm, 5 μm particle size) using the same gradient as described above with a flow rate 1 mL/min. Peptides were quantified by UV absorbance at 280 nm, using an extinction coefficient (*ε*) value of 5500 M^−1^·cm^−1^ For the analogs with all Cys protected with Trt: out of 53 mg of the resin, 2800 nmols of the linear At1.1 and 2000 nmols of At1.2 out of 50 mg (both with 95%) purity were prepared.

#### 4.4.2. Oxidative Folding of At1.1 and At1.2 in the Presence of Reduced and Oxidized Glutathione

Eight hundred nanomoles of linear At1.11 in 5.6 mL of HPLC buffer mixture was added to a solution containing 20 mL of 0.2 M Tris·HCl (pH 7.5) plus 0.2 mM EDTA, 0.5 mL of 10 mM reduced and 4 mL of 10 mM oxidized glutathione, and 10.4 mL of water. The final peptide concentration in the folding mixture was 20 μM. The folding reaction was conducted for 3.5 h and quenched with formic acid to a final concentration of 8%. The quenched reaction mixture was then separated by RP-HPLC using a semi-preparative C_18_ column and a linear gradient ranging from 20% to 50% of buffer B90 in 30 min with a flow rate of 4 mL/min. The eluent was monitored by absorbance at 220/280 nm. The purity of the folded peptide was assessed by an analytical C_18_ Vydac RP-HPLC using the gradient described above, with a flow rate 1 mL/min. Pure fully folded At1.11 was quantified by absorbance at 280 nm, as described for the linear peptide. Next, 270 nmols of the major product was isolated in 34% yield with 96% purity. The same method was used to obtain fully folded At1.2 in 33% yield and 96% purity. The identities of At1.1 and At1.2 were confirmed by MALDI MS (calculated monoisotopic MH^+1^: 1949.90, determined monoisotopic MH^+1^: 1949.71 for At1.1 and MH^+1^: 1949.91, determined monoisotopic MH^+1^: 1949.71 for At1.2, at the University of Utah Mass Spectrometry and Proteomics Core Facility. 

#### 4.4.3. Peptide Co-Elution

Solutions of the synthetic peptides At1.1 and At1.2 were prepared in 10% CH_3_CN in buffer A. Peptides were analyzed individually on the analytical C18 column using HPLC with a gradient ranging from 20% to 50% buffer B in 30 min, with a flow rate of 1 mL/min. Their elution times were compared to the native material. Since At1.1 had the same retention time as the native material, they were mixed and re-injected on the column to produce a uniform peak (Figure 4).

#### 4.4.4. Synthesis of the Globular At1.1 (αCtx-AtIA)

To confirm that the active form of the peptide is globular, At1.1 was resynthesized as described above with Cys3 and Cys9 Trt-protected and Cys4 and Cys17 protected with Acm. The cleavage protocol was the same as described earlier. For the purification of the linear peptide, it was applied to a semi-preparative C18 Vydac column (218TP510, 250 × 10 mm, 5 μm particle size) and eluted with a linear gradient ranging from 20 to 50% buffer B90 in 30 min with a flow rate 4 mL/min. The HPLC buffers were 0.1% (*v*/*v*) TFA in water (buffer A) and 0.1% TFA (*v*/*v*) in 90% aqueous CH_3_CN for the semi-preparative purification. Out of 24 mg of the resin, 500 nmol of the linear peptide was obtained, with purity ranging from 79 to 87%.

#### 4.4.5. First Disulfide Bond Formation (^3^Cys-^9^Cys)

Three hundred nanomoles of linear At1.1 [4,17Cys(Acm)] in 1.5 4.4 mL of HPLC buffers mixture was added to a solution containing 7.5 mL of 0.2 M Tris·HCl (pH 7.5) plus 0.2 mM EDTA, 1.5 mL of 10 mM 1:1 mixture of the reduced and oxidized glutathione, and 4.5 mL of water. The final peptide concentration in the folding mixture was 20 μM. The folding reaction was conducted for 2 h and quenched with formic acid to a final concentration of 8%. The quenched reaction mixture was then separated by RP-HPLC using a semi-preparative C_18_ column and a linear gradient ranging from 20% to 50% of buffer B90 in 30 min with a flow rate of 4 mL/min. The monocyclic At1.1 was obtained with 63% yield and with 90% purity. The identity of the peptide was confirmed by MALDI MS: calculated monoisotopic MH^+1^: 2093.81, determined MH^+1^: 2093.87. Additional masses representing sodium and potassium adducts were also present: M+Na^+^: 2115.88 and M+K^+^: 2131.85.

#### 4.4.6. Second Disulfide Bond Formation (^4^Cys-^17^Cys)

Removal of the acetamidomethyl groups and the second disulfide bridge formation was accomplished by iodine oxidation. First, 10 mg of iodine (Acros Organics, Antwerp, Belgium) was added to 5 mL of CH_3_CN and stirred until completely dissolved. Then, 15 mL of nanopure water was added, followed by 0.6 mL of TFA. The monocyclic At1.1 solution of 192 nmol in 475 µL of the HPLC buffer was dripped into 475 µL of the iodine solution (prepared as described above) and allowed to react for 5 min at room temperature. The reaction was quenched by adding 50 µL of 1 M freshly prepared ascorbic acid (0.176 g, 1 mmol; Research Products International, Mount Prospect, IL, USA) solution in water until the reaction mixture became transparent. The reaction was then diluted to 5 mL with buffer A and subsequently purified by RP-HPLC using a semipreparative C18-column and a linear gradient ranging from 20% to 50% of buffer B90 in 30 min with a flow rate of 4 mL/min. The peptide was quantified by UV absorbance at 280 nm, using an extinction coefficient (*ε*) value of 5500 M^−1^·cm^−1^. At1.1 (αCtx-AtIA) was obtained in 25% yield and with 98% purity. The identity of the peptide was confirmed by ESI MS: calculated monoisotopic MH^+1^: 1949.90, determined monoisotopic MH^+1^: 1949.71

#### 4.4.7. One-Pot, Two-Step Folding of AtIA[des18W] and AtIA[des1Z]

To 5 mL of the solution of 1000 nmol of crude linear AtIA[des18W] in 90% glacial acetic acid, 5% water, and 5% methanol in a small Erlenmeyer flask equipped with a magnetic stirring bar, a few drops of 40 mM iodine solution in methanol were added until a pale-yellow color appeared. Next, 5 mL of 50 mM HCl was added, followed by 2 mL of 40 mM iodine solution in methanol. The reaction was stirred for 1h and then quenched with 1 M freshly prepared ascorbic acid, diluted with water to 35 mL, and purified by RP-HPLC using C18 column and a gradient ranging from 10% to 40% in 30 min with a 4 mL/min flow. The peptide was quantified by analytical HPLC by comparing the area under the peak of the peptide vs. a reference peptide. The same method was used to synthesize AtIA[des1Z]. AtIA[des18W] was obtained in 42% with 98% purity and AtIA[des1Z] in 13% with 97% as determined by the analytical RP-HPLC. The identities of the peptides were confirmed by ESI MS: AtIA[des18W] calculated monoisotopic MH^+1^: 1763.65, determined monoisotopic MH^+1^: 1763.64; AtIA[des1Z] calculated monoisotopic MH^+1^: 1838.69, determined monoisotopic MH^+1^: 1838.69.

### 4.5. Constellation Pharmacology Assay

We used 37–42 days old transgenic mice (Tg(Calca-EGFP)104Gsat) obtained from our collaborator (Ginty Lab, Harvard University) in all experiments. Primary cell cultures from mouse dorsal root ganglia (DRG) were prepared as previously described [45]. Briefly, lumbar DRG, L1-L6, were removed, trypsinized, and plated on a 24-well poly-D-lysine-coated plate. The plated cells were placed in a 95% O2−5% CO_2_ incubator at 37 °C overnight in a minimum essential medium (MEM) supplied with fetal bovine serum (FBS) and supplements. Then, 10–12 h after the primary cell culture preparation, the dissociated cells were loaded with 1mM Fura-2-AM dye for 1 h at 37 °C before the Constellation Pharmacology experiments were performed. In experimental trials, individual responses from ~1000 neurons were simultaneously monitored under 4× objective. 

During the venom components screening, aliquots of HPLC venom fractions from *C. ateralbus* were assayed for activity on DRG neurons that were exposed to two 15 s depolarizing control stimuli (20 mM KCl) that temporarily elevated [Ca^2+^]_i_ as measured by calcium imaging. The protocol included several control pulses of 20 mM KCl, followed by incubation with a venom fraction. The responses from the venom fractions during and after the incubation of venom were categorized as direct responses (direct calcium influx during venom fraction incubation) and indirect effects (amplification calcium response elicited by 20 mM KCl following the venom fraction incubation) that indicated pharmacological activity.

During the synthetic peptide characterization, for the mouse DRG Constellation Pharmacology experiments, intracellular calcium changes were monitored for response to the application of pharmacological stimuli 100 μM allyl isothiocyanate (AITC), 300 nM capsaicin, 40 mM KCl, 400 μM menthol, and 1 μM RIIIJ, 5 μM PNU-120596 (PNU), 1mM acetylcholine (ACh), 10 μM of αCtx-PeIA, 10 μM synthetic αCtx-AtIA, αCtx-AtIA[des1Z] and αCtx-AtIA[des18W]. Two experimental protocols were used to test the bioactivity effects of the synthetic αCtx-AtIA. Protocols one and two used different cocktails to characterize the bioactivity of conotoxins: 

Protocol one used a cocktail containing 1mM ACh and 10 μM of peptide of interest: αCtx-PeIA, αCtx-AtIA, αCtx-AtIA[des1Z], or αCtx-AtIA[des18W].

Protocol two used two different cocktails, one containing 1mM ACh + 5 μM PNU, and other containing 1mM ACh + 5 μM PNU + 10 μM of peptide of interest: αCtx-PeIA, αCtx-AtIA, αCtx-AtIA[des1Z] or αCtx-AtIA[des18W], or 200 nM of ArIB[V11L;V16D]. 

Both protocols one and two began with 7 min intervals then shifted to 5 min intervals (after the 20 mM KCl application) when neuronal cell classification pharmacological stimuli were applied.

#### 4.5.1. Constellation Pharmacology Data Analysis

A single calcium imaging experiment measured the intracellular calcium concentration (ICC) for 100s–1000s of cells at 2 s intervals over a time course up to 2 h. During the experiment specific modifications were made to the cell culture to test for induced changes in ICC. The ICC values for each cell across the entire experiment were referred to as the calcium response phenotype (CRP) of the cell. These responses were plotted as a time series in Figure 6, Figure 7, Figure 8, Figure 10, and Figure 11. Then, 1mM ACh or 1mM ACh+PNU was added at regular intervals to induce a CRP. The CRP is very specific for each cell but was conditional upon a complex set of cellular variables. Cells that showed a repeated strong response to ACh were assumed to be expressing ACh receptors other than α7 (primarily α3β4 and α6β4). These cells were used to test the cellular response to ACh only. Cells that showed no response to ACh alone but showed a repeated strong response to ACh+PNU were assumed to be expressing the α7 nicotinic receptor (CHRNA7) and were used to test the cellular response to stimulation of that receptor. We tested for activity of a compound of interest by incubating the cell culture in the compound between ACh/ACh+PNU pulses and estimating the change in the phenotype of the specified cells. A change in the ICC that occurred while the compound was in the well was referred to as a direct effect (DE). A change that altered the CRP after incubation was referred to as an indirect effect (IDE). 

The degree of inhibition induced by pre-incubation with each peptide was estimated by comparing a full model with simple linear model. The block fraction was estimated as the 1-f/l, where f was the predicted magnitude of response in the full model (accounting for the effects of the peptide) and l was the predicted magnitude of response in a null linear trend model. These values were plotted as cumulative distributions in Figure 6, Figure 7, Figure 8, Figure 10, and Figure 11. 

#### 4.5.2. Estimation of Indirect Effects

Each experiment had a series of ACh- and/or ACh+PNU-induced CRPs. The magnitude of the CRP was measured as the maximum area under the curve for any 15 s interval. The deviation of this response after a test compound incubation was used to estimate the effect of the compound. We estimated this effect on each neuron using a multiple linear regression (the lm function in R) with lm(auc15 ~ linear + compound), where auc15 was the maximum area under the curve for any 15 s interval for each ACh pulse, linear was the trend coded as sequential integers, and compound was an indicator variable for the CRP immediately following incubation with the compound of interest. The Tstat values from the coefficients matrix were taken as estimates of the magnitude and direction of the indirect effects (IDE).

#### 4.5.3. Controlling for Multiple Tests Estimating Significance

Each experiment gave results for 100s–1000s of cells. To control for false positives and set appropriate thresholds for significance, we used Monte Carlo simulations. Each simulation generated random numbers drawn from a normal distribution with mean and standard deviations of the actual data. The Tstats were estimated for all cells and recorded. This was considered a null distribution. The Tstat estimates from 100 Monte Carlo simulations were used to establish the 0.05 experiment-wide threshold for single-cell significance. The counts of cells that exceed this threshold were used to define the contingency tables in Figure 6, Figure 7, Figure 8, Figure 10, and Figure 11. 

### 4.6. Biological Activity in Mice

Fraction 22 and all sub-fractions were assayed in Swiss Webster mice. Each sample was resuspended in 12 µL of saline solution (0.9% NaCl) before intracranial injection into mice (15 days old—weight average 8.90 ± 0.84 g). The injection was performed using a 1 mL insulin syringe, and the same volume of normal saline solution was injected into the control mice. The mice were observed for at least 1 h, as previously described [46]. The same protocol was followed for fraction 22.5 and the synthetic peptide αCtx-AtIA (i.c. injections in 15- to 18-day old mice (weight 9.07 ± 0.74 g)). 

### 4.7. Oocyte Electrophysiology

Frogs were purchased from Xenopus1 (Dexter, MI, USA) and maintained by University of Utah personnel in an AAALAC accredited facility. Oocytes were obtained from frogs anesthetized with 0.4% wt/vol Tricaine-S (Thermo Fisher Scientific, Waltham, MA, USA) and were sacrificed after removal of the ovarian lobes.

Methods for the preparation of cRNA constructs for expression of nAChRs in *X. laevis* oocytes have been previously described [47]. Clones for expression of mouse α3, β4, α6 and α7 subunits were provided by J. Stitzel, University of Colorado, Boulder, CO, USA. An α6/α3 chimera was used to enable expression of nAChRs with the α6 subunit [27]. Briefly, stage IV–V oocytes were injected with equal ratios of cRNAs encoding nAChR subunits and subjected to two-electrode voltage clamp (TEVC) electrophysiology 1–5 days after injection. The oocyte membranes were clamped at a holding potential of −70 mV, and 200 μM acetylcholine (ACh) was applied at 60 s intervals for a duration of 1s. For the assessment of peptide activity, the oocytes were continuously perfused with frog saline (control solution) and pulsed with ACh until a stable baseline response was observed, then the saline was switched to a solution containing the peptide and the ACh responses were monitored for changes in amplitude. The ACh responses in the presence of peptide were normalized to the average of three responses in control solution. Peptides were applied in this manner for concentrations ≥1 μM. For concentrations >1 μM, the peptides were applied in a static bath for 5 min and normalized to the ACh response after a 5 min bath application of the control solution.

#### 4.7.1. Statistical Analysis for Electrophysiology

All statistical analyses were performed using Prism 8 (GraphPad Software, San Diego, CA, USA). The estimated IC_50_ values for inhibition of ACh-evoked currents by the peptides were obtained by nonlinear regression using a four-parameter logistic equation and presented with the corresponding 95% CI to evaluate the precision of the IC_50_ estimate. The error bars represent the SD of the data obtained at each concentration and are provided to assess the variance of the data.

## Figures and Tables

**Table 1:** Behavior observed when synthetic αCtx-AtIA was injected i.c. in 15- to 18-day old mice. Average weights of the mice are indicated with standard deviation.

αCtx	Amount Injected (nmol)	*n*	Weight (g)	Observation
Control	0	5	9.84 ± 2.38	Normal, grooming, walking
Synthetic AtIA	0.5	3	7.32 ± 0.18	Exploring, walking, grooming; one mouse showed splayed legs at 14 min; one mouse showed hiccup-like movements at 19 and 70 min
Synthetic AtIA	5	3	9.33 ± 1.22	Wobbling/rolling while rubbing its head; shaking with consistent head shaking every few seconds (2–6 min); seizures (9–35 min; paralysis and death (15–35 min); one mouse showed scratching and biting of foot at 19–25 min

**Table 2:** Effects of α-conotoxins PeIA and AtIA on selected subtypes of mouse nAChRs.

αCtx	Sequence	Subtype of m nAChRs	IC_50_ (95% CI) ^1^
PeIA	G**CC**SHPA**C**SVNHPEL**C**# ^2^	α3β4	0.57 (0.4–0.8) µM
AtIA	ZG**CC**SHPA**C**SVNHPEL**C**W	α7	0.64 (0.4–0.9) µM
		α6/α3β4	0.1 (0.07–0.16) µM
		α3β4	>10 µM
		α7	0.16 (0.15–0.19) µM
		α6/α3β4	0.62 (0.52–0.73) µM

^1^ Data were collected by applying 200 μM ACh to *Xenopus* oocytes heterologously expressing the receptor. The IC_50_ values and 95% confidence intervals (CI) were obtained using 3–5 separate oocytes. ^2^ # denotes C-terminal amidation.

## Data Availability

The data presented in this study are available on request from the corresponding author (accurately indicated status).

## Supplementary Materials

The following supporting information can be downloaded at https://www.mdpi.com/article/10.3390/md22030118/s1, Figure S1: Biological activity of the native peptide in fraction F22.5 observed in calcium-imaging traces from selected DRG neurons.; Figure S2: Effects αCtx-AtIA on induced 20 mM KCl calcium influx pulses; Figure S3: The ACh or ACh+PNU block count tables.; Dataset S1: Phylogenetic marker alignment file.
