# Supplementary material for: Using Constellation Pharmacology to Characterize a Novel α-Conotoxin from Conus ateralbus
Source: Mar Drugs. 2024 Feb 29;22(3):118. doi: 10.3390/md22030118 (PMC10971446; doi:10.3390/md22030118)
Supplement: Supplementary file 1 [file marinedrugs-22-00118-s001.zip › marinedrugs-2873120-supplementary.pdf]

Supplementary Materials

Using Constellation Pharmacology to characterize a novel α-conotoxin from *Conus ateralbus*

Jorge L. B. Neves [1,2], Cristoval Urcino[2], Kevin Chase[2], Cheryl Dowell[2], Arik J. Hone[2,3], David Morgenstern[4], Victor M. Chua[2], Iris Bea L. Ramiro[2,5], Julita S. Imperial[2], Lee S. Leavitt[2], Jasmine Phan[2], Fernando A. Fisher[2], Maren Watkins[2], Shrinivasan Raghuraman[2], Jortan O. Tun[2], Beatrix M. Ueberheide[4], Michael McIntosh[2,6,7], Vitor Vasconcelos[1,8], Baldomero M. Olivera[2], Joanna Gajewiak[2,*]

1 Interdisciplinary Centre of Marine and Environmental Research (CIIMAR/CIMAR-LA), University of Porto, Terminal de Cruzeiros do Porto de Leixões, Avenida General Norton de Matos, S/N, 4450-208 Matosinhos, Portugal

2 School of Biological Sciences, University of Utah, Salt Lake City, UT 84112 USA

3 Mental Illness Research Education and Clinical Center George E. Whalen Veterans Affairs Medical Center, Salt Lake City, UT 84148, USA

4 Departments of Biochemistry and Molecular Pharmacology, New York University Langone Medical Center, New York, NY 10016, USA;

5 The Marine Science Institute, University of the Philippines, Quezon City 1101, Philippines

6 Department of Psychiatry, University of Utah, Salt Lake City, UT 84108, USA

7 Mental Health Department, George E. Whalen Veterans Affairs Medical Center, Salt Lake City, UT 84148, USA

8 Faculty of Sciences, University of Porto, Rua do Campo Alegre, 4169-007 Porto, Portugal

* Correspondence: jgajewiak@gmail.com.

Figure S1. Biological activity of the native peptide in fraction F22.5 observed in Calcium-imaging traces from selected DRG neurons. Each trace represents the intracellular calcium concentration (y-axis) of a single neuron over the time course of the experiment (x-axis). Arrows on the x axis mark 15-s applications of 20 mM KCl (K^+) to depolarize the neurons. The horizontal bar indicates when the venom fraction was present in the bath solution. The pure venom fraction (F22.5) caused an enhancement of the amplitude and duration of the response to a depolarizing stimulus, which was reversible.

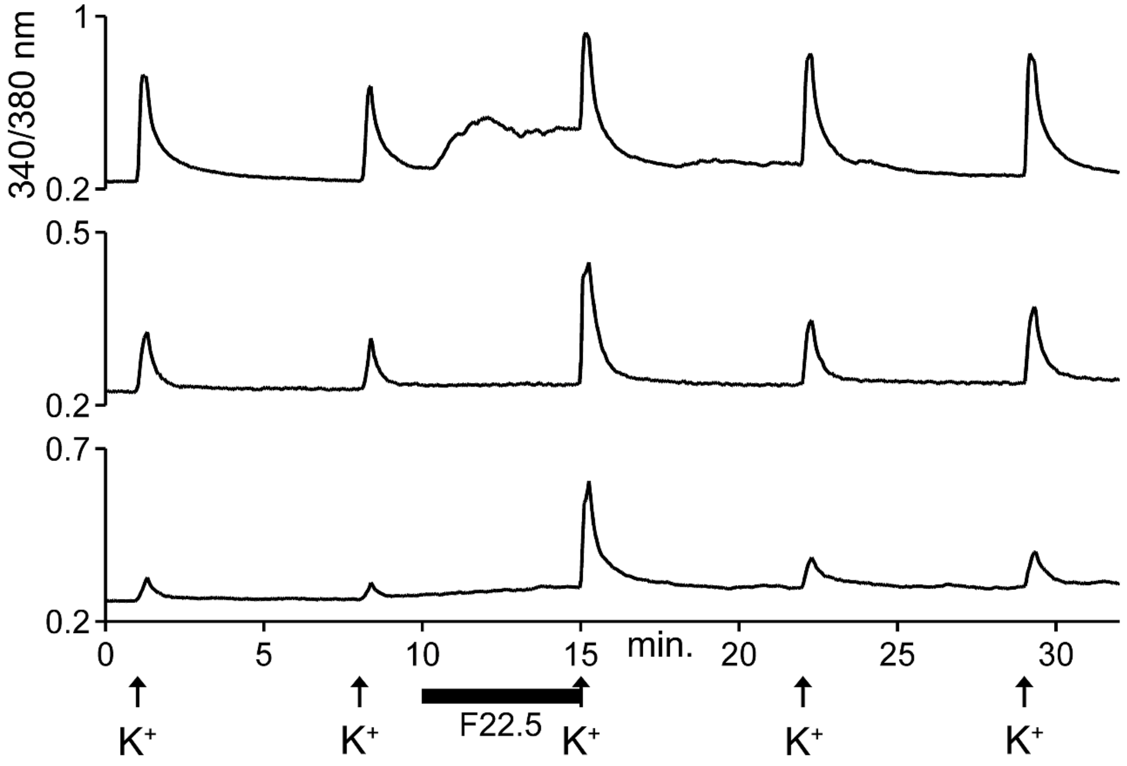

Figure S2. Effects αCtx-AtIA on induced 20 mM KCl calcium influx pulses. Cell images and calcium imaging traces are shown for 6 representative neurons showing an αCtx-AtIA-induced amplified response to a depolarizing stimulus of 20 mM KCl. The calcium imaging protocol begins with two control 20 mM KCl pulses (7 min intervals between all pulses), an incubation of 10 μM αCtx-AtIA, followed by a pulse of 20 mM KCl + 10 μM αCtx-AtIA. The remaining pulses, as well as cell fluorescence and morphology, were used to classify cell types. Approximately 4% of total neurons displayed this amplification effect from the peptide. These cells were classified as medium diameter peptidergic and non-peptidergic nociceptors.

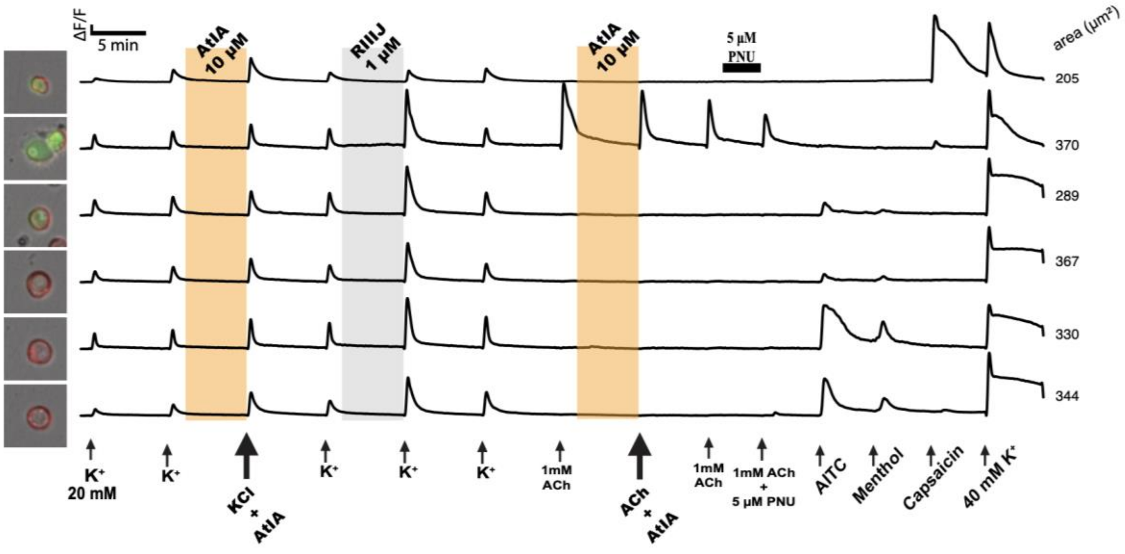

Figure S3. The ACh or ACh+PNU block count tables. Each 2x2 table shows the number of cells not blocked (left column) or blocked (right column) by the peptide listed as the column header and the counts for the same cells that were not blocked (top row) or blocked (bottom row) with the peptide listed as the row header. Only cells tested by both peptides in the same experiment are shown in these contingency tables. The number of experiments for each pair of peptides are: (**A**) n=6 (**B**) n=4 (**C**) n=5 (**D**) AtIA x PeIA n=6, AtIA[des18W] x PeIA n= 4, AtIA[des1Z] x PeIA n=6 (**E**) AtIA x PeIA n=4, AtIA[des18W] x PeIA n= 4, AtIA[des1Z] x PeIA n=7.

A

ACh block count		
	AtIA	
PeIA	11	4
	101	104

B

ACh+PNU block count		
	AtIA	
PeIA	54	58
	0	2

C

ACh+PNU block count		
	ArIB [V11L;V16D]	
AtIA	104	24
	25	49

D

ACh block count		
	PeIA	
AtIA	11	101
	4	104
AtIA [des18W]	33	11
	10	199
AtIA[des1Z]	35	288
	0	28

E

ACh+PNU block count		
	AtIA	
PeIA	54	58
	0	2
AtIA [des18W]	80	85
	1	38
AtIA[des1Z]	157	27
	4	57

File S1. Fasta file of aligned sequences used for phylogenetic analysis.

>ebraeus
AACCAAGAAATTTAGTCATTCTTTTACGGTAAAAAGACAGTTAAGCAGG-TAGAATCATT
AGATCTTCAATATAGGAGTAAAATCTAAATATTGGAAGTTATACCTAACGTGGTTT-ATT
TATGCTGAAGCTGTGATAGCTTTAAGGGAAACTGGGATTGGATACCCCATTATTTTTAGT
CGTAAATTTAGTT--TAAGCTTACCAGAGTACTACGAATGTTTAAAACTCAAAGGGCTTG
GCGGTGTTTTAGACCTCTCAGGGGAACCTGTCTCATAATCGACAATCCACGTTAAACCTG
ACTCTTTGTGGCAAGTCAGCCTGTATACCGTCGTCGTCAGGTAACTTCTTAAAATATAGT
AGTTAGCTAGAGAATT--AT-ATTAATTAGAACGTCAGATCAAGGTGCAGCTAATAAAAG
AGTGAGGATGGGTTACAATTACAGATTTGTAATTACGGCACT-TGAAAT-AAGTGTCTTG
AAGGAGGACTTGAAAGTAATTTTGAGTATATAAACAAAATGAATAAGGCTCTGAAACGTG
CAGAATTTTAATGGTCGAACAGACCAACCCTCAAAGACTTCTGCATCTTTAGGATATTCT
GGTCCAACATCGAGGTCACAAACCTTTTTTTCGATATGGGCTCTCAAAAAAGATAATGCT
GTTATCCCTACGGTAACTAATTCCTTTGATCAAAATTCT-TGGATCCTC-TCAAGTAAGA
CTTC-AAGGGAGGCTTTATCTACTCCTCGGTTGCCCCAACCAAAGT--ATTTAATAGCTT
T-TTTTTTA-GTTAATT---AAATACTCCATTAATTTTTCTGAAGCTCGATAGGGTCTTC
TTGTCTTTTAATAATATCTGGGCTTTTTCGCCCAAAGATAAAATTCTAAATAATCTAAAA
GAGACAGGTGTATTCTTGTCAAACCATTCATTCCAGCCTTCAATTATAAGGCAAATGATT
ATGCTACCTTTGCACGGTCAGAGTACCGCGGCCGTT-TAAAC-CACTGGGCAGGTCCGAC
TTCGCATTTGAT-GATTACACGACATTGTATATTTTATTTGGGATATGGTCCGGTTTGGT
CGGAACTGCCCTGAGATTGCTAATTCGTGCAGAGTTAGGACAACCGGGAGCCCTACTTGG
AGATGATCAGCTGTATAACGTGATTGTAACAGCCCATGCTTTTGTTATAATTTTTTTCTT
AGTGATACCTATGATGATTGGTGGTTTCGGAAACTGACTAGTACCACTAATATTAGGAGC
TCCAGATATGGTATTTCCTCGGTTAAATAATATAAGTTTCTGGTTACTTCCTCCTGCACT
TTTACTTCTCTTATCTTCAGCCGCGGTAGAGAGTGGAGTGGGTACGGGATGAACGGTGTA
TCCACCTCTATCAGGAAACTTAGCACATGCCGGTGGGTCAGTAGATCTGGCAATTTTTTC
TTTACACCTTGCCGGGGTTTCTTCTATTTTAGGAGCGGTGAATTTTATTACTACAATTAT
TAATATGCGATGACAAGGGATAAAATTTGAACGTCTTTCGCTATTTGTGTGGTCAGTAAA
AATTACAGCTATTTTACTTTTACTGTCTCTTCCTGTGTTAGCGGGGGCAATTACGATGCT
TTTGACAGATCGAAATTTTAATACTGCCTTCTTCGACCCAGCAGGAGGTGGTGATCCAAT
CTTGTATCAGCACTTGTTT
>chaldeus
AACCAAGAAATTTAGTCATTCTTTTACGGTAAAAAGACAGTTAAGCAGG-TGAGATCATC
AGATCTTCAATATAGGAGTAAAATCTAAATATTGAAAGTAATGCCTAACGTGATTT-ATT
TATGCTGAAGCTGTGATAGCTTTAAGGGAAACTGGGATTGGATACCCCATTATTTTTAGT
CGTAAATTTAGTA--TAAGCTTACCAGAGTACTACGAATGTTTAAAACTCAAAGGGCTTG
GCGGTGTTTTAGACCTCTCAGGGGAACCTGTCTCATAATCGACAATCCACGTTAAACCTA
GCTCTTTATGGCAAGCCAGCCTGTATACCGTCGTCGTCAGGTAACTTCTTAAAATATAGT
AGTTAGCTAGAGAATT--AT-ATTAATTAAAACGTCAGATCAAGGTGCAGCTAATAAAAG
AGTGAGGATGGGTTACAATTACAGATTTGCAATTACGGCACT-TGAAAT-AAGTGTCTTA
AAGGAGGACTTGAAAGTAATTTTGATTATATAAACAAAATGAATAAGGCTCTGAAACGTG
CAGAATTTTAATGGTCGAACAGACCAACCCTCAAAGACTTCTGCATCTTTAGGATATTCT
GGTCCAACATCGAGGTCACAAACCTTTTTTTCGATATGGGCTCTCAAAAAAGATAATGCT
GTTATCCCTACGGTAACTAATTCCTTTGATCAAAATTCT-TGGGTCCTC-ACAAGTAAGA
CTTC-AAAGGAGGCTTTGTCTACTCCTCGGTTGCCCCAACCAAAGT--ATTTAATAGCCT
T-TTTTTTA-ATTAATT---AAACACTCTATTAATTTTTCTGAAGCTCGATAGGGTCTTC
TTGTCTATTAATAAAATCTGGGCTTTTTCACCCAAAGATAAAATTCTAAATAATCTAAAA

GAGACAGGTGTATTCTTGTCAAACCATTCATTCCAGCCTTCAATTATAAGGCAAATGATT
ATGCTACCTTTGCACGGTCAGAGTACCGCGGCCGTT-TAAAC-CACTGGGCAGGTCCGAC
TTCGCATTTGAT-AATTACACGACATTGTATATTTTATTTGGGATATGGTCCGGTCTGGT
TGGGACTGCCTTGAGATTGTTAATTCGTGCAGAATTAGGACAACCGGGAGCCCTCCTGGG
AGATGATCAGCTGTATAACGTAATTGTGACGGCTCATGCTTTTGTTATAATTTTTTTCTT
AGTGATACCTATGATGATCGGTGGTTTTGGAAATTGATTAGTACCATTAATATTAGGGGC
TCCAGATATAGTATTTCCTCGATTAAATAATATAAGTTTCTGGTTACTTCCTCCTGCTCT
TTTACTTCTATTATCTTCGGCTGCAGTAGAAAGTGGAGTGGGTACAGGATGAACGGTGTA
TCCGCCTCTATCAGGAAATTTAGCACATGCTGGTGGGTCAGTAGATCTGGCAATTTTTTC
TTTACACCTTGCTGGGGTTTCTTCTATTTTAGGAGCAGTAAACTTTATTACTACAATTAT
TAATATGCGATGACAAGGAATAAAATTTGAACGTCTTTCGCTATTCGTGTGATCAGTAAA
GATTACGGCTATTTTACTTTTACTGTCTCTCCCTGTGTTAGCAGGGGCAATTACAATGCT
TTTGACGGATCGAAATTTTAATACTGCTTTCTTCGATCCAGCAGGAGGTGGTGATCCGAT
CTTATATCAGCACTTGTTT
>coronatus
AACCAAGAAATTTAGTCATTCTTTTACGGTAAAAAGACAGTTAAGCAAG-CGAAATCATT
AGATCTTTAATATAGGAGTAAAATCTAAATATTGAAAGTTATACCTAGCGTGATTT-ATT
TAAGCTGAAGCTGTGATAGCTTTAAGGGAAACTGGGATTGGATACCCCATTATTTTTAGT
CGTAAATTTAGTT--TAGGCTTACCAGAGTACTACGAATGTTTAAAACTCAAGGGGCTTG
GCGGTGTTTTAGACCTCTCAGGGGAACCTGTCTCGTAATCGACAATCCACGTTAAACCTA
ACTCTTTATGGCAAGTCAGCCTGTATACCGTCGTCGTCAGGTAACTTCTTAAAATATAGT
AGTTAGCTCGAGAATT--TC-ATTAATTAAAACGTCAGATCAAGGTGCAGCTAATAAAAG
AGTGAGGATGGGTTACAATTACAAATTTGTAATTACGGCACT-TGAAACGAAGTGACTTA
AAGGAGGACTTGAAAGTAATTTTGATTATATAAACAAAATGAATAAGGCTCTGAAACGTG
CAGAATTTTAATGGTCGAACAGACCAACCCTCAAAGACTTCTGCATCTTTAGGATATTCT
GGTCCAACATCGAGGTCACAAACCTTTTTTTCGATATGGGCTCTCAAAAAAGATAATGCT
GTTATCCCTACGGTAACTAATTTCTTTGATCAAAATTCT-TGGATCCTC-ACAAGTAAGA
CTTC-AAAGGAGGCTTTGTTTACTCCTCGGTTGCCCCAACCAAAGT--ATTTAATAGCTT
T-CTTTTTA-ACCAATT---AAAATCTCTATTAATTTTTCTGAAGCTCGATAGGGTCTTC
TTGTCTTTTAATACAATCTGGGCTTTTTCACCCAAAGATAAAATTCTAAATAATCTAAAA
GAGACAGGTGTATTCTTGTCAAACCATTCATTCCAGCCTTCAATTATAAGGCAAATGATT
ATGCTACCTTTGCACGGTCAGAGTACCGCGGCCGTT-TAAAC-CACTGGGCAGGTCCGAC
TTCGTATTTAAC-GACTACACGACATTGTACATTTTATTTGGAATATGATCTGGCTTAGT
TGGGACTGCCTTGAGGTTACTAATCCGTGCAGAATTAGGACAACCGGGAGCCCTGCTTGG
AGACGATCAGTTGTATAATGTAATTGTAACAGCCCATGCTTTTGTTATAATTTTTTTCTT
AGTGATACCTATGATGATTGGTGGCTTTGGGAACTGATTGGTGCCACTAATATTAGGGGC
TCCAGACATGGTATTTCCTCGTTTAAATAATATGAGTTTCTGGTTACTTCCTCCTGCTCT
TTTACTTCTTTTATCTTCAGCTGCAGTAGAAAGTGGGGTGGGTACTGGATGAACAGTATA
CCCTCCTTTATCAGGAAACTTGGCGCATGCTGGTGGGTCAGTAGATCTGGCAATTTTCTC
TTTGCATCTTGCCGGAGTTTCTTCTATTTTAGGGGCTGTAAATTTTATTACTACAATTAT
TAATATGCGATGACAAGGAATAAAATTTGAACGTCTTTCGCTGTTTGTGTGATCAGTAAA
GATTACAGCTATTTTACTTTTACTGTCTCTTCCTGTCTTAGCAGGAGCAATTACAATACT
TTTAACGGATCGAAACTTTAATACTGCTTTCTTTGACCCAGCAGGAGGTGGTGACCCTAT
CTTGTATCAGCACTTGTTT
>miliaris
AACCAAGAAATTTAGTCATTCTTTTACGGTAAAAAGACAGTTAAGCAAG-GGAAATCATT
AGACCTTTAGTATAGGAGTAAAATCCAAATATTAAAAGTTATACCCAACGTGGTTT-ATT
TATGCTGAAGCTGTGATAGCTTTAAGGGAAACTGGGATTGGATACCCCATTATTTTTAGT

CGTAAATTTAGTT--CAAGCTTACCAGAGTACTACGAATGTTTAAAACTCAAAGGGCTTG
GCGGTGTTTTAGACCTCTCAGGGGAACCTGTCTCATAATCGACAATCCACGTTATACCTA
ACTCTTTATGGCAAGTCAGCCTGTATACCGTCGTCGTCAGGTAACTTCTAAAAATATAGT
AGTTAGCTCGAGAACT--TT-ATTAGTTAAAACGTCAGATCAAGGTGCAGCTAATAAAAG
AGTGAGGATGGGTTACAATTACAGATTTGTAATTACGGCACT-TGAAATGAAGTGACTTA
AAGGAGGACTTGAAAGTAATTTTGATTATATAAGCAAAATGAATAAGGCTCTGAAACGTG
CNNNNNNNNNNNNNNNNNNNNNNNNNNNNNNNNNNNAGACTTCTGCATCTTTAGGATATTCT
GGTCCAACATCGAGGTCACAAACCTTTTTTTCGATATGGGCTCTCAAAAAAGATAATGCT
GTTATCCCTACGGTAACTAATTCCTTTGATCAAAATTCT-TGGATCATC-TCAAGTAAGA
CTTC-AAAGGAGGCTTTATTTACTCCTCGGTTGCCCCAACCAAAGT--ATTTAATAGCTT
T-TTTTTTA-ATTAATT---AAGTACTCTATTAATTTTTCTGAAGCTCGATAGGGTCTTC
TTGTCTTTTAATAAAATCTAGGCTTTTTCACCCAGAGATAAAATTCTAAATAATCTAAAA
GAGACAGGTGTATTCTTGTCAAACCATTCATTCCAGCCTTCAATTATAAGGCAAATGATT
ATGCTACCTTTGCACGGTCAGAGTACCGCGGCCGTT-TAAAC-CACTGGGCAGGTCCGAC
TTCGTATTTAAT-GCTTACACGACATTGTATATTTTATTTGGGATATGATCTGGCTTGGT
TGGGACTGCCCTAAGATTGCTAATTCGTGCAGAGTTAGGACAGCCGGGAGCTTTGCTTGG
AGATGATCAGCTATATAATGTGATTGTAACAGCCCATGCTTTTGTTATAATTTTTTTCTT
AGTGATACCTATAATGATTGGTGGTTTTGGTAATTGATTAGTGCCGCTGATGTTAGGGGC
TCCAGATATGGTGTTTCCTCGTTTAAATAACATAAGTTTCTGGTTACTCCCTCCTGCCCT
TCTACTTCTTTTATCTTCAGCTGCGGTAGAAAGCGGAGTGGGTACGGGATGAACGGTGTA
TCCACCTTTATCAGGAAACTTGGCACATGCCGGCGGGTCTGTAGATCTGGCAATTTTCTC
TTTACACCTTGCTGGGGTTTCTTCTATTTTAGGAGCAGTAAACTTTATTACTACAATTAT
CAATATACGATGACAAGGAATAAAATTCGAACGTCTTTCGCTATTTGTGTGGTCAGTAAA
AATTACGGCTATTCTGCTTCTGCTGTCTCTTCCTGTGTTAGCAGGAGCAATTACAATGCT
TTTAACGGATCGAAATTTTAATACTGCTTTCTTCGACCCAGCAGGAGGTGGTGACCCTAT
CTTATATCAGCACTTGTTT
>ventricosus
AACCAAGAAATTTAGTCATTCTTTTATGGTAAAAAGACAGTTAAGTAAA-AAAAGTCGTT
AGATCTTTAGTATAGGAGTAAAATCCATGTACTAAAAGTTATACCGAATATGATTT-AGT
TATGCTGAAGCTGTGATAGCTTTAAGGGAAACTGGGATTGGATACCCCATTATTTTTAGC
TGTAAATTTAGTT--TAAGCTTACCAGAGTACTACGAATGTTTAAAACTCAAAGAGCTTG
GCGGTGTTTTAGACCTCTCAGGGGAACCTGTCTCATAATCGACAATCCACGTTAAACCTA
ACCTTTGATAGTAATTCAGCCTGTATACCGTCGTCGTCAGGTAACTTCTCAAAATATAGT
AGTTAGCTTGAGAATT--AT-ATTAATTAAAACGTCAGATCAAGGTGCAGCTAATGAAAA
GGTGAGGATGGGTTACAATTACATATTTGTAAACACGGCATT-TGAAA--AAATGTCTTA
AAGGAGGACTTGAAAGTAATTTTGATTATATAAGCAGAATGAATAGGGCTCTGAAACGTG
CAGAATTTTAATGGTCGAACAGACCAACCCTTAAAGACTTCTGCATCTTTAGGATATTCT
GGTCCAACATCGAGGTCACAAACCTTTTTTTCGATATGGGCTCTTGAAAAAGATAATGCT
GTTATCCCTACGGTGACTAATTCCTTTGATCAAAATTTCTTGGATCAAC-ACAAGTAGGA
TTTA-AGAGGAAGCTTTATTTGTTCCTCGGTTGCCCCAACCAAAGT--ATTTAATAGCTT
T-TCTTTTA-CTTAATTG--ACAAGGTCTACTAACTTCTCTAAAGCTCGATAGGGTCTTC
TTGTCTTTTAATGCTATCTGGACTTTTTCATCCAAAAATAAAATTCTAAATAATCTAAAA
GAGACAGGTGTATTCTTGTTAAACCATTCATTCCAGCCTTCAATTATAAGGCAAATGATT
ATGCTACCTTTGCACGGTCAGAGTACCGCGGCCGTT-TAAAA-CACTGGGCAGGTCCGAC
TTCGTATTTAAT-TTTAACACGACATTATATATTTTATTTGGGATGTGATCTGGGTTGGT
TGGAACTGCCCTTAGGTTACTGATTCGTGCAGAATTAGGTCAACCGGGTGCCTTACTTGG
AGATGATCAATTATATAATGTGATTGTGACAGCCCATGCTTTTGTTATAATTTTTTTCTT
AGTGATGCCTATAATAATTGGGGGTTTTGGAAACTGGTTGGTACCTTTGATGTTAGGGGC

TCCAGATATGGTATTTCCTCGCTTAAATAATATAAGTTTTTGACTTCTTCCCCCTGCACT
TCTACTTCTTTTATCATCAGCTGCTGTGGAAAGTGGGGTAGGTACAGGATGAACGGTATA
TCCACCTTTGTCTGGAAATTTGGCTCATGCTGGTGGATCAGTAGATTTGGCGATTTTTTC
TTTACATCTTGCTGGTGCGTCTTCTATTTTAGGAGCGGTGAATTTTATTACTACAATTAT
TAATATACGATGACAAGGGATAAAATTTGAACGTCTTTCGTTATTTGTATGATCAGTAAA
GATTACAGCTATTTTACTCCTTTTATCTCTACCTGTGTTAGCAGGTGCAATCACTATACT
TTTAACTGATCGAAATTTTAATACTGCTTTCTTTGATCCAGCAGGAGGTGGAGATCCTAT
TTTATATCAGCATCTATTT
>dorotheae
AACCAAGAAATTTAGTCATTCTTTTATGGTAAAAAGACAGTTAAGCAGG-AAAAGTCATT
GGACCTTTAATATAGGAGTAAAATCTAGATACTAAAAGTTATATCAGACATGGCTT-AAT
TATGCTGAAGCTGTGATAGCTTTAAGGGAAACTGGGATTGGATACCCCATTATTTTTAGT
TGTAAATTTAGTT--TAAGCTTACCAGAGTACTACGAATGTTTAAAACTCAAAGAGCTTG
GCGGTGTTTTAGACCTCTCAGGGGAACCTGTCTCATAATCGACAATCCGCGTTAAACCTA
ACCTTTGATAGCAATTCAGCCTGTATACCGTCGTCGTCAGGTAACTTCTTAAAATATAGT
AGTTAGCTTGAGAATT--TT-ATTAATTAAAACGTCAGATCAAGGTGCAGCTAATGAAAG
GGTGAGGATGGGTTACAATTACACATTTGTAAATACGGCATT-TGAAAT--AATGTCTTA
AAGGAGGACTTGAAAGTAATTTTGATTATATAAATAGAATGAATAGGGCTCTGAAACGTG
CAGAATTTTAATGGTCGAACAGACCAACCCTTAAAGACTTCTGCATCTTTAGGATATTCT
GGTCCAACATCGAGGTCACAAACCTTTTTTTCGATATGGGCTCTTGAAAAAGATAATGCT
GTTATCCCTACGGTAACTAATTCCTTTGATCAAAATTTC-TGGATCAAC-ACAAGTAAGA
TTTA-AGAGGAGGCTTTGTCTGCTCCTCGGTTGCCCCAACCAAAAT--ATTTAATAGCTT
T---TTTTA-CTTAATTA--ATGAAGTCTACTAACTTTTCTAAAGCTCGATAGGGTCTTC
TTGTCTTTTAATACTATCTGGACTTTTTCATCCAGAAATAAAATTCTAAATAATCTAAAA
GAGACAGGTGTATTCTTGTCAAACCATTCATTCCAGCCTTCAATTATAAGGCAAATGATT
ATGCTACCTTTGCACGGTCAGAGTACCGCGGCCGTT-TAAAA-CACTGGGCAGGTCCGAC
TTCGTATTTAAT-ATTAACACGACATTGTATATTTTATTTGGGATATGATCTGGGTTGGT
TGGAACCGCCCTTAGATTACTAATTCGTGCAGAGTTAGGTCAACCGGGTGCCTTACTTGG
AGATGATCAGTTATATAATGTAATTGTAACAGCCCATGCTTTTGTTATAATTTTTTTCTT
AGTGATGCCTATGATGATTGGGGGTTTTGGAAACTGGTTGGTGCCTTTAATGTTAGGAGC
TCCAGATATGGTATTTCCTCGTTTAAATAATATAAGTTTCTGGCTTCTTCCTCCTGCACT
TTTACTTCTTCTATCATCAGCTGCTGTGGAAAGTGGGGTAGGTACAGGATGAACTGTGTA
TCCGCCTTTATCTGGAAATCTGGCTCATGCTGGTGGGTCAGTAGATCTGGCAATTTTTTC
TTTACACCTTGCTGGTGTGTCTTCTATTTTAGGGGCGGTGAATTTTATTACTACAATTAT
TAATATACGATGACAAGGGATGAAATTTGAACGCCTTTCGTTGTTTGTGTGGTCAGTGAA
GATTACAGCTATTTTACTTCTTTTATCTTTACCTGTGTTAGCGGGTGCAATTACTATGCT
CCTGACTGATCGAAATTTTAATACTGCTTTCTTTGATCCAGCAGGAGGTGGAGATCCTAT
TTTATATCAACATTTATTT
>ateralbus
AACCAAGAAATTTAGTCATTCTTTTATGGTAAAAAAACAGTTAGGCAGG-GAAAATTATT
AGATCTTTAGTATAGGAGTAAAATCTAAATACTAAAAGTTATATCAGACATGATTT-ATT
TATGCTGAGGCTGTGATAGCTTTAAGGGAAACTGGGATTGGATACCCCATTATTTTTAGC
TGTAAATTTAGCT--TGAGCTTACCAGAGTACTACGAATGTTTAAAACTCAAAGGGCTTG
GCGGTGTTTTAGACCTCTCAGGGGAACCTGTCTCGTAATCGACAATCCACGATAAACCTA
ACCTTTAATGGCAATTCAGCCTGTATACCGTCGTCGTCAGGTAACTTCTTAAAATATAGT
AGTTAGCTTGAGAATT--AT-ATTAATTAGAACGTCAGATCAAGGTGCAGCCAATGAAGA
GGTGAGGATGGGTTACAATTACATATTTGTAAATACGGCGTT-TGAAATGAAGTGTCTTA
AAGGAGGACTTGAAAGTAATTTTATCTATATAAATAGAATGAATAGGGCTCTGAAACGTG

CAGAATTTTAATGGTCGAACAGACCAACCCTTAAAGACTTCTGCATCTTTAGGACATTCT
GGTCCAACATCGAGGTCACAAACCTTTTTTTCGATATGGGCTCTTGAAAAAGATTATGCT
GTTATCCCTACGGTAACTAATTCCTTTAATCAATATTCT-TGGATCAAC-ACAAGTAAGA
TTTA-AAAGGAGGTTTTATTTGCTCCTTGGTTGCCCCAACCAAAAT--ATTTAATAGCTC
T-TCTTCTA-ATTAATTG--ATAAAATCTATTAACTTCTCTAAAGCTCGATAGGGTCTTC
TTGTCTTTTAATTATATCTGGACTTTTTCACCCAAAAATAAAATTCTAAATAATCTAAAA
GAGACAGGTATATTCTTGTCAAACCATTCATTCCAGCCTTCAATTATAAGGCAAGTGATT
ATGCTACCTTTGCACGGTCAGAGTACCGCGGCCGTT-TAAAA-CACTGGGCAGGTCCGAC
TTCGTATTTAAT-TTTAACACNNNNNNNNNNNNNNNNNNNNNNNNNNNTGATCCGGGTTAGT
TGGAACTGCTTTAAGATTATTAATTCGTGCGGAGTTAGGGCAGCCAGGTGCTTTGCTTGG
AGATGATCAACTATATAATGTAATTGTAACGGCCCATGCTTTTGTTATAATTTTTTTCTT
AGTAATACCTATGATGATTGGGGGTTTTGGAAATTGATTAGTGCCTTTGATATTAGGGGC
TCCAGATATAGTATTTCCTCGTTTAAATAATATGAGTTTCTGACTTCTTCCCCCTGCGCT
TTTACTTCTTTTGTCGTCAGCTGCGGTAGAAAGTGGTGTTGGCACAGGATGGACTGTATA
TCCCCCTTTATCTGGAAATTTGGCTCATGCTGGTGGGTCTGTAGATCTGGCGATTTTTTC
TTTACATCTTGCTGGGGTGTCTTCTATTTTAGGAGCGGTGAATTTCATTACTACAATTAT
TAATATGCGATGACAAGGAATAAAATTTGAGCGTCTTTCGTTGTTTGTCTGATCGGTAAA
AATTACAGCTATTTTACTTCTTTTATCTTTACCTGTGTTGGCGGGTGCAATCACTATACT
TCTAACTGATCGAAATTTTAATACTGCTTTCTTTGATCCGGCNNNNNNNNNNNNNNNNNN
NNNNNNNNNNNNNNNNNNN
>venulatus
AACCAAGAAATTTAGTCATTCTTTTATGGTAAAAAAACAGTTAGGCAGG-GAGAATTATT
AGATCTTTAGTATAGGAGTAAAATCTAAATACTAAAAGTTATATCAGACATGATTT-ATT
TATGCTGAGGCTGTGATAGCTTTAAGGGAAACTGGGATTGGATACCCCATTATTTTTAGC
TGTAAATTTAGCT--TGAGCTTACCAGAGTACTACGAATGTTTAAAACTCAAAGGGCTTG
GCGGTGTTTTAGACCTCTCAGGGGAACCTGTCTCGTAATCGACAATCCACGATAAACCTA
ACCTTTAATGGCAATTCAGCCTGTATACCGTCGTCGTCAGGTAACTTCTTAAAATATAGT
AGTTAGCTTGAGAATT--AT-ATTAATTAAAACGTCAGATCAAGGTGCAGCCAATGAAGA
GGTGCGGATGGGTTACAATTATATATTTGTAAATACGGCGTT-TGAAATGAGGTGTCTTA
AAGGAGGACTTGAAAGTAATTTTATCTATATAAATAGAATGAATAGGGCTCTGAAACGTG
CAGAATTTTAATGGTCGAACAGACCAACCCTTAAAGACTTCTGCATCTTTAGGACATTCT
GGTCCAACATCGAGGTCACAAACCTTTTTTTCGATATGGGCTCTTGAAAAAGATTATGCT
GTTATCCCTACGGTAACTAATTCCTTTAATCAATATTCT-TGGATCAAC-ACAAGTAAGA
TTTA-AAAGGAGGTTTTATTTGCTCCTTGGTTGCCCCAACCAAAAT--ATTTAATAGCTC
T-TCTTTTA-ATTAATCG--ATAAAATCTATTAACTTCTCTAAAGCTCGATAGGGTCTTC
TTGTCTTTTAATTATATCTGGACTTTTTCACCCAAAAATAAAATTCTAAATAATCTAAAA
GAGACAGATATATTCTTGTCAAACCATTCATTCCAGCCTTCAATTATAAGGCAAGTGATT
ATGCTACCTTTGCACGGTCAGAGTACCGCGGCCGTT-TAAAA-CACTGGGCAGGTCCGAC
TTCGTATTTAAT-TTTAACACNNNNNNNNNNNNNNNNNNTTGGAATATGATCCGGGTTAGT
TGGAACTGCTTTAAGGTTATTAATTCGTGCGGAGTTAGGGCAGCCAGGTGCTTTGCTTGG
AGATGATCAACTATATAATGTAATTGTAACGGCCCATGCTTTTGTTATAATTTTTTTCTT
AGTAATACCTATGATGATTGGGGGTTTTGGGAATTGATTAGTGCCTTTAATATTAGGGGC
TCCAGATATAGTATTTCCACGTTTAAACAATATAAGTTTCTGACTTCTTCCCCCTGCGCT
TTTACTTCTTTTGTCGTCAGCTGCGGTAGAAAGTGGTGTTGGTACAGGATGGACTGTATA
TCCCCCTTTATCTGGAAATTTGGCTCATGCTGGCGGGTCTGTAGATCTGGCGATTTTTTC
TTTACATCTTGCTGGGGTGTCTTCTATTTTAGGAGCGGTGAATTTCATTACTACAATTAT
TAATATGCGATGACAAGGAATAAAATTTGAGCGTCTTTCGTTGTTTGTCTGATCGGTAAA
AATTACAGCTATTTTACTTCTTTTATCTTTACCTGTGTTGGCGGGTGCAATTACTATACT

TCTAACTGATCGAAATTTTAATACTGCTTTCTTTGATCCGGCAGGAGGTGGCGATCCTAT
CTNNNNNNNNNNNNNNNNN
>trochulus
AACCAAGAAATTTAGTCATTCTTTTATGGTAAAAAAACAGTTAGGCAGG-GAAAATCATT
AGATCTTTAGTATAGGAGTAAAATCTAAATACTAAAAGTTATATCAGACATGATTT-ATT
TATGCTGAGGCTGTGATAGCTTTAAGGGAAACTGGGATTGGATACCCCATTATTTTTAGC
TGTAAATTTAGCT--TGAGCTTACCAGAGTACTACGAATGTTTAAAACTCAAAGGGCTTG
GCGGTGTTTTAGACCTCTCAGGGGAACCTGTCTCGTAATCGACAATCCACGATAAACCTA
ACCTTTAATGGCAGTTCAGCCTGTATACCGTCGTCGTCAGGTAACTTCTTAAAATATAGT
AGTTAGCTTGAGAATT--AT-ATTAATTAAAACGTCAGATCAAGGTGCAGCCAATGAAGA
GGTGAAGATGGGTTACAATTACATATTTGTAAATACGGCGTT-TGAAATGAAGTGTCTTA
AAGGAGGACTTGAAAGTAATTTTATCTATATAAATAGAATGAATAGGGCTCTGAAACGTG
CAGAATTTTAATGGTCGAACAGACCAACCCTTAAAGACTTCTGCATCTTTAGGACATTCT
GGTCCAACATCGAGGTCACAAACCTTTTTTTCGATATGGGCTCTTGAAAAAGATTATGCT
GTTATCCCTACGGTAACTAATTCCTTTAATCAATATTCT-TGGATCAAC-ACAAGTAAGA
TTTA-AAAGGAGGTTTTATTTGCTCCTTGGTTGCCCCAACCAAAAT--ATTTAATAGCTC
T-TCTTTTA-ATTAATTG--GTAAAATCTATTAACTTTTCTAAAGCTCGATAGGGTCTTC
TTGTCTTTTAATTATATCTGGACTTTTTCACCCAAAAATAAAATTCTAAATAATCTAAAA
GAGACAGATATATTCTTGTCAAACCATTCATTCCAGCCTTCAATTATAAGGCAAGTGATT
ATGCTACCTTTGCACGGTCAGAGTACCGCGGCCGTT-TAAAA-CACTGGGCAGGTCCGAC
TTCGTATNNNNN-NNNNNNNNNNNNNNNNNNNNNNNNNNNTTGGAATATGATCCGGGTTAGT
TGGAACTGCTTTAAGATTATTAATTCGTGCGGAGTTAGGGCAGCCAGGTGCTTTGCTTGG
AGATGATCAGCTATATAATGTAATTGTAACGGCCCATGCTTTTGTTATAATTTTTTTCTT
AGTAATACCTATGATGATTGGGGGTTTTGGAAATTGATTAGTGCCTTTAATATTAGGGGC
TCCAGATATAGTATTTCCACGTTTAAATAATATAAGTTTCTGACTTCTTCCCCCTGCGCT
TTTACTTCTTTTGTCGTCAGCTGCGGTAGAAAGTGGTGTTGGTACAGGATGGACTGTATA
TCCCCCTTTATCTGGAAATTTGGCTCATGCTGGCGGGTCTGTAGATCTGGCAATTTTTTC
TTTACATCTTGCTGGAGTGTCTTCTATTTTAGGAGCGGTGAATTTCATTACTACAATTAT
TAATATGCGATGACAAGGAATAAAATTTGAGCGTCTTTCGTTGTTTGTCTGATCGGTAAA
AATTACAGCTATTTTACTTCTTTTATCTTTACCTGTGTTGGCGGGTGCAATTACTATACT
TCTAACTGATCGAAATTTTAATACTGCTTTCTTTGATCCAGCGGGAGGTGGCGATCCTAN
NNNNNNNNNNNNNNNNNNN
>pergrandis
AACCAAGAAATTTAGTCATTCTTTTATGGTAAAAAGACAGTTAGGCAGA-TAAAATCATT
AGACCTTTAATAGAGGAGTAAAATCTATATATTAAAAGTTATATCTAATGTGGTTT-ATT
TATGTTGAATCTGTGATAGCTTTAAGGGAAACTGGGATTGGATACCCCATTATTTTTAGT
CGTAAATTTAGTT--TAAGCTTACCGGAGTACTACGAATGTTTAAAACTCAAAGAGCTTG
GCGGTGTTTTAGACCCCTCAGGGGAACCTGTCTCGTAATCGACAATCCACGTTAGACCTA
ACCCTTTATGGTCAAACAGCCTGTATACCGTCGTCGTCAGGTAACTTCTTAAAATATAGT
AGTTAGCTCGAGAATT--TT-ATTAATTAAAACGTCAGATCAAGGTGCAGCTAATGAAAG
GGCGAGGATGGGTTACAATTATATATTTGTAAATACGACATT-TGAAATGAAGTGTCTTA
AAGGAGGACTTGAAAGTAATTTCGATTATATAAACGGAATGAATAGGGCTCTGAAACGTG
CAGAATTTTAATGGTCGAACAGACCAACCCTTAAAGACTTCTGCATCTTTAGGACATTCT
GGTCCAACATCGAGGTCACAAACCTTTTTTTCGATATGGGCTCTTGAAAAAGATAATGCT
GTTATCCCTACGGTAACTATTTCCTTCGATCAAAATTTT-TGGATCAAC-ACAAGCAAGA
TTTA-AAAGGAGGCTCTATTTGCTCCTCGGTTGCCCCAACCAAAGT--ATTTAATAGTTT
T-TCTTTTA-TTTAATTG--ATAAAGTCCATTAATTTTTCTGAAGCTCGATAGGGTCTTC
TTGTCTTTTAATAATATCTGGACTTTTTCATCCAAAAATAAAATTCTAAACAATCTAAAA

GAGACAGGTGCATTCTTGTCAAACCATTCATTCCAGCCTTCAATTATAAGGCAAATGATT
ATGCTACCTTTGCACGGTCAGAGTACCGCGGCCGTT-TAAAA-CACTGGGCAGGTCCGAC
TTCGTATTTAAT-ATTGACACGACATTGTATATTTTGTTTGGAATATGATCTGGGTTGGT
TGGGACTGCTCTGAGGTTGTTAATTCGTGCAGAGTTAGGACAACCTGGTGCTTTGCTTGG
AGACGATCAGTTATATAATGTGATTGTGACAGCCCATGCTTTTGTTATAATTTTTTTCTT
GGTAATACCCATGATGATTGGAGGATTCGGAAATTGATTGGTGCCTCTAATGCTAGGGGC
TCCCGATATAGTATTTCCACGTTTAAATAATATAAGTTTCTGGCTTCTCCCTCCTGCACT
TTTACTTCTTTTGTCGTCAGCTGCAGTGGAAAGTGGGGTAGGTACGGGATGAACCGTATA
TCCGCCTTTAGCTGGTAATCTGGCTCACGCCGGTGGCTCTGTAGATCTGGCAATTTTTTC
TTTACATCTAGCTGGGGTTTCTTCTATTTTAGGGGCAGTAAATTTTATTACTACAATTAT
TAACATGCGATGGCAGGGAATAAAGTTCGAACGTCTTTCGTTATTTGTGTGGTCGGTAAA
GATTACAGCTATTTTACTTCTTTTATCCTTACCTGTGTTAGCCGGAGCAATTACTATACT
TCTAACTGACCGGAATTTTAATACTGCTTTCTTCGATCCGGCAGGAGGTGGTGATCCTAT
TTTATATCAGCATCTGTTT
>brettinghami
AACCAAGGAGTTTAGTCATTCTTTCATGGTAAAAAGACAGTTAGGCAGG-TAAAGTCATT
AGATCTTTAGTAAAGAAGTAAAATTTACATACTAAAAGTTATGCCAAACATGATTT-GTT
TATGCTGAATCTGTGATAGCTTTAAGGGAAACTGGGATTGGATACCCCATTATTTTTAGT
TGTAAATTTAGTT--TAGGCTTACCAGAGTACTACGAATGTTTAAAACTCAAAGGGCTTG
GCGGTGTTTTAGACCTCTCAGGGGAACCTGTCTCATAATCGACAATCCACGTTAGACCTA
ACCTTTAATAGTAATACAGCCTGTATACCGTTGTCGTCAGGTAACTTTTAAAAATACAGC
AGTTAGCTCGAGAATT--AT-ATTAATTAGGACGTCAGATCAAGGTGCAGCTAATGAAAA
GGTGAGGATGGGTTACAATTATATATTTATAACTACGGCACT-TGAAAT-AAGTGTCTTA
AAGGAGGACTTGAAAGTAATTTTGATTATATAAACAAAATGAATAGGGCTCTGAAACGTG
NNNNNNNNNNNNNNNNNNNNNNNNNNNNNNNNNNNNNNNNNTTCTGCATCTTTAGGATATTCT
GGTCCAACATCGAGGTCACAAACCTTTTTTTCGATATGGGCTCTTGAAAAAGATAATGCT
GTTATCCCTACGGTAACTAATTCCTTTGATCAAAATTCT-TGGATCAAA-ACAAGGAGGT
TTTA-AAAGGAGGCTTTACTTACTCCTTGGTTGCCCCAACCAAAGT--ATTTAATAGCCT
A-TCTTTA-CTTAATTG--A-GAAGCCTATTAATTCTTCTAAAGCTCGATAGGGTCTTC
TTGTCTTTTAATAATATTTGGACTTTTTCATCCAAAAATAAAATTCTAAGGAATCTAAAA
GAGACAGCTGTATTCTTGTCAAACCATTCATTCCAGCCTTCAATTATAAGGCAAATGATT
ATGCTACCTTTGCACGGTCAGAGTACCGCGGCCGTT-TAAAA-CACTGGGCAGGTCCGAC
TTCATATNNNNN-NNNNNNNNNTACATTGTATATTTTATTTGGTATATGATCAGGGTTGGT
TGGAACAGCTTTGAGACTCTTAATTCGTGCAGAACTAGGACAACCAGGTGCCTTGCTTGG
TGATGATCAGCTGTATAATGTTATTGTAACAGCACATGCTTTTGTTATAATTTTTTTTTT
AGTAATACCTATAATGATTGGGGGCTTTGGAAACTGGTTGGTACCACTAATGTTAGGGGC
TCCAGATATGGTATTTCCTCGTTTGAATAACATAAGTTTCTGACTTCTTCCTCCTGCACT
TTTACTTCTTTTGTCATCAGCTGCAGTGGAAAGTGGAGTAGGTACAGGATGGACTGTGTA
TCCACCATTAGCAGGAAATCTAGCTCATGCTGGCGGTTCTGTAGATCTAGCAATTTTTTC
TTTACATCTTGCTGGTGTATCATCTATTTTAGGGGCAGTGAATTTTATTACTACGATTAT
TAACATACGATGACAGGGAATAAAATTTGAACGTCTTTCATTGTTTGTATGGTCAGTAAA
GATTACAGCTATTTTATTACTCTTATCCTTACCGGTGTTAGCTGGAGCCATTACTATACT
TTTGACTGATCGGAATTTTAATACTGCCTTCTTTGATCCGGCAGGAGGTGGTGATCCTAT
CTTATATCAGCATTTATTC
>neocostatus
AACCAAGGAATTTAGTCATTCTTTTACGGTAAAAAGACAGTTAGGCAGA-TAGAGTCATT
AGATCCTTAGTAAAGGAGTAAAATCTATATACTAAAAGTTATACCTAATATGATTT-GTT
TATGCTGAATCTGTGATAACTTTAAGGGAAACTGGGATTGGATACCCCATTATTTTTAGT

TGTAAATTTAGTT--TAGGCTTACCAGAGTACTACGAATGTTTAAAACTCAAAGGGCTTG
GCGGTGTTTTAGACCTCTCAGGGGAACCTGTCTCATAATCGACAATCCACGTTAGACCTG
ACCTTTAATGGTAATACAGCCTGTATACCGTCGTCGTCAGGTAACTTTTAAAAATACAGG
AGTTAGCTCGAGAATT--GC-ATTAATTAAGACGTCAGATCAAGGTGCAGCTAATGAAAA
GGTGAGGATGGGTTACAATTATATATTTATAACCACGGCACT-TGAAATGAAGTGTCTTA
AAGGAGGACTTGAAAGTAATTTTGATTATATAAATAAAATGAATAGGGCTCTGAAACGTG
CAGAATTTTAATGGTCGAACAGACCAACCCTTAAAGACTTCTGCATCTTTAGGATATTCT
GGTCCAACATCGAGGTCACAAACCTTTTTTTCGATATGGGCTCTTGAAAAAGATAATGCT
GTTATCCCTACGGTAACTAATTCCTTTGATCAAAATTCT-TGGATCAAC-ACAAGCAAGT
TTTA-AAAGGAGGCTTTATTTACTCCTCGGTTGCCCCAACCAAAGT--ATTTAATAGTTT
A-TCTTTTA-CTTAATTG--ATAAAGCCCATTAATTCCTCTAAAGCTCGATAGGGTCTTC
TTGTCTTTTAATAATATTTGGACTTTTTCATCCAAAAATAAAATTCTAAACAATCTAAAA
GAGACAGCTGTATTCTTGTCAAACCATTCATTCCAGCCTTCAATTATAAGGCAAATGATT
ATGCTACCTTTGCACGGTCAGAGTACCGCGGCCGTT-TAAAA-CACTGGGCAGGTCCGAC
TTCATATTTAAT-ATTGACATTACATTATATATTTTATTTGGGATATGATCTGGACTGGT
TGGGACTGCTTTAAGGTTGTTAATTCGTGCAGAACTGGGACAACCAGGTGCTTTGCTTGG
GGATGATCAGCTGTATAATGTAATTGTAACAGCGCATGCTTTTGTTATAATTTTTTTCTT
AGTAATGCCTATAATGATTGGAGGTTTTGGAAACTGATTAGTGCCATTGATGTTAGGGGC
TCCAGACATGGTGTTTCCTCGTTTAAATAATATAAGTTTCTGGCTTCTTCCTCCTGCACT
TTTACTTCTTTTGTCGTCAGCTGCAGTGGAAAGTGGGGTAGGTACGGGATGGACTGTATA
TCCACCGTTGGCAGGAAATCTAGCTCATGCTGGCGGTTCTGTAGATCTAGCAATTTTTTC
TTTACATCTTGCTGGTGTGTCTTCTATTTTAGGGGCAGTAAATTTTATTACTACGATTAT
TAATATACGATGACAGGGAATAAAATTTGAACGTCTTTCATTGTTTGTGTGGTCAGTAAA
GATTACGGCTATTCTATTGCTTTTATCTTTACCGGTGTTAGCAGGTGCAATTACTATACT
TTTAACGGATCGGAATTTTAACACTGCCTTTTTTGATCCGGCAGGAGGTGGTGATCCTAT
TTTGTATCAGCATTTATTT
>rolani
AACCAAGGAATTTAGTCATTCTTTTATGGTAAAAAGACAGTTAGGCAGA-TATAGTCATT
AGATCCTTAGTAAAGGAGTAAAATCTACATACTAAAAGTTATACCGAATATGATTT-GTT
TATGCTGAATCTGTGATAGCTTTAAGGGAAACTGGGATTGGATACCCCATTATTTTTAGT
TGTAAATTTAGTT--TAGGCTTACCGGAGTACTACGAATGTTTAAAACTCAAAGGGCTTG
GCGGTGTTTTAGACCTCTCAGGGGAACCTGTCTCATAATCGACAATCCACGTTAGACCTA
ACCTTTAATAGTAATACAGCCTGTATACCGTCGTCGTCAGGTAACTTTTAAAAATACAGC
AGTTAGCTCGAGAATT--AT-ATTAATTAAGACGTCAGATCAAGGTGCAGCTAATGAAAA
GGTGAGGATGGGTTACAATTATATATTTATAACTACAGCACT-TGAAATTAAGTGTCTTA
AAGGAGGACTTGAAAGTAATTTTAATTATATAAGTAAAATGAATAGGGCTCTGAAACGTG
CAGAATTTTAATGGTCGAACAGACCAACCCTTAAAGACTTCTGCATCTTTAGGATATTCT
GGTCCAACATCGAGGTCACAAACCTTTTTTTCGATATGGGCTCTTGAAAAAGATAATGCT
GTTATCCCTACGGTAACTAATTCCTTTGATCAAAATTCT-TGGATCAAC-ACAAGCAGGT
TTTA-AAAGGAGGCTCTATTTACTCCTCGGTTGCCCCAACCAAAGT--ATTTAATAGTCT
A-TCTTTTA-CTTAATTG--ATAAAGCCTATTAATTCCTCTAAAGCTCGATAGGGTCTTC
TTGTCTTTTAATAATATCTGGACTTTTTCATCCAAAAATAAAATTCTAAGCAATCTAAAA
GAGACAGCTGTATTCTTGTCAAACCATTCATTCCAGCCTTCAATTATAAGGCAAATGATT
ATGCTACCTTTGCACGGTCAGAGTACCGCGGCCGTT-TAAAA-CACTGGGCAGGTCCGAC
TTCATATTTAAT-ATTGACATTACATTGTATATTTTATTTGGTATATGATCTGGGTTGGT
TGGAACTGCCCTAAGGTTGTTAATTCGTGCAGAACTAGGACAGCCAGGTGCTTTGCTTGG
GGATGATCAATTGTATAATGTAATTGTAACAGCTCATGCTTTTGTTATAATTTTTTTCTT
AGTAATACCTATAATGATTGGGGGTTTTGGAAACTGATTAGTGCCGTTGATGTTAGGGGC

TCCAGATATAGTATTTCCTCGTTTAAATAATATAAGTTTCTGGCTTCTTCCTCCTGCGCT
TTTACTTCTTTTGTCATCAGCCGCGGTAGAAAGTGGGGTAGGTACAGGATGGACTGTATA
CCCGCCGTTAGCAGGAAATCTAGCTCATGCTGGCGGTTCTGTAGATCTAGCAATTTTTTC
TTTACATCTTGCTGGTGTATCCTCTATTTTAGGGGCAGTAAATTTTATTACTACGATTAT
TAATATACGATGACAGGGAATAAAATTTGAACGTCTTTCATTGTTTGTGTGGTCAGTAAA
GATTACGGCTATTTTATTGCTTTTGTCCTTACCAGTGTTAGCGGGTGCAATTACTATACT
TCTGACTGATCGGAATTTTAACACTGCCTTCTTTGATCCGGCAGGAGGTGGTGATCCTAT
TTTGTATCAGCATTTATTT
>sulcatus
AACCAAGGAATTTAGTCATTCTTTTATGGTAAAAAGACAGTTAGGCAGA-TAGAGTCATT
AGATCCTTAGTAAAGGAGTAAAATCTATATACTAAAAGTTATGCCAAACATGATTT-GTT
TGTGCTGAATCTGTGATAACTTTAAGGGAAACTGGGATTGGATACCCCATTATTTTTAGT
TGTAAAATTAGTT--TA-GCTTACCGGAGTACTACGAATGTTTAAAACTCAAAGGGCTTG
GCGGTGTTTTAGACCTCTCAGGGGAACCTGTCTCATAATCGACAATCCACGTTAGACCTA
ACCTTTAATAGTAACACAGCCTGTATACCGTCGTCGTCAGGTAACTTTTAAAAACACAGT
AGTTAGCCCGAGAATT--AT-ATTAATTAAGACGTCAGATCAAGGTGCAGCTAATGAAAA
GGTGAGGATGGGTTACAATTATATATTTATAACTACGGCACT-TGAAATGAAGTGTCTTA
AAGGAGGACTTGAAAGTAATTTTGATTATATAAATAAAATGAATAGGGCTCTGAAACGTG
CAGAATTTTAATGGTCGAACAGACCAACCCTTAAAGACTTCTGCATCTTTAGGATATTCT
GGTCCAACATCGAGGTCACAAACCTTTTTTTCGATATGGGCTCTTGAAAAAGATAATGCT
GTTATCCCTACGGTAACTAATTCCTTTGATCAAAATTCT-TGGATCAAC-ACAAGCAAGT
TTTA-AGAGGAGGCTTTATTTACTCCTCGGTTGCCCCAACCAAAGT--ATTTAATAGTTT
A-TCTTTTA-CTTAATTG--A-AAAGTCTATTAATTCTTCTAAAGCTCGATAGGGTCTTC
TTGTCTTTTAATAGTATTTGGACTTTTTCATCCAAAAATAAAATTCTAAACAATCTAAAA
GAGACAGCTGTATTCTTGTCAAACCATTCATTCCAGCCTTCAATTATAAGGCAAATGATT
ATGCTACCTTTGCACGGTCAGAGTACCGCGGCCGTT-TAAAA-CACTGGGCAGGTCCGAC
TTCATATNNNNN-NNNNNNNNTACATTGTATATCTTATTTGGAATATGATCCGGATTGGT
TGGAACTGCTTTAAGGTTGTTAATTCGTGCAGAACTAGGACAGCCAGGTGCTTTGCTTGG
GGACGATCAGTTGTATAATGTAATTGTAACAGCACATGCTTTTGTTATAATTTTTTTCTT
AGTAATGCCTATAATGATTGGGGGTTTTGGAAATTGATTAGTGCCGTTAATATTAGGGGC
TCCAGATATAGTATTTCCTCGTTTAAATAATATAAGTTTTTGGCTTCTCCCTCCTGCGCT
TTTACTTCTTTTGTCATCAGCTGCAGTGGAAAGCGGGGTAGGCACGGGATGGACTGTATA
TCCGCCGTTAGCAGGAAATCTGGCTCATGCTGGCGGTTCTGTAGATCTAGCGATTTTTTC
TTTACATCTTGCTGGTGTATCTTCTATTTTAGGGGCGGTAAATTTTATTACTACGATTAT
TAATATACGATGACAGGGAATAAAATTTGAACGTCTTTCATTATTTGTATGATCAGTAAA
AATTACGGCTATTTTATTGCTTTTATCCTTACCAGTGTTAGCGGGGGCAATTACTATACT
TCTGACTGATCGGAATTTTAACACTGCCTTTTTTGATCCTGCAGGAGGTGGTGACCCTAT
TTTGTATCAGCATTTATTT
>bocki
AACCAAGGAATTTAGTCATTCTTTTATGGTAAAAAGACAGTTAGGCAGA-TGAAATCATT
GGATCCTTAGTAAAGGAGTAAAATCTATATACTAAAAGTTATACCGGATATGGTTT-GTT
TGTGTTGAATCTGTGATAGCTTTAAGGGAAACTGGGATTGGATACCCCATTATTTTTAGT
TGTAAATTTAGTT--TAGGCTTACCAGAGTACTACGAATGTTTAAAACTCAAAGGGCTTG
GCGGTGTTTTAGACCTCTCAGGGGAACCTGTCTCATAATCGACAATCCACGTTAGACCTA
ACCTTTTATGGTAATACAGCCTGTATACCGTCGTCGTCAGGTAACTTTTAAAAGTACAGA
AGTTAGCTCGAGAATT--AT-ATGAATTAAGACGTCAGATCAAGGTGCAGCTAATAAGAA
GGTGAGGATGGGTTACAATTATATATTTATAACTACGGCATT-TGAAATGAAGTGTCTTA
AAGGAGGACTTGAAAGTAATTTTGATTATATAAATAAAATGAATAAGGCTCTGAAACGTG

CAGAATTTTAATGGTCGAACAGACCAACCCTTAAAGACTTCTGCATCTTTAGGATATTCT
GGTCCAACATCGAGGTCACAAACCTTTTTTTCGATATGGGCTCTTAAAAAAGATAATGCT
GTTATCCCTACGGTAACTAATTCCTTTGATCAAAATCCT-TGGATCAAC-ACAAGCAGGT
CTTA-AAAGGAGGCTTTATTTACTCCTCAGTTGCCCCAACCAAAGT--ATTTAATGGTCT
A-TCTTTTA-CTTAATTG--ATAAAGTCCATTAATTCTTCTAAAGCTCGATAGGGTCTTC
TTGTCTTTTAATAGTATCTGGACCTTTTCATCCAAAAATAAAATTCTAAATAGTCTAAAA
GAGACAGCTGTATTCTTGTCAAACCATTCATTCCAGCCTTCAATTATAAGGCAAATGATT
ATGCTACCTTTGCACGGTCAGAGTACCGCGGCCGTT-TAAAA-CACTGGGCAGGTCCGAC
TTCATATTTAAT-GCTGACATTACATTGTATATTTTATTTGGTATGTGATCTGGATTGGT
TGGAACTGCTTTAAGGTTGTTAATTCGTGCAGAATTAGGACAACCAGGTGCTTTGCTTGG
GGATGATCAATTATATAATGTGATTGTAACAGCGCATGCTTTTGTTATAATTTTTTTTT
AGTAATGCCTATAATGATTGGGGGATTTGGAAACTGGTTAGTACCGTTGATGTTAGGGGC
TCCAGATATAGTATTTCCTCGTTTGAACAACATAAGTTTTTGGCTTCTTCCTCCTGCACT
TTTACTTCTTTTGTCATCAGCTGCAGTGGAAAGTGGGGTTGGTACAGGATGGACTGTGTA
TCCACCATTAGCAGGAAATCTGGCTCATGCTGGCGGTTCTGTAGACCTGGCAATTTTTTC
TCTACATCTTGCTGGTGTATCTTCTATTTTAGGAGCAGTAAATTTTATTACTACAATTAT
TAATATACGATGACAGGGAATAAAATTTGAACGTCTTTCATTGTTTGTATGGTCAGTAAA
GATTACAGCTATTTTATTGCTCTTGTCCTTACCTGTATTAGCGGGTGCAATTACCATACT
TCTAACTGATCGGAATTTTAATACTGCTTTCTTTGATCCAGCAGGAGGTGGTGATCCTAT
TTTGTATCAGCATTTATTT
>bruuni
AACCAAGAAATTTAGTCATTCTTTTATGGTAAAAAGACAGTTAGGCAAA-TAAAATCATC
AGATCTCTAGTAAAGGAGTAAAATCTAAATACTAAGAGTTATACCGAACATGGTTT-ATT
TATGTTGAGTCTGTGACAGCTTTAAGGGAAACTGGGATTGGATACCCCATTATTTTTAGT
TATAAATTTAGTT--TAAGCTTACCGGAGTACTACGAATGTTTAAAACTCAAAGAGCTTG
GCGGTGTTTTAGACCTCTCAGGGGAACCTGTCTCGTAATCGACAATCCACGTTAAACCTA
ACCTTTTATAGTGATACAGCCTGTATACCGTCGTCGTCAGGTAACTTCTTAAAATATAGT
AGTTAGCTTGAGAATT--TA-ATTAATTAAAACGTCAGATCAAGGTGCAGCTAATAAAGA
GGTGAGGATGGGTTACAATTACATATTTGTAAATACGGTACT-TGAAATGAAGTGTTTGG
AAGGAGGACTTGAAAGTAATTTTGATTATATAAACACAATGAATAAGGCTCTGAAACGTG
CAGAACTTTAATGGTCGAACAGACCAACCCTTAAAGACTTCTGCATCTTTAGGATATTCT
GGTCCAACATCGAGGTCACAAACCTTTTTTTCGATATGGGCTCTTGAAAAAGATAATGCT
GTTATCCCTACGGTAACTAATTCCTTTGATCAAAATTAT-TGGATCAAC-ACAAGTAGGA
CTTA-AAAGGAGGCTCTATATGCTCCTCGGTTGCCCCAACCAAAGT--ATTTAATAGTTT
TATTTTTA-CCTAATTA--ATTAAACCTATTAATTTTTCTGAAGCTCGATAGGGTCTTC
TTGTCTTTTACTAGTATTTGGACTTTTTCATCCAAAAATAAAATTCTAAATATTCTAAAA
GAGACAGCTGTATTCTTGTCAAACCATTCATTCCAGCCTTCAATTATAAGGCAAATGATT
ATGCTACCTTTGCACGGTCAGAGTACCGCGGCCGTT-TAAAA-CACTGGGCAGGTCCGAC
TTCGTATCTGGT-GCTCACACNNNNNNATATATCTTATTTGGAATATGATCAGGATTAGT
AGGGACTGCTTTAAGACTATTAATTCGTGCAGAATTAGGGCAACCCGGTGCTTTGCTTGG
AGATGATCAGTTATATAATGTAATTGTAACGGCTCATGCTTTTGTTATAATCTTTTTTTT
AGTGATACCTATGATGATTGGGGGCTTTGGGAATTGGTTAGTACCGTTGATGCTAGGGGC
TCCAGACATAGTATTTCCTCGCTTAAATAATATAAGTTTTTGGCTTCTGCCTCCTGCACT
TTTACTCCTTTTATCTTCAGCTGCGGTAGAAAGTGGGGTAGGGACAGGATGGACTGTATA
TCCACCATTATCAGGAAACCTAGCTCATGCTGGCGGTTCTGTAGATCTAGCAATTTTTTC
CTTACATCTTGCTGGTGTTTCTTCTATTTTAGGGGCTGTAAATTTTATTACTACAATTAT
TAATATACGATGACAGGGAATGAAATTTGAACGTCTTTCCTTGTTTGTATGGTCAGTAAA
AATTACGGCTATCTTGCTTCTTTTATCCCTACCTGTTTTAGCGGGGGCAATTACTATACT

TCTAACTGATCGAAATTTCAATACTGCATTTTTTGATCCGGCAGGAGGTGGTGATCCTAT
TTTATATCAGCATTTGTTT
>kinoshitai
AACCAAGAAATTTAGTCATTCTTTTATGGTAAAAAGACAGTTAGGCAAA-CGAAATCATT
AGATCTTTAGTAAAGGAGTAAAATCTAAATACTAAAAGTTATACCAAACATGATTT-ATT
TATGTTGAGTCTGTGACAGCTCTAAGGGAAACTGGGATTGGATACCCCATTATTTTTAGT
TATAAATTTAGTT--TAAGCTTACCGGAGTACTACGAATGTTTAAAACTCAAAGAGCTTG
GCGGTGTTTTAGACCTCTCAGGGGAACCTGTCTCGTAATCGACAATCCGCGTTAAACCTA
ACCTTTTATAGTAATACAGCCTGTATACCGTCGTCGTCAGGTAACTTCTAAAAATGTAGT
AGTTAGCTTGAGAACT--TA-ATTAGTTAAAACGTCAGATCAAGGTGCAGCTAATGAAGA
GGTGAGGATGGGTTACAATTACATATTTGTAAATACGGCACT-ATAAATGAAGTGTTTAG
AAGGAGGACTTGAAAGTAATTTTGATTACATAAACACAATGAATAAGGCTCTGAAACGTG
CAGAATTTTAATGGTCGAACAGACCAACCCTTAAAGACTTCTACATCTTTAGGATATTCT
GGTCCAACATCGAGGTCACAAACCTTTTTTTCGATATGGGCTCTTGAAAAAGATAATGCT
GTTATCCCTACGGTAACTAATTCCTTTGATCAAAATTGT-TGGATCAAT-ACAAGTAGGA
CTTA-AAAGGAGGCTCTATATACTCCTCGGTTGCCCCAACCAAAGT--ATTTAATAGTTT
TATCTTTTA-CTTAATCA--AT-AAACCTACCAATTTTTCTGAAGCTCGATAGGGTCTTC
TTGTCTTTTACTAATATTTGGACTTTTTCATCCAAAAATAAAATTCTAAACATTCTAAAA
GAGACAGCTGTATTCTTGTCAAACCATTCATTCCAGCCTTCAATTATAAGGCAAATGATT
ATGCTACCTTTGCACGGTCAGAGTACCGCGGCCGTT-TAAAA-CACTGGGCAGGTCCGAC
TTCGTATTTAGT-GTCTACACGACACTATATATCTTATTTGGGATATGATCGGGATTAGT
GGGGACTGCTTTAAGACTATTAATCCGTGCAGAATTAGGGCAACCCGGTGCTTTGCTTGG
AGATGATCAATTATATAATGTAATTGTGACGGCTCACGCTTTTGTTATAATCTTTTTTTT
AGTAATACCTATAATGATTGGTGGTTTTGGGAATTGGTTAGTACCGTTGATGTTAGGAGC
TCCGGATATAGTATTCCCTCGTCTAAATAATATAAGTTTTTGACTTCTTCCTCCTGCACT
TTTACTTCTTTTATCTTCGGCTGCGGTAGAAAGCGGGGTAGGCACAGGATGAACTGTATA
TCCGCCACTATCGGGAAATTTAGCTCATGCTGGTGGTTCTGTAGATCTAGCAATTTTTTC
CTTGCACCTTGCTGGGGTTTCTTCTATTTTAGGGGCGGTAAACTTTATTACTACAATTAT
TAATATACGATGACAGGGAATGAAATTTGAACGTCTTTCATTGTTCGTATGATCAGTAAA
AATTACAGCTATCTTGCTTCTTTTGTCTTTGCCTGTTTTGGCGGGCGCAATTACTATACT
TCTAACTGATCGAAATTTCAATACTGCATTTTTTGATCCGGCAGGAGGCGGTGATCCTAT
CTTATATCAGCATTTATTT
>achatinus
AACCAAGAAATTTAGTCATTCTCTTACGGTAAAAAGACAGTTAGGCAAA-TGAAATTATT
GGATCTTTAGTAAAGGAGTAAAATCTAAATACTAAAAGTTATACCAGATATAATTT-ATT
TATGTTGAATCTGTGACAGCTTTAAGGGAAACTGGGATTGGATACCCCATTATTTTTAGT
TATAAATTTAGTT--TAAGCTTACCAGAGTACTACGAATGTTTAAAACTCAAAGGGCTTG
GCGGTGTTTTAGACCTCTCAGGGGAACCTGTCTCGTAATCGACAATCCACGTTAAACCTA
ACCTTTTGTAGTAATACAGCTTGTATACCGTCGTCGTCAGGTAACTTCTTAAAATATAGT
AGTTAGCTTGAGAATT--TC-ATTAATTAAAACGTCAGATCAAGGTGCAGCTAATGAAGA
GGTGAGGATGGGTTACAATTACATATTTGTAAACACGGTGCT-TGAAATGGAGTATTTTA
AAGGAGGACTTGAAAGTAATTTTGATTATATAAGCAGAATGAATAAGGCTCTGAAACGTG
TAGAATTTTAATGGTCGAACAGACCAACCCTCAAAGACTTCTGCATCTTTAGGATATTCT
GGTCCAACATCGAGGTCACAAACCTTTTTTTCGATATGAGCTCTCAAAAAAGATAATGCT
GTTATCCCTACGGTAACTAATTTCTTTGATCAAAATTCC-TGGATCAAC-ACAAGTAAGA
CTCA-AAAGGAGGCTTTATTTACTCCTCGGTTGCCCCAACCAAAGT--ATTTAATAGCTA
TGTCTTTTA-CTTAATTA--ATAAAATCTACTAATTTCTCTAAAGCTCGATAGGGTCTTC
TTGTCTTTTAATAATATTTGGACTTTTTCATCCAAAAATAAAATTCTAAACAATCTAAAA

GAGACAGCTGCATTCTTGTCAAACCATTCATTCCAGCCTTCAATTATAAGGCAAATGATT
ATGCTACCTTTGCACGGTCAGAGTACCGCGGCCGTT-TAAAATCACTGGGCAGGTCCGAC
TTCGTATT-AGA-TCTCACACTACATTGTACATTTTATTTGGAATATGATCAGGACTAGT
AGGGACTGCTTTAAGGTTGTTGATTCGTGCGGAATTAGGGCAGCCTGGTGCTTTGCTTGG
GGACGATCAGTTATATAACGTAATTGTAACGGCGCATGCCTTTGTTATAATTTTCTTTTT
AGTAATGCCTATAATGATTGGGGGGTTTGGAAATTGATTAGTACCTTTGATATTAGGAGC
TCCAGACATAGTATTTCCTCGCTTAAATAATATGAGTTTCTGGCTTCTTCCTCCTGCGCT
TTTACTTCTTTTGTCCTCGGCTGCGGTGGAAAGAGGGGTAGGTACTGGATGGACTGTATA
TCCACCTCTTGCAGGAAATCTAGCTCATGCTGGTGGTTCTGTAGATCTTGCAATTTTTTC
TTTACATCTTGCTGGGGTATCTTCTATTTTAGGGGCTGTAAACTTTATTACTACAATTAT
TAACATACGGTGACAAGGGATGAAATTTGAGCGTCTTTCATTGTTCGTGTGATCGGTGAA
GATTACGGCTATTTTACTTCTTTTGTCTTTACCTGTTTTGGCAGGGGCTATTACTATACT
TTTGACTGATCGAAATTTCAATACTGCTTTCTTTGACCCAGCAGGAGGTGGTGATCCTAT
TTTGTATCAGCATCTTTTC
>monachus
AACCAAGAAATTTAGTCATTCTTTTACGGTAAAAAGACAGTTAGGCAAA-TGAAATTATC
GGATCTTTAGTAAAGGAGTAAAATCTAAATACTAAAAGTTATACCAAATATAATTT-ATT
TATGTTGAATCTGTGACAGCTTTAAGGGAAACTGGGATTGGATACCCCATTATTTTTAGT
TATAAATTTAGTT--TAAGCTTACCAGAGTACTACGAATGTTTAAAACTCAAAGGGCTTG
GCGGTGTTTTAGACCTCTCAGGGGAACCTGTCTCGTAATCGACAATCCACGTTAAACCTA
ACCTTTTGTAGTAATACAGCTTGTATACCGTCGTCGTCAGGTAACTTCTTAAAATATAGT
AGTTAGCTTGAGAATT--TC-ATTAATTAAAACGTCAGATCAAGGTGCAGCTAATGAAGA
GGTGAGGATGGGTTACAATTACATATTTGTAAATACGGTGCT-TGAAATGGAGTATTTTA
AAGGAGGACTTGAAAGTAATTTTGATTATATAAGCGGAATGAATAAGGCTCTGAAACGTG
CAGAATTTTAATGGTCGAACAGACCAACCCTCAAAGACTTCTGCATCTTTAGGATATTCT
GGTCCAACATCGAGGTCACAAACCTTTTTTTCGATATGAGCTCTCAAAAAAGATAATGCT
GTTATCCCTACGGTAACTAATTTCTTTGATCAAAATTCC-TGGATCAAC-ACAAGTAAGA
CTCA-AAAGGAGGCTTTATTTACTCCTCGGTTGCCCCAACCAAAGT--ATTTAATAGCTA
TGTCTTTTA-CTTAATTA--ATAAAATCTATTAATTTCTCTAAAGCTCGATAGGGTCTTC
TTGTCTTTTAATAATATTTGGACTTTTTCATCCAAAAATAAAATTCTAAACAATCTAAAA
GAGACAGCTGTATTCTTGTCAAACCATTCATTCCAGCCTTCAATTATAAGGCAAATGATT
ATGCTACCTTTGCACGGTCAGAGTACCGCGGCCGTT-TAAAACCACTGGGCAGGTCCGAC
TTCATATT-AGA-TCTCACACTACATTGTACATTTTATTTGGAATATGATCAGGACTAGT
AGGGACTGCTTTAAGGTTGTTGATTCGTGCGGAATTAGGGCAGCCTGGTGCTTTGCTTGG
GGACGATCAGTTATATAATGTAATTGTAACGGCGCATGCCTTTGTTATAATTTTCTTTTT
AGTAATACCTATAATGATTGGGGGGTTTGGAAATTGATTAGTACCTTTGATATTAGGAGC
TCCAGACATAGTATTTCCTCGCTTAAATAATATGAGTTTCTGGCTTCTTCCTCCTGCGCT
TTTACTTCTTTTGTCCTCGGCTGCGGTGGAAAGAGGGGTAGGTACTGGATGGACTGTATA
TCCACCTCTTGCAGGAAATCTAGCTCATGCTGGTGGTTCTGTAGATCTTGCAATTTTTTC
TCTACATCTTGCTGGAGTATCTTCTATTTTAGGGGCTGTAAACTTTATTACTACAATTAT
TAACATACGGTGACAAGGGATGAAATTTGAGCGTCTTTCGTTGTTCGTGTGATCGGTGAA
GATTACGGCTATTTTACTTCTTTTGTCTTTACCTGTTTTGGCAGGGGCTATTACTATACT
TTTGACTGATCGAAATTTCAATACTGCTTTCTTTGACCCAGCAGGAGGTGGTGATCCTAT
TTTGTATCAGCATCTTTTC
>simonis
AACCAAGAAATTTAGTCATTCTTTTACGGTAAAAAGACAGTTAGGCAAA-TGAAATTATC
GGATCTTTAGTAAAGGAGTAAAATCTAAATACTAAAAGTTATACCAGATATAATTT-ATA
TATGTTGAATCTGTGACAGCTTTAAGGGAAACTGGGATTGGATACCCCATTATTTTTAGT

TATAAATTTAGTT--TAAGCTTACCAGAGTACTACGAATGTTTAAAACTCAAAGGGCTTG
GCGGTGTTTTAGACCTCTCAGGGGAACCTGTCTCGTAATCGACAATCCACGTTAAACCTA
ACCTTTTGTAGTAATACAGCTTGTATACCGTCGTCGTCAGGTAACTTCTTAAAATATAGT
AGTTAGCTTGAGAATT--TA-ATTAATTAAAACGTCAGATCAAGGTGCAGCTAATGAAGA
GGTGAGGATGGGTTACAATTACATATTTGTAAACACGGTGCT-TGAAATGGAGTATTTTA
AAGGAGGACTTGAAAGTAATTTTGATTATATAAACAGAATGAATAAGGCTCTGAAACGTG
CAGAATTTTAATGGTCGAACAGACCAACCCTCAAAGACTTCTGCATCTTTAGGATATTCT
GGTCCAACATCGAGGTCACAAACCTTTTTTTCGATATGAGCTCTCAAAAAAGATAATGCT
GTTATCCCTACGGTAACTAATTTCTTTGATCAAAATTCC-TGGATCAAC-ACAAGTAAGA
CTCA-AAAGGAGGCTTTATTTACTCCTCGGTTGCCCCAACCAAAGT--ATTTAATAGCTA
TGTCTTTTA-CTTAATTA--ATAAAATCTACTAATTTCTCTAAAGCTCGATAGGGTCTTC
TTGTCTTTTAATAATATTTGGACTTTTTCATCCAAAAATAAAATTCTAAACAATCTAAAA
GAGACAGCTGCATTCTTGTCAAACCATTCATTCCAGCCTTCAATTATAAGGCAAATGATT
ATGCTACCTTTGCACGGTCAGAGTACCGCGGCCGTT-TAAAATCACTGGGCAGGTCCGAC
TTCGTATT-AGA-TCTCACACNNNNNNNNNNNNNNCTATTTGGAATATGATCGGGATTAGT
AGGAACTGCTTTAAGGTTGTTGATTCGTGCGGAATTAGGGCAGCCTGGTGCTTTGCTTGG
GGATGATCAATTATATAACGTAATTGTAACGGCGCATGCCTTTGTTATAATTTTTTTTTT
AGTAATGCCTATGATGATTGGTGGGTTTGGAAATTGATTAGTACCTTTGATATTAGGAGC
TCCAGACATAGTATTTCCTCGCTTAAATAATATAAGTTTTTGGCTTCTTCCCCCCGCGCT
TTTACTTCTTTTATCTTCGGCTGCGGTGGAAAGGGGGGTAGGTACTGGGTGGACTGTATA
TCCACCTCTTGCAGGAAATCTAGCTCATGCTGGTGGTTCTGTAGATCTTGCAATTTTTTC
TTTACATCTTGCTGGGGTATCTTCTATTTTAGGGGCTGTAAATTTTATTACCACAATTAT
TAATATACGGTGACAAGGAATGAAATTTGAGCGTCTTTCATTATTTGTGTGATCGGTGAA
GATTACGGCTATTTTACTTCTTTTATCTTTACCTGTTCTGGCAGGGGCTATTACTATGCT
TTTGACTGATCGAAATTTTAATACTGCTTTCTTTGACCCAGCAGGAGGTGGTGATCCTGT
TTTGTACCAGCATCTTTTT
>magus
AACCAAGAAATTTAGTCATTCTTTTACGGTAAAAAGACAGTTAGGCAAA-TGAAATTATT
GGATCTTTAGTAAAGGAGTAAAATCTGAATACTAAAAGTTATACCAGATATAGTTT-ATT
TATGTTGAATCTGTGACAGCTTTAAGGGAAACTGGGATTGGATACCCCATTATTTTTAGT
TATAAATTTAGTT--TAAGCTTACCAGAGTACTACGAATGTTTAAAACTCAAAGGGCTTG
GCGGTGTTTTAGACCTCTCAGGGGAACCTGTCTCATAATCGACAATCCACGTTAAACCTA
ACCTTTTGTAGTAATACAGCTTGTATACCGTCGTCGTCAGGTAACTTCTTAAAATATAGT
AGTTAGCTTGATAATT--TG-ATTAATTAAAACGTCAGATCAAGGTGCAGCTAATGAAAA
GGTGAGGATGGGTTACAATTACATATTTGTAAATACGGTGCT-TGAAATGAAGTATCTTA
AAGGAGGACTTGAAAGTAATTTTGATTATATAAGCAGAATGAATAAGGCTCTGAAACGTG
CAGAATTTTAATGGTCGAACAGACCAACCCTTAAAGACTTCTGCATCTTTAGGATATTCT
GGTCCAACATCGAGGTCACAAACCTTTTTTTCGATATGAGCTCTCAAAAAAGATAATGCT
GTTATCCCTACGGTAACTAATTTCTTTGATCAAAATTTC-TGGATCAAC-ACAAGTAAGA
CTCA-AAAGGAGGCTTTATTTACTCCTCGGTTGCCCCAACCAAAGT--GTTTAATAGTTA
TGTCTTTTA-CTTAATGA--ATAAAATCTATTAATTTCTCTAAAGCTCGATAGGGTCTTC
TTGTCTTTTAATAACATTTGGACTTTTTCATCCAAAAATAAAATTCTAAACAATCTAAAA
GAGACAGCTGTATTCTTGTCAAACCATTCATTCCAGCCTTCAATTATAAGGCAAATGATT
ATGCTACCTTTGCACGGTCAGAGTACCGCGGCCGTT-TAAAATCACTGGGCAGGTCCGAC
TTCGCATT-AAA-CTTTACACGACATTGTACATTCTATTTGGAATATGATCGGGACTAGT
AGGGACTGCTTTAAGGTTGTTGATTCGTGCGGAATTAGGGCAACCTGGTGCTTTGCTTGG
GGATGATCAGTTATATAATGTAATTGTAACGGCGCATGCCTTTGTTATAATTTTTTTTTT
AGTAATGCCTATGATAATTGGGGGGTTTGGAAATTGATTAGTACCTTTGATATTAGGAGC

TCCAGACATAGTATTTCCTCGCTTAAATAATATGAGTTTTTGGCTTCTTCCTCCTGCACT
TTTACTTCTTTTGTCCTCGGCTGCAGTGGAAAGGGGGGTAGGTACTGGATGGACTGTATA
TCCACCTCTTGCAGGAAATCTAGCTCATGCTGGTGGTTCTGTAGATCTTGCAATTTTTTC
TTTACATCTTGCTGGGGTATCTTCTATTTTAGGAGCTGTAAACTTTATTACCACAATTAT
TAATATACGGTGACAAGGAATGAAATTTGAGCGTCTTTCGTTATTTGTGTGATCGGTGAA
GATTACGGCTATTTTACTTCTTTTATCTTTACCTGTTTTGGCAGGGGCTATTACTATACT
TTTGACTGATCGAAATTTTAATACTGCTTTCTTTGATCCAGCAGGAGGTGGTGATCCTAT
TTTGTACCAACATCTTTTC
>consors
AACCAAGAAATTTAGTCATTCTTTTACGGTAAAAAGACAGTTAGACAAA-TTAAATTATT
GGATCTTTAGTAAAGGAGTAAAATCTAAATACTAGAAGTTATACCAGATATAGTTT-ATT
TATGTTGAATCTGTGACAGCTTTAAGGGAAACTGGGATTGGATACCCCATTATTTTTAGT
TATAAATTTAGTT--TAAGCTTACCAGAGTACTACGAATGTTTAAAACTCAAAGGGCTTG
GCGGTGTTTTAGACCTCTCAGGGGAACCTGTCTCATAATCGACAATCCACGTTAAACCTA
ACCTTTTGTAGTAATACAGCTTGTATACCGTCGTCGTCAGGTAACTTCTTAAAATATAGT
AGTTAGCTTGAGAATT--TT-ATTAATTAAAACGTCAGATCAAGGTGCAGCTAATGAAAA
GGTGAGGATGGGTTACAATTACATATTTGTAAACACGGTGCT-TGAAACGGAGTATTTTA
AAGGAGGACTTGAAAGTAATTTTGATTATATAAGTGGAATGAATAAGGCTCTGAAACGTG
CAGAATTTTAATGGTCGAACAGACCAACCCTTAAAGACTTCTGCATCTTTAGGATATTCT
GGTCCAACATCGAGGTCACAAACCTTTTTTTCGATATGAGCTCTCAAAAAAGATAATGCT
GTTATCCCTACGGTAACTAATTTCTTTGATCAAAATTTC-TGGATCAAC-ACAAGTAAGA
CTCA-AAAGGAGGCTTTATTTACTCCTCGGTTGCCCCAACCAAAGT--ATTTAATAGCTA
TGTCTTTTA-CTTAATTA--ATAAAATCTATTAATTTCTCTAAAGCTCGATAGGGTCTTC
TTGTCTTTTAATAATATTTGGACTTTTTCATCCAAAAATAAAATTCTAAACAATCTAAAA
GAGACAGCTGTATTCTTGTCAAACCATTCATTCCAGCCTTCAATTATAAGGCAAATGATT
ATGCTACCTTTGCACGGTCAGAGTACCGCGGCCGTT-TAAAATCACTGGGCAGGTCCGAC
TTCGTATT-AGC-TTTTACACTACATTGTACATTCTATTTGGAATATGATCAGGACTAGT
AGGGACTGCTTTAAGGTTGTTGATTCGTGCGGAATTAGGGCAGCCTGGTGCTTTGCTTGG
AGACGATCAGTTATATAATGTAATTGTAACGGCGCATGCCTTTGTTATAATTTTCTTTTT
AGTAATGCCTATGATGATTGGGGGGTTTGGAAATTGATTAGTACCTTTGATATTAGGAGC
TCCAGACATAGTATTTCCCCGTTTAAATAATATGAGTTTTTGGCTTCTTCCTCCTGCACT
TTTACTTCTTTTGTCCTCGGCTGCGGTTGAAAGGGGGGTAGGTACTGGATGGACTGTATA
TCCACCTCTTGCAGGAAATCTAGCTCATGCTGGTGGTTCTGTAGATCTTGCAATTTTTTC
TTTACACCTTGCTGGAGTATCTTCTATTTTAGGGGCTGTAAATTTTATTACCACAATTAT
TAATATACGGTGACAAGGGATGAAATTTGAGCGTCTTTCATTATTTGTGTGATCGGTAAA
GATCACGGCTATTTTACTTCTTTTATCTTTACCTGTTTTGGCAGGGGCTATTACTATACT
TTTAACTGATCGAAATTTCAATACTGCTTTCTTTGATCCAGCAGGAGGTGGTGATCCTAT
CTTGTATCAGCATCTTTTT
>catus
AACCAAGAAATTTAGTCATTCTTTAATGGTAAAAAGACAGTTAGGCAAA-TGAAACTATT
GGATCTTTAGTAGAGGAGTAAAATCTAAATACTAAAAGCTACATCAAACATAGTTT-ATT
TATGTTGAATCTGTGACAGCTTTAAGGGAAACTGGGATTGGATACCCCATTATTTTTAGT
TATAAATTTAGTT--TAAGCTTACCAGAGTACTACGAATGTTTAAAACTCAAAGGGCTTG
GCGGTGTTTTAGACCTCTCAGGGGAACCTGTCTCATAATCGACAATCCACGTTAAACCTA
ACCTTTTATAGTAATACAGCTTGTATACCGTCGTCGTCAGGTAACTTCTTAAAATATAGT
AGTTAGCTTGAGAATT--TG-ATTAATTAAAACGTCAGATCAAGGTGCAGCTAATGAAAA
GGTGAGGATGGGTTACAATTACATATTTGTAAACACGGCACT-TGAAATGAAGCGTCTTA
AAGGAGGACTTGAAAGTAATTTTGGTTATATAGGCAGAATGAATAAGGCTCTGAAACGTG

CAGAATTTTAATGGTCGAACAGACCAACCCTTAAAGACTTCTGCATCTTTAGGATATTCT
GGTCCAACATCGAGGTCACAAACCTTTTTTTCGATATGAGCTCTCAAAAAAGATAATGCT
GTTATCCCTACGGTAACTAATTCCTTTGATCAAAAATTC-TGGATCAAC-ACAAGTAAGA
CTTA-AAAGGAGGCTTTATCTACTCCTCGGTTGCCCCAACCAAAGT--ATTTAATAGTTA
TGTCTTTTA-CTTAATTA--ATAAAATCTATTAATTTCTCTAAAGCTCGATAGGGTCTTC
TTGTCTTTTAATAACATTTGGACTTTTTCATCCAAAGATAAAATTCTAAACAATCTAAAA
GAGACAGCTGTATTCTTGTCAAACCATTCATTCCAGCCTTCAATTATAAGGCAAATGATT
ATGCTACCTTTGCACGGTCAGAGTACCGCGGCCGTT-TAAAACCACTGGGCAGGTCCGAC
TTCGTATT-AGC-TTTTACACTACATTGTATATTCTATTTGGAATATGATCGGGACTGGT
AGGAACTGCTTTAAGGTTGTTGATTCGTGCAGAATTAGGGCAACCTGGTGCTTTGCTTGG
GGACGATCAGTTATATAATGTAATTGTAACGGCGCATGCCTTTGTTATAATTTTCTTTTT
AGTAATGCCTATGATGATTGGGGGGTTTGGAAACTGATTGGTGCCTTTAATATTAGGAGC
TCCAGATATAGTATTTCCTCGTTTAAATAATATGAGTTTTTGGCTTCTTCCTCCTGCACT
TTTGCTTCTTTTATCCTCGGCTGCGGTAGAAAGGGGAGTAGGTACCGGATGGACTGTATA
TCCACCTCTTTCAGGAAATCTAGCTCATGCTGGTGGTTCCGTAGATCTTGCGATTTTTTC
TTTACACCTTGCTGGGGTATCTTCTATTTTAGGGGCGGTAAACTTTATTACTACAATCAT
TAACATACGGTGACAAGGAGTGAAATTTGAACGTCTTTCGTTGTTTGTGTGATCGGTAAA
GATTACGGCTATCTTGCTTCTTTTATCTTTACCTGTTTTAGCTGGGGCAATTACTATACT
TTTAACTGATCGAAATTTCAATACTGCTTTTTTTGACCCCGCAGGAGGTGGTGATCCTAT
TTTGTATCAACATCTTTTC
>leobottoni
AACCAAGAAATTTAGTCATTCTTTTACGGTAAAAAGACAGTTAGGCAAA-TAAAATTATT
AGATCTTTAGTAGAGGAGTAAAATCTAAATACTAAAAGTTATATCAGGTATAGTTT-ATT
TATGTTGAATCTGTGACACCTTTAAGGGAAACTGGGATTGGATACCCCATTATTTTTAGT
TATAAATTTAGTT--TAAGCTTACCAGAGTACTACGAATGTTTAAAACTCAAAGGGCTTG
GCGGTGTTTTAGACCTCTCAGGGGAACCTGTCTCATAATCGACAATCCACGTTAAACCTA
ACCTTTTATAGTAATACAGTTTGTATACCGTCGTCGTCAGGTAACTTCTTAAAATCTAGT
AGTTAGCTTGAGAATT--TG-GTTAATTAAAACGTCAGATCAAGGTGCAGCTAATGAAAA
GGTGAGGATGGGTTACAATTATATATTTGTAAATATGGTATT-TGAAATGAAGTATTTTA
AAGGAGGACTTGAAAGTAATTTTGATTATATAAGTAAAATGAATAAGGCTCTGAAACGTG
CAGAATTTTAATGGTCGAACAGACCAACCCTTAAAGACTTCTGCATCTTTAGGATATTCT
GGTCCAACATCGAGGTCACAAACCTTTTTTTCGATATGAACTCTCAAAAAAGATAATGCT
GTTATCCCTACGGTAACTAATTCCTTTCATCAAAAATTT-TGGATCAAC-ACAAGTAAGA
CTAC-AAAGGAGGCTTTATTTACTCCTCGGTTGCCCCAACCAAAGT--ATTTAATAGCTA
TATCTTTTA-CTAAATCA--ATAAAACCTATTAATTTCTCTAAAGCTCGATAGGGTCTTC
TTGTCTTTTAATAATATTTGGACTTTTTCATCCAAAAATAAAATTCTAAACAATCTAAGA
GAGACAGCCGTATTCTTGTCAAACCATTCATTCCAGCCTTCAATTATAAGGCAAATGATT
ATGCTACCTTTGCACGGTCAGAGTACCGCGGCCGTT-TAAAATCACTGGGCAGGTCCGAC
TTCGTATT-AGC-TTTTACACTACATTGTATATTCTATTCGGAATATGATCAGAGCTGGT
AGGAACTGCTTTAAGGTTGTTGATTCGTGCAGAGTTAGGGCAGCCTGGTGCATTGCTTGG
GGATGATCAGTTATATAATGTAATTGTAACAGCGCATGCTTTTGTTATAATTTTTTTTTT
AGTAATACCTATGATGATTGGAGGGTTTGGAAATTGATTAGTACCTTTAATGTTAGGGGC
TCCAGACATAGTATTTCCTCGCTTGAATAATATGAGTTTTTGACTTCTTCCTCCTGCACT
TTTGCTTCTTTTATCCTCGGCTGCAGTGGAAAGGGGGGTAGGTACAGGATGGACTGTATA
TCCCCCTCTTGCAGGAAATCTAGCTCATGCTGGTGGTTCTGTAGATCTTGCGATTTTTTC
TTTACATCTTGCTGGGGTGTCTTCTATTTTAGGGGCGGTAAATTTTATTACTACAATTAT
TAATATACGATGACAAGGGATGAAATTTGAGCGTCTTTCTTTATTTGTGTGGTCGGTGAA
GATCACGGCTATCTTGCTTCTTTTATCTTTACCAGTTTTAGCGGGGGCTATTACTATACT

TTTAACTGATCGAAATTTTAATACTGCTTTTTTGACCCAGCAGGAGGTGGTGATCCTAT
TTTGTATCAACATCTTTTT
>striolatus
AACCAAGAAATTTAGTCATTCTTTTATGGTAAAAAGACAGTTAGGCAAA-TGAAATTACT
GGATCTCTAGTAGAGGAGTAAAATCTAAATACTAAGAGTTATACCAGACGTAGTTT-ATT
TATGTTGAATCTGTGACAGCTTTAAGGGAAACTGGGATTGGATACCCCACTATTTCTAGT
TATAAATTTAGTT--TAAGCTTACCAGAGTACTACGAATGTTTAAAACTCAAAGGGCTTG
GCGGTGTTTTAGACCTCTCAGGGGAACCTGTCTCATAATCGACAATCCACGTTAGACCTA
ACCTTTTGTAGTAATACAGCTTGTATACCGTCGTCGTCAGGTAACTTCTTAAAATATAGT
AGTTAGCTCGAGAATT--TG-ATTAATTAAAACGTCAGATCAAGGTGCAGCTAATAAAAA
GGTGAGGATGGGTTACAATTATATATTTATAAATACGGTACT-TGAAATGAAGTATCTTA
AAGGAGGACTTGAAAGTAATTCTGATTATATAAACAGAATGAATAAGGCTCTGAAACGTG
CAGAATTTTAATGGTCGAACAGACCAACCCTTAAAGACTTCTGCATCTTTAGGACATTCT
GGTCCAACATCGAGGTCACAAACCTTTTTTTCGATATGAGCTCTCAAAAAAGATAATGCT
GTTATCCCTACGGTAACTAATTTCTTTAATCAAAATTCT-TGGATCAAT-ACAAGTAAGG
CCTA-AAAGGAGGCTTTATTTACTCCTCGGTTGCCCCAACCAAAGT--GTTTAATAGCTA
TGTCTTTTA-CTTGATTA--ATAAAATCTATTAATTTCTCTAAAGCTCGATAGGGTCTTC
TTGTCTTTTAATAATATCTGGACTTTTTCATCCAAAAATAAAATTCTAAACAATCTAAAA
GAGACAGCCGTATTCTTGTCAAACCATTCATTCCAGCCTTCAATTATAAGGCAAATGATT
ATGCTACCTTTGCACGGTCAGAGTACCGCGGCCGTT-TAAAACCACTGGGCAGGTCCGAC
TTCGTATT-GAT-TTTTACACTACATTATATATCTTGTTTGGAATATGATCAGGACTGGT
GGGGACTGCTTTAAGGTTATTAATTCGTGCAGAGTTAGGACAGCCTGGTGCCTTGCTTGG
GGATGATCAATTATATAATGTAATTGTGACGGCTCATGCTTTTGTTATAATTTTTTTTT
AGTAATACCTATAATGATTGGGGGGTTTGGAAATTGATTGGTACCTTTAATATTAGGGGC
TCCAGATATAGTGTTTCCTCGCTTAAATAATATGAGTTTTTGGCTTCTTCCTCCTGCGCT
TTTACTCCTTTTATCTTCGGCTGCGGTGGAAAGGGGGGTAGGTACTGGATGGACTGTATA
TCCTCCTCTAGCAGGGAATCTAGCTCATGCTGGTGGTTCTGTAGATCTTGCGATTTTTTC
ATTACATCTTGCTGGGGTATCTTCTATTTTAGGGGCGGTAAATTTTATTACCACAATTAT
TAATATACGATGACAAGGAATGAAGTTTGAGCGCCTTTCGTTATTCGTCTGATCAGTGAA
GATCACGGCTATTTTACTTCTTTTATCTTTACCTGTTTTGGCGGGAGCTATTACTATACT
TCTAACTGATCGAAATTTCAATACTGCTTTCTTTGATCCAGCAGGAGGCGGTGATCCTAT
TTTGTATCAGCATCTTTTT
>aurisiacus
NNNNNNNNNNNNNNNNNNNNTTCTTTTATGGTAAAAAGACAGTTAGGCAGA-TGAGATTATT
AGATCTTTAGTAAAGGAGTAAAATCTAAATACTAAAAGATACACCGGATATAATTT-ATT
TATGTTGAATCTGTGACAGCTTTAAGGGAAACTGGGATTGGATACCCCATTATTTTTAGT
TATAAATTTAGTT--TAAGCTTACCGGAGTACTACRAATGTTTAAAACTCAAAGGGCTTG
GCGGTGTTTTAGACCTCTCAGGGGAACCTGTCTCGTAATCGACAATCCACGTTAAACCTA
ACCTTTTATGGTAAAACAGCTTGTATACCGTCGTCGTCAGGTAACTTCTTAAAATATAGT
AGTTAGCTTGAGAATT--TA-ATTAATTAAAACGTCAGATCAAGGTGCAGCTAATGAAAA
GGTGAGGATGGGTTACAATTATATATTTATAAATACGGTACT-TGAAATGGAGTATCTTA
AAGGAGGACTTGAAAGTAATTTTAATTATATAAATAGAATGAATAAGGCTCTGAAACGTG
CNNNNNNNNNNNNGGTCGAACAGACCAACCCTT-AAGACTTCTGCATCTTTAGGATATTCT
GGTCCAACATCGAGGTCACAAACCTTTTTTTCGATACGGACTCTTAAAAAAGATAATGCT
GTTATCCCTACGGTAACTAATTTCTTTGATCAAAATTCT-TGGATCAGC-GCAAGTAAGA
CTTA-AAAGGAGGCTTTATTTACTCCTCGGTTGCCCCAACCAAAGT--ATTTAATAGTTA
TGCTTTTTA-CTTAATTA--GTAAAACCTATTAATTTTTCTAAAGCTCGATAGGGTCTTC
TTGTCTTTTAATAATATCTGGACTTTTTCATCCAAAAATAAAATTCTAAACAATCAAAAA

GAGACAGCTGTATTCTTGTCAAACCATTCATTCCAGCCTTCAATTATAAGGCAAATGATT
ATGCTACCTTTGCACGGTCAGAGTACCGCGGCCGTT-TAAAACCACTGGGCAGGTCCGAC
TTCGCATT-AAT-TTTTACACTACATTATATATTCTATTTGGGATATGATCAGGCTTGGT
AGGGACTGCTTTAAGGCTATTAATTCGTGCAGAGTTAGGACAGCCAGGTGCTTTGCTTGG
AGACGATCAGCTATATAATGTAATTGTGACGGCGCATGCTTTTGTTATAATCTTCTTTTT
AGTAATGCCTATGATGATTGGGGGTTTTGGGAATTGATTGGTACCCTTGATATTAGGGGC
TCCAGATATAGTATTTCCTCGCTTAAATAACATGAGTTTTTGGCTTCTTCCCCCTGCACT
TTTACTTCTTTTGTCTTCGGCTGCGGTAGAAAGAGGGGTAGGAACGGGATGAACTGTATA
CCCACCTCTGGCAGGGAATCTTGCTCATGCTGGTGGTTCTGTAGATCTTGCAATTTTTTC
TTTACACCTTGCTGGGGTATCTTCTATTTTAGGGGCTGTAAACTTCATTACTACAATTAT
TAACATACGATGACAGGGAATGAAGTTTGAGCGTCTCTCGCTGTTTGTTTGATCGGTGAA
GATCACGGCGATCTTACTTCTTCTATCCCTACCTGTTTTGGCGGGGGCTATTACTATGCT
TTTAACTGATCGAAATTTCAATACTGCTTTCTTTGATCCAGCAGGGGGTGGTGATCCTAT
TTTGTATCAGCACCTTTTT
>gauguini
AACCAAGAAATTTAGTCATTCTTTTATGGTAAAAAGACAGTTAGGCAGA-TGAGATTATT
AGATCTTTAGTAAAGGAGTAAAATCTAAATACTAAAAGATATACCGGACATAATTT-ATT
TATGTTGAATCTGTGACAGCTTTAAGGGAAACTGGGATTGGATACCCCATTATTTTTAGT
TATAAATTTAGTT--TAAGCTTACCGGAGTACTACGAATGTTTAAAACTCAAAGGGCTTG
GCGGTGTTTTAGACCTCTCAGGGGAACCTGTCTCGTAATCGACAATCCACGTTAAACCTA
ACCTTTTATGGTAAAACAGCTTGTATACCGTCGTCGTCAGGTAACTTCTTAAAATATAGT
AGTTAGCTTGAGAATT--TA-ATTAATTAAAACGTCAGATCAAGGTGCAGCTAATGAAAA
GGTGAGGATGGGTTACAATTATATATTTATAAATACGGTACT-TGAAATGGAGTATCTTA
AAGGAGGACTTGAAAGTAATTTTAATTATATAAATAGAATGAATAAGGCTCTGAAACGTG
CAGAATTTTAATGGTCGAACAGACCAACCCTTAAAGACTTCTGCATCTTTAGGATATTCT
GGTCCAACATCGAGGTCACAAACCTTTTTTTCGATACGGGCTCTTAAAAAAGATAATGCT
GTTATCCCTACGGTAACTAATTTCTTTGATCAAAATTCC-TGGATCAAC-GCAAGTAAGA
CTTA-AAAGGAGGCTTTACTTACTCCTCGGTTGCCCCAACCAAAGT--ATTTAATAGTTA
TGCTTTTTA-CTTAATTA--GTAAAACCTATCAATTTTTCTAAAGCTCGATAGGGTCTTC
TTGTCTTTTAATAATATCTGGACTTTTTCATCCAAAAATAAAATTCTAAACAATCAAAAA
GAGACAGCTGTATTCTTGTCAAACCATTCATTCCAGCCTTCAATTATAAGGCAAATGATT
ATGCTACCTTTGCACGGTCAGAGTACCGCGGCCGTT-TAAAACCACTGGGCAGGTCCGAC
TTCGCATT-AAT-TTCTACACTACATTATATATTCTGTTTGGGATATGATCAGGCTTGGT
AGGGACCGCTTTAAGGCTATTAATCCGTGCAGAGTTAGGACAGCCAGGTGCTTTGCTTGG
AGATGATCAGCTATATAATGTAATCGTGACGGCGCATGCTTTTGTTATAATCTTCTTCTT
AGTAATGCCTATGATGATTGGGGGTTTTGGGAATTGATTGGTACCTTTGATATTAGGGGC
TCCAGATATAGTATTTCCTCGCTTAAACAACATGAGTTTTTGGCTTCTTCCCCCTGCACT
TTTACTTCTTTTGTCTTCGGCTGCGGTAGAAAGAGGGGTAGGAACGGGATGAACTGTATA
CCCACCTCTGGCAGGGAATCTTGCTCATGCTGGTGGTTCTGTAGATCTTGCAATTTTTTC
TTTACACCTTGCTGGGGTATCTTCCATTTTAGGGGCTGTAAACTTTATTACTACAATTAT
TAACATACGATGACAGGGAATGAAGTTTGAGCGTCTCTCGCTGTTTGTTTGATCGGTGAA
GATCACGGCGATCTTACTTCTTCTATCCCTACCTGTTTTGGCGGGGGCTATTACTATGCT
TTTAACTGATCGAAATTTCAATACTGCTTTCTTTGATCCAGCAGGGGGTGGTGATCCTAT
TTTGTATCAGCACCTTTTT
>circumcisus
AACCAAGAAATTTAGTCATTCTTTTATGGTAAAAAGACAGTTAGGCAGA-TGAGATTATT
AGATCTTTAGTAAAGGAGTAAAATCTAAATACTAAAAGATACACCAGATATAATTT-ATT
TATGTTGAATCTGTGACAGCTTTAAGGGAAACTGGGATTGGATACCCCATTATTTTTAGT

TATAAATTTAGTT--TAAGCTTACCGGAGTACTACGAATGTTTAAAACTCAAAGGGCTTG
GCGGTGTTTTAGACCTCTCAGGGGAACCTGTCTCGTAATCGACAATCCGCGTTAAACCTA
ACCTTTTATGGTAAAACAGCTTGTATACCGTCGTCGTCAGGTAACTTCTTAAAATATAGT
AGTTAGCTTGAGAATT--TA-ATTAATTAAAACGTCAGATCAAGGTGCAGCTAATGAAAA
GGTGAGGATGGGTTACAATTATATATTTATAAATACGGTACT-TGAAATGGAGTATCTTA
AAGGAGGACTTGAAAGTAATTTTAATTATATAAATAGAATGAATAAGGCTCTGAAACGTG
CAGAATTTTAATGGCCGAACAGACCAACCCTTAAAGACTTCTGCATCTTTAGGATATTCT
GGTCCAACATCGAGGTCACAAACCTTTTTTTCGATACGGGCTCTTAAAAAAGATAATGCT
GTTATCCCTACGGTAACTAATTTCTTTGATCAAAATTCT-TGGATCAAC-GCAAGTAAGA
CTTA-AAAGGAGGCTTTATTTACTCCTCGGTTGCCCCAACCAAAGT--ATTTAATAGTTA
TGCCTTTTA-CTTAATTA--GCAAAACCTATTAATTTTTCTAAAGCTCGATAGGGTCTTC
TTGTCTTTTAATAATATCTGGACTTTTTCATCCAAAAATAAAATTCTAAACAATCAAAAA
GAGACAGCTGTATTCTTGTCAAACCATTCATTCCAGCCTTCAATTATAAGGCAAATGATT
ATGCTACCTTTGCACGGTCAGAGTACCGCGGCCGTT-TAAAACCACTGGGCAGGTCCGAC
TTCGCATT-AAT-TTTTACACTACATTATATATTCTATTTGGGATATGATCAGGCTTGGT
AGGGACTGCTTTAAGGTTATTAATTCGTGCAGAGTTAGGACAGCCAGGTGCTTTGCTTGG
AGACGATCAGCTATATAATGTAATTGTGACGGCGCATGCTTTTGTTATAATTTTCTTTTT
AGTAATGCCTATGATGATTGGGGGTTTTGGGAATTGATTGGTACCTTTGATATTAGGGGC
TCCAGATATAGTATTTCCTCGCTTAAACAATATGAGTTTTTGGCTTCTTCCCCCTGCACT
TCTACTTCTTTTGTCTTCGGCTGCGGTAGAAAGAGGGGTAGGAACGGGATGAACTGTATA
CCCACCTCTGGCAGGGAATCTTGCTCATGCTGGTGGTTCTGTAGATCTTGCAATTTTTTC
TTTACACCTTGCTGGGGTATCTTCTATTTTAGGGGCTGTAAACTTCATTACTACAATTAT
TAATATACGATGACAGGGAATGAAGTTTGAGCGTCTTTCGTTGTTTGTTTGATCGGTGAA
GATCACGGCGATCTTACTTCTTTTATCCCTACCTGTTTTGGCGGGGGCTATTACTATGCT
TTTAACTGATCGAAATTTCAATACTGCTTTCTTTGATCCAGCAGGGGGTGGTGATCCTAT
TTTGTATCAACATCTTTTT
>stercusmuscarum
AACCAAGAAATTTAGTCATTCTTTTATGGTAAAAAGACAGTTAGGCAAA-TGAGATTATT
AGACCTTTAGTATAGGAGTAAAATCTAAATACTAGAAGTTATACCAGATATAATTT-GTT
TGTGTTGAATCTGTGACAGCTTTAAGGGAAACTGGGATTGGATACCCCATTATTTTTAGT
TATAAATTTAGTT--TAAGCTTACCGGAGTACTACGAATGTTTAAAACTCAAAGGGCTTG
GCGGTGTTTTAGACCTCTCAGGGGAACCTGTCTCGTAATCGACAATCCACGCTAGACCTA
ACCTTTTGTGGTAGTACAGCCTGTATACCGTCGTCGTCAGGTAACTTCTTAAAATATAGT
AGTTAGCTTGAGAATT--TG-ATTAATTAGAACGTCAGATCAAGGTGCAGCTAATGAAAA
GGTGAGGATGGGTTACAATTACATATTTGTAAATACGGTACT-TGAAATGAAGTATCTTA
AAGGAGGACTTGAAAGTAATTTTAATTATATAAATAGAATGAATAAGGCTCTGAAACGTG
CAGAATTTTAATGGTCGAACAGACCAACCCTTAAAGACTTCTGCATCTTTAGGACATTCT
GGTCCAACATCGAGGTCACAAACCTTTTTTTCGATATGGGCTCTTAAAAAAGATAATGCT
GTTATCCCTACGGTAACTAATTTCTTTAATCAAAACTCC-TGGATCAAC-GCAAGTAAGA
CTTA-AAAGGAGGCTTTATTTACTCCTCGGTTGCCCCAACCAAAGT--ATTTAATAGTTA
TACTTTTTA-CTTAATTA--GTAAAACCTATTAATTTTTCTAAAGCTCGATAGGGTCTTC
TTGTCTTTTAATAATATCTGGACTTTTTCATCCAAAAATAAAATTCTAAACAATCAAAAA
GAGACAGTTGTATTCTTGTCAAACCATTCATTCCAGCCTTCAATTATAAGGCAAATGATT
ATGCTACCTTTGCACGGTCAGAGTACCGCGGCCGTT-TAAAATCACTGGGCAGGTCCGAC
TTCGCATT-AAT-TATTACACTACACTATATATTTTATTTGGGATATGATCAGGCTTGGT
AGGGACTGCTTTAAGGCTATTAATTCGTGCAGAGTTAGGACAGCCAGGTGCATTGCTAGG
AGATGATCAACTATATAATGTAATTGTGACGGCGCATGCTTTTGTTATAATCTTCTTTTT
GGTAATGCCTATGATGATTGGGGGTTTTGGAAACTGGTTGGTACCTTTAATATTAGGGGC

TCCAGATATAGTATTTCCTCGTTTAAACAATATGAGTTTTTGGCTTCTCCCTCCTGCGCT
CTTACTCCTTTTGTCTTCGGCTGCGGTAGAAAGTGGGGTAGGAACTGGATGAACTGTATA
TCCGCCTCTGGCAGGGAATCTTGCTCATGCTGGTGGCTCTGTAGATCTTGCAATTTTTTC
TTTACACCTTGCTGGAGTATCTTCTATTTTAGGGGCGGTAAACTTTATCACTACAATCAT
TAATATACGATGACAAGGAATGAAGTTTGAGCGTCTTTCGTTGTTTGTTTGGTCAGTGAA
GATCACGGCAATCTTGCTTCTTTTGTCTCTGCCTGTTTTAGCTGGAGCCATTACTATGCT
TTTAACTGATCGAAATTTTAATACTGCTTTTTTCGATCCAGCAGGAGGTGGTGACCCTAT
TTTATATCAGCATCTTTTT
>floccatus
AACCAAGAAATTTAGTCATTCTTTTATGGTAAAAAGACAGTTAGGCAGG-TGAAATTATT
GGATCTTTAGTAAAGGAGTAAAATCTAAATACTAAAAGTTATACCAGAGATAGTTT-ATT
TATGTTGAATCTGTGACAGCTTTAAGGGAAACTGGGATTGGATACCCCATTATTTTTAGT
TATAAATTCAGTT--TAAGCTTACCGGAGTACTACGAATGTTTAAAACTCAAAGGGCTTG
GCGGTGTTTTAGACCTCTCAGGGGAACCTGTCTCGTAATCGACAATCCACGTTAGACCTA
ACCTTTTATAGTAATACAGCTTGTATACCGTCGTCGTCAGGTAACTTCTTAAAATGTAGC
AGTTAGCTTGAGAATT--TA-ATTAATTAGAACGTCAGATCAAGGTGCAGCTAATGAAAG
GGTGAGGATGGGTTACAATTACATATTTGTAAACACGGTACT-TGAAATGAAGTATCTTA
AAGGAGGACTTGAAAGTAATTCTATTTATATAAATAGAATGAATAAGGCTCTGAAACGTG
CAGAATTTTAATGGTCGAACAGACCAACCCTTAAAGACTTCTGCATCTTTAGGACATTCT
GGTCCAACATCGAGGTCACAAACCTTTTTTTCGATATGGGCTCTTAAAAAAGATAATGCT
GTTATCCCTACGGTAACTAATTTCTTTAATCAAAACTCC-TGGATCAAC-ACAAGTAAGA
CTTA-AAAGGAGGCTTTATTTACTCCTCGGTTGCCCCAACCAAAGT--ATTTAATAGCTA
TGTCTTTTA-CTTGATTA--ACTAAGCCTATTAATTTTTCTAAAGCTCGATAGGGTCTTC
TTGTCTTTTAATAATATTTGGACTTTTTCATCCAAAAATAAAATTCTAAACAATTTAAGA
GAGACAGCTGTATTCTTGTCAAACCATTCATTCCAGCCTTCAATTATAAGGCAAATGATT
ATGCTACCTTTGCACGGTCAGAGTACCGCGGCCGTT-TAAAATCACTGGGCAGGTCCGAC
TTCGTATT-AAT-TATTACACTACATTATATATTTTATTTGGAATATGATCAGGCTTGGT
AGGAACTGCTTTAAGACTATTGATTCGTGCAGAGTTAGGACAGCCCGGTGCTTTGCTTGG
AGACGATCAGTTATATAATGTGATTGTGACGGCGCATGCTTTTGTTATAATTTTCTTTTT
AGTAATGCCTATAATAATTGGGGGGTTTGGGAATTGGTTAGTGCCTTTAATATTAGGGGC
TCCAGACATAGTATTTCCTCGCTTAAATAATATGAGTTTTTGACTTCTTCCTCCTGCACT
TTTACTTCTTTTGTCTTCGGCTGCAGTAGAAAGGGGGGTAGGTACTGGATGGACTGTATA
TCCACCTCTGGCAGGGAATCTTGCTCATGCTGGCGGCTCTGTAGATCTTGCAATTTTTTC
TTTACATCTTGCTGGGGTATCTTCTATTTTAGGAGCGGTAAACTTTATTACTACAATTAT
TAATATGCGATGACAAGGGATGAAATTTGAGCGTCTTTCCTTGTTTGTATGGTCGGTGAA
GATCACGGCTATCTTACTTCTTTTATCTCTACCTGTTTTGGCAGGGGCTATTACTATGCT
TTTAACTGATCGAAATTTCAATACTGCTTTTTTTGACCCAGCAGGAGGTGGTGATCCTAT
CTTGTATCAACATCTTTTT
>koukae
AACCAAGAAATTTAGTCATTTTTTTACGGTAAAAAGACAGTTAGGCGAA-TGAAATCATT
GGACCTTTAGTAGAGGAGTAAAATCTAAATACTAAGAGTTATATCAGACATGGTTT-ATT
TATGCTGAATCTGTGACAGCTTTAAGGGAAACTGGGATTGGATACCCCATTATTTTTAGT
TATAAATTTAGTT--TAGGCTTACCAGAGTACTACGAATGTTTAAAACTCAAAGGGCTTG
GCGGTGTTTTAGACCTCTCAGGGGAACCTGTCTCGTAATCGATAATCCACGTTAAACCTA
GCCTTTTGTGGTAATACAGCTTGTATACCGTCGTCGTCAGGTAACTTCTTAAAATTTAGT
AGTTAGCTTGAGAATT--TG-ATTAATTAAAACGTCAGATCAAGGTGCAGCTAATGAGAA
GGTGAGGATGGGTTACAATTATATATTTGTAAATACGGTACT-TGAAATGAAGTATCTTA
AAGGAGGACTTGAAAGTAATTTTGATTATATAAGCAAAATGAATGCGGCTCTGAAACGTG

NNNNATTTTAATGGTCGAACAGACCAACCCTTAAAGACTTCTGCATCTTTAGGACATTCT
GGTCCAACATCGAGGTCACAAACCTTTTTTTCAATATGGGCTCTTAAAAAAGATAATGCT
GTTATCCCTACGGTAACTAATTCCTTTAATCAAAACTCT-TGGATCAAC-ACAAGTAAGA
CTTA-AAAGGAGGCTTTATTTACTCCTCGGTTGCCCCAACCAAAGT--ATTTAATAGTTA
TATCTTTTA-CTTAATTA--ACGAACCCTACTAATTTATCTAAAGCTCGATAGGGTCTTC
TTGTCTTTTAATAATATCTGGACTTTTTCATCCAAAAATAAAATTCTAAACAATCTAAAA
GAGACAGCTGCATTCTTGTCAAACCATTCATTCCAGCCTTCAATTATAAGGCAAATGATT
ATGCTACCTTTGCACGGTCAGAGTACCGCGGCCGTT-TAAAATCACTGGGCAGGTCCGAC
TTCGTATTNNNN-NNNNNNNNTACATTATATATTCTATTTGGAATATGATCGGGGCTGGT
TGGGACTGCTTTAAGGCTATTAATTCGTGCAGAGTTAGGGCAGCCTGGTGCTTTGCTTGG
GGATGACCAGTTATATAATGTAATTGTAACGGCACATGCTTTTGTTATAATTTTCTTTTT
GGTGATGCCTATAATAATTGGGGGTTTTGGAAACTGGTTGGTACCGCTGATATTAGGGGC
TCCAGACATAGTGTTTCCCCGTTTAAATAATATGAGTTTTTGGCTTCTTCCTCCTGCGCT
TTTACTTCTTTTGTCTTCAGCTGCGGTAGAAAGTGGGGTAGGAACTGGATGAACTGTATA
TCCACCATTGTCGGGGAATTTAGCCCATGCTGGCGGATCTGTAGATCTTGCGATTTTTTC
TTTGCATCTTGCTGGGGTATCTTCTATTTTAGGGGCAGTAAACTTTATTACTACAATTAT
TAATATACGATGACAAGGAATGAAGTTTGAACGTCTTTCGTTATTTGTATGGTCGGTAAA
AATTACGGCTATTTTACTTCTTTTATCTTTACCTGTTTTGGCAGGAGCTATTACTATGCT
TTTGACTGATCGGAATTTTAATACTGCCTTTTTTGATCCAGCAGGAGGCGGAGATCCTAT
TTTATATCAGCATCTTTTT
>gubernator
AACCAAGAAATTTAGTCATTCTTTTGCGGTAAAAAGACAGTTAGGCAAG-AGGAATCATT
GGATCTTTAGTAGAGGAGTAAAATCTAAATACTAAAAGCTATACCGAGTGTGGTTT-ATT
TATGCTGAGTCTGTGACAGCTTCAAGGGAAACTGGGATTGGATACCCCATTATTTTTAGT
TATAAA-TTAGTT--TAGGCTTACCAGAGTACTACGAATGTTTAAAACTCAAAGAGCTTG
GCGGTGTTTTAGACCTCTCAGGGGAACCTGTCTCGTAATCGACAATCCACGTTAAACCTA
ACCTTTTATGGTAATACAGCCTGTATACCGTCGTCGTCAGGTAACTTCTTAAAACATAGT
AGTTAGCTCGAGAATT--TA-ATTAATTAGGACGTCAGATCAAGGTGCAGCTAATGAGAA
GGTGAGGATGGGTTACAATTACATATTTGTAAATACGGCACT-TGAAATGAAGTGTCTTA
AAGGAGGACTTGAAAGTAATTTTAATTATATAGATGGAATGAATGCGGCTCTGAAACGTG
CAGAATTTTAATGGTCGAACAGACCAACCCTTAAAGACTTCTGCATCTTTAGGATATTCT
GGTCCAACATCGAGGTCACAAACCTTTTTTTCGATATGGGCTCTTGAAAAAGATTATGCT
GTTATCCCTACGGTAACTAATTTCTTTAATCAAAACTCC-TGGATCAAC-ACAAGTAAGA
CTTA-AAAGGAGGCTTTATTTACTCCTCGGTTGCCCCAACCAAAGT--ATTTAATAGTTT
TGCCTTTTA-CTTAATCA--GCAAAATCTATTAACTTTTCTAAAGCTCGATAGGGTCTTC
TTGTCTTTTAATAACATTTGGGCTTTTTCACCCAAAAATAAAATTCTAAACAATCTAAAA
GAGACAGCTGTATTCTTGTCAAACCATTCATTCCAGCCTTCAATTATAAGGCAAATGATT
ATGCTACCTTTGCACGGTCAGAGTACCGCGGCCGTT-TAAAA-CACTGGGCAGGTCCGAC
TTCACATT-AAT-CTATACACTACATTATATATTTTATTTGGAATATGATCAGGGTTAGT
AGGGACTGCCTTAAGGTTGTTAATTCGTGCAGAGTTAGGACAGCCAGGTGCTCTGCTTGG
GGATGATCAGCTATATAATGTAATTGTGACGGCTCATGCTTTTGTTATAATCTTTTTCTT
AGTGATGCCTATGATGATTGGAGGTTTTGGGAATTGACTAGTGCCTTTAATATTAGGGGC
TCCAGATATAGTGTTTCCTCGGTTGAATAATATGAGTTTTTGGCTTCTTCCTCCTGCGCT
TTTACTTCTTTTATCTTCGGCCGCGGTAGAAAGCGGAGTAGGTACAGGATGAACTGTATA
TCCACCTTTGGCAGGGAATCTAGCCCACGCCGGTGGTTCTGTGGATCTTGCTATTTTCTC
TTTACACCTTGCTGGTGTGTCTTCTATTTTAGGGGCGGTAAATTTTATTACCACAATTAT
TAATATGCGATGACAAGGAATGAAATTTGAGCGTCTTTCGTTGTTTGTTTGATCGGTAAA
GATTACTGCTATCTTGCTTCTTTTATCTTTGCCTGTTTTGGCAGGGGCTATTACTATGCT

TTTAACTGATCGAAATTTTAATACTGCTTTTTTTGACCCGGCAGGAGGTGGTGATCCTAT
TTTGTATCAGCATCTGTTT
>striatus
AACCAAGAAATTTAGTCATTCTTTTATGGTAAAAAGACAGTTAGGCAGG-TAAAATCGTT
GGACCTTTAGTAGAGGAGTAAAATCTAAATACTAAAAGTTATACCGGGGATGATTT-ATT
TATGCTGAGTCTGTGACAGCTTTAAGGGAAACTGGGATTGGATACCCCATTATTTTTAGT
TATAAA-TTAGTT--TAAGCTTACCAGAGTACTACGAATGTTTAAAACTCAAAGAGCTTG
GCGGTGTTTTAGACCTCTCAGGGGAACCTGTCTCATAATCGACAATCCACGTTAAACCTA
ACCTTTTATAGTAGTTCAGCTTGTATACCGTCGTCGTCAGGTAACTTCTTAAAATATAGT
AGTTAGCTCGAGAATT--GG-ATTAATTAGAACGTCAGATCAAGGTGCAGCTAATGAGAA
GGTGAGGATGGGTTACAATTACATATTTGTAAATACGGCACT-TGAAATGAAGTGTCTTA
AAGGAGGACTTGAAAGTAATTTTGATTATATAAATGGAATGAATGCGGCTCTGAAACGTG
CAGAATTTTAATGGTCGAACAGACCAACCCTTAAAGACTTCTGCATCTTTAGGATATTCT
GGTCCAACATCGAGGTCACAAACCTTTTTTTCGATATGGGCTCTTGAAAAAGATAATGCT
GTTATCCCTACGGTAACTAATTTCTTTAATCAAAATTCC-TGGATCAAC-ACAAGTAAGA
CTTA-AAAGGAGGTTTTATTTGCTCCCCGGTTGCCCCAACCAAAGT--ATTTAATAGCTT
TGCTTTTTA-CTTAATTA--ATAAAATCTATTAATTTTTCTGAAGCTCGATAGGGTCTTC
TTGTCTTTTAATAATATTTGGACTTTTTCATCCAAAGATAAAATTCTAAACAATCTAAAA
GAGACAGCTGTATTCTTGTCAAACCATTCATTCCAGCCTTCAATTATAAGGCAAATGATT
ATGCTACCTTTGCACGGTCAGAGTACCGCGGCCGTT-TAAAA-CACTGGGCAGGTCCGAC
TTCGTATT-AAC-TTATACACTACATTATATATTTTATTTGGAATGTGATCAGGACTAGT
AGGGACTGCTTTAAGGTTGTTGATTCGTGCAGAGTTGGGACAGCCAGGTGCTTTGCTTGG
GGATGATCAGCTATATAATGTAATTGTGACGGCCCATGCTTTTGTTATAATTTTTTTCTT
AGTGATGCCTATAATGATTGGGGGTTTTGGAAATTGGTTGGTGCCTCTTATATTAGGGGC
TCCAGATATAGTATTTCCTCGATTAAATAATATGAGTTTTTGGCTTCTTCCTCCTGCTCT
TCTGCTTCTTCTATCTTCGGCTGCGGTGGAGAGAGGGGTAGGTACTGGATGAACTGTGTA
TCCACCTCTGGCAGGGAATTTAGCACATGCTGGTGGTTCTGTAGATCTTGCTATTTTCTC
TTTACATCTTGCTGGTGTCTCTTCCATTTTAGGGGCGGTAAATTTTATTACTACAATTAT
TAATATACGATGACAAGGAATGAAATTTGAGCGTCTTTCATTGTTTGTTTGATCGGTAAA
GATTACGGCTATTTTGCTTCTTTTGTCTTTGCCTGTTCTGGCGGGGGCTATTACTATACT
TTTGACTGATCGAAATTTTAATACTGCTTTCTTTGATCCAGCAGGAGGTGGTGATCCCAT
TTTATATCAACACCTGTTT
>andamanesis
AACCAAGAAATTTAGTCATTCTTTTATGGTAAAAAGACAGTTATGCAGA-TATAATCATT
AGATCTTCAGTAAAGGAGTAAAATCTATATACTGAAAGTTATACTAGTTGTGATAC-ACT
TATGCTGAATCTGTGATAGCTTTAAGGGAAACTGGGATTGGATACCCCATTATTTTTAGC
CGTAAATTCAGTT--TGAACTTACCAGAGTACTACGAATATTTAAAACTCAAAGGGCTTG
GCGGTGTTTTAGACCTCTCAGGGGAACCTGTCTCGTAATCGACAATCCACGTTAAACCTA
ACCCTTTATGGTAATACAGTTTGTATACCGTCGTCGTCAGGTAACTTCTTAAAATATAGT
AGTTAGCTCGAGAATT--CC-ATTAATTAGAACGTCAGATCAAGGTGCAGCCAATAAAAG
GGTGAGGATGGGTTACAATTATATATTTATAATTACGGCACT-TGAAATCGAGTGC-TTA
AAGGAGGACTTGAAAGTAATTTTAATTATATAAATAAAATGAATTAGGCTCTGAAACGTG
CAGAATTTTAATGGTCGAACAGACCAACCCTTAAAGACTTCTGCATCTTTAGGACATTCT
GGTCCAACATCGAGGTCACAAACCTTTTTTTCGATATGGGCTCTTGAAAAAGATAATGCT
GTTATCCCTACGGTAACTAATTCCTTTAATCAAAACTCT-TGGATCAAT-TCAAGTAGGA
TTTACAAAGGAGGCTCTAACTACTCCTCGGTTGCCCCAACCAAAGT--GTTTAATAGCTT
T-TCTTCTA-CTTAATTA--A-AAAGTCTATTAACTTTTCTGAAGCTCGATAGGGTCTTC
TTGTCTTTTAATAATATTTGGACTTTTTCATCCAAAAATAAAATTCTAAACAATCAAAAA

GAGACAGATGTATTCTTGTCAAACCATTCATTCCAGCCTTCAATTATAAGGCAAATGATT
ATGCTACCTTTGCACGGTCAGAGTACCGCGGCCGTT-TAAAA-CACTGGGCAGGTCCGAC
TTCGCATTTAAA-TCTGACACGACATTGTATATTTTATTTGGGATGTGATCTGGCTTAGT
TGGAACGGCTTTAAGATTATTAATTCGTGCAGAATTAGGGCAGCCTGGTGCTTTGCTTGG
AGACGATCAATTATACAATGTAATTGTAACAGCACATGCTTTTGTTATAATTTTTTTCTT
AGTAATACCCATAATGATTGGGGGTTTTGGGAACTGATTAGTACCGCTAATATTGGGGGC
TCCAGATATAGTGTTTCCTCGTTTAAATAATATGAGTTTTTGACTTCTTCCTCCAGCGCT
TTTACTTCTTTTATCGTCAGCCGCAGTGGAAAGTGGAGTAGGTACGGGATGAACTGTATA
TCCGCCTTTAGCAGGGAATTTAGCTCACGCCGGTGGTTCAGTAGATCTGGCAATTTTTTC
TCTGCATCTTGCTGGTGCCTCTTCTATCTTAGGAGCAGTAAATTTCATTACCACAATTAT
TAACATACGGTGACAGGGAATAAAATTTGAACGTCTTTCATTGTTTGTTTGATCAGTAAA
GATTACAGCTATCTTACTTCTTTTGTCATTACCTGTATTAGCAGGTGCAATTACTATACT
TCTAACTGATCGAAATTTTAATACTGCATTTTTTGACCCGGCAGGAGGTGGTGACCCTAT
TTTGTATCAACATTTATTT
>blanfordianus
AACCAAGAAATTTAGTCATTCTTTTGTGGTAAAAAGACAGTTATGCAGA-TATAATCATT
AGATCTTTAGTAAAGGAGTAAAATCTATATACTAAAAGCTATATTAGTTGTGATGT-GCT
TATGCTGAATCTGTGACAGCTTTAAGGGAAACTGGGATTGGATACCCCATTATTTTTAGC
CGTAAATTTAGTT--TGAACTTACCAGAGTACTACGAATATTTAAAACTCAAAGGGCTTG
GCGGTGTTTTAGACCTCTCAGGGGAACCTGTCTCATAATCGACAATCCGCGTTAGACCTA
ACCCTTTATGGTAATACAGCCTGTATACCGTCGTCGTCAGGTAACTTCTTAAAACATAGT
AGTTAGCTGGAGAATT--TT-ATTAATTAGAACGTCAGATCAAGGTGCAGCTAATAAGAG
GGTGAGGATGGGTTACAATTATATATTTATAACTACGGCACT-AGAAATTGAGTGC-TTA
AAGGAGGACTTGAAAGTAATTTTAATTACATAAATAAAATGAATTAGGCTCTGAAACGTG
NAGAATTTTAATGGTCGAACAGACCAACCCTCAAAGACTTCTACATCTTTAGGACATTCT
GGTCCAACATCGAGGTCACAAACCTTTTTTTCGATATGGGCTCTTAAAAAAGATAATGCT
GTTATCCCTACGGTAACTAATTCCTTTGATCAAAACTCT-TGGATCAAC-TCAAGCAGGA
TTTATAAAGGAGGCTCTAGCTACTCCTCGGTTGCCCCAACCAAAGT--GTTTAATAGCTT
T-TCTTTTA-CTTAATTA--A-AAAGCCTATTAATTTTTCTGAAGCTCGATAGGGTCTTC
TTGTCTTTTAATAATATTTGGATTTTTTCATCCAAAAATAAAATTCTAAACAATCAAAAA
GAGACAGGTGTATTCTTGTCAAACCATTCATTCCAGCCTTCAATTATAAGGCAAATGATT
ATGCTACCTTTGCACGGTCAGAGTACCGCGGCCGTT-TAAAT-CACTGGGCAGGTCCGAC
TTCGTATTTAGG-TCTGACACGACATTGTATATTTTATTTGGGATGTGATCCGGCTTAGT
TGGAACGGCTTTAAGATTGCTAATTCGTGCAGAATTAGGGCAGCCTGGTGCTTTGCTTGG
AGACGATCAACTATACAATGTAATTGTAACAGCACATGCTTTTGTTATAATTTTTTTCTT
AGTAATGCCCATGATAATCGGGGGATTTGGAAACTGATTAGTACCGTTAATGTTGGGGGC
TCCTGATATAGTGTTTCCTCGTTTAAATAATATGAGTTTCTGACTTCTTCCTCCGGCGTT
ATTACTTCTTTTGTCGTCAGCCGCAGTGGAAAGTGGAGTAGGTACGGGATGAACTGTATA
TCCGCCTTTAGCAGGGAATTTAGCTCACGCCGGGGGTTCAGTGGATCTGGCAATTTTTTC
TTTACATCTTGCCGGTGCGTCTTCTATTTTGGGGGCAGTAAACTTTATTACCACAATTAT
TAATATACGGTGACAGGGAATAAAATTTGAACGCCTTTCATTGTTCGTTTGATCGGTAAA
AATTACAGCTATCTTACTTCTTTTGTCATTGCCTGTATTAGCGGGTGCAATTACTATGCT
TCTAACTGATCGAAATTTTAATACTGCATTTTTTGATCCGGCAGGAGGTGGTGATCCTAT
TTTGTACCAACATTTATTT
>australis
AACCAAGAAATTTAGTCATTCTTCTACGGTAAAAAGACAGTTATACAAA-TATAATCATT
AGATCTTCAGTAGAGGAGTAAAATCTATATACTGAAAGTTATACTAAACATGATTT-ACT
TATGTTGAATCTGTGACAGCTTTAAGGGAAACTGGGATTGGATACCCCATTATTTTTAGT

CGTAAATTTAGTT--TAAGCTTACCAGAGTACTACGAATGTTTAAAACTCAAAGGGCTTG
GCGGTGTTTTAGACCTCTCAGGGGAACCTGTCTCGTAATCGACAATCCACGTTAGACCTA
ACCTTTTGTAGTAATACAGCCTGTATACCGTCGTCGTCAGGTAACTTCTTAAAATATAGT
AGTTAGCTCGAGAATT--TT-ATTAATTAAAACGTCAGATCAAGGTGCAGCTAATAAAAA
GGTGAGGATGGGTTACAATTATATATTTGTAAATACGGCACT-TGAAATGAAGTGTCTTA
AAGGAGGACTTGAAAGTAATTTTAATTATATAAGTGAAATGAATAAGGCTCTGAAACGTG
CAGAATTTTAATGGTCGAACAGACCAACCCTTAAAGACTTCTGCATCTTTAGGACATTCT
GGTCCAACATCGAGGTCACAAACCTTTTTTTCGATATGAGCTCTTGAAAAAGATAATGCT
GTTATCCCTACGGTAACTAATTCCTTTGATCAAAATTCT-TGGATCAAC-ACAGGTAAGA
TTTA-AAAGGAGGCTCTAATTACTCCTCGGTTGCCCCAACCAAAGT--ATTTAATAGCTT
T-TCTTCTA-CTTAATTG--ATAAAGCCTACTAATTTTTCTGAAGCTCGATAGGGTCTTC
TTGTCTTTTAATAACATTTGGACTTTTTCATCCAAAAATAAAATTCTAAACAATCAAAAA
GAGACAGCTGTATTCTTGTCAAACCATTCATTCCAGCCTTCAATTATAAGGCAAATGATT
ATGCTACCTTTGCACGGTCAGAGTACCGCGGCCGTT-TAAAA-CACTGGGCAGGTCCGAC
TTCGTATTTGTT-TCTGACACGACATTATATATTTTATTTGGGATGTGGTCCGGCTTAGT
TGGAACGGCTTTAAGACTGTTAATTCGTGCAGAATTAGGGCAGCCGGGTGCTTTACTTGG
AGATGATCAACTATATAATGTGATTGTAACGGCCCATGCTTTTGTTATAATTTTTTTCTT
AGTAATGCCTATGATGATTGGAGGTTTTGGAAATTGATTGGTACCTTTAATATTAGGGGC
TCCAGATATAGTATTTCCTCGCTTAAACAATATAAGTTTTTGGCTTCTTCCTCCGGCACT
TTTACTTCTTTTGTCGTCAGCTGCAGTAGAAAGTGGGGTAGGTACAGGATGAACTGTATA
TCCACCTTTGGCTGGAAATTTAGCTCATGCTGGTGGTTCTGTAGATCTGGCGATTTTTTC
TTTACATCTTGCTGGTGTATCTTCTATTTTAGGAGCAGTGAATTTCATTACTACAATTAT
TAATATACGGTGACAAGGGATAAAATTTGAGCGTCTTTCCTTGTTTGTTTGGTCGGTGAA
AATTACGGCCATTTTACTTCTTTTGTCTTTACCTGTATTAGCGGGTGCAATTACTATGCT
TTTAACTGATCGGAACTTTAATACTGCATTTTTTGATCCGGCAGGAGGTGGTGATCCTAT
CTTATATCAACATTTGTTT
>laterculatus
AACCAAGAAATTTAGTCATTCTTTTACGGTAAAAAGACAGTTATACAAG-TATAATCATT
AGATCTTCAGTAGAGGAGTAAAATCTATATACTGAAAGTTATACTAGATGTGATTT-ACT
AGTGTTGAATCTGTGACAGCTTTAAGGGAAACTGGGATTGGATACCCCATTATTTTTAGT
CGTAAATTTAGTT--TAAACTTACCGGAGTACTACGAATTTTTAAAACTCAAAGGGCTTG
GCGGTGTTTTAGACCTCTCAGGGGAACCTGTCTCGTAATCGACAATCCACGTTAGACCTA
ACCCTTTATGGTAATACAGCCTGTATACCGTCGTCGTCAGGTAACTTCTTAAAATATAGT
AGTTAGCTCGAGAATT--TT-ATTAATTAAAACGTCAGATCAAGGTGCAGCTAATGAAAG
GGTGAGGATGGGTTACAATTATATATTTGTAAATACGGCACT-TGAAATGAAGTGTCTTA
AAGGAGGACTTGAAAGTAATTTTAATTATATAAGTAAAATGAATAAGGCTCTGAAACGTG
CAGAATTTTAATGGTCGAACAGACCAACCCTTAAAGACTTCTGCATCTTTAGGACATTCT
GGTCCAACATCGAGGTCACAAACCTTTTTTTCGATATGGGCTCTTGAAAAAGATAATGCT
GTTATCCCTACGGTAACTAATTTCTTTGATCAAAATTCT-TGGATCCAC-ACAAGTAAGA
CTTA-AAAGGAGGCTCTAATTACTCCTCGGTTGCCCCAACCAAAGT--ATTTAATAGTTT
T-TCTTTTA-CTTAATTGATATAAAGCCTACTAATTTTTCTGAAGCTCGATAGGGTCTTC
TTGTCTTTTAATAATATCTGGACTTTTTCATCCAAAAATAAAATTCTAAATAATCAAAAA
GAGACAGGTGTATTCTTGTCAAACCATTCATTCCAGCCTTCAATTATAAGGCAAATGATT
ATGCTACCTTTGCACGGTCAGAGTACCGCGGCCGTT-TAAAA-CACTGGGCAGGTCCGAC
TTCGTATTT-AT-TCTGACACGACATTATATATTTTGTTTGGGATGTGGTCTGGTTTAGT
TGGAACGGCTTTAAGATTATTGATTCGTGCAGAGTTAGGGCAGCCGGGTGCTTTACTTGG
AGATGATCAGCTATATAATGTAATTGTAACAGCTCATGCTTTTGTTATAATTTTTTTCTT
AGTGATACCCATGATAATTGGGGGTTTTGGAAATTGATTGGTACCTTTGATATTAGGGGC

TCCAGACATAGTATTTCCTCGTTTGAATAATATAAGTTTTTGACTTCTTCCTCCGGCACT
TTTACTTCTTTTATCGTCAGCTGCAGTGGAAAGAGGGGTAGGTACAGGATGAACTGTATA
TCCACCTTTAGCCGGAAATTTGGCTCACGCCGGTGGCTCTGTAGATCTGGCGATTTTTTC
TTTACATCTTGCTGGTGTGTCTTCTATTTTAGGAGCAGTGAATTTCATTACTACGATTAT
TAATATGCGGTGACAGGGGATAAAATTTGAACGTCTTTCTCTGTTTGTTTGATCGGTAAA
AATTACAGCTATCTTACTTCTTTTGTCTTTACCTGTATTAGCGGGTGCAATTACTATGCT
TTTAACTGATCGAAATTTTAATACTGCATTTTTTGACCCGGCAGGGGGTGGTGATCCTAT
TTTATATCAACATTTGTTT
>neptunus
AACCAAGAAATTTAGTCATTCTTTTACGGTAAAAAGACAGTTATACAAG-TATAATCATT
AGATCTTCAGTAGAGGAGTAAAATCTATATACTGAAAGTTATACTAGGTATGGTTT-ATT
TATGTTGAATCTGTGACAGCTTTAAGGGAAACTGGGATTGGATACCCCATTATTTTTAGT
CGTAAATTTAGTT--TAAACTTACCGGAGTACTACGAATATTTAAAACTCAAAGGGCTTG
GCGGTGTTTTAGACCTCTCAGGGGAACCTGTCTCGTAATCGACAATCCACGTTAGACCTA
ACCCTTTATGGTAATACAGCCTGTATACCGTCGTCGTCAGGTAACTTCTTAAAATGTAGT
AGTTAGCTCGAGAATT--TT-ATTAATTAAAACGTCAGATCAAGGTGCAGCTAATGAAAG
GGTGAGGATGGGTTACAATTATATATTTGTAAGTACAGCACT-TGAAATGAAGTGTCTTA
AAGGAGGACTTGAAAGTAATTTTAATTATATAAGTAAAATGAATAAGGCTCTGAAACGTG
CAGAATTTTAATGGTCGAACAGACCAACCCTTAAAGACTTCTGCATCTTTAGGACATTCT
GGTCCAACATCGAGGTCACAAACCTTTTTTTCGATATGGGCTCTTGAAAAAGATAATGCT
GTTATCCCTACGGTAACTAATTCCTTTGATCAAAATTTT-TGGATCAAC-ACAAGTAAGA
CTTA-AAAGGAGGCTCTAATTACTCCTCGGTTGCCCCAACCAAAGT--ATTTAGTAGCTT
T-TCTTTTA-TTTGATTG--ATAAAGCCTACTAATTTTTCTAAAGCTCGATAGGGTCTTC
TTGTCTTTTAATAATATCTGGACTTTTTCATCCAAAAATAAAATTCTAAATAATCAAAAA
GAGACAGGTGTATTCTTGTCAAACCATTCATTCCAGCCTTCAATTATAAGGCAAATGATT
ATGCTACCTTTGCACGGTCAGAGTACCGCGGCCGTT-TAAAA-CACTGGGCAGGTCCGAC
TTCGTATTTAAA-TTTGACACGACATTATACATTTTATTTGGGATGTGGTCCGGTTTAGT
TGGAACGGCTTTAAGATTGTTGATTCGTGCAGAATTAGGACAGCCGGGTGCTTTACTTGG
GGATGATCAGCTATATAATGTGATTGTAACAGCCCATGCTTTTGTTATAATTTTTTTCTT
AGTGATGCCTATGATGATTGGAGGTTTTGGAAATTGATTAGTACCTTTGATATTAGGGGC
TCCAGATATAGTGTTTCCTCGCTTAAATAATATAAGTTTTTGACTTCTTCCTCCGGCGCT
TTTACTTCTTTTGTCGTCAGCTGCAGTAGAGAGAGGGGTAGGTACAGGGTGAACTGTGTA
TCCACCTTTGGCCGGAAATTTAGCTCATGCCGGTGGTTCTGTAGATCTGGCAATTTTTTC
TCTACATCTTGCTGGTGTGTCTTCTATTTTAGGAGCAGTGAACTTCATTACTACAATTAT
TAATATGCGGTGGCAGGGGATAAAATTTGAGCGTCTTTCCTTGTTTGTTTGGTCAGTGAA
AATTACGGCTATTTTACTTCTTTTATCTTTGCCTGTATTAGCGGGTGCAATTACTATGCT
TTTAACAGATCGGAATTTTAATACTGCATTCTTTGATCCAGCAGGGGGTGGTGATCCTAT
TTTATATCAGCATTTATTC
>moluccensis
AACCAAGAAATTCAGTCATTCTTTCACGGTAAAAAGACAGTTATACAAG-CATGATCATT
AGACCTTTAGTAGAGGAGTAAAATCTATATACTAAAAGTTATACTGGATATGATTT-ATT
GATGTTGAATCTGTGACAGCTTTAAGGGAAACTGGGATTGGATACCCCATTATTTTTAGT
CGTAAATTTAGTT--TAAACTTACCAGAGTACTACGAATATTTAAAACTCAAAGGGCTTG
GCGGTGTTTTAGACCTCTCAGGGGAACCTGTCTCGTAATCGACAATCCACGTTAGACCTA
ACCCTTTATGGTAATTCAGCCTGTATACCGTCGTCGTCAGGTAACTTCTTAAAATATAGT
AGTTAGCTCGAGAACA--TT-ATTAATTAAAACGTCAGATCAAGGTGCAGCTAATAAGAG
GGTGAGGATGGGTTACAATTACAGATTTGTAAATACGGCATT-TGAAATGGAGTGTTTTA
AAGGAGGACTTGAAAGTAATTTTGATTATATAAATAAGATGAATAAGGCTCTGAAACGTG

CAGAATTTTAATGGTCGAACAGACCAACCCTTAAAGACTTCTGCATCTTTAGGACATTCT
GGTCCAACATCGAGGTCACAAACCTTTTTTTCGATATGGGCTCTTGAAAAAGATAATGCT
GTTATCCCTACGGTAACTAATTCCTTTGATCAAAATTCC-TGGATCTGC-ACAAGTAGGA
CTTA-AAAGGAGGCTCTAATTACTCCTCGGTTGCCCCAACCAAAGT--ATTTAATAGCTT
T-TCTTTTA-CTTAATAG--ATGAAGCCTACTAATTTTTCTGAAGCTCGATAGGGTCTTC
TTGTCTTTTAATAATATCTGGACTTTTTCATCCAAAAATAAAATTCTAAATAATCAAAAA
GAGACAGGTATATTCTTGTCAAACCATTCATTCCAGCCTTCAATTATAAGGCAAATGATT
ATGCTACCTTTGCACGGTCAGAGTACCGCGGCCGTT-TAAAA-CACTGGGCAGGTCCGAC
TTCGTATTTAAT-TTTGACACGACATTATATATTTTATTCGGGATATGGTCCGGTTTAGT
TGGAACGGCTTTAAGGTTGTTAATTCGTGCAGAATTAGGGCAGCCGGGTGCTTTACTTGG
AGATGATCAGCTATATAATGTGATTGTAACGGCCCATGCTTTTGTTATAATTTTTTTCTT
AGTGATGCCTATAATGATTGGAGGTTTTGGGAATTGATTAGTGCCTTTGATATTAGGGGC
TCCAGATATAGTATTTCCTCGTTTGAATAATATAAGTTTTTGGCTTCTTCCTCCGGCACT
TTTACTTCTTCTATCGTCAGCTGCAGTAGAAAGGGGGGTAGGTACGGGATGAACTGTATA
CCCGCCTTTGGCTGGAAATTTGGCTCACGCCGGTGGTTCTGTGGATCTAGCGATTTTTTC
TTTACACCTTGCTGGTGTATCTTCTATTTTAGGGGCAGTGAACTTTATTACTACAATTAT
TAACATGCGATGACAGGGAATAAAGTTTGAGCGTCTTTCTTTGTTTGTTTGGTCGGTGAA
GATTACGGCTATTTTACTTCTTTTGTCTTTACCTGTATTGGCGGGTGCAATTACTATGCT
TTTAACTGATCGAAATTTTAATACTGCGTTTTTTGATCCAGCAGGAGGTGGTGATCCTAT
TTTGTATCAACACTTGTTT
>cebuensis
NNNNNNNNNNNNNNNNNNNNNNNNNNNNCGGT-AAAAGACAGTTATACAAG-CATGATCATT
AGACCTTTAGTAGAGGAGTAAAATCTATATACTGAAAGTTATACTGGATATGATTT-ATT
GATGTTGAATCTGTGACAGCTTTAAGGGAAACTGGGATTGGATACCCCATTATTTTTAGT
CGTAAATTTAGTT--TAAACTTACCAGAGTACTACGAATATTTAAAACTCAAAGGGCTTG
GCGGTGTTTTAGACCTCTCAGGGGAACCTGTCTCGTAATCGACAATCCGCGTTAAACCTA
ACCCTTTATGGTAATTCAGCCTGTATACCGTCGTCGTCAGGTAACTTCTTAAAATATAGT
AGTTAGCTCGAGAACA--TT-ATTAATTAAAACGTCAGATCAAGGTGCAGCTAATAAGAG
GGTGAGGATGGGTTACAATTACAGATTTGTAAATACGGCATT-TGAAACGAAGTGTCTTA
AAGGAGGACTTGAAAGTAATTTTAATTATATAAATAAGATGAATAAGGCTCTGAAACGTG
NNNNATTTTAATGGTCGAACAGACCAACCCTTAAAGACTTCTGCATCTTTAGGACATTCT
GGTCCAACATCGAGGTCACAAACCTTTTTTTCGATATGGGCTCTTGAAAAAGATAATGCT
GTTATCCCTACGGTAACTAATTCCTTTGATCAAAATTCT-TGGATCTAC-ACAAGTAAGA
CTTA-AAAGGAGGCTCTAATTACTCCTCGGTTGCCCCAACCAAAGT--ATTTAATAGCTT
T-TCTTTTA-CTTAATAG--ATCAAGCCTACTAATTTTTCTGAAGCTCGATAGGGTCTTC
TTGTCTTTTAATAATATCTGGACTTTTTCATCCAAAAATAAAATTCTAAATAATCAAAAA
GAGACAGATGTATTCTTGTCAAACCATTCATTCCAGCCTTCAATTATAAGGCAAATGATT
ATGCTACCTTTGCACGGTCAGAGTACCGCGGCCGTT-TAAAA-CACTGGGCAGGTCCGAC
TTCGTATNNNNN-NNNNNNNNNNCATTATATATTTTATTTGGGATATGGTCTGGTTTAGT
TGGAACGGCTTTAAGGTTGTTAATTCGTGCAGAATTAGGGCAGCCGGGTGCTTTACTTGG
AGATGATCAGCTATATAATGTGATTGTAACGGCCCATGCTTTTGTTATAATTTTTTTCTT
AGTGATGCCTATAATGATTGGAGGTTTTGGGAATTGATTAGTGCCTTTGATATTAGGGGC
TCCAGATATAGTATTTCCTCGTTTAAATAATATAAGTTTTTGGCTTCTTCCTCCGGCACT
TTTACTTCTTCTGTCGTCAGCTGCAGTAGAAAGGGGGGTAGGTACGGGATGAACTGTATA
CCCGCCTTTGGCTGGAAATTTGGCTCACGCCGGTGGTTCTGTGGATCTAGCGATTTTTTC
TTTACACCTTGCTGGTGTATCTTCTATTTTAGGAGCAGTGAACTTTATTACTACGATTAT
TAACATACGATGACAGGGGATAAAGTTTGAGCGTCTTTCTTTGTTTGTTTGGTCGGTGAA
GATTACGGCTATTTTACTTCTTTTGTCTTTACCTGTATTGGCAGGTGCAATTACTATGCT

TTTAACTGATCGAAATTTTAATACTGCGTTTTTTGACCCAGCAGGAGGTGGTGATCCTAT
TTTGTATCAACACTTGTTT
>goudeyi
AACCAAGAAATTCAGTCATTCTTTTACGGTAAAAAGACAGTTATACAAG-CATGATCATT
AGACCTTTAGTAGAGGAGTAAAATCTATATACTAAAAGTTATACTGGATATGATTT-ATT
GATGTTGAATCTGTGACAGCTTTAAGGGAAACTGGGATTGGATACCCCATTATTTATAGT
CGTAAATTTAGTT--TAAACTTACCAGAGTACTACGAATATTTAAAACTCAAAGGGCTTG
GCGGTGTTTTAGACCTCTCAGGGGAACCTGTCTCGTAATCGACAATCCACGTTAGACCTA
ACCCTTTGTGGTAATTCAGCCTGTATACCGTCGTCGTCAGGTAACTTCTTAAAATATAGT
AGTTAGCTCGAGAACA--TT-ATTAATTGAAACGTCAGATCAAGGTGCAGCTAATAAAAG
GGTGAGGATGGGTTACAATTACAGATTTGTAAATACGGCATT-TGAAATGAAGTGTCTTA
AAGGAGGACTTGAAAGTAATTTTAATTATATAAATAAAATGAATAAGGCTCTGAAACGTG
CAGAATTTTAATGGTCGAACAGACCAACCCTTAAAGACTTCTGCATCTTTAGGACATTCT
GGTCCAACATCGAGGTCACAAACCTTTTTTTCGATATGGGCTCTTGAAAAAGATAATGCT
GTTATCCCTACGGTAACTAATTCCTTTGATCAAAATTCC-TGGATCTAC-ACAAGTAAGA
CTTA-AAAGGAGGCTCTAATTACTCCTCGGTTGCCCCAACCAAAGT--ATTTAATAGCTT
T-TCTTTTA-CTTAATAG--ATAAAACCTACTAATTTTTCTGAAGCTCGATAGGGTCTTC
TTGTCTTTTAATAATATCTGGACTTTTTCATCCAAAAATAAAATTCTAAATAATCAAAAA
GAGACAGGTGTATTCTTGTCAAACCATTCATTCCAGCCTTCAATTATAAGGCAAATGATT
ATGCTACCTTTGCACGGTCAGAGTACCGCGGCCGTT-TAAAA-CACTGGGCAGGTCCGAC
TTCGTATTTAAT-TTTGACACGACATTATATATTTTATTTGGGATGTGGTCTGGTTTAGT
TGGAACGGCTTTAAGGTTGTTAATTCGTGCAGAATTAGGGCAGCCGGGTGCTTTACTTGG
AGATGATCAGCTGTATAATGTGATTGTAACGGCCCATGCTTTTGTTATAATTTTTTTCTT
AGTGATGCCTATAATGATTGGAGGTTTTGGGAATTGATTAGTGCCTTTGATATTAGGGGC
TCCAGATATAGTGTTTCCTCGTTTGAATAATATAAGTTTTTGGCTTCTTCCTCCGGCACT
TTTACTTCTTCTGTCGTCAGCTGCAGTAGAGAGGGGGGTAGGTACAGGATGAACTGTATA
CCCGCCTTTGGCTGGAAATTTGGCTCACGCCGGTGGTTCTGTGGATCTAGCGATTTTTTC
TTTACATCTTGCTGGTGTATCTTCTATTTTAGGAGCAGTGAACTTTATTACTACAATTAT
TAATATACGATGACAGGGAATAAAGTTTGAGCGTCTTTCTTTGTTTGTTTGGTCGGTGAA
GATTACGGCTATTTTACTTCTTTTGTCCTTACCTGTATTGGCGGGTGCAATTACTATGCT
TTTAACTGATCGAAATTTTAATACTGCATTTTTTGACCCAGCAGGAGGTGGTGATCCTAT
TTTATATCAACACTTGTTT
>madecassinus
AACCAAGAAATTTAGTCATTCTTTTATGGTAAAAAGACAGTTATACAAG-TATAATCATT
AGATCTTTAGTAGAGGAGTAAAATCTATATACTGATAGTTATACTAAATATGATTT-GCT
TATGTTGAATCTGTGACAGCTTTAAGGGAAACTGGGATTGGATACCCCATTATTTTTAGT
CGTAAATTTAGTT--TAAACTTACCAGAGTACTACGAATGTTTAAAACTCGAAGGGCTTG
GCGGTGTTTTAGACCTCTCAGGGGAACCTGTCTCGTAATCGACAATCCACGTTAGACCTA
ACCCTTTATAGTAATACAGCCTGTATACCGTCGTCGTCAGGTAACTTCTTAAAATATAGT
AGTTAGCCCGAAAATT--TT-ATTAATTAAAACGTCAGATCAAGGTGCAGCTAATAAGAG
GGTGAGGATGGGTTACAATTATACATTCATAATTACGGCATT-TGAAATGAAGTGT-ATA
AAGGAGGACTTGAAAGTAATTTTAATTATATAAATAAAATGAATAAGGCTCTGAAACGTG
CAGAATTTTAATGGTCGAACAGACCAACCCTTAAAGACTTCTGCATCTTTAGGACATTCT
GGTCCAACATCGAGGTCACAAACCTTTTTTTCGATATGGGCTCTTGAAAAAGATAATGCT
GTTATCCCTACGGTAACTAATTCCTTTGATCAAAACTCT-TGGATCAAC-ATAAGTAAGA
TTTA-AAAGGAGGCTCTAATTACTCCTCGGTTGCCCCAACCAAAGT--ATTTAATAGCTT
C-TCTTTTA-TATAATTG--ATAAAGCCTACTAATTTTTCTAAAGCTCGATAGGGTCTTC
TTGTCTTTTAATAATATTTGAACTTTTTCATCCAAAAATAAAATTCTAAACAATCAAAAA

GAGACAGGTGTATTCTTGTCAAACCATTCATTCCAGCCTTCAATTATAAGGCAAATGATT
ATGCTACCTTTGCACGGTCAGAGTACCGCGGCCGTT-TAAAA-CACTGGGCAGGTCCGAC
TTCGTATTTAAT-TTCGACACGACATTGTACATTTTATTTGGGATGTGATCTGGTTTAGT
TGGAACGGCTTTAAGGTTGTTAATTCGTGCAGAATTAGGACAGCCGGGTGCTTTACTTGG
AGATGATCAGTTATATAATGTAATTGTAACAGCCCATGCTTTTGTTATAATTTTTTTCTT
AGTAATACCTATGATGATTGGGGGTTTTGGAAATTGATTAGTACCTTTAATATTAGGAGC
TCCAGATATAGTGTTTCCTCGTTTAAATAATATAAGTTTTTGGCTTCTTCCTCCGGCACT
TTTACTTCTTTTATCATCAGCTGCAGTGGAGAGTGGGGTAGGTACAGGATGAACTGTATA
TCCGCCTTTAGCTGGAAATTTAGCTCATGCTGGTGGTTCTGTAGATTTGGCGATTTTTTC
TTTACATCTTGCTGGAGCGTCTTCTATTTTAGGAGCGGTAAACTTCATTACCACAATTAT
TAATATGCGGTGACAAGGAATAAAATTTGAGCGTCTTTCTTTATTTGTTTGGTCAGTAAA
GATTACGGCTATTTTACTTCTTTTATCTTTACCTGTGTTAGCAGGTGCAATTACTATGCT
TCTGACTGATCGGAATTTTAATACTGCATTTTTTGATCCGGCAGGAGGTGGCGATCCTAT
TTTATATCAGCATTTATTT
>mucronatus
AACCAAGAAATTTAGTCATTCTTTTACGGTAAAAAGACAGTTATACAAA-GATAATCATT
AGATCTTCAGTAGAGGAGTAAAATCTACATACTGAAAGATATACTAGATGTGGTTT-ACT
TATGTTGAATCTGTGACAGCTTTAAGGGAAACTGGGATTGGATACCCCATTATTTTTAGT
CGTAAATTTAGTT--TAAGCTTACCTGAGTACTACGAATGTTTAAAACTCAAAGGGCTTG
GCGGTGTTTTAGACCCCTCAGGGGAACCTGTCTCGTAATCGACAATCCACGTTAGACCTA
ACCCTTTATGGTAATACAGCTTGTATACCGTCGTCGTCAGGTAACTTTTTAAAATATAGT
AGTTAGCCTGAGAATT--TT-ATTAATTAAAACGTCAGATCAAGGTGCAGCTAATAAAAG
GGTGAGGATGGGTTACAATTATATATTTGTAAATATGGCATT-TGAAATGAAGTGTTTTA
AAGGAGGACTTGAAAGTAATTTTAATTATATAAATAAAATGAATAAGGCTCTGAAACGTG
CAGAATTTTAATGGTCGAACAGACCAACCCTTAAAGACTTCTGCATCTTTAGGACATTCT
GGTCCAACATCGAGGTCACAAACCTTTTTTTCGATATGGGCTCTTGAAAAAGATAATGCT
GTTATCCCTACGGTAACTAATTCCTTTGATCAAAACTCT-TGGATCTAT-ACATGTAAGA
CTTA-AAAGGAGGCTCTAATTACTCCTCGGTTGCCCCAACCAAAGT--GTTTAATAGCTT
T-TCTTTTA-TTTAATTG--A-AAAGCCTATTAATTTTTCTAAAGCTCGATAGGGTCTTC
TTGTCTTTTAATAGCATTTGGACTTTTTCATCCAAAAATAAAATTCTAAACAATCAAAAA
GAGACAGGTGTATTCTTGTCAAACCATTCATTCCAGCCTTCAATTATAAGGCAAATGATT
ATGCTACCTTTGCACGGTCAGAGTACCGCGGCCGTT-TAAAA-CACTGGGCAGGTCCGAC
TTCATATTTAATCCTTAACATGACATTGTATATTTTGTTTGGAATATGATCCGGTTTAGT
TGGAACGGCTTTAAGATTGTTAATTCGTGCAGAATTAGGGCAGCCAGGTGCTTTACTTGG
GGATGACCAGCTGTATAATGTGATTGTAACAGCCCATGCTTTTGTTATAATTTTTTTCTT
AGTAATGCCCATGATGATTGGAGGTTTTGGAAACTGGTTGGTACCTTTGATATTAGGGGC
TCCAGATATAGTATTCCCCCGTTTAAACAATATAAGTTTTTGACTTCTTCCTCCGGCACT
TTTACTTCTCTTATCGTCAGCTGCAGTAGAGAGTGGGGTAGGTACAGGATGAACTGTATA
TCCACCTTTAGCTGGGAATTTGGCTCATGCTGGTGGTTCCGTAGATCTGGCAATTTTCTC
TTTACATCTTGCTGGTGTGTCTTCTATTTTAGGGGCAGTAAACTTTATTACTACGATTAT
TAATATACGGTGACAGGGAATAAAATTTGAGCGTCTTTCCTTGTTTGTTTGGTCAGTAAA
AATTACGGCTATCTTACTTCTCTTATCTTTGCCTGTATTGGCAGGTGCAATCACTATGCT
TTTAACTGATCGGAATTTCAATACTGCATTTTTTGATCCAGCAGGAGGTGGAGATCCTAT
TTTATATCAACATTTGTTT
>sutanorcum
AACCAAGAAATTTAGTCATTCTTTTACGGTAAAAAGACAGTTATACAAA-GATAATCATT
AGATCTTCAGTAGAGGAGTAAAATCTACATACTGAAAGATATACTAGATGTGGTTT-ACT
TATGTTGAATCTGTGACAGCTTTAAGGGAAACTGGGATTGGATACCCCATTATTTTTAGT

CGTAAATTTAGTT--TAAGCTTACCTGAGTACTACGAATGTTTAAAACTCAAAGGGCTTG
GCGGTGTTTTAGACCCCTCAGGGGAACCTGTCTCGTAATCGACAATCCACGTTAGACCTA
ACCCTTTATGGTAATACAGCTTGTATACCGTCGTCGTCAGGTAACTTTTTAAAATATAGT
AGTTAGCCTGAGAATT--TT-ATTAATTAAAACGTCAGATCAAGGTGCAGCTAATAAAAG
GGTGAGGATGGGTTACAATTATATATTTGTAAATATGGCATT-TGAAATGAAGTGTTTTA
AAGGAGGACTTGAAAGTAATTTTAATTATATAAATAAAATGAATAAGGCTCTGAAACGTG
CAGAATTTTAATGGTCGAACAGACCAACCCTTAAAGACTTCTGCATCTTTAGGACATTCT
GGTCCAACATCGAGGTCACAAACCTTTTTTTCGATATGGGCTCTTGAAAAAGATAATGCT
GTTATCCCTACGGTAACTAATTCCTTTGATCAAAACTCT-TGGATCTAT-ACATGTAAGA
CTTA-AAAGGAGGCTCTAATTACTCCTCGGTTGCCCCAACCAAAGT--GTTTAATAGCTT
T-TCTTTTA-TTTAATTG--A-AAAGCCTATTAATTTTTCTAAAGCTCGATAGGGTCTTC
TTGTCTTTTAATAGCATTTGGACTTTTTCATCCAAAAATAAAATTCTAAACAATCAAAAA
GAGACAGGTGTATTCTTGTCAAACCATTCATTCCAGCCTTCAATTATAAGGCAAATGATT
ATGCTACCTTTGCACGGTCAGAGTACCGCGGCCGTT-TAAAA-CACTGGGCAGGTCCGAC
TTCATATTTAACTCCTAACATGACATTGTATATTTTGTTTGGAATATGATCCGGTTTAGT
TGGAACGGCTTTAAGGTTGTTAATTCGTGCAGAATTAGGGCAGCCAGGTGCTTTACTTGG
GGATGATCAGCTATATAATGTGATTGTAACAGCCCATGCTTTTGTTATAATTTTTTTCTT
AGTAATGCCTATGATGATTGGGGGTTTTGGGAACTGGTTAGTGCCTTTGATATTAGGGGC
TCCAGATATGGTATTCCCTCGTTTAAACAATATAAGTTTTTGACTTCTTCCTCCGGCACT
TTTACTTCTCTTGTCGTCAGCTGCAGTAGAAAGTGGGGTAGGTACAGGATGAACTGTATA
TCCACCTTTAGCTGGGAATTTGGCTCATGCCGGTGGTTCTGTAGATCTGGCAATTTTTTC
TTTACATCTTGCTGGTGTGTCTTCTATTTTAGGAGCGGTGAACTTTATTACTACGATTAT
TAATATGCGGTGACAGGGAATAAAATTTGAGCGTCTTTCTTTGTTTGTCTGGTCAGTAAA
AATTACGGCTATCTTACTTCTTTTATCTTTGCCTGTATTGGCGGGTGCAATTACTATGCT
TTTAACTGATCGGAATTTTAATACTGCATTTTTTGATCCAGCAGGAGGTGGAGATCCTAT
TTTATATCAACATTTGTTT
>flavus
AACCAAGAAATTTAGTCATTCTTTTATGGTAAAAAGACAGTTATACAGA-TATAATCATT
AGATCTTTAGTAGAGGAGTAAAATCTATATACTGAAAGTTATACTAGGAGTGGTTT-ACT
TATGTTGAATCTGTGACAGCTTTAAGGGAAACTGGGATTGGATACCCCATTATTTTTGGT
CGTAAATTTAGTT--TAGACTTACCAGAGTACTACGAATGTTTAAAACTCAAAGGGCTTG
GCGGTGTTTTAGACCTCTCAGGGGAACCTGTCTCGTAATCGACAATCCACGTTAGACCTA
ACCCTTTGTGGTAATACAGCCTGTATACCGTCGTCGTCAGGTAACTTCTTAAAATATAGT
AGTTAGCTTGAGAATC--AG-ATTAATTAAAACGTCAGATCAAGGTGCAGCTAATAAAAG
GGTGAGGATGGGTTACAATTATATATTTGTAATTACAGCACT-TGAAATGAAGTGC-TTA
AAGGAGGACTTGAAAGTAATTTTAGTTATATAAGTAAAATGAATAAGGCTCTGAAACGTG
CAGAATTTTAATGGTCGAACAGACCAACCCTTAAAGACTTCTGCATCTTTAGGATATTCT
GGTCCAACATCGAGGTCACAAACCTTTTTTTCGATATGGGCTCTTAAAAAAGATAATGCT
GTTATCCCTACGGTAACTAATTCCTTTGATCAAAAATTT-TGGATCAAC-ACAAGTAAGG
TTTA-AAAGGAGGCTTTAATTACTCCTCGGTTGCCCCAACCAAAGT--GTTTAATAGCTT
T-TCTTTTA-CTTAATTG--ATAAAACCTACTAATTTCTCTTAAGCTCGATAGGGTCTTC
TTGTCTTTTAATAATATTTGGACTTTTTCATCCAAAAATAAAATTCTAAATAATCAAAAA
GAGACAGATGTATTCTTGTTAAACCATTCATTCCAGCCTTCAATTATAAGGCAAATGATT
ATGCTACCTTTGCACGGTCAGAGTACCGCGGCCGTT-TAAAA-CACTGGGCAGGTCCGAC
TTCATATTTGAT-TTCGACATGACATTGTATATTTTATTTGGGATATGATCTGGTTTAGT
TGGAACGGCTTTAAGGTTATTAATTCGTGCAGAGTTAGGACAGCCGGGGGCTTTACTTGG
AGACGATCAGCTATATAATGTAATTGTAACAGCCCATGCTTTTGTTATAATTTTTTTTTT
AGTAATACCTATAATGATTGGGGGTTTTGGAAATTGACTGGTACCATTAATGTTAGGGGC

TCCAGATATAGTGTTTCCTCGTTTGAATAATATAAGTTTTTGGCTTCTTCCTCCGGCGCT
TTTACTTCTCTTATCATCAGCTGCGGTAGAAAGTGGAGTGGGTACGGGATGAACTGTGTA
TCCACCTTTGGCTGGAAATTTAGCTCATGCCGGTGGTTCTGTAGATTTGGCGATTTTTTC
TTTACATCTTGCTGGGGTGTCTTCTATTTTAGGTGCGGTGAATTTCATTACCACGATTAT
TAATATGCGATGACAGGGAATAAAATTTGAGCGCCTTTCTTTGTTTGTCTGATCAGTAAA
AATTACGGCTATTTTACTTCTTTTGTCTTTGCCTGTATTAGCGGGTGCTATTACTATGCT
TTTAACCGATCGAAATTTTAATACTGCCTTTTTTGATCCAGCAGGAGGTGGTGATCCAAT
CTTGTATCAACACTTGTTT
>ochroleucus
AACCAAGAAATTTAGTCATTCTTTTATGGTAAAAAGACAGTTATACAGA-CATAATCATC
AGATCTTTAGTAGAGGAGTAAAATCTATATACTGAAAGTTATACTAGAAGTGGTTT-ACT
TATGTTGAATCTGTGACAGCTTTAAGGGAAACTGGGATTGGATACCCCATTATTTTTAGT
CGTAAATTTAGTT--TAAACTTACCAGAGTACTACGAATGTTTAAAACTCAAAGAGCTTG
GCGGTGTTTTAGACCTCTCAGGGGAACCTGTCTCGTAATCGACAATCCACGTTAGACCTA
ACCCTTTGTGGTAATACAGCCTGTATACCGTCGTCGCCAGGTAACTTCTTAAAATATAGT
AGTTAGCTTGAGAATT--AA-ATTAATTAAGACGTCAGATCAAGGTGCAGCTAATAAAAG
GGTGAGGATGGGTTACAATTATATATTTGTAATTACGGCACT-TGAAATGAAGTGC-TTA
AAGGAGGACTTGAAAGTAATTTTAATTATATGAGTAAAATGAATAAGGCTCTGAAACGTG
CAGAATTTTAATGGTCGAACAGACCAACCCTTAAAGACTTCTGCATCTTTAGGACATTCT
GGTCCAACATCGAGGTCACAAACCTTTTTTTCGATATGGGCTCTTGAAAAAGATAATGCT
GTTATCCCTACGGTAACTAATTCCTTTGATCAAAATTTT-TGGATCAGT-ACAAGTAAGA
CTTA-AAAGGAGGCTCTAGTTACTCCTCGGTTGCCCCAACCAAAGT--GTTTAATAGCTT
T-TCTTTTA-CTTAATTG--ATGAAACCTACTAATTTCTCTTAAGCTCGATAGGGTCTTC
TTGTCTTTTAATAATATTTGGACTTTTTCATCCAAAAATAAAATTCTAAATAATCAAAAA
GAGACAGGTGTATTCTTGTCAAACCATTCATTCCAGCCTTCAATTATAAGGCAAATGATT
ATGCTACCTTTGCACGGTCAGAGTACCGCGGCCGTT-TAAAA-CACTGGGCAGGTCCGAC
TTCATATTTAAT-TCCAACATGACATTGTATATTTTATTTGGGATATGATCTGGTTTAGT
TGGAACGGCTTTAAGATTGTTGATTCGTGCAGAGTTAGGGCAGCCGGGGGCTTTACTTGG
AGATGATCAGCTATATAATGTGATTGTAACGGCCCATGCTTTTGTTATAATTTTTTTTTT
AGTAATACCCATGATGATTGGAGGTTTTGGGAATTGATTGGTACCGTTAATGTTAGGGGC
TCCAGATATGGTGTTTCCTCGCTTGAATAATATAAGTTTTTGGCTTCTTCCTCCAGCGCT
TTTACTTCTTTTATCATCAGCTGCGGTAGAAAGTGGGGTAGGTACGGGATGAACTGTGTA
TCCACCTTTGGCTGGGAATTTAGCTCATGCCGGTGGTTCTGTAGATTTGGCAATTTTTTC
TTTACATCTTGCTGGGGTGTCTTCTATTTTAGGGGCGGTGAATTTCATCACTACAATTAT
TAATATGCGATGACAGGGAATAAAATTTGAGCGTCTTTCCTTGTTTGTCTGATCAGTAAA
AATTACGGCTATTTTACTTCTTTTATCTTTGCCTGTATTAGCGGGTGCTATTACTATGCT
TTTGACCGATCGAAATTTTAATACTGCATTTTTTGATCCAGCAGGAGGTGGTGATCCAAT
CTTATATCAACATTTGTTT
>radiatus
AACCAAGAAATTTAGTCATTCTTTTATGGTAAAAAGACAGTTATACAGG-TATGATTATG
AGACCTTTAGTAGAGGAGTAAAATCTATATACTGAAAGTTATACTAGAAGTAATTT-ATT
TATGTTGAATCTGTGACAGCTTTAAGGGAAACTGGGATTGGATACCCCATTATTTTTAGT
CGTAAATTTAGTT--TAAACTTACCGGAGTACTACGAATATTTAAAACTCAAAGGGCTTG
GCGGTGTTTTAGACCTCTCAGGGGAACCTGTCTCGTAATCGACAATCCACGTTAAACCTA
ACCCTTTTTGGTAATACAGCCTGTATACCGTCGTCGTCAGGTAACTTCTTAAAATATAGT
AGTTGGCTTGAGAATC--AG-ATTAATTAAAACGTCAGATCAAGGTGCAGCTAATAAGAG
GGTGAGGATGGGTTACAATTACATATTTGTAATTACGGTGCT-TGAAATAGAGTGC-TTA
AAGGAGGACTTGAAAGTAATTTTAATTATATAAATAAAATGAATAAGGCTCTGAAACGTG

CAGAATTTTAATGGTCGAACAGACCAACCCTTAAAGACTTCTGCATCTTTAGGATATTCT
GGTCCAACATCGAGGTCACAAACCTTTTTTTCGATATGGGCTCTTAAAAAAGATAATGCT
GTTATCCCTACGGTAACTAATTCCTTTGATCAAAAATTT-TGGATCGAT-ACAAGTAAGA
TTTA-AAAGGAGGCTCTAGTTACTCCTCGGTTGCCCCAACCAAAGT--ATTTAATAGTTT
T-TCTTTTA-CTTAATTT--GCATAGCCTACTAATTTCTCTCAAGCTCGATAGGGTCTTC
TTGTCTTTTAATAATATTTGGACTTTTTCATCCAAAAATAAAATTCTAAACAATCAAAAA
GAGACAGGTGTATTCTTGTCAAACCATTCATTCCAGCCTTCAATTATAAGGCAAATGATT
ATGCTACCTTTGCACGGTCAGAGTACCGCGGCCGTT-TAAAA-CACTGGGCAGGTCCGAC
TTCATATTTAAT-TTTAACATGACATTGTATATTTTATTTGGAATGTGGTCTGGTTTAGT
TGGAACGGCTTTAAGGCTATTAATTCGTGCAGAATTAGGGCAGCCGGGGGCTTTACTTGG
AGATGATCAACTGTATAATGTGATTGTAACTGCCCATGCTTTTGTTATAATTTTTTTCTT
AGTGATACCTATAATGATTGGGGGTTTTGGAAATTGGTTGGTACCGCTAATATTAGGGGC
TCCAGATATGGTGTTTCCTCGTTTGAATAATATAAGTTTTTGGCTTCTTCCTCCAGCACT
TCTACTTCTTCTATCATCAGCTGCAGTGGAAAGTGGGGTAGGTACGGGGTGGACTGTATA
TCCACCTTTAGCCGGAAATTTGGCTCATGCCGGTGGTTCTGTAGACCTGGCAATTTTCTC
TTTACATCTTGCTGGGGTGTCTTCTATTTTAGGGGCAGTGAACTTTATTACTACAATTAT
TAATATACGATGACAGGGAATAAAATTTGAGCGTCTTTCCTTGTTTGTTTGGTCAGTGAA
AATTACGGCTATTTTACTTCTCTTGTCTTTACCTGTATTAGCGGGTGCAATTACTATGCT
TCTAACTGATCGGAATTTTAATACTGCATTCTTTGATCCAGCAGGAGGTGGTGATCCCAT
CTTATATCAACATCTGTTT
>parius
AACCAAGAAATTTAGTCATTCTTTTATGGTAAAAAGACAGTTATACAAG-TATGATCATA
GGATCTTTAGTAAAGGAGTAAAATCTACATACTAAAAGTTATACTAGACGTGATTT-ATT
TATGTTGAGTCTGTGACAACTTTAAGGGAAACTGGGATTGGATACCCCATTATTTTTAGT
CGTAAATTTAGTT--TAAACTTACCAGAGTACTACGAATATTTAAAACTCAAAGGGCTTG
GCGGTGTTTTAGACCTCTCAGGGGAACCTGTCTCGTAATCGACAATCCACGTTAGACCTA
ACCCTTTGTGGTAATACAGCCTGTATACCGTCGTCGTCAGGTAACTTCTTAAAATATAGT
AGTTAGCTTGAGAATA--TG-ATTAATTAAAACGTCAGATCAAGGTGCAGCTAATAAAGG
GGTGAGGATGGGTTACAATTACATATTTGTAATTACGGCACT-TGAAATGAAGTGC-TTA
AAGGAGGACTTGAAAGTAATTTTGATTATATAAATAAAATGAATAAGGCTCTGAAACGTG
CAGAATTTTAATGGTCGAACAGACCAACCCTTAAAGACTTCTGCATCTTTAGGATATTCT
GGTCCAACATCGAGGTCACAAACCTTTTTTTCGATATGGGCTCTTAAAAAAGATAATGCT
GTTATCCCTACGGTAACTAATTCCTTTGATCAAAAATTT-TGGATCAAT-ACAAGTAAGA
TTCA-AAAGGAGGCTCTAGTTACTCCTCGGTTGCCCCAACCAAAGT--TTTTAATAGTTT
T-TCTTTTA-CTTAATTA--ACATAGCCTACTAATTTCTCTTAAGCTCGATAGGGTCTTC
TTGTCTTTTAATAATATTTGGACTTTTTCATCCAAAAATAAAATTCTAAACAATCAAAAA
GAGACAGGTGTATTCTTGTCAAACCATTCATTCCAGCCTTCAATTATAAGGCAAATGATT
ATGCTACCTTTGCACGGTCAGAGTACCGCGGCCGTT-TAAAA-CACTGGGCAGGTCCGAC
CTCATATTTAAT-TTTAACATGACATTATATATTTTATTTGGAATATGATCTGGTTTAGT
TGGAACGGCTTTAAGGTTGCTAATTCGTGCAGAATTAGGGCAACCTGGGGCCTTACTTGG
AGATGATCAACTTTATAATGTGATTGTAACTGCCCATGCTTTTGTTATAATTTTTTTCTT
AGTGATACCTATGATAATTGGGGGTTTTGGGAATTGATTAGTACCGTTGATATTAGGGGC
TCCAGATATAGTGTTTCCTCGTTTGAATAACATGAGTTTTTGACTTCTTCCTCCGGCACT
TTTACTTCTTTTGTCATCAGCTGCAGTAGAAAGTGGGGTAGGTACAGGATGAACTGTATA
TCCACCTTTGGCTGGGAATTTGGCCCATGCCGGTGGTTCTGTAGATCTAGCAATTTTTTC
TTTGCATCTTGCTGGGGTGTCTTCTATTTTAGGGGCGGTAAACTTTATTACTACAATTAT
TAACATACGATGACAGGGAATAAAGTTTGAACGCCTTTCCTTGTTTGTTTGATCGGTAAA
AATTACAGCTATCTTACTTCTTTTATCTTTGCCTGTATTAGCGGGTGCAATTACTATGCT

TCTGACCGATCGGAATTTTAATACTGCATTTTTTGATCCAGCAGGAGGTGGTGATCCTAT
CTTATATCAACATTTATTT
>lynceus
AACCAAGAAATTTAGTCATTCTTTTATGGTAAAAAGACAGTTATACAAA-TATAATCATA
AGATCTTTGGTAGAGGAGTAAAATCTATATACTAAAAGTTATACTAGAAGTGATTT-ACT
TATGTTGAATCTGTGACAGCTTTAAGGGAAACTGGGATTGGATACCCCATTATTTTTAGT
CGTAAATTTAGTT--TAAGCTTACCAGAGTACTACGAATGTTTAAAACTCAAAGGGCTTG
GCGGTGTTTTAGACCTCTCAGGGGAACCTGTCTCATAATCGACAATCCACGTTAGACCTA
ACCCTTTATGGTAATACAGCCTGTATACCGTCGTCGTCAGGTAACTTCTTAAAATATAGT
AGTTAGCTTGAGAATC--AG-ATTAATTAAAACGTCAGATCAAGGTGCAGCTAATAAAAG
GGTGAGGATGGGTTACAATTATATATTTATAATTATGGCACT-TGAAATGAAGTGC-TTA
AAGGAGGACTTGAAAGTAATTTTAATTATATAAATAAAATGAATAAGGCTCTGAAACGTG
CAGAATTTTAATGGTCGAACAGACCAACCCTCAAAGACTTCTGCATCTTTAGGACATTCT
GGTCCAACATCGAGGTCACAAACCTTTTTTTCGATATGGGCTCTTGAAAAAGATAATGCT
GTTATCCCTACGGTAACTAATTCCTTTGATCAAAATTCT-TGGATCAAC-ACAGGTAAGA
TTTA-AAAGGAGGCTCTTATTGCTCCTTGGTTGCCCCAACCAAAGT--GTTTAATAGCTT
T-TCTTTTA-CTTGATTG--ATATAGCCTATTAATTTTTCTGAAGCTCGATAGGGTCTTC
TTGTCTTTTAATAACATCTGGACTTTTTCATCCAAAAATAAAATTCTAAACAATCAAAAA
GAGACAGGTGTATTCTTGTCAAACCATTCATTCCAGCCTTCAATTATAAGGCAAATGATT
ATGCTACCTTTGCACGGTCAGAGTACCGCGGCCGTT-TAAAA-CACTGGGCAGGTCCGAC
TTCATATTTAAT-TTTGACATGACATTATATATTTTGTTTGGAATGTGATCTGGTTTAGT
TGGAACGGCTTTGAGATTACTGATTCGTGCAGAATTAGGGCAGCCGGGTGCTTTACTTGG
AGATGATCAATTATATAATGTTATCGTAACGGCTCATGCTTTTGTTATAATTTTTTTTTT
AGTGATGCCTATGATAATTGGGGGTTTTGGTAATTGATTGGTACCACTAATGTTAGGGGC
TCCAGATATAGTATTTCCTCGTTTGAATAATATAAGTTTTTGGCTTCTTCCTCCAGCACT
TTTACTTCTTCTATCATCAGCTGCGGTGGAAAGCGGGGTAGGCACAGGATGGACTGTATA
TCCTCCTTTAGCTGGGAATTTGGCTCACGCCGGTGGTTCTGTAGATCTGGCGATTTTTTC
TTTACATCTTGCTGGGGTTTCTTCTATTTTAGGGGCGGTAAATTTCATTACCACAATTAT
TAATATACGGTGACAGGGGATAAAATTTGAACGTCTTTCTTTGTTTGTATGATCGGTAAA
GATTACAGCTATTTTACTTCTTTTATCTTTGCCTGTATTAGCGGGTGCAATTACTATGCT
TTTAACTGATCGGAATTTTAATACTGCATTCTTTGATCCAGCAGGAGGTGGTGATCCTAT
TTTATACCAGCATTTATTT
>pica
AACCAAGAAATTTAGTCATTCTTTTATGGTAAAAAGACAGTTATACAGA-TATAATCATT
AGATCTTGAGTAAAGGAGTAAAATCTATATACTGAAAGATATGCTAAGCATGGTTT-ACT
TATGTTGAAGCTGTGATAGCTTTAAGGGAAACTGGGATTGGATACCCCATTATTTTTAGT
CGTAAATTTAGTT--TAAGCTTACCAGAGTACTACGAATTTTTAAAACTCAAAGGGCTTG
GCGGTGTTTTAGACCTCTCAGGGGAACCTGTCTCATAATCGACAATCCACGTTAAACCTA
ACCTTTTATTGTAATGCAGCCTGTATACCGTCGTCGCCAGGTAACTTCTTAAAATATAGT
AGTTAGCTTGAGAATT--TT-ATTAATTAGAACGTCAGATCAAGGTGCAGCTAATGAAAG
GGTGAGGATGGGTTACAATTATATATTCATAATGACGGCACT-TGAAATATAAAGTGTTA
AAGGAGGACTTGAAAGTAATTTTAATTATATAAATAAAGTGAATAAGGCTCTGAAACGTG
CAGAATTTTAATGGTCGAACAGACCAACCCTTAAAGGCTTCTGCACCTTTAGGACATTCT
GGTCCAACATCGAGGTCACAAACCTTTTTTTCGATATGGGCTCTTGAAAAAGATAATGCT
GTTATCCCTACGGTAACTAATTCCTTTAATCAAAAATCT-TGGATCAAC-ACAAGTAAAG
TTTA-AAAGGAGGCTCGAATTGCTCCTCGGTTGCCCCAACCAAAGT--ATTTAATAGCTT
T-CCTTTTATCATAATTG--GTAAAGCCTATTAATTTTTCTAAAGCTCGATAGGGTCTTC
TTGTCTTTTAATACTATCTGGACTTTTTCATCCAAAAATAAAATTCTAAATAATCAAAAA

GAGACAGGTGTATTCTTGTCAAACCATTCATTCCAGCCTTCAATTATAAGGCAAATGATT
ATGCTACCTTTGCACGGTCAGAGTACCGCGGCCGTT-TAAAA-CACTGGGCAGGTCCGAC
TTCGTATTTAAT-TTTGACACGACATTATATATTTTGTTTGGGATGTGATCAGGCTTGGT
TGGAACGGCTTTAAGACTATTAATTCGTGCAGAACTAGGACAGCCTGGTGCATTACTTGG
AGACGATCAGCTATATAATGTCATTGTAACGGCCCATGCTTTTGTTATGATTTTTTTCTT
AGTGATGCCCATAATAATTGGAGGTTTTGGAAACTGATTAGTGCCATTGATGTTAGGGGC
TCCAGACATAGTATTTCCTCGTTTGAATAATATAAGTTTTTGGCTTCTTCCTCCAGCGCT
GTTACTTCTTTTGTCATCAGCTGCAGTGGAAAGAGGGGTGGGCACAGGATGAACTGTATA
TCCTCCTTTAGCTGGAAATTTAGCTCATGCCGGTGGTTCTGTAGATCTGGCAATTTTTTC
TTTACATCTTGCTGGGGTCTCTTCTATTTTGGGGGCGGTAAATTTTATTACTACAATTAT
TAATATGCGATGGCAGGGAATAAAATTTGAGCGTCTTTCTTTGTTTGTTTGGTCGGTAAA
AATTACGGCTATTTTACTTCTTTTGTCTTTGCCTGTATTAGCGGGCGCAATTACTATGCT
TTTAACTGATCGAAATTTTAATACTGCATTTTTTGATCCAGCGGGAGGCGGGGATCCTAT
TTTATATCAACATTTATTT
>spectrum
AACCAAGAAATTTAGTCATTTGTTCATGGTAAAAAGACAGTTATACAAA-TATAATCATT
AGATCTTCAGTAAAGGAGTAAAATCTAAATACTGAAAGCTATTCTAGGCGTGATAT-ACT
TATGTTGAATCTGTGATAGCTTTAAGGGAAACTGGGATTGGATACCCCATTATTTTTAGT
CGTAAATTTAGTT--AATACTTACCAGAGTACTACGAATATTTAAAACTCAAAGGGCTTG
GCGGTGTTTTAGACCTCTCAGGGGAACCTGTCTCGTAATCGACAATCCACGTTAAACCTA
ACCCTTTATGGTAATACAGCCTGTATACCGTCGTCGTCAGGTAACTTCTTAAAATATAGT
AGTTAGCTTGAGAATT--TC-ATTAATTAGGACGTCAGATCAAGGTGCAGCTAATAAAAG
GGTGAGGATGGGTTACAATTACATATTTGTAATTACAGCACT-TGAAATTGAGTGT-CTA
AAGGAGGACTTGAAAGTAATCTTAATTATCTAAATAAAATGAATTGGGCTCTGAAACGTG
CAGAATTTTAATGGTCGAACAGACCAACCCTTAAAGACTTCTGCATCTTTAGGACATTCT
GGTCCAACATCGAGGTCACAAACCTTTTTTTCGATATGAACTCTTAAAAAAGATAATGCT
GTTATCCCTACGGTAACTAATTCCTTTGATCAAAACTTT-TGGATCAAC-ACAAGTAAGA
TTTA-AAAGGAGGTTTAAACTGCTCCTCGGTTGCCCCAACCAAAGT--GTTTAATAGTTT
TATTTTTTA-CTTAATTA--ATAAAACCTACTAATTTTTCTGAAGCTCGATAGGGTCTTC
TTGTCTTTTAATAAAATTTGGACTTTTTCATCCAAAAATAAAATTCTATAAAGTCAGAAA
GAGACAGGTGTATTCTTGTCAAACCGTTCATTCCAGCCTTCAATTATAAGGCAAATGATT
ATGCTACCTTTGCACGGTCAGAGTACCGCGGCCGTT-TAAAA-CACTGGGCAGGTCCGAC
TTCGTATTTAAG-CCTAACACGACATTATATATTTTATTTGGGATGTGATCCGGCTTAGT
TGGAACGGCCTTAAGATTGTTAATTCGTGCAGAGTTAGGGCAACCCGGTGCTTTACTCGG
AGATGATCAACTATATAATGTAATTGTAACAGCCCATGCTTTTGTTATAATTTTTTTCTT
AGTGATACCCATGATAATTGGGGGTTTTGGAAATTGGTTGGTGCCATTAATGTTAGGAGC
TCCAGACATAGTATTTCCTCGCTTGAATAATATGAGTTTTTGACTCCTTCCTCCAGCGCT
TTTACTTCTTTTGTCATCAGCCGCAGTAGAAAGTGGAGTAGGTACAGGGTGAACTGTATA
TCCACCTTTAGCCGGAAATTTAGCTCATGCCGGCGGTTCTGTAGATCTGGCAATTTTTTC
TTTACATCTTGCTGGTGTGTCTTCTATTTTAGGGGCAGTAAACTTCATTACCACAATTAT
TAACATACGATGACAGGGAATAAAATTTGAACGTCTTTCTTTGTTTGTTTGATCAGTAAA
AATTACAGCTATTTTACTTCTTTTATCATTACCTGTATTGGCGGGTGCAATTACTATGCT
TCTAACTGATCGAAATTTTAATACTGCGTTTTTTGACCCAGCAGGAGGTGGTGATCCTGT
TTTATATCAACATTTGTTT
>cervus
AACCAAGAAATTTAGTCATTCTTTCATGGTAAAAAGATAGTTAGGCAAA-TAAAACTATT
AGATCTTTAGTAAAGGAGTAAAATCTAAATACTAAAAGTTATACCAAATGTAGTTT-ATT
TACGTTGAATCTGTGACAGCTTTAAGGGAAACTGGGATTGGATACCCCATTATTTTTAGT

TATAAATTTAGTT--TATGCTTACCGGAGTACTACGAATGTTTAAAACTCAAAGGGCTTG
GCGGTGTTTTAGACCTCTCAGGGGAACCTGTCTCGTAATCGACAATCCACGTTAAACCTA
ACCTTTTGTAGTAATACAGCTTGTATACCGTCGTCGCCAGGTAACTTCTTAAAATATAGT
AGTTAGCGTGATAATT--TT--TAAATTAAAACGTCAGATCAAGGTGCAGCTAATAAGAA
GGTGAGGATGGGTTACAATTACATATTTGTAAATACGGCACT-TGAAATTGAGGGTTTTA
AAGGAGGACTTGAAAGTAATTTTGATTATATAAGCAAAATGAATACGGCTCTGAAACGTG
CAGAATTTTAATGGTCGAACAGACCAACCCTTAAAGACTTCTGCATCTTTAGGACATTCT
GGTCCAACATCGAGGTCACAAACCTTTTTTTCGATATGGGCTCTTAAAAAAGATAATGCT
GTTATCCCTACGGTAACTAATTCCTTTGATCAAAAATTT-TGGATCAAC-ACAAGTAGGA
TTTA-AAAGGAGGTTCTATATGCTCCTCGGTTGCCCCAACCAAAGT--ACTTAATAGTTT
TGTCTTCTA-CTTAATTG--AT-AGATCTATTAATTTTTCTGAAGCTCGATAGGGTCTTC
TTGTCTTTTACTAAAATTTGGACTTTTTCATCCAAAAATAAAATTCTAAATAATCTAAAA
GAGACAGCTGTATTCTTGTCAAACCATTCATTCCAGCCTTCAATTATAAGGCAAATGATT
ATGCTACCTTTGCACGGTCAGAGTACCGCGGCCGTT-TAAAA-CACTGGGCAGGTCCGAC
TTCGCATCTAGA-GCCTACGCGACATTATATATTTTATTTGGAATATGATCAGGACTGGT
GGGAACTGCCTTAAGACTTTTGATTCGTGCGGAGTTGGGGCAACCTGGTGCCCTACTTGG
GGACGATCAGTTATATAATGTTATTGTAACAGCCCATGCTTTTGTTATAATTTTTTTCTT
AGTGATGCCTATAATGATTGGGGGGTTTGGAAATTGATTGGTACCGTTGATGTTGGGGGC
TCCAGATATGGTATTTCCTCGTTTAAATAACATAAGTTTCTGACTTCTTCCTCCTGCACT
TTTACTTCTCCTGTCTTCGGCCGCGGTGGAAAGCGGAGTGGGAACAGGGTGAACTGTATA
TCCCCCTTTGGCAGGAAATCTGGCTCATGCTGGTGGTTCTGTAGATCTGGCAATTTTTTC
TCTACATCTTGCTGGTGTATCTTCTATTTTGGGAGCGGTAAATTTTATTACTACAATTAT
TAACATACGATGACAGGGAATGAAATTTGAACGTCTTTCACTTTTTGTGTGATCAGTAAA
AATTACAGCTATTTTACTTCTTCTGTCTTTGCCTGTTTTAGCGGGTGCTATTACTATACT
TTTAACTGGTCGAAATTTTAATACTGCTTTCTTTGATCCAGCAGGAGGCGGTGATCCTAT
TTTGTATCAACACTTGTTT
>dusavelli
AACCAAGAAATTTAGTCATTCTCTCATGGTAAAAAGACAGTTAGGCAAA-CAAGATTATT
AGACCTTTAGTAAAGGAGTAAAATCTAAATACTAAAAGTTATACCGGGTGTAGTCT-ACC
TATGTTGAATCTGTGACAGCTTTAAGGGAAACTGGGATTGGATACCCCATTATTTTTAGT
TATAAATTTAGTT--TATGCTTACCAGAGTACTACGAATGTTTAAAACTCAAAGGGCTTG
GCGGTGTTTTAGACCTCTCAGGGGAACCTGTCTCGTAATCGACAATCCACGTTAAACCTA
ACCTTTTGTAGTAGCACAGCCTGTATACCGTCGTCGTCAGGTAACTTCTTAAAATATAGT
AGTTAGCATGAGAATT--TT-ATAAATTAAAACGTCAGATCAAGGTGCAGCTAATAAAAA
GGTGAGGATGGGTTACAATTACATATTTGTAAATACGGCACT-TGAAATGAAGAGTTTCA
AAGGAGGACTTGAAAGTAATTTTGATTATATAGACAAAATGAATACGGCTCTGAAACGTG
CAGAATTTTAATGGTCGAACAGACCAACCCTTAAAGACTTCTGCATCTTTAGGATATTCT
GGTCCAACATCGAGGTCACAAACCTTTTTTTCGATATGGGCTCTTGAAAAAGATAATGCT
GTTATCCCTACGGTAACTAATTCCTTTGATCAAAATTTC-TGGATCGAC-ACAAGTAGGA
TTTA-AAAGGAAGCTTTATATGTTCCTCGGTTGCCCCAACCAAAGT--ACTTAATAGTTT
TGTCTTTTA-TCTAATTG--AT-AAGTCTATTAATTTTTCTGAAGCTCGATAGGGTCTTC
TTGTCTTTTACTAGAATTTGGACTTTTTCATCCAAAGATAAAATTCTAAATAATCTTAAA
GAGACAGCTGTATTCTTGTCAAACCATTCATTCCAGCCTTCAATTATAAGGCAAATGATT
ATGCTACCTTTGCACGGTCAGAGTACCGCGGCCGTT-TAAAA-CACTGGGCAGGTCCGAC
TTCGTATTTAGA-ACCTACACGACATTATATATCTTATTCGGAATATGATCAGGACTAGT
GGGAACTGCCCTAAGACTCTTAATTCGTGCGGAGTTGGGACAACCTGGTGCTTTACTCGG
AGATGATCAGCTATATAATGTAATTGTGACAGCCCATGCTTTTGTTATAATTTTTTTCTT
AGTAATGCCTATAATGATTGGGGGATTTGGAAACTGGTTGGTACCTTTAATACTAGGGGC

CCCAGATATGGTGTTTCCTCGCTTAAATAACATAAGTTTCTGACTTCTTCCTCCTGCGCT
TTTACTTCTTCTATCCTCGGCTGCAGTAGAGAGTGGGGTAGGAACAGGATGAACTGTGTA
TCCTCCTTTGGCAGGAAATTTAGCTCATGCTGGTGGTTCTGTAGATCTGGCAATTTTTTC
CTTACATCTTGCTGGTGTATCTTCTATTTTGGGGGCGGTAAATTTTATTACTACAATTAT
CAATATACGATGACAAGGAATGAAATTTGAACGGCTTTCGCTTTTTGTATGGTCGGTAAA
AATTACGGCCATTTTACTTCTTTTATCTTTACCTGTTTTGGCTGGAGCCATTACTATGCT
TTTAACTGATCGAAATTTTAATACTGCTTTCTTTGACCCAGCAGGAGGTGGTGATCCTAT
TTTATATCAGCACTTGTTT
>auricomus
AACCAAGAAATTTAGTCATTCTTTTATGGTAAAAAGACAGTTAGGCAGA-AAGAATCATT
AGACCCTTAGTATAGGAGTAAAATCTAGATACTAAGAGTTATACCGGGTGTGATTT-ATT
AGTGTTGAATCTGTGATAGCTTTAAGGGAAACTGGGATTGGATACCCCATTATTTTTAGT
TGTAAATTTAGCT--TAGGCTTACCAGAGTACTACGAATGTTTAAAACTCAAAGGGCTTG
GCGGTGTTTTAGACCTCTCAGGGGAACCTGTCTCATAATCGACAATCCGCGTTAAACCTA
ACCTTTTATAGCAACACAGCCTGTATACCGTCGTCGTCAGGTAACTTCTTAAAATATAGT
AGTTAGCTCGAGAATC--AG-GTTAATTAAAACGTCAGATCAAGGTGCAGCTAATAAAAA
GGTGAGGATGGGTTACAATTACAAATTTGTAAATACGGTACT-TGAAATTAAGTATCCTA
AAGGAGGACTTGAAAGTAATTCTAATTATATAAATAGGATGAATAGGGCTCTGAAACGTG
CNNNNNNNNNNNNGGTCGAACAGACCAACCCTCAAAGACTTCTGCATCTTTAGGACATTCT
GGTCCAACATCGAGGTCACAAACCTTTTTTTCGATATGGGCTCTTGAAAAAGATAATGCT
GTTATCCCTACGGTAACTAATTCCTTTGATCAAAAAGTT-TGGATCAAC-ACAAGCAAGA
TTTG-AGAGGAGGCTTTGTTTACTCCTTGGTTGCCCCAACCAAAGT--ATTTAATAGTTT
T-CTTTTTA-CCCGATTA--GTAAAGTCTATTAATTTCTCTAAAGCTCGATAGGGTCTTC
TTGTCTTTTAATCACATCTGGACTTTTTCATCCAAAGATAAAATTCTAAGCAATCTAAAA
GAGACAGGTGTATTCTTGTCAAACCATTCATTCCAGCCTTCAATTATAAGGCAAATGATT
ATGCTACCTTTGCACGGTCAGAGTACCGCGGCCGTT-TAAAA-CACTGGGCAGGTCCGAC
TTCGTATTTAAT-ATTGACACGACATTGTATATTTTATTTGGTATATGATCTGGGTTGGT
CGGAACGGCTCTGAGATTGTTAATTCGTGCAGAGCTAGGACAACCAGGTGCCTTGCTTGG
AGATGATCAATTATATAATGTGATTGTAACAGCTCATGCTTTTGTTATAATTTTTTTCTT
AGTAATGCCCATGATAATTGGGGGTTTTGGGAACTGACTGGTGCCGCTAATGTTGGGAGC
TCCAGATATAGTATTTCCGCGTTTAAATAATATGAGTTTCTGGCTTTTGCCCCCTGCACT
TTTACTTCTTCTATCATCAGCAGCCGTGGAAAGAGGGGTAGGGACAGGATGAACTGTATA
TCCACCTCTAGCTGGAAACTTAGCGCATGCCGGCGGGTCTGTAGATCTAGCAATTTTTTC
TTTGCATCTTGCTGGGGTTTCTTCCATTTTAGGGGCTGTAAATTTTATTACTACAATTAT
TAATATACGATGACAAGGAATGAAGTTTGAGCGTCTTTCTTTGTTCGTATGATCTGTAAA
AATTACAGCTATTTTACTTCTTTTATCTTTACCTGTGCTGGCCGGGGCAATTACTATGCT
CCTGACTGATCGAAATTTTAATACTGCCTTTTTTGACCCCGCAGGAGGCGGTGATCCTAT
TTTGTATCAGCACTTGTTT
>retifer
NNNNNNNNNNNNNNNNNNNNNNNNNNTATGGT-AAAAGACAGTTAGGCAAA-AAGGATCATG
AGACCCTTAGTATAGGAGTAAAATCTAAATACTAAGAGTTATACCAGATATGATTT-ATT
AGTGCTGAAGCTGTGATAGCTTTAAGGGAAACTGGGATTGGATACCCCATTATTTTTAGT
TGTAAATTTAGTT--TAGACTTACCAGAGTACTACGAATGTTTAAAACTCAAAGGGCTTG
GCGGTGTTTTAGACCTCTCAGGGGAACCTGTCTCATAATCGACAATCCACGTTAGACCTA
ACCTTCTATAGCAATACAGCCTGTATACCGTCGTCGTCAGGTAACTTCTTAAAATACAGT
AGTTAGCCCGAGAATC--AG-ATTAGTTAAAACGTCAGATCAAGGTGCAGCTAATAAAAA
GGTGAGGATGGGTTACAATTACAAATTTGTAAATACGGTACT-TGAAATTAAGTATCTTA
AAGGAGGACTTGAAAGTAATTCTAATTATATAGATAGGATGAATAGGGCTCTGAAACGTG

NNNNATTTTAATGGTCGAACAGACCAACCCTCAAAGACTTCTGCATCTTTAGGACATTCT
GGTCCAACATCGAGGTCACAAACCTTTTTTTCGATATGGGCTCTTAAAAAAGATAATGCT
GTTATCCCTACGGTAACTAATTCCTTTGATCAAAAAGCT-TGGATCAAC-ACAAGCAAGA
TTTA-AAAGGAGGCTTTATCTGCTCCTCGGTTGCCCCAACCAAAGT--ATTTAATAGCTT
T-CCTTTTA-CTTAATTA--GTGAAGTCTATTAACTTCTCTAAAGCTCGATAGGGTCTTC
TTGTCTTTTAATTGCATCTGGACTTTTTCATCCAAAAATAAAATTCTAAGCAATCTAAAA
GAGACAGGTGTATTCTTGTCAAACCATTCATTCCAGCCTTCAATTATAAGGCAAATGATT
ATGCTACCTTTGCACGGTCAGAGTACCGCGGCCGTT-TAAAA-CACTGGGCAGGTCCGAC
TTCGTATNNNNN-NNNNNNNNGACATTGTATATTTTATTTGGTATATGATCCGGGTTGGT
TGGAACGGCCCTGAGGTTACTAATTCGTGCGGAGTTGGGACAGCCAGGTGCCTTGCTTGG
AGATGATCAGCTGTATAATGTGATTGTAACGGCCCATGCTTTTGTTATGATTTTTTTCTT
GGTAATGCCCATGATGATTGGGGGATTTGGAAACTGACTGGTGCCGTTAATGTTAGGGGC
CCCAGACATGGTATTTCCGCGCCTAAATAATATAAGTTTCTGGCTTTTGCCTCCTGCACT
TTTACTTCTCTTATCATCAGCAGCTGTGGAAAGAGGGGTAGGAACGGGATGAACTGTATA
TCCGCCTTTAGCTGGGAATTTAGCGCATGCCGGTGGGTCTGTAGATCTAGCAATTTTCTC
TCTACACCTTGCTGGGGTTTCTTCTATTTTAGGGGCTGTAAATTTTATTACTACAATTAT
TAACATACGATGACAAGGAATGAAGTTTGAACGTCTTTCTTTGTTTGTATGATCAGTAAA
AATTACAGCCATTTTGCTTCTTCTATCTTTACCTGTATTAGCTGGGGCTATTACTATGCT
CCTGACTGATCGAAATTTTAATACCGCCTTTTTTGACCCTGCAGGAGGTGGGGATCCTAT
TTTATATCAACACTTATTT
>gloriamaris
AACCAAGAAATTTAGTCATTCTTTTATGGTAAAAAGACAGTTAAGCAGA-GAGGATCATT
AGATCCTTAGTACAGGAGTAAAATCTAAATACTAAGAGTTATACCTGACATGGTTT-ATT
AGTGCTGAATCTGTGATAGCTTTAAGGGAAACTGGGATTGGATACCCCATTATTTTTAGT
TGTAAATTTAGTT--AAGACTTACCGGAGTACTACGAATGTTTAAAACTCAAAGGGCTTG
GCGGTGTTTTAGACCTCTCAGGGGAACCTGTCTCATAATCGACAATCCACGTTAAACCTA
ACCTTTTATAGCGATACAGCTTGTATACCGTCGTCGTCAGGTAACTTCTTAAAATATAGT
AGTTAGCCCGAGAATC--AA-GTTAATTAAAACGTCAGATCAAGGTGCAGCCAATAAAAA
GGTGAGGATGGGTTACAATTATATATTTGTAAATACGGTACT-TGAAACAAAGTATCTTA
AAGGAGGACTTGAAAGTAATTCTAATTATATAAACAGGATGAATAAGGCTCTGAAACGTG
CAGAATTTTAATGGTCGAACAGACCAACCCTTAAAGACTTCTGCATCTTTAGGACATTCT
GGTCCAACATCGAGGTCACAAACCTTTTTTTCGATATGGGCTCTTGAAAAAGATAATGCT
GTTATCCCTACGGTAACTAATTCCTTTGATCAAAAGTTT-TGGATCAAC-ACAAGCAAGA
TTTA-AAAGGAGGCTTTGCTTACTCCCCGGTTGCCCCAACCAAAGT--ATTTAATAGTTT
T-CTTTTTA-TTTAATTA--GTAAACTCTATTAATTTCTCTAAAGCTCGATAGGGTCTTC
TTGTCTTTTAATTATATCTGGACTTTTTCATCCAAAAATAAAATTCTAAGCAATCTAAAA
GAGACAGGTGTATTCTTGTCAAACCATTCATTCCAGCCTTCAATTATAAGGCAAATGATT
ATGCTACCTTTGCACGGTCAGAGTACCGCGGCCGTT-TAAAA-CACTGGGCAGGTCCGAC
TTCGTATTTAAC-ACTGACACGACATTGTATATTTTGTTTGGTATATGATCTGGATTAGT
TGGAACGGCCCTAAGGCTATTGATTCGTGCAGAGTTAGGACAACCAGGTGCCTTGCTTGG
GGACGATCAGCTATATAATGTGATTGTAACAGCTCATGCTTTTGTTATGATTTTTTTTTT
AGTTATGCCTATAATAATCGGGGGTTTTGGAAATTGACTGGTACCGCTGATGTTGGGAGC
TCCAGATATAGTATTCCCACGTTTAAATAATATGAGTTTTTGGCTTTTACCTCCCGCACT
TTTACTTCTTCTGTCATCGGCAGCTGTGGAAAGAGGGGTAGGAACAGGATGAACTGTATA
TCCACCTTTGGCTGGAAATTTGGCACATGCTGGCGGATCTGTAGATCTAGCAATTTTCTC
TTTACATCTTGCTGGAGTTTCTTCTATTTTGGGAGCTGTAAATTTTATTACTACGATTAT
TAACATACGATGACAAGGAATGAAATTTGAACGTCTTTCATTATTTGTATGATCTGTAAA
AATCACAGCTATTTTACTTCTTTTATCTTTACCCGTGCTGGCTGGGGCTATTACTATGCT

TCTGACTGATCGAAATTTTAATACTGCTTTTTTTGATCCTGCAGGAGGTGGGGATCCTAT
TTTATATCAGCATTTATTT
>bengalensis
AACCAAGAAATTTAGTCATTCTTTTATGGTAAAAAGACAGTTAGGCAGA-AAGGACCCTT
AGACCCTTAGTACAGGAGTAAAATCTAAATACTAAGAGTTATACCTGACATGGTTT-ATT
AGTGCTGAATCTGTGATAGCTTTAAGGGAAACTGGGATTGGATACCCCATTATTTTTAGT
TGTAAATTTAGTT--AAGACTTACCGGAGTACTGCGAATGTTTAAAACTCAAAGGGCTTG
GCGGTGTTTTAGACCTCTCAGGGGAACCTGTCTCATAATCGACAATCCACGTTAAACCTA
ACCTTTTATAGCGATACAGTTTGTATACCGTCGTCGTCAGGTAACCTCTTAAAATATAGT
AGTTAGCCCGAGAATC--AA-GTTAATTAAAACGTCAGATCAAGGTGCAGCCAATAAAAA
GGTGAGGATGGGTTACAATTACACATTTGTAAATACGGTACT-TGAAACAAAGTATCTTA
AAGGAGGACTTGAAAGTAATTCTAATTATATAAATAGGATGAATAAGGCTCTGAAACGTG
CAGAATTTTAATGGTCGAACAGACCAACCCTTAAAGACTTCTGCATCTTTAGGACATTCT
GGTCCAACATCGAGGTCACAAACCTTTTTTTCGATATGGGCTCTTGAAAAAGATAATGCT
GTTATCCCTACGGTAACTAATTCCTTTGATCAAAAGCTT-TGGATCAAC-GCAAGCAAGA
TTTA-AAAGGAGGCTTTGCTTACTCCTCGGTTGCCCCAACCAAAGT--GTTTAATAGTTT
T-CTTTTTA-TTTAATTA--GTAAACTCTATTAATTTCTCTAAAGCTCGATAGGGTCTTC
TTGTCTTTTAATTATATCTGGACTTTTTCATCCAGAAATAAAATTCTAAGCAATCTAAAA
GAGACAGGTGTATTCTTGTCAAACCATTCATTCCAGCCTTCAATTATAAGGCAAATGATT
ATGCTACCTTTGCACGGTCAGAGTACCGCGGCCGTT-TAAAA-CACTGGGCAGGTCCGAC
TTCGTATTTAAC-ACTGACACGACATTGTATATTTTGTTTGGTATATGATCTGGATTAGT
TGGAACGGCCCTAAGGCTATTGATTCGTGCAGAGTTAGGACAACCGGGTGCCTTGCTTGG
GGATGATCAGCTATATAATGTGATTGTAACAGCCCATGCTTTTGTTATGATTTTTTTTTT
AGTTATGCCTATAATAATCGGGCGTTTTGGAAATTGATTGGTACCGCTGATGTTGGGAGC
TCCAGATATAGTATTCCCACGTTTAAATAATATGAGTTTTTGGCTTTTACCTCCCGCACT
TTTACTTCTTCTATCATCGGCAGCTGTGGAAAGAGGGGTAGGAACAGGATGAACTGTATA
TCCACCTTTGGCTGGAAATTTGGCACATGCTGGCGGATCTGTAGATCTGGCAATTTTTTC
TTTACATCCTGCTGGTGTTTCTTCTATTTTAGGAGCTGTAAATTTTATTACTACAATTAT
TAACATACGATGACAAGGAATGAAATTTGAACGCCTTTCATTATTTGTGTGATCTGTAAA
AATTACAGCTATTTTACTTCTTTTATCTTTACCAGTGCTGGCTGGGGCTATTACTATGCT
TCTAACCGATCGAAATTTTAATACTGCTTTTTTTGACCCTGCAGGAGGTGGGGATCCTAT
TTTATATCAGCATTTATTT
>victoriae
AACCAAGAAATTTAGTCATTCTTTTATGGTAAAAAGACAGTTAAGCAGA-GAGGATCATT
AGACCCTTAGTACAGGAGTAAAATCTAAATACTGAGAGTTATACCTGGTATGATTT-ATT
AGTGCTGAATCTGTGATAGCTTTAAGGGAAACTGGGATTGGATACCCCATTATTTTTAGT
TGTAAATTTAGTT--AAGGCTTACCGGAGTACTACGAATGTTTAAAACTCAAAGGGCTTG
GCGGTGTTTTAGACCTCTCAGGGGAACCTGTCTCATAATCGACAATCCACGTTAAACCTA
ACCTTTTATAGCGATACAGCTTGTATACCGTCGTCGTCAGGTAACTTCTTAAAATATAGT
AGTTAGCCCGAGAATT--AA-GTTAATTAAAACGTCAGATCAAGGTGCAGCTAATAAAAA
GGTGAGGATGGGTTACAATTATATATTTATAAATACGGTACT-TGAAATAAAGTATCTTA
AAGGAGGACTTGAAAGTAATTCTAATTATATAAATAGGATGAATAGGGCTCTGAAACGTG
CNNNATTTTAATGGTCGAACAGACCAACCCTTAAAGACTTCTGCATCTTTAGGACATTCT
GGTCCAACATCGAGGTCACAAACCTTTTTTTCGATATGAGCTCTTGAAAAAGATAATGCT
GTTATCCCTACGGTAACTAATTCCTTTAATCAAAATATT-TGGATCAAC-ACAAGCAAGA
TTTA-AAAGGAGGCTTTACTTACTCCTCGGTTGCCCCAACCAAAGT--GTTTAATAGTTT
T-CTTTTTA-CCTAATTA--GTAAACTCTATTAATTTCTCTAAAGCTCGATAGGGTCTTC
TTGTCTTTTAATCATATCTGGACTTTTTCATCCAAAAATAAAATTCTAAGCAATCTAAAA

GAGACAGCTGCATTCTTGTCAAACCATTCATTCCAGCCTTCAATTATAAGGCAAATGATT
ATGCTACCTTTGCACGGTCAGAGTACCGCGGCCGTT-TAAAA-CACTGGGCAGGTCCGAC
TTCGTATNNNNN-NNNNNNNNNNNNNNNNNNNNATTTTGTTTGGTATATGATCTGGATTAGT
TGGAACGGCCCTAAGGYTATTGATTCGTGCAGAGTTAGGACAACCAGGTGCCTTGCTTGG
GGACGATCAGCTATATAATGTRATTGTAACAGCCCATGCTTTTGTTATGATTTTTTTCTT
AGTTATGCCTATAATAATTGGGGGTTTTGGAAACTGGCTGGTACCCCTCATGTTGGGAGC
TCCAGATATAGTATTCCCACGTTTAAATAATATAAGTTTTTGGCTTTTACCTCCAGCACT
TTTACTTCTTCTATCGTCGGCAGCTGTGGAAAGGGGGGTGGGAACAGGATGAACTGTATA
TCCGCCTTTGGCTGGAAATTTAGCACATGCTGGTGGATCTGTAGATCTGGCAATTTTTTC
TTTACATCTTGCTGGGGCTTCTTCTATTTTGGGAGCTGTAAATTTTATTACTACGATTAT
TAACATACGATGACAAGGAATGAAATTTGAACGTCTTTCATTGTTTGTATGATCTGTAAA
AATTACGGCTATTTTGCTTCTTTTATCTTTACCTGTGCTGGCTGGGGCCATTACTATGCT
TCTAACTGATCGAAATTTTAATACTGCTTTCTTTGATCCTGCAGGAGGCGGGGATCCTAT
TTTATATCAGCATTTATTT
>aureus
AACCAAGAAATTTAGTCATTCTTTTACGGTAAAAAGACAGTTAAGCAGA-AAGGATCATT
AGACCCTTAATATAGGAGTAAAATCCAAATATTAAGAGTTATATCAGATGTGGTTT-ATT
AATGCTGAGTCTGTGATAGCTTTAAGGGAAACTGGGATTGGATACCCCATTATTTTTAGT
TGTAAACTTAGTT--TAGGCTTACCGGAGTACTACGAATGTTTAAAACTCAAAGGGCTTG
GCGGTGTTTTAGACCTCTCAGGGGAACCTGTCTCATAATCGACAATCCGCGTTAAACCTG
ACCTTTTATAGCAATACAGCCTGTATACCGTCGTCGCCAGGTAACTTCTTAAAATATAGT
AGTTAGCCCGAGAATC--AA-GTTAATTAAAACGTCAGATCAAGGTGCAGCTAATAAAAA
GGTGAGGATGGGTTACAATTATATATTTGTAAATACGGCACT-TGAAACAAAGTGTCTTA
AAGGAGGACTTGAAAGTAATTCTAATTATATAAATAGGATGAATAAGGCTCTGAAACGTG
CAGAATTTTAATGGTCGAACAGACCAACCCTTAAAGACTTCTGCATCTTTAGGACATTCT
GGTCCAACATCGAGGTCACAAACCTTTTTTTCGATATGGGCTCTTGAAAAAGATAATGCT
GTTATCCCTACGGTAACTAATTCCTTTGATCAAAAGCTT-TGGATCAAC-ACAAGTAAGA
CTTA-AAAGGAGGCTTTATTTACTCCTCGGTTGCCCCAACCAAAAT--ATTTAATAGTTT
T-CTTTTTA-CTTAATTA--ATAAATTCTATTAATTTCTCTAAAGCTCGATAGGGTCTTC
TTGTCTTTTAATTATATCTGGACTTTTTCATCCAAAAATAAAATTCTAAGTAATCTAAAA
GAGACAGGTGTATTCTTGTCAAACCATTCATTCCAGCCTTCAATTATAAGGCAAATGATT
ATGCTACCTTTGCACGGTCAGAGTACCGCGGCCGTT-TAAAA-CACTGGGCAGGTCCGAC
TTCGTATTTAAT-ATTGACACGACATTGTATATTTTGTTTGGTATATGATCTGGATTAGT
TGGAACAGCCCTAAGGTTACTGATTCGTGCGGAGTTAGGGCAACCAGGTGCCTTGCTTGG
GGATGATCAGCTATATAATGTGATTGTAACGGCCCATGCTTTTGTTATGATCTTTTTCTT
AGTTATACCTATGATAATTGGGGGCTTTGGAAACTGGCTAGTACCGTTGATGTTAGGGGC
TCCGGATATAGTATTCCCGCGCTTAAATAACATGAGTTTTTGGCTTTTACCCCCTGCACT
TTTACTTCTTTTATCATCGGCAGCTGTGGAAAGGGGGGTAGGAACAGGATGAACTGTATA
TCCGCCCTTAGCTGGGAATTTAGCGCATGCTGGCGGATCTGTAGATCTAGCGATTTTTTC
TTTGCATCTTGCTGGAGTTTCTTCTATTTTGGGAGCTGTAAATTTTATTACTACGATTAT
TAATATACGATGACAAGGAATGAAATTTGAACGCCTTTCATTGTTTGTATGATCTGTAAA
AATTACAGCTATTTTGTTACTTTTATCTTTACCTGTGTTGGCTGGGGCTATTACTATGCT
TCTAACTGATCGGAATTTTAACACTGCTTTCTTTGACCCTGCAGGAGGTGGAGATCCTAT
TTTATATCAGCATTTATTT
>legatus
AACCAAGAAATTTAGTCATTCTTTTACGGTAAAAAGACGGTTAAGCAGA-GATGATCATT
AGACCCTTAGTATAGGAGTAAAATCTAAATACTAAGAGTTATACCAGCTGTGATTT-ATT
AGTGCTGAATCTGTGATAGCTTTAAGGGAAACTGGGATTGGATACCCCATTATTTTTAGT

TGTAAATTTAGTT--TAGACTTACCGGAGTACTACGAATGTTTAAAACTCAAAGGGCTTG
GCGGTGTTTTAGACCTCTCAGGGGAACCTGTCTCGTAATCGACAATCCACGTTAAACCTA
ACCTTTTATAGCAGTACAGCCTGTATACCGTCGTCGTCAGGTAACTTCTTAAAATATAGT
AGTTAGCTCGAGAATC--AA-GTTAATTAAAACGTCAGATCAAGGTGCAGCTAATAAAAA
GGTGAGGATGGGTTACAATTATATATTTGTAAGTACGGTACT-TGAAATGAAGTATCTTA
AAGGAGGACTTGAAAGTAATTTTAATTATATAAATAGAATGAATAGGGCTCTGAAACGTG
CAGAATTTTAATGGTCGAACAGACCAACCCTTAAAGACTTCTGCATCTTTAGGATATTCT
GGTCCAACATCGAGGTCACAAACCTTTTTTTCGATATGGGCTCTTGAAAAAGATAATGCT
GTTATCCCTACGGTAACTAATTCCTTTGATCAAAAACTT-TGGATCAAC-ACAAGTAAGA
TTTA-AAAGGAGGCTTTATTTACTCCTCGGTTGCCCCAACCAAAGT--ATTTAATAGTTT
T-CTTTTTA-TTTGATTA--GTAAAGTCTATTAACTTCTCTAAAGCTCGATAGGGTCTTC
TTGTCTTTTAATCATATCTGGACTTTTTCATCCAGAAATAAAATTCTAAGTAATCTAAAA
GAGACAGGTGTATTCTTGTCAAACCATTCATTCCAGCCTTCAATTATAAGGCAAATGATT
ATGCTACCTTTGCACGGTCAGAGTACCGCGGCCGTT-TAAAA-CACTGGGCAGGTCCGAC
TTCGTATTTAAT-ATTGACACGACATTATATATTTTATTTGGTATATGGTCTGGCTTGGT
TGGAACGGCCCTAAGTCTATTGATTCGTGCAGAATTAGGACAACCAGGTGCCTTACTTGG
GGATGATCAACTATATAACGTGATTGTAACAGCCCATGCTTTTGTTATGACTTTTTTCTT
AGTAATACCTATGATAATTGGGGGTTTTGGAAACTGGTTGGTGCCTTTAATGTTGGGGGC
TCCAGATATGGTGTTTCCTCGCTTAAATAACATAAGTTTTTGGCTTTTGCCTCCTGCGCT
TTTACTCCTTCTATCATCAGCAGCCGTAGAGAGGGGGGTAGGAACAGGATGAACTGTATA
TCCACCTTTAGCTGGAAATCTAGCGCATGCCGGTGGATCTGTAGATTTGGCAATTTTTTC
TTTACACCTTGCTGGGGTTTCTTCTATTTTAGGGGCTGTAAACTTTATTACCACGATTAT
TAATATGCGATGACAAGGAATGAAATTTGAACGTCTTTCATTGTTTGTGTGATCTGTAAA
AATTACAGCTATTTTGCTTCTTCTGTCTTTACCTGTATTGGCTGGAGCCATTACTATGCT
TCTAACTGATCGAAATTTCAATACTGCCTTTTTTGATCCTGCAGGAGGTGGTGATCCTAT
TTTATATCAACATTTATTT
>aulicus
AACCAAGAAATTTAGTCATTCTTTTACGGTAAAAAGACAGTTAGGCAAA-AATAATCATT
AGACCTTTGGTATAGGAGTAAAATCTAAATACTAAAAGTTATATTAGTTGTGGTTT-ATT
AGTGCTGAATCTGTGATAGCTTTAAGGGAAACTGGGATTGGATACCCCGTTATTTTTAGT
TGTAAATTTAGTT--TAAGCTTACCAGAGTACTACCAATGTTTAAAACTCAAAGGGCTTG
GCGGTGTTTTAGACCTCTCAGGGGAACCTGTCTCGTAATCGACAATCCACGTTAAACCTA
ACCTTTTATGGCGATACAGCCTGTATACCGTCGTCGTCAGGTAACTTCTTAAAATATAGC
AGTTAGCTCGAGAACT--TA-ATTAATTAGAACGTCAGATCAAGGTGCAGCTTATAAAGA
GGTGAGGATGGGTTACAATTACATATTTGTAAATACGGTACT-TGAAACATAGTATCATA
AAGGAGGACTTGAAAGTAATTCTAGTTATATAAATAGAATGAATATGGCTCTGAAACGTG
CAGAATTTTAATGGTCGAACAGACCAACCCTCAAAGACTTCTGCATCTTTAGGACATTCT
GGTCCAACATCGAGGTCACAAACCTTTTTTTCGATATGGGCTCTTGAAAAAGATAATGCT
GTTATCCCTACGGTAACTAATTCCTTTGATCAAAAATTT-TGGATCAAT-ACAAGCAAGA
TTTA-AAAGGAGGCTTTACTTACTCCTTGGTTGCCCCAACCAAAGT--TTTTAATAGCTT
T-TCTTTTA-ATTAATTG--ATAAACTCTATTAATTCTTCTAAAGCTCGATAGGGTCTTC
TTGTCTTTTAATCGTATCTGGACTTTTTCATCCAAAAATAAAATTCTAGCTAATCTAAAA
GAGACAGGTGTATTCTTGTCAAACCATTCATTCCAGCCTTCAATTATAAGGCAAATGATT
ATGCTACCTTTGCACGGTCAGAGTACCGCGGCCGTT-TAAAA-CACTGGGCAGGTCCGAC
TTCGTATTTAAT-ACCGACACGACACTGTATATTTTATTTGGTATATGATCTGGGTTAGT
TGGAACGGCTCTAAGATTATTGATTCGTGCAGAGTTAGGACAACCGGGTGCTTTACTTGG
TGATGATCAACTGTATAATGTAATTGTAACAGCCCACGCTTTTGTTATAATTTTTTTCTT
AGTGATGCCTATAATAATCGGAGGATTTGGAAATTGGCTGGTGCCATTGATATTGGGGGC

TCCAGACATAGTATTTCCGCGTTTAAATAACATAAGTTTTTGGCTTTTACCTCCTGCACT
TTTACTTCTTTTATCATCGGCTGCAGTAGAAAGTGGTGTTGGAACAGGATGGACTGTGTA
TCCACCTTTAGCAGGAAATTTAGCACATGCCGGGGGGTCTGTAGATCTAGCAATTTTTTC
TTTACACCTTGCTGGGGTTTCTTCTATTTTAGGAGCGGTAAATTTTATTACCACAATCAT
TAATATGCGATGACAAGGAATAAAATTTGAGCGTCTTTCATTATTTGTATGATCTGTAAA
AATTACAGCTATTCTACTTCTTCTATCTTTACCTGTCTTAGCTGGAGCTATTACTATGCT
TTTGACTGATCGAAATTTTAATACTGCTTTCTTTGATCCTGCAGGAGGCGGCGACCCTAT
TTTATATCAGCATTTATTT
>episcopatus
AACCAAGAGATTTAGTCATTCTTTTATGGTAAAAAGACAGTTAAGCAGA-AAGGATCACC
AGACCTTTAGTATAGGAGTAAAATCTAAATACTAAAAGTTATACCAGCTGTGGTTC-ATC
TGTGCTGAATCTGTGATAACTTTAAGGGAAACTGGGATTGGATACCCCATTATTTCTAGT
TGTAAATTTAGCT--TAAGCTTACCAGAGTACTACGAATGTTTAAAACTCAAAGGGCTTG
GCGGTGTTTTAGACCTCTCAGGGGAACCTGTCTCGTAATCGACAATCCACGTTAAACCTA
ACCTCTTATAGCGACACAGCCTGTATACCGTCGTCGTCAGGTAACTTCTTAAAATGTAGT
AGTTAGCTCGAGAATT--AG-ATTAATTAGAACGTCAGATCAAGGTGCAGCTTATAAAGA
GGTGAGGATGGGTTACAATTACATATTTGTAAATACGGTACT-TGAAATATAGTATCATA
AAGGAGGACTTGAAAGTAATTTTAGTTATATAAATAGAATGAATATGGCTCTGAAACGTG
CAGAATTTTAATGGTCGAACAGACCAACCCTTAAAGACTTCTGCATCTTTAGGATATTCT
GGTCCAACATCGAGGTCACAAACCTTTTTTTCGATATGGGCTCTTGAAAAAGATAATGCT
GTTATCCCTACGGTAACTAATTCCTTTGATCAAAAAATT-TGGATCAAT-ACAAGTAAGA
TTTA-AAAGGAGGCTTTATTTACTCCTTGGTTGCCCCAACCAAAGT--GTTTAATAGCTT
T-TCTTTTA-TCAAATCA--AGAAACTCTATTAATTCCTCTAAAGCTCGATAGGGTCTTC
TTGTCTTTTAATTATATCTGGACTTTTTCATCCAAAAATAAAATTCTAATCAATCTAAAA
GAGACAGGTGTATTCTTGTCAAACCATTCATTCCAGCCTTCAATTATAAGGCAAATGATT
ATGCTACCTTTGCACGGTCAGAGTACCGCGGCCGTT-TAAAA-CACTGGGCAGGTCCGAC
TTCGTATTTAGT-AATGACACGACATTGTATATTTTATTTGGTATATGATCTGGATTGGT
TGGAACAGCCCTAAGATTATTAATTCGTGCAGAGTTAGGGCAACCAGGTGCTTTGCTTGG
AGACGATCAACTATATAATGTAATTGTAACAGCGCATGCTTTTGTTATAATTTTTTTCTT
AGTGATGCCTATAATAATTGGAGGGTTTGGAAACTGGCTGGTGCCATTGATATTAGGAGC
TCCAGATATAGTATTTCCACGTTTAAATAACATAAGTTTTTGACTTTTACCTCCTGCACT
CTTACTTCTTTTGTCATCAGCTGCAGTAGAAAGTGGTGTTGGAACAGGATGAACTGTATA
TCCGCCTTTAGCAGGAAATTTAGCACATGCTGGCGGATCTGTAGATCTGGCAATTTTTTC
TTTACATCTTGCTGGGGTTTCATCTATTTTAGGAGCGGTAAATTTTATTACCACAATCAT
TAACATACGATGACAAGGAATAAAATTTGAACGTCTTTCACTATTTGTATGATCTGTGAA
GATTACAGCTATTCTACTTCTTCTATCTTTACCTGTCTTAGCTGGGGCTATTACTATACT
TTTAACTGATCGAAATTTTAATACTGCTTTCTTTGACCCTGCAGGCGGCGGTGACCCTAT
TTTGTACCAACATTTATTT
>crocatus
AACCAAGAGATTTAGTCATTCTTTTACGGTAAAAAGACAGTTAAGCAGA-AGAGATCATT
AGACCTTTAGTATAGGAGTAAAATCTAAATACTAAAAGTTATATCGGTTGTGGTTC-ATT
TGTGCTGAGTCTGTGATAGCTTTAAGGGAAACTGGGATTGGATACCCCATTATTTTTAGT
TGTAAATTTAGTT--TAAGCTTACCGGAGTACTACGAATGTTTAAAACTCAAAGGGCTTG
GCGGTGTTTTAGACCTCTCAGGGGAACCTGTCTCGTAATCGACAGTCCACGTTAAACCTA
ACCTTTTATAGCAATACAGCTTGTATACCGTCGTCGTCAGGTAACTTCTTAAAATATAGT
AGTTAGCTCGAGAATC--AA-ATTAATTAGGACGTCAGATCAAGGTGCAGCCAATAAAGA
GGTGAGGATGGGTTACAATTACATACTTGTAAATACGGTATT-TGAAACATAGTATCATA
AAGGAGGACTTGAAAGTAATTTTAGTTATATAAATAGAATGAATATGGCTCTGAAACGTG

CAGAATTTTAATGGTCGAACAGACCAACCCTTAAAGACTTCTGCATCTTTAGGATATTCT
GGTCCAACATCGAGGTCACAAACCTTTTTTTCGATATGGGCTCTTGAAAAAGATAATGCT
GTTATCCCTACGGTAACTAATTCCTTTGATCAAAAATTT-TGGATCAGC-ACAAGTAAGA
TTCA-AAAGGAGGCTTTACTTACTCCTCGGTTGCCCCAACCAAAGT--ATTTAATAGTCT
T-CCTTTTA-CCTAATTA--GTAAACCCTATTAATTTTCCTAAAGCTCGATAGGGTCTTC
TTGTCTTTTAATTGCATCTGGGCTTTTTCACCCAAAAATAAAATTCTAATAAATCTAAAA
GAGACGGCTGTATTCTTGTCAAACCGTTCATTCCAGCCTTCAATTATAAGGCAAATGATT
ATGCTACCTTTGCACGGTCAGAGTACCGCGGCCGTT-TAAAA-CACTGGGCAGGTCCGAC
TTCATATTTAAT-ACTGACACGACGTTGTATATTTTATTTGGTATATGATCTGGTTTAGT
CGGAACGGCTTTGAGATTGCTAATTCGTGCAGAACTAGGACAACCAGGTGCTTTACTTGG
TGACGATCAACTATACAATGTAATTGTAACAGCTCATGCTTTTGTCATAATTTTTTTCTT
AGTGATGCCTATAATAATCGGAGGCTTTGGAAACTGATTAGTACCATTAATGTTGGGGGC
TCCAGATATAGTATTTCCGCGGTTAAATAACATAAGTTTTTGGCTTTTGCCGCCTGCACT
TTTACTTCTTTTGTCGTCAGCTGCAGTGGAGAGGGGGGTAGGAACAGGATGAACTGTATA
TCCACCTTTGGCAGGAAATCTGGCGCATGCTGGTGGGTCTGTGGATCTAGCGATTTTTTC
TTTACATCTTGCTGGAGTTTCTTCTATTTTAGGAGCGGTGAACTTTATTACTACTATCAT
TAATATGCGATGGCAAGGAATAAAGTTTGAGCGTCTTTCGTTGTTTGTGTGATCTGTGAA
GATTACGGCTATTTTACTCCTTTTATCTTTGCCTGTCTTGGCTGGAGCCATTACTATGCT
TTTAACTGATCGAAATTTTAACACTGCTTTCTTTGATCCGGCGGGGGGTGGTGATCCTAT
TTTGTACCAGCATTTATTT
>pennaceus
AACCAAGAAATTTAGTCATTCTTTTACGGTAAAAAGACAGTTAAGCAAA-AAGGATCATT
AGACCTTTAGTATAGGAGTAAAATCTAAATACTAAAAGTTATATCAGTTATGGTTT-GTT
TGTGCTGAATCTGTGATAGCTTTAAGGGAAACTGGGATTGGATACCCCATTATTTTTAGT
TGTAAATTTAGTT--TAAGCTTACCAGAGTACTACGAATGTTTAAAACTCAAAGGGCTTG
GCGGTGTTTTAGACCTCTCAGGGGAACCTGTCTCGTAATCGACAGTCCGCGTTAAACCTA
ACCTTTTATAGCAATACAGCTTGTATACCGTCGTCGTCAGGTAACTTCTTAAAATATAGT
AGTTAGCTCGAGAATC--AA-GTTAATTAGGACGTCAGATCAAGGTGCAGCTAATAAAGA
GGTGAGGATGGGTTACAATTACATACTTGTAAATACGGTATT-TGAAATATAGTATCATA
AAGGAGGACTTGAAAGTAATTTTAGTTATATAAATAGGATGAATATGGCTCTGAAACGTG
CAGAATTTTAATGGTCGAACAGACCAACCCTTAAAGACTTCTGCATCTTTAGGATATTCT
GGTCCAACATCGAGGTCACAAACCTTTTTTTCGATATGGGCTCTTGAAAAAGATAATGCT
GTTATCCCTACGGTAACTAATTCCTTTGATCAAAAAATT-TGGATCAAT-ACAAGTAAGA
TCTA-AAAGGAGGCTTTGCTTACTCCTCGGTTGCCCCAACCAAAGT--GTTTAATAGTCT
T-CCTTTTA-CTTAATCA--GTAAACTCTATTAATTTTCCTAAAGCTCGATAGGGTCTTC
TTGTCTTTTAATCATATCTGGGCTTTTTCACCCAAAAATAAAATTCTAATAAATCTAAAA
GAGACAGGTGTATTCTTGTCAAACCGTTCATTCCAGCCTTCAATTATAAGGCAAATGATT
ATGCTACCTTTGCACGGTCAGAGTACCGCGGCCGTT-TAAAA-CACTGGGCAGGTCCGAC
TTCGTATTTAAT-ACTGACACNACATTATATATTTTATTTGGTATATGGTCTGGCTTAGT
TGGAACGGCTTTAAGATTGTTGATTCGTGCAGAGCTAGGACAACCAGGTGCTTTACTTGG
TGATGATCAATTATATAATGTAATTGTAACAGCTCATGCTTTTGTCATAATTTTTTTCTT
AGTAATACCTATAATAATCGGGGGCTTTGGAAACTGATTAGTACCGTTAATGCTAGGGGC
TCCAGATATAGTATTTCCACGCTTAAATAACATAAGTTTTTGGCTTTTACCGCCCGCACT
TTTACTTCTTTTGTCGTCAGCTGCGGTGGAAAGAGGGGTAGGAACAGGATGAACTGTATA
TCCACCTTTAGCAGGAAACTTGGCGCATGCCGGTGGATCTGTAGATCTAGCAATTTTTTC
TTTACATCTTGCTGGGGTTTCTTCTATTTTAGGAGCGGTAAACTTTATTACTACCATTAT
TAATATACGATGGCAAGGAATAAAATTTGAGCGTCTTTCGTTGTTTGTGTGGTCTGTAAA
AATTACAGCTATTTTACTTCTTTTATCTTTACCTGTCTTGGCCGGAGCTATTACTATGCT

TTTAACTGATCGAAATTTTAATACCGCTTTCTTTGATCCTGCAGGAGGTGGTGATCCTAT
TTTGTACCAACATTTATTT
>textile
AACCAAGAAATTTAGTCATTCTTTTACGGTAAAAAGACAGTTAGGCAAA-AAGGATTATT
GGACCTTTAGTATAGGAGTAAAATCTAAATACTAATAGTTATATCAAGCATAATTT-GTT
-GTGCTGAGTCTGTGATAGCTTTAAGGGAAACTGGGATTGGATACCCCATTATTTTTAGT
TGTAAATTTAGTT--TAGACTTACCGGAGTACTACGAATGTTTAAAACTCAAAGGGCTTG
GCGGTGTTTTAGACCTCTCAGGGGAACCTGTCTCGTAATCGACAATCCGCGTTAAACCTA
ACCTTTTATAGCAATACAGCCTGTATACCGTCGTCGTCAGGTAACTTCTTAAAATATAGT
AGTTAGCCCGAGAATC--AG-ATTAATTAAAACGTCAGATCAAGGTGCAGCTAATAAAAA
GGTGAGGATGGGTTACAATTACATATTTGTAATTACGGTACT-TGAAATGAGGTATTTTA
AAGGAGGACTTGAAAGTAATTTTAATAATATAAATAGAATGAATAGGGCTCTGAAACGTG
CAGAATTTTAATGGTCGAACAGACCAACCCTTAAAGACTTCTGCATCTTTAGGACATTCT
GGTCCAACATCGAGGTCACAAACCTTTTTTTCGATATGGGCTCTTAAAAAAGATAATGCT
GTTATCCCTACGGTAACTAATTCCTTTGATCAAAAAATT-TGGATCACC-ACATGCAGGA
TTCA-AAAGGAGGCTTTACTTGCTCCTCGGTTGCCCCAACCAAAGT--ATTTAATAGTTT
T-CCTTTTA-TCTAATTG--GTAAAGTCTATTAACTTCCCTAAAGCTCGATAGGGTCTTC
TTGTCTTTTAATTATATCTGGACTTTTTCATCCAAAGATAAAATTCTAAGTAATCTAAAA
GAGACAGCTGTATTCTTGTCAAACCATTCATTCCAGCCTTCAATTATAAGGCAAATGATT
ATGCTACCTTTGCACGGTCAGAGTACCGCGGCCGTT-TAAAA-CACTGGGCAGGTCCGAC
TTCGTATTTAAT-ACTTACACGACATTGTATATTTTATTTGGTATATGGTCTGGGTTGGT
CGGAACCGCTCTAAGGTTGTTGATTCGTGCAGAGTTAGGACAACCAGGTGCTTTGCTTGG
AGATGATCAGCTATACAATGTGATTGTAACAGCTCATGCTTTTGTTATAATTTTTTTCTT
GGTAATGCCTATAATAATTGGAGGTTTTGGAAACTGGTTGGTACCCTTAATATTAGGAGC
TCCGGATATAGTATTTCCACGTTTAAATAACATGAGTTTTTGGCTTTTGCCGCCTGCACT
TTTACTTCTTTTATCATCAGCAGCTGTAGAAAGAGGAGTGGGGACAGGATGGACTGTATA
TCCACCTTTAGCAGGAAATTTAGCGCATGCCGGCGGATCTGTAGATCTAGCGATTTTTTC
TTTGCATCTTGCTGGGGTTTCTTCTATTTTAGGAGCTGTAAATTTTATTACTACGATTAT
TAATATACGATGACAAGGAATAAAATTTGAGCGCCTTTCACTGTTTGTATGATCTGTAAA
AATTACAGCTATTTTGCTTCTTTTATCTCTGCCTGTGTTAGCTGGGGCTATTACTATACT
TTTGACTGATCGAAATTTTAATACTGCATTCTTTGATCCAGCAGGAGGTGGGGATCCTAT
TTTATACCAGCATTTATTT
>dalli
AACCAAGAAATTTAGTCATTCTTTTACGGTAAAAAGATAGTTAAGCAAA-AAGGATTATA
AGATCTTTAGTATAGGAGTAAAATCTAGATACTAATAGTTATACCGAGCATAATTT-GTT
-GTGCTGAGTCTGTGATAGCTTTAAGGGAAACTGGGATTGGATACCCCATTATTTTTAGT
TGTAAATTTAGTT--TAGGCTTACCAGAGTACTACGAATGTTTAAAACTCAAAGGGCTTG
GCGGTGTTTTAGACCTCTCAGGGGAACCTGTCTCGTAATCGACAATCCGCGTTAAACCTA
ACCTTTTATAGCAATACAGCCTGTATACCGTTGTCGTCAGGTAACTTCTTAAAATATAGT
AGTTAGCTCGAGAATT--AG-ATTAATTAAAACGTCAGATCAAGGTGCAGCTAATAAAAA
GGTGAGGATGGGTTACAGTTACATATTTGTAATTACGGTGCT-TGAAATGAAGTATTTTA
AAGGAGGACTTGAAAGTAATTTTAATAATATAAGTAGGATGAATAGGGCTCTGAAACGTG
CAGAATTTTAATGGTCGAACAGACCAACCCTTAAAGACTTCTGCATCTTTAGGATATTCT
GGTCCAACATCGAGGTCACAAACCTTTTTTTCGATATGGGCTCTTAAAAAAGATAATGCT
GTTATCCCTACGGTAACTAATTCCTTTGATCAAAAATTT-TGGATCAAT-ACAAGCAAGA
TTTA-AAAGGAGGCTTTACTTACTCCTCGGTTGCCCCAACCAAAGT--ATTTAATAGTTT
T-CCTTTTA-TCTTATTG--GTAAAATCTATTAACTTCTCTAAAGCTCGATAGGGTCTTC
TTGTCTTTTAATCATATCTGGACTTTTTCATCCAAAGATAAAATTCTAAGTAATCTAAAA

GAGACAGGTGTATTCTTGTCAAACCATTCATTCCAGCCTTCAATTATAAGGCAAATGATT
ATGCTACCTTTGCACGGTCAGAGTACCGCGGCCGTT-TAAAA-CACTGGGCAGGTCCGAC
TTCGTATTTAAT-ACCTACACGACATTGTATATTTATTTGGTATATGATCTGGGCTGGT
TGGAACTGCCCTAAGATTATTAATTCGTGCAGAGCTAGGGCAACCAGGTGCTTTGCTAGG
GGATGACCAACTATATAATGTGATCGTAACAGCCCATGCTTTTGTTATGATTTTTTTCTT
AGTAATGCCTATGATAATTGGAGGATTTGGGAACTGGTTGGTACCCTTAATATTAGGAGC
TCCAGATATAGTGTTTCCACGTTTAAATAATATGAGTTTTTGGCTTTTGCCGCCTGCGCT
TTTACTTCTTTTATCATCAGCAGCTGTTGAAAGAGGAGTAGGGACAGGATGAACTGTATA
TCCACCCTTAGCAGGAAATTTGGCGCATGCCGGCGGGTCTGTAGATCTAGCAATTTTTTC
TTTACATCTTGCTGGTGTTTCTTCTATTTTAGGGGCTGTAAACTTTATTACTACGATTAT
TAATATACGATGACAAGGAATAAAATTTGAGCGTCTTTCGCTGTTTGTGTGATCTGTAAA
AATTACGGCTATTTTGCTTCTTTTATCTCTGCCTGTATTGGCTGGAGCCATTACTATGCT
TTTAACTGATCGAAACTTTAATACTGCCTTCTTTGATCCTGCAGGAGGTGGGGATCCTAT
TTTATATCAGCATCTATTT
>canonicus
NNNNNNNNNNNNNNNNNNNNNNNNNTACGGT-AAAAGATAGTTAAGCAAA-AAGGATTATA
AGATCTTTAGTATAGGAGTAAAATCTAGATACTAATAGTTATACCGAGCATAATTT-GTT
-GTGCTGAGTCTGTGATAGCTTTAAGGGAAACTGGGATTGGATACCCCATTATTTTTAGT
TGTAAATTTAGTT--TAGGCTTACCAGAGTACTACGAATGTTTAAAACTCAAAGGGCTTG
GCGGTGTTTTAGACCTCTCAGGGGAACCTGTCTCGTAATCGACAATCCGCGTTAAACCTA
ACCTTTTATAGCAATACAGCCTGTATACCGTCGTCGTCAGGTAACTTCTTAAAATATAGT
AGTTAGCTCGAGAATT--AG-ATTAATTAAAACGTCAGATCAAGGTGCAGCTAATAAAAA
GGTGAGGATGGGTTACAATTACATATTTGTAATTACGGCGCT-TGAAATGAAGCATCTTA
AAGGAGGACTTGAAAGTAATTTTAATAATATAAGTAAGATGAATAGGGCTCTGAAACGTG
NNNNATTTTAATGGTCGAACAGACCAACCCTTAAAGACTTCTGCATCTTTAGGATATTCT
GGTCCAACATCGAGGTCACAAACCTTTTTTTCGATATGGGCTCTTAAAAAAGATAATGCT
GTTATCCCTACGGTAACTAATTCCTTTGATCAAAAATTT-TGGATCAAT-ACAAGCAAGA
TCTA-AAAGGAGGCTTTACTTACTCCTCGGTTGCCCCAACCAAAGT--ATTTAATAGTTT
T-CCTTTTA-TCTTATTG--GTAAAATCTATTAACTTCTCTAAAGCTCGATAGGGTCTTC
TTGTCTTTTAATCATATCTGGACTTTTTCATCCAAAGATAAAATTCTAAGTAATCTAAAA
GAGACAGGTGTATTCTTGTCAAACCATTCATTCCAGCCTTCAATTATAAGGCAAATGATT
ATGCTACCTTTGCACGGTCAGAGTACCGCGGCCGTT-TAAAA-CACTGGGCAGGTCCGAC
TTCGTATNNNNN-NNNNNNNNNNNNATTGTATATTTATTTGGTATATGATCTGGGCTGGT
TGGAACTGCCCTAAGATTGTTAATTCGTGCAGAGCTGGGGCAACCAGGTGCTTTGCTAGG
GGATGACCAACTATATAATGTGATCGTAACAGCCCATGCTTTTGTTATGATTTTTTTCTT
AGTAATGCCTATGATAATTGGAGGATTTGGGAACTGGTTGGTACCCTTAATATTAGGAGC
TCCAGATATAGTGTTTCCACGTTTAAATAATATGAGTTTTTGGCTTTTGCCGCCTGCGCT
TTTACTTCTTTTATCATCAGCAGCTGTTGAAAGAGGAGTAGGGACGGGATGAACTGTATA
TCCACCCTTAGCAGGAAATTTGGCGCATGCCGGCGGGTCTGTAGATCTAGCAATTTTTTC
TTTACATCTTGCTGGTGTTTCTTCTATTTTAGGGGCTGTAAACTTTATTACTACGATTAT
TAATATACGATGACAAGGAATAAAGTTTGAGCGTCTTTCGCTGTTTGTGTGATCTGTAAA
AATTACGGCTATTTTGCTTCTTTTATCTCTGCCTGTATTGGCTGGAGCCATTACTATGCT
TTTAACTGATCGAAACTTTAATACTGCCTTCTTTGACCCTGCAGGAGGTGGGGATCCTAT
TTTATATCAACATCTATTT
>ammiralis
AACCAAGAAATTTAGTCATTCTTTTATGGTAAAAAGACAGTTAGGCAAG-AAGGACTATA
GGACCTATAGTATAGGAGTAAAATCTAGATACTAATAGTTATACCGGTTGTGGTTT-ATT
-GTGCTGAGTCTGTGATAGCTTTAAGGGAAACTGGGATTGGATACCCCATTATTTTTAGT

TGTAAATTTAGTT--TAGGCTTACCAGAGTACTACGAATGTTTAAAACTCAAAGGGCTTG
GCGGTGTTTTAGACCTCTCAGGGGAACCTGTCTCGTAATCGACAATCCACGTTATACCTG
ACCTTTTACAGCGATACAGCCTGTATACCGTCGTCGTCAGGTAACTTCTTAAAATATAGT
AGTTAGCTCGAGAGTT--AG-ATTAGTTAAAACGTCAGATCAAGGTGCAGCTAATAAAAA
GGTGAGGATGGGTTACAATTATATATTTATAAATACGGTACT-TGAAATGAAGTATCTTA
AAGGAGGACTTGAAAGTAATTCTAATAATATAAATAGGATGAATAGGGCTCTGAAACGTG
CAGAATTTTAATGGTCGAACAGACCAACCCTTAAAGACTTCTGCATCTTTAGGACATTCT
GGTCCACCATCGAGGTCACAAACCTTTTTTTCGATATGGGCTCTTAAAAAAGATAATGCT
GTTATCCCTACGGTAACTAATTCCTTTGATCAAAAATTT-TGGATCAAC-ACAGGTAAGA
TTTA-AAAGGAGGCTTTACTTACTCCTCGGTTGCCCCAACCAAAGT--ATTTAATAGCTT
T-CCTTTTA-TCTAATTG--GTGAA-TCTATTAATTTCTCTAAAGCTCGATAGGGTCTTC
TTGTCTTTTAATTATATCTGGACTTTTTCATCCAAAGATAAAGTTCTAAGTAATCTAAAA
GAGACAGCTGTATTCTTGTCAAACCATTCATTCCAGCCTTCAATTATAAGGCAAATGATT
ATGCTACCTTTGCACGGTCAGAGTACCGCGGCCGTT-TAAAA-CACTGGGCAGGTCCGAC
TTCGTATTTAAT-ATCTACACGACATTGTATATTTTGTTTGGTATATGATCTGGGTTGGT
TGGAACTGCCCTAAGGCTATTGATTCGTGCAGAGTTAGGGCAACCAGGTGCTTTGCTTGG
GGATGATCAGCTATATAATGTAATTGTAACAGCCCATGCTTTTGTTATAATTTTTTTCTT
AGTTATACCTATGATAATTGGGGGTTTTGGAAACTGGTTGGTACCACTAATATTAGGGGC
TCCAGATATAGTATTTCCACGTTTAAATAATATAAGTTTTTGGCTTTTGCCACCTGCACT
TTTACTTCTTTTATCATCAGCAGCTGTAGAAAGAGGGGTAGGGACGGGATGAACTGTATA
TCCACCTTTAGCGGGAAATTTGGCGCATGCCGGCGGATCTGTAGATCTAGCGATTTTCTC
TTTACATCTTGCTGGGGTTTCTTCTATTTTAGGGGCTGTAAATTTTATTACTACAATTAT
TAATATACGATGACAAGGAATAAAATTTGAGCGTCTTTCGTTGTTTGTATGGTCTGTAAA
AATTACGGCTATTTTGCTTCTTTTATCTCTGCCTGTGTTAGCTGGAGCCATTACTATGCT
TTTAACTGATCGAAATTTTAATACTGCTTTTTTTGACCCTGCAGGAGGTGGGGATCCTAT
TTTATACCAGCATTTATTT
>omaria
AACCAAGAAATTTAGTCATTCTTTTATGGTAAAAAGACAGTTAAGCAAA-AAAGATCATT
AGACCTTTAGTACAGGAGTAAAATCTAAATACTAAAAGTTATACCGGTTATGGTTT-ATT
TGTGCTGAGGCTGTGATAGCTTTAAGGGAAACTGGGATTGGATACCCCATTATTTTTAGT
TGTAAATCTAGTT--TAAGCTTACCAGAGTACTACGAATGTTTAAAACTCAAAGGGCTTG
GCGGTGTTTTAGACCTCTCAGGGGAACCTGTCTCGTAATCGACAATCCACGTTAGACCTA
ACCTCTTATGGCAACACAGCCTGTATACCGTCGTCGTCAGGTAACTTCTTAAAATATAGT
AGTTAGCTCGAGAATT--AA-ATTAATTAGAACGTCAGATCAAGGTGCAGCTAATGAAGA
GGTGAGGATGGGTTACAATTACATATTTGTAAATACGGTACT-TGAAATATAGTATCATA
AAGGAGGACTTGAAAGTAATCTTAGTTATATAAATAAGATGAATATGGCTCTGAAACGTG
CAGAATTTTAATGGTCGAACAGACCAACCCTTAAAGACTTCTGCATCTTTAGGATATTCT
GGTCCAACATCGAGGTCACAAACCTTTTTTTCGATATGGGCTCTTGAAAAAGATAATGCT
GTTATCCCTACGGTAACTAATTCCTTTGATCAAAAAGTT-TGGATCAGC-ACAGGTAAGA
CTTA-AAAGGAGGCTTTATTTGCTCCTCGGTTGCCCCAACCAAAGT--ATTTAATAGTCT
T-CCTTTTA-CTTAATTG--ATAAACTCTATTAATTTTTCTAAAGCTCGATAGGGTCTTC
TTGTCTTTTAATCATATCTGGACTTTTTCATCCAAAAATAAAATTCTAGTCAATCTAAAA
GAGACAGGTGTATTCTTGTCAAACCATTCATTCCAGCCTTCAATTATAAGGCAAATGATT
ATGCTACCTTTGCACGGTCAGAGTACCGCGGCCGTT-TAAAA-CACTGGGCAGGTCCGAC
TTCGTATTTAA--GCTGACACGACATTATATATTTTATTTGGTATGTGGTCCGGGTTAGT
TGGGACAGCCCTAAGGTTGCTGATTCGTGCAGAGTTGGGACAACCGGGTGCTTTGCTTGG
CGATGATCAGTTATATAATGTTATTGTAACAGCTCATGCTTTTGTTATGATTTTTTTCTT
GGTAATGCCAATGATAATTGGTGGCTTTGGGAACTGATTGGTACCTTTAATATTAGGAGC

TCCAGATATGGTGTTCCCACGATTAAATAATATAAGCTTTTGGCTTTTGCCACCTGCACT
TCTACTTCTTTTATCATCAGCTGCAGTGGAAAGTGGCGTGGGTACTGGATGGACTGTATA
TCCACCTCTTGCAGGAAATCTAGCTCATGCTGGTGGTTCTGTAGATCTTGCAATTTTTTC
TTTACATCTTGCTGGGGTATCTTCTATTTTAGGAGCTGTAAACTTTATTACCACAATTAT
TAATATACGGTGACAAGGAATGAAATTTGAGCGTCTTTCGTTATTTGTATGATCGGTGAA
GATTACGGCTATTTTGCTTCTTTTATCTTTACCTGTTTTGGCAGGGGCTATTACTATACT
TTTGACTGATCGAAATTTTAATACTGCTTTCTTTGATCCAGCAGGAGGTGGTGATCCTAT
TTTGTACCAACATTTATTT
>furvus
AACCAAGAAATTTAGTCATTCTTTTTTGGTAAAAAGACAGTTAGGTAGT-TAGAATTATA
GGACCTTTAGTGCAGGAGTAAAATCTAAATATTAAAAGTTATTTCAGGTATAATTT-ATT
TTTGCTGAATCTGTGATAGCTTTAAGGGAAACTGGGATTGGATACCCCATTATTTTTAGT
TGTAAATATAGTT--TAAACTTACCAGAGTACTACGAATGTTTAAAACTCAAAGGGCTTG
GCGGTGTTTTAGACCCTTCAGGGGAACCTGTCTCGTAATCGACAATCCACGTTAAACCTA
ATCTCTTGTAGCAATACAGCTTGTATACCGTCGTCGTCAGGTAACTTCTTAAAATATAGT
AGTTAGCTTGATAATC--AG-ATTAGTTAAAACGTCAGATCAAGGTGCAGCTAATAAAGA
GGTGAGGATGGGTTACAATTATATATTTGTAAATACGGTACT-TGAAATAAGGTATTTTA
AAGGAGGACTTGAAAGTAATTTTAGTTATATAAATAAGATGAATAAGGCTCTGAAACGTG
CAGAATTTTAATGGTCGAACAGACCAACCCTTAAAGACTTCTGCATCTTTAGGACATTCT
GGTCCAACATCGAGGTCACAAACCTTTTTTTCGATATGGGCTCTTAAAAAAGATAATGCT
GTTATCCCTACGGTAACTAATTTCTTTAATCAAAAACTT-TGGATCAAT-ACAAGTAAGA
CTCA-AAAGGAGGCTTTACCTACTCCTCGGTTGCCCCAACCAAAAT--ATTTAATAGCTT
T-TCTTTTA-CTCAATTA--A-AAAATCTATCAACTTCTTTAAAGCTCGATAGGGTCTTC
TTGTCTTTTAATCATATCTGAACTTTTTCATCCAAAAATAAAATTCTAAGCAATCTAAAA
GAGACAGGTGTATTCTTGTCAAACCATTCATTCCAGCCTTCAATTATAAGGCAAATGATT
ATGCTACCTTTGCACGGTCAGAGTACCGCGGCCGTT-TAAAA-CACTGGGCAGGTCCGAC
TTTGTATTTAAA-ATTGACACGACATTGTATATTTTATTTGGTATGTGATCTGGACTAGT
TGGAACGGCTCTTAGATTATTAATTCGTGCAGAGTTAGGGCAACCAGGTGCTTTGCTTGG
AGATGATCAGCTATATAATGTAATTGTAACAGCTCATGCCTTTGTTATGATTTTTTTTT
AGTAATACCTATAATAATTGGAGGTTTCGGGAACTGGTTAGTACCTTTAATATTAGGAGC
ACCGGATATGGTATTTCCGCGCTTAAATAATATAAGTTTTTGGCTTTTACCTCCTGCGCT
TTTACTTCTCTTATCGTCGGCTGCTGTAGAAAGTGGAGTAGGAACAGGATGAACTGTATA
TCCTCCTCTGGCGGGGAATTTAGCACATGCTGGTGGATCTGTAGATCTAGCAATTTTTTC
TTTGCATCTTGCAGGAGCTTCTTCTATCTTAGGAGCAGTAAATTTTATTACTACGATTAT
CAATATACGATGACAAGGAATAAAGTTTGAGCGTCTTTCATTGTTTGTATGATCTGTAAA
AATTACAGCTATTTTGCTTCTCTTATCTTTGCCTGTATTGGCTGGGGCAATTACTATACT
TTTAACTGATCGAAATTTTAATACTGCTTTCTTTGATCCTGCAGGAGGTGGTGATCCTAT
TTTATATCAGCATTTGTTT
>araneosus
AACCAAGAAATTTAGTCATTCTTTTACGGTAAAAAGACAGTTAAGCAAA-ATTAGCCATT
AGATCCTTAGTATAGGAGTAAAATCTAGATACTAAAAGTTATATCGAAAATGGTTT-GTT
TGTGTTGAATCTGTGATAGCTTTAAGGGAAACTGGGATTGGATACCCCATTATTTTTAGC
TGTAAATTTAGTT--TAAGCTTACCGGAGTACTACGAATGTTTAAAACTCAAAGGGCTTG
GCGGTGTTTTAGACCTCTCAGGGGAACCTGTCTCATAATCGATAATCCACGTTAGACCTA
ACCTTTTGTAGCATGTCAGCTTGTATACCGTCGTCGTCAGGTAACTTCTTAAAATACAGT
AGTTAGCTCGATAATT--TA-GTTAATTAACACGTCAGATCAAGGTGCAGCTAATAAAGA
GGCGAGGATGGGTTACAATTACATATTTGTAAATACGACACT-TGAAATAAAATGTTTCA
AAGGAGGACTTGAAAGTAATTTTAGTTATATAAATAGAATGAATCTGGCTCTGAAACGTG

CAGAATTTTAATGGTCGAACAGACCAACCCTTAAAGACTTCTGCATCTTTAGGACATTCT
GGTCCAACATCGAGGTCACAAACCTTTTTTTCGATATGGGCTCTCAAAAAAGATAATGCT
GTTATCCCTACGGTAACTAATTCCTTTGATCAAAAATTT-TGGATCAAC-ATAAGTAAGA
TTTG-AGAGGAGGCTTTATTTACTCCTCGGTTGCCCCAACCAAAGT--ATTTAATAGCCT
T-TCTTTTA-TTTAATAG--ATAGAGTCTACTAATTTTTCTAAAGCTCGATAGGGTCTTC
TTGTCTTTTAATCGTATCTGGACTTTTTCATCCAAAAATAAAATTCTAAGCAATCTAAAA
GAGACAGGTGTATTCTTGTCAAACCATTCATTCCAGCCTTCAATTATAAGGCAAATGATT
ATGCTACCTTTGCACGGTCAGAGTACCGCGGCCGTT-TAAAA-CACTGGGCAGGTCCGAC
TTCGTATTTAAT-ATTAACACGACATTGTATATTTTATTTGGAATGTGATCTGGCTTAGT
TGGGACCGCCCTTAGGTTGTTAATTCGTGCAGAATTAGGACAACCGGGTGCCTTGCTTGG
AGATGATCAGCTGTATAATGTAATTGTGACAGCTCATGCTTTTGTTATGATTTTTTTCTT
AGTAATACCCATAATAATCGGGGGCTTTGGAAATTGATTAGTGCCTTTGATGTTAGGAGC
TCCAGATATAGTATTTCCACGTTTAAATAATATGAGTTTTTGGTTATTACCTCCTGCACT
TTTACTTCTTTTATCGTCGGCTGCAGTGGAGAGTGGAGTAGGTACAGGATGGACTGTATA
TCCGCCATTAGCTGGGAATTTAGCGCATGCTGGTGGGTCAGTAGATCTAGCAATTTTTTC
TTTACATCTTGCTGGTGTCTCTTCTATTTTAGGGGCAGTAAATTTTATTACTACGATCAT
TAATATACGATGACAGGGAATAAAATTTGAGCGTCTTTCGTTGTTTGTATGGTCTGTAAA
AATTACAGCAATTTTGCTTCTCTTGTCTCTACCAGTATTGGCTGGAGCTATTACTATGCT
TTTAACTGATCGAAATTTTAACACTGCTTTTTTTGATCCAGCAGGAGGCGGTGATCCTAT
CTTATATCAGCATTTATTT
>bandanus
AACCAAGAAATTTAGTCATTCTTTTATGGTAAAAAGACAGTTAAACAAA-ATTAGTCATT
GGATCCTTAGTATAGGAGTAAAATCTAAATACTAAGAGTTATACCGAAGATGGTTT-GTT
TGTGTTGAATCTGTGATAGCTTTAAGGGAAACTGGGATTGGATACCCCATTATTTTTAGT
TGTAAATTTAGTT--TGAGCTTACCGGAGTACTACGAATGTTTAAAACTCAAAGGGCTTG
GCGGTGTTTTAGACCTCTTAGGGGAACCTGTCTCATAATCGATAATCCACGTTAAACCTA
ACCTTTTATAGCAAAACAGCCTGTATACCGTCGTCGTCAGGTAACTTCTTAAAATATAGT
AGTTAGCTTGACAATT--TA-GTTAATTAAGACGTCAGATCAAGGTGCAGCCAATAAAAA
GGTGAGGATGGGTTACAATTACACATTTGTAAATACGGCACT-TGAAATAAAGTGTTCTG
AAGGAGGACTTGAAAGTAATTTTAGTTATATAGGTAAAATGAATCTGGCTCTGAAGCGTG
CAGAATTTTAATGGTCGAACAGACCAACCCTTAAAGACTTCTGCATCTTTAGGACATTCT
GGTCCAACATCGAGGTCACAAACCTTTTTTTCGATATGAGCTCTCAAAAAAGATAATGCT
GTTATCCCTACGGTAACTAATTCCTTTGATCAAAAATTT-TGGATCAAC-ACAAGCAAGA
TTTA-AAAGGAGGCTTTATTTACTCCTCGGTTGCCCCAACCAAAGT--GTTTAATAGCTT
T-TCTTTTA-CTTAATAG--ATAAAATCTACTAATTCTTCTAAAGCTCGATAGGGTCTTC
TTGTCTTTTAATTATATCTAGACTTTTTCATCCAGAGATAAAATTCTAAGCAATCTAAAA
GAGACAGGTGTATTCTTGTCAAACCATTCATTCCAGCCTTCAATTATAAGGCAAATGATT
ATGCTACCTTTGCACGGTCAGAGTACCGCGGCCGTT-TAAAA-CACTGGGCAGGTCCGAC
TTCGTATCTAAT-ACTAACATGACATTATATATTTTATTTGGAATGTGATCTGGTTTGGT
TGGAACTGCTCTCAGGTTGTTAATTCGTGCAGAGTTAGGACAACCAGGTGCTTTGCTTGG
GGACGATCAACTATATAATGTAATTGTAACAGCTCATGCTTTTGTTATAATTTTTTTCTT
AGTGATGCCTATAATGATTGGGGGTTTTGGGAATTGATTAGTACCATTAATATTAGGGGC
TCCGGATATGGTATTTCCACGTTTAAATAATATAAGTTTTTGATTATTGCCTCCTGCACT
TTTGCTTCTTTTGTCATCTGCTGCGGTAGAAAGTGGAGTGGGTACGGGGTGAACTGTGTA
TCCGCCATTAGCTGGAAATCTAGCACATGCTGGTGGGTCAGTAGACCTAGCAATTTTTTC
TTTACATCTTGCTGGTGTTTCTTCTATTTTAGGGGCAGTAAACTTTATTACTACAATTAT
TAATATACGATGACAGGGAATAAAATTTGAACGTCTTTCGTTGTTTGTCTGATCTGTGAA
AATTACAGCAATTTTGCTTCTTTTGTCTTTACCTGTATTGGCCGGTGCAATTACTATACT

TTTGACTGATCGAAATTTCAATACTGCTTTTTTTGATCCGGCAGGAGGTGGTGATCCTAT
TTTATATCAGCACTTGTTC
>nigrescens
--NNNNNNNNNNNNNNNNNNNNNNNNNNNNAAAAAGACAGTTAAACGAG-ATTAGTCATT
AGATCCTTAGTATAGGAGTAAAATCTAAATACTAAGAGTTATACCGAAAATGGTTT-GTT
TGTGTTGAATCTGTGATAGCTTTAAGGGAAACTGGGATTGGATACCCCATTATTTTTAGT
TGTAAATTTAGTT--TAAGCTTACCGGAGTACTACGAATGTTTAAAACTCAAAGGGCTTG
GCGGTGTTTTAGACCTCTCAGGGGAACCTGTCTCATAATCGATAATCCACGTTAAACCTA
ACCTTTTATAGCAAAACAGCCTGTATACCGTCGTCGTCAGGTAACTTCTTAAAATATAGT
AGTTAGCTTGATAATT--TA-GTTAATTAAGACGTCAGATCAAGGTGCAGCCAATAAAAA
GGTGAGGATGGGTTACAATTACATATTTGTAAACACGACACT-TGAAATAAAGTGTTCCG
AAGGAGGACTTGAAAGTAATTTTAGTTATATAGGTAGAATGAATCTGGCTCTGAAGCGTG
NAGAATTTTAATGGTCGAACAGACCAACCCTTAAAGACTTCTGCATCTTTAGGACATTCT
GGTCCAACATCGAGGTCACAAACCTTTTTTTCGATATGAGCTCTCAAAAAAGATAATGCT
GTTATCCCTACGGTAACTAATTCCTTTGATCAAAAATTT-TGGATCAAC-ACAAGCAAGA
TTTA-AAAGGAGGCTTTATTTACTCCTCGGTTGCCCCAACCAAAGT--GTTTAATAGTTT
T-TCTTTTA-CTTAATAG--ATAAAATCTACTAATTCTTCTAAAGCTCGATAGGGTCTTC
TTGTCTTTTAATTATATCTAGACTTTTTCATCCAGAGATAAAATTCTAAGCAATCAGAAA
GAGACAGGTGTATTCTTGTCAAACCATTCATTCCAGCCTTCAATTATAAGGCAAATGATT
ATGCTACCTTTGCACGGTCAGAGTACCGCGGCCGTT-TAAAA-CACTGGGCAGGTCCGAC
TTCGTATCTAAC-ACTAACACGACATTATATATTTTATTTGGAATGTGATCTGGTTTGGT
TGGAACTGCTCTCAGGTTGTTAATTCGTGCAGAATTAGGACAACCAGGTGCTTTGCTTGG
GGACGATCAACTATATAATGTAATTGTAACAGCTCATGCTTTTGTTATAATTTTTTTCTT
AGTGATGCCTATAATGATTGGGGGTTTTGGGAATTGATTAGTACCATTAATATTAGGGGC
TCCGGATATAGTATTTCCACGTTTAAATAATATAAGTTTTTGATTATTACCTCCTGCACT
TTTGCTTCTTTTATCATCTGCTGCAGTAGAAAGTGGAGTAGGTACGGGATGAACTGTGTA
TCCACCATTAGCTGGAAATCTAGCACATGCTGGCGGGTCGGTAGACCTAGCAATTTTTTC
TTTACATCTTGCTGGTGTTTCTTCTATTTTAGGGGCAGTAAATTTTATTACTACAATTAT
TAATATACGATGACAAGGAATAAAATTTGAACGTCTTTCGTTGTTTGTCTGATCTGTGAA
AATTACAGCAATTTTGCTTCTTTTATCTTTACCTGTATTGGCTGGCGCAATTACTATACT
TTTGACTGATCGAAATTTCAATACTGCTTTTTTCGATCCGGCAGGAGGTGGTGATCCTAT
TTTATACCAGCACTTATTT
>marmoreus
AACCAAGAAATTTAGTCATTCTTTTATGGTAAAAAGACAGTTAAGCAAA-ATTAGTCATT
AGATCCTTAGTATAGGAGTAAAATCTAAATACTAAGAGTTATACCGAAGATGGTTT-GTT
TGTGTTGAGTCTGTGATAGCTTTAAGGGAAACTGGGATTGGATACCCCATTATTTTTAGT
TGTAAATTTAGTT--TAAGCTTACCGGAGTACTACGAATGTTTAAAACTCAAAGGGCTTG
GCGGTGTTTTAGACCTCTCAGGGGAACCTGTCTCATAATCGATAATCCACGTTAAACCTA
ACCTTTTATAGCAGAACAGCCTGTATACCGTCGTCGTCAGGTAACTTCTTAAAATATAGT
AGTTAGCTTGACAATT--TA-GTTAATTAAGACGTCAGATCAAGGTGCAGCCAATAAAAA
GGTGAGGATGGGTTACAATTACATATTTGTAAATACGACACT-TGAAATAAAGTGTTCCG
AAGGAGGACTTGAAAGTAATTTTAGTTATATAGATAGAATGAATCTGGCTCTGAAGCGTG
CAGAATTTTAATGGTCGAACAGACCAACCCTTAAAGACTTCTGCATCTTTAGGACATTCT
GGTCCAACATCGAGGTCACAAACCTTTTTTTCGATATGAGCTCTCAAAAAAGATAATGCT
GTTATCCCTACGGTAACTAATTCCTTTGATCAAAAATTT-TGGATCAAC-ACAAGTAAGA
TTTA-AAAGGAGGCTTTATTTACTCCTCGGTTGCCCCAACCAAAGT--ATTTAATAGTTT
T-TCTTTTA-CTTAATAG--ATAAAATCTACTAATTCTTCTAAAGCTCGATAGGGTCTTC
TTGTCTTTTAATTATATCTAGACTTTTTCATCCAGAGATAAAATTCTAAGCAATCTAAAA

GAGACAGGTGTATTCTTGTCAAACCATTCATTCCAGCCTTCAATTATAAGGCAAATGATT
ATGCTACCTTTGCACGGTCAGAGTACCGCGGCCGTT-TAAAA-CACTGGGCAGGTCCGAC
TTCGTATCTAAC-ACTAACACGACATTATATATTTTATTTGGAATGTGATCTGGTTTGGT
TGGAACTGCTCTCAGGTTGTTAATTCGTGCAGAATTAGGACAACCAGGTGCTTTGCTTGG
GGACGATCAACTATATAATGTAATTGTAACAGCTCATGCTTTTGTTATGATTTTTTTTTT
AGTAATGCCTATAATGATTGGGGGTTTTGGGAATTGATTAGTACCATTAATATTAGGGGC
TCCGGATATAGTATTTCCGCGTTTGAATAATATAAGTTTTTGATTATTACCTCCTGCACT
TTTGCTTCTTTTGTCATCTGCTGCAGTAGAAAGTGGAGTTGGTACGGGGTGAACTGTGTA
TCCACCATTAGCCGGAAATCTAGCACATGCTGGCGGGTCGGTAGACCTAGCAATTTTTTC
CTTACATCTTGCTGGTGTTTCTTCTATTTTAGGGGCAGTAAATTTTATTACTACAATTAT
TAATATACGATGACAGGGAATAAAATTTGAACGTCTTTCGTTATTTGTGTGATCTGTGAA
GATTACAGCAATTTTGCTTCTTTTGTCTTTACCTGTATTGGCTGGTGCAATTACTATACT
TTTGACTGATCGAAATTTCAATACTGCTTTTTTTGATCCGGCAGGGGGTGGTGATCCTAT
TTTATATCAGCACTTGTTC
>eburneus
AACCAAGAAATTTAGTCATTCTTTTACGGTAAAAAGATAGTTAGGCAAG-AAGGACCACT
GGATCTTTAGTGGAGGAGTAAAATCTAGACACTAAAAGTTACACCGGACGTGGTTTAATT
TGTGCTGAATCTGTGATAGCTTTAAGGGAAACTGGGATTGGATACCCCATTATTTATAGT
TGTAAATTTAGTT--TGAGCTTACCAGAGTACTACGAATGTTTAAAACTCAAAGGGCTTG
GCGGTGTTTTAGACCTCTCAGGGGAACCTGTCTCGTAATCGACAATCCACGTTAGACCTA
ACCTTTTATGGCGAAACAGCCTGTATACCGTCGTCGTCAGGTAACTTCTTAAAATATAGT
AGTTAGCTCGAGAATT--TT-ATTAATTAAGACGTCAGATCAAGGTGCAGCCAATAAAAA
GGTGAGGATGGGTTACAATTACATATTTGTAAATACGGCGTT-TGAAATTAAGTGTTTTA
AAGGAGGACTTGAAAGTAATTTTAGTTATATAAATAGGATGAATAAGGCTCTGAAACGTG
CAGAATTTTAATGGTCGAACAGACCAACCCTTAAAGACTTCTGCATCTTTAGGACATTCT
GGTCCAACATCGAGGTCACAAACCTTTTTTTCGATATGGGCTCTTGAAAAAGATAATGCT
GTTATCCCTACGGTAACTAATTCCTTTGATCAAGAAATT-TGGATCAAC-ACAAGTAAGA
CTTA-AGAGGAGGCTTTACTTGCTCCTCGGTTGCCCCAACCAAAGT--ATTTAATAGTTT
T-TCTTTTA-CTTAATTG--ATAAAGTCTATTAATTTCTCTAAAGCTCGATAGGGTCTTC
TTGTCTTTTAATTCAATCTAGGCTTTTTCACCTAAAAATAAAATTCTATAGAATCTAAAA
GAGACAGGTGTATTCTTGTCAAACCATTCATTCCAGCCTTCAATTATAAGGCAAATGATT
ATGCTACCTTTGCACGGTCAGAGTACCGCGGCCGTT-TAAAA-CACTGGGCAGGTCCGAC
TTCACATCTTGT-AATAACACGACATTATATATCTTATTTGGTATATGATCTGGGTTGGT
TGGAACTGCTCTAAGGTTGCTAATTCGTGCAGAGTTGGGACAACCGGGGGCTTTACTTGG
AGACGATCAATTATATAATGTAATTGTGACAGCTCATGCTTTTGTTATGATTTTTTTCTT
GGTTATGCCTATGATGATTGGGGGCTTTGGAAATTGATTGGTGCCATTAATATTAGGGGC
TCCTGATATGGTATTTCCTCGGTTAAATAATATAAGTTTTTGACTTCTTCCTCCCGCACT
TTTACTCCTTCTGTCATCGGCTGCGGTGGAAAGTGGAGTAGGTACTGGGTGGACTGTATA
TCCACCTCTAGCAGGAAATTTGGCACATGCTGGTGGGTCTGTAGACCTGGCTATTTTCTC
TTTACACCTTGCTGGTGTGTCTTCTATTTTGGGGGCGGTAAATTTTATTACTACAATTAT
TAATATACGATGACAGGGGATGAAATTTGAACGCCTTTCGTTGTTTGTATGGTCGGTGAA
AATTACGGCTATTTTACTTTTGTTATCTTTGCCTGTGTTGGCTGGTGCAATTACTATGCT
TTTAACTGATCGGAATTTTAATACTGCTTTCTTTGATCCAGCAGGAGGTGGAGATCCTAT
CTTATATCAGCATTTATTT
>tessulatus
AACCAAGAAATTTAGTCATTCTTTTACGGTAAAAAGATAGTTAGGCAAG-AAGGACCACT
GGATCTTTAGTGGAGGAGTAAAATCTAGACACTAAAAGTTATACCGGACGTGGTTTAATT
TGTGCTGAATCTGTGATAGCTTTAAGGGAAACTGGGATTGGATACCCCATTATTTATAGT

TGTAAATTTAGTT--TGAGCTTACCAGAGTACTACGAATGTTTAAAACTCAAAGGGCTTG
GCGGTGTTTTAGACCTCTCAGGGGAACCTGTCTCGTAATCGACAATCCACGTTAGACCTA
ACCTTTTATGGCGAAACAGCCTGTATACCGTCGTCGTCAGGTAACCTCTTAAAATATAGT
AGTTAGCTCGAGAATT--TT-ATTAATTAAGACGTCAGATCAAGGTGCAGCCAATGAAAA
GGTGAGGATGGGTTACAATTACATATTTGTAAATACGGCGTT-TGAAACTAAGTGTTTTA
AAGGAGGACTTGAAAGTAATTTTAGTTATATAAATAGAATGAATAAGGCTCTGAAACGTG
CAGAATTTTAATGGTCGAACAGACCAACCCTTAAAGACTTCTGCATCTTTAGGACATTCT
GGTCCAACATCGAGGTCACAAACCTTTTTTTCGATATGGGCTCTTGAAAAAGATAATGCT
GTTATCCCTACGGTAACTAATTCCTTTGATCAAAAAGTT-TGGATCAAC-ACAAGTAAGA
CTTA-AGAGGAGGCTTTACTTGCTCCTCGGTTGCCCCAACCAAAGT--ATTTAATAGTTT
T-TCTTTTA-CTTAATTG--ATAAAGTCTATTAACTTCTCTAAAGCTCGATAGGGTCTTC
TTGTCTTTTAATTCAATCTAGGCTTTTTCACCTAAAAATAAAATTCTATAGAATCTAAAA
GAGACAGGTGTATTCTTGTCAAACCATTCATTCCAGCCTTCAATTATAAGGCAAATGATT
ATGCTACCTTTGCACGGTCAGAGTACCGCGGCCGTT-TAAAA-CACTGGGCAGGTCCGAC
TTCACATCTTAT-AATAACACGACATTATACATCTTATTTGGTATATGATCTGGGTTGGT
TGGAACTGCTCTAAGGTTGCTAATTCGTGCAGAGTTGGGACAACCAGGGGCTTTACTTGG
AGACGATCAATTATATAATGTAATTGTGACAGCTCATGCTTTTGTTATGATTTTTTTCTT
GGTTATGCCTATGATGATTGGGGGCTTTGGAAATTGATTGGTGCCACTAATATTAGGGGC
CCCTGATATGGTATTTCCTCGATTAAATAATATAAGTTTTTGACTTCTTCCTCCCGCGCT
TTTACTCCTTCTGTCATCAGCTGCGGTGGAAAGTGGGGTAGGTACTGGGTGGACTGTATA
TCCACCTCTAGCAGGAAATTTGGCACATGCTGGTGGGTCTGTAGATCTGGCTATTTTTTC
TTTACATCTTGCTGGTGTGTCTTCTATTTTGGGGGCGGTAAACTTTATTACTACAATTAT
TAACATACGATGACAGGGGATGAAATTTGAACGCCTTTCGTTGTTTGTGTGGTCGGTGAA
AATTACGGCTATTTTACTTTTGTTATCTTTGCCTGTGCTGGCTGGTGCAATTACTATGCT
TTTAACTGATCGGAATTTTAATACTGCTTTCTTTGATCCAGCAGGAGGTGGAGATCCTAT
CTTATATCAGCATTTATTT
>sandwichensis
AACCAAGAAATTTAGTCATTCTTTTACGGTAAAAAGATAGTTAGGCAAG-AAGGACCGCT
GGATCTTTAGTGGAGGAGTAAAATCTAGACACTAAGAGTTATACCGGACGTGGTTTAATT
TATGCTGAATCTGTGATAGCTTTAAGGGAAACTGGGATTGGATACCCCATTATTTATAGT
TGTAAATTTAGTT--TGAGCTTACCAGAGTACTACGAATGTTTAAAACTCAAAGGGCTTG
GCGGTGTTTTAGACCTCTCAGGGGAACCTGTCTCATAATCGACAATCCACGTTAGACCTA
ACCTTTTATGGCGAAACAGCCTGTATACCGTCGTCGTCAGGTAACTTCTTAAAATATAGT
AGTTAGCTCGAGAATT--TT-ATTAATTGAGACGCCAGATCAAGGTGCAGCTAATAAAAA
GGTGAGGATGGGTTACAATTACATATTTGTAAATACGGCGTT-TGAAACTAAGTGTTTTA
AAGGAGGACTTGAAAGTAATTTTAGTTATATAAATAGGATGAATAAGGCTCTGAAACGTG
CAGAATTTTAATGGTCGAACAGACCAACCCTTAAAGACTTCTGCATCTTTAGGACATTCT
GGTCCAACATCGAGGTCACAAACCTTTTTTTCGATATGGGCTCTTGAAAAAGATAATGCT
GTTATCCCTACGGTAACTAATTCCTTTGATCAAAAAATT-TGGATCAAC-ACAAGTAAGA
CTTA-AGAGGAGGCTTTACTTGCTCCTCGGTTGCCCCAACCAAAGT--ATTTAATAGTTT
T-TCTTTTA-CTTAATTG--ATAAAGTCTATTAACTTCTCTAAAGCTCGATAGGGTCTTC
TTGTCTTTTAATTCAATCTAGGCTTTTTCACCTAAAAATAAAATTCTATAGAATCTAAAA
GAGACAGATGTATTCTTGTCAAACCATTCATTCCAGCCTTCAATTATAAGGCAAATGATT
ATGCTACCTTTGCACGGTCAGAGTACCGCGGCCGTT-TAAAA-CACTGGGCAGGTCCGAC
TTCACATCTTAT-AATAACACGACATTGTATATCTTATTTGGTATATGGTCTGGGTTGGT
TGGAACCGCTCTAAGGTTGCTAATTCGTGCAGAGTTGGGACAACCAGGGGCTTTACTTGG
AGATGATCAATTATATAATGTAATTGTGACAGCTCATGCTTTTGTTATGATTTTTTTCTT
GGTTATGCCTATGATGATTGGGGGCTTTGGAAATTGATTGGTGCCATTAATATTAGGGGC

TCCTGATATGGTATTTCCCCGATTAAATAATATAAGTTTTTGACTTCTCCCTCCTGCACT
TTTACTCCTTCTGTCGTCAGCTGCGGTGGAAAGTGGAGTAGGTACTGGGTGGACTGTATA
TCCACCTCTAGCAGGAAATTTGGCACATGCTGGTGGGTCTGTAGATCTGGCTATTTTTTC
TTTACACCTTGCTGGTGTGTCTTCTATTTTGGGGGCGGTAAATTTTATTACTACAATTAT
TAATATACGATGACAGGGGATGAAGTTTGAACGCCTTTCGTTGTTTGTATGGTCGGTGAA
AATTACGGCTATTTTACTTTTGTTATCTTTGCCTGTGCTGGCTGGTGCAATTACTATGCT
TTTAACCGATCGAAATTTTAATACTGCTTTTTTCGATCCGGCAGGAGGTGGAGATCCTAT
CTTATATCAGCATTTATTT
>ermineus
NNNNNNNNNNNNNNNNNNNNNNNTTACGGTAAAAAGACAGTTAAGCAGA-AAGAATCGTT
AGACCTTTAGTATAGGAGTAAAATCTAAATACTAGAAGTTATATCGGATACGGTTT-ATT
TATGCTGAATCTGTGATAGCTTTAAGGGAAACTGGGATTGGATACCCCATTATTTTTAGT
CGTAAATTAAGTT--TGAGCTTACCAGAGTACTACGAATGTTTAAAACTCAAAGAGCTTG
GCGGTGTTTTAGACCTCTCAGGGGAACCTGTCTCGTAATCGACAATCCACGTTAGACCTA
ACCCTTTATGGCGAAACAGCCTGTATACCGTCGTCGTCAGGTAACTTCTTAAAATATAGC
AGTTAGCTTGAGAATT--TT-ATTAATTAAAACGTCAGATCAAGGTGCAGCTGATAAAAG
GGTGAGGATGGGTTACAATTACACACTTGTAAATACGATATT-TGAAATAAAATGACCTG
AAGGAGGACTTGAAAGTAATTTTAGATATATAAATAGAATGAATAAGGCTCTGAAACGTG
CNNNNNNNNNNNNNTCGAACAGACCAACCCTT-AAGACTTCTGCATCTTCAGGATATTCT
GGTCCAACATCGAGGTCACAAACCTTTTTTTCGATATGGGCTCTTGAAAAAGATAATGCT
GTTATCCCTACGGTAACTAATTCCTTTGATCAAAATTTT-TGGATCAATTACATGTAGGA
TTTG-AAAGGAGGCTTTATTTACTCCTCGGTTGCCCCAACCAAAGT--ATTTAGTAGTTT
T-CCTTTTA-CTTAATTG--ATAAAGTCTACTAATTTTTCTAAAGCTCGATAGGGTCTTC
TTGTCTTTTAATAATATTTGGACTTTTTCATCCAAAGATAAAATTCTAGGCAGCCTAAAA
GAGACAGGTGTATTCTTGTCAAACCATTCATTCCAGCCTTCAATTATAAGGCAAATGATT
ATGCTACCTTTGCACGGTCAGAGTACCGCGGCCGTT-TAAAA-CACTGGGCAGGTCCGAC
TTCGTATTTAGT-TTCAACACGACATTATATATTTTATTTGGAATATGATCCGGACTAGT
CGGAACTGCCCTAAGGTTATTAATTCGTGCAGAATTAGGACAACCAGGGGCTTTGCTAGG
AGACGATCAGCTATATAATGTGATTGTAACGGCGCATGCTTTTGTTATAATTTTTTTCTT
AGTAATACCTATAATGATTGGGGGTTTTGGAAACTGGCTGGTGCCTTTAATGTTAGGGGC
TCCAGATATGGTATTCCCACGCTTAAATAATATAAGTTTTTGACTTCTTCCTCCTGCGCT
TTTGCTTCTGCTATCATCGGCTGCAGTGGAAAGTGGTGTGGGTACAGGATGGACTGTATA
TCCACCTTTAGCAGGAAACCTAGCTCATGCTGGTGGATCTGTGGATCTAGCGATTTTTTC
TTTACATCTTGCTGGGGTTTCTTCTATTTTAGGAGCCGTGAACTTTATTACTACAATTAT
TAATATACGATGGCAGGGAATGAAGTTTGAGCGTCTTTCGTTGTTTGTTTGGTCGGTAAA
GATTACTGCTATTTTGCTTCTTTTGTCGTTACCTGTGTTGGCGGGTGCAATTACTATGCT
TTTGACTGATCGGAATTTCAATACTGCTTTCTTTGATCCAGCAGGAGGTGGTGATCCTAT
CTTATATCAGCACCTATTT
>purpurascens
AACCAAGAAATTTAGTCATTCTTTTACGGTAAAAAGACAGTTAAGCAGA-AGAAATCGTT
AGACCTTTAGTATAGGAGTAAAATCTAAATACTGAAAGTTATATCAGATACGGTTT-ATT
TATGCTGAATCTGTGACAGCTTTAAGGGAAACTGGGATTGGATACCCCACTATTTTTAGT
CGTAAATTAAGTT--TGGGCTTACCAGAGTACTACGAATGTTTAAAACTCAAAGAGCTTG
GCGGTGTTTTAGACCTCTCAGGGGAACCTGTCTCGTAATCGACAATCCACGTTAGACCTA
ACCCTTTATGGCGAAACAGCCTGTATACCGTCGTCGTCAGGTAACTTCTTAAAATATAGT
AGTTAGCTTGAGAATT--TT-ATTAATTAAAACGTCAGATCAAGGTGCAGCTGATAAAAG
GGTGAGGATGGGTTACAATTACATACTTGTAAAAACGATATT-TGAAACAAAATAACCTG
AAGGAGGACTTGAAAGTAATTTTAGATATATAAATAAAATGAATAAGGCTCTGAAACGTG

CAGAATTTTAATGGTCGAACAGACCAACCCTTAAAGACTTCTGCATCTTCAGGATATTCT
GGTCCAACATCGAGGTCACAAACCTTTTTTTCGATATGGGCTCTTGAAAAAGATAATGCT
GTTATCCCTACGGTAACTAATTCCTTTGATCAAAACTTT-TGGATCAACTACATGTAAGA
TTTG-AAAGGAGGCTCTATTTACTCCTCGGTTGCCCCAACCAAAGT--GTTTAGCAGTTT
T-TCTTTTA-CTTAATTG--ATAAAATCTACTAATTTTTCTAAAGCTCGATAGGGTCTTC
TTGTCTTTTAATAATATTTGGACTTTTTCATCCAAAGATAAAATTCTAAGCAGCCTAAAA
GAGACAGGTGTATTCTTGTCAAACCATTCATTCCAGCCTTCAATTATAAGGCAAATGATT
ATGCTACCTTTGCACGGTCAGAGTACCGCGGCCGTT-TAAAA-CACTGGGCAGGTCCGAC
TTCGTATTTAGT-CTCAACACNNNNNNNNNNTATTTATTTGGAATATGATCCGGACTCGT
TGGAACTGCCCTAAGGTTATTAATTCGTGCAGAATTAGGACAACCAGGAGCTTTGCTGGG
AGACGATCAGCTATATAATGTGATTGTAACGGCCCATGCTTTTGTTATAATTTTTTTCTT
AGTAATACCTATAATGATTGGGGGGTTTGGAAACTGGCTGGTGCCTTTAATGTTAGGAGC
TCCAGACATGGTATTCCCTCGCTTAAATAATATAAGTTTTTGACTTCTTCCTCCTGCCCT
TTTGCTTCTGTTATCATCGGCTGCAGTAGAAAGTGGTGTGGGTACAGGATGAACTGTATA
TCCTCCTTTAGCAGGGAACCTGGCCCATGCTGGCGGATCTGTAGATCTAGCGATTTTTTC
TTTACATCTCGCTGGGGTTTCTTCTATTTTAGGAGCTGTGAACTTTATCACTACAATTAT
TAATATACGATGACAGGGAATAAAGTTTGAACGTCTTTCATTGTTTGTTTGGTCGGTAAA
GATTACTGCTATTTTACTCCTTTTGTCCTTGCCTGTGTTGGCGGGTGCGATTACTATGCT
TCTGACTGACCGGAATTTTAATACTGCTTTCTTTGATCCAGCAGGAGGTGGNNNNNNNNN
NNNNNNNNNNNNNNNNNNN
>arenatus
AACCAAGAAATTTAGTCATTCTTGTACGGTAAAAAGACAGTTAAGCATG-AAGGACCATT
AGATCTTCAATAAAGGAGTAAAATCTATATATTGAAAGTTATGTTAGACGTGGTTT----
TACGCTGAATCTGTGAAATCTTTAAGGGAAACTGGGATTGGATACCCCATTATTTTTAGT
CGTAAATTTAGCT--TGAGCTTACCAGAGTACTACGAATGTTTAAAACTCAAAGGGCTTG
GCGGTGTTTTAGACCTCTCAGGGGAACCTGTCTCGTAATCGACAATCCACGTTAGACCTG
ACCCTTTATTGTAAAACAGCCTGTATACCGTCGTCGTCAGGTAACTTTTTAAAATATAGA
AGTTAGCTAGAAAATT--AC-ATTAATTAAAACGTCAGATCAAGGTGCAGCTAATAAGAG
GGAGAGGATGGGTTACAATTATAGATTTATAAATACGATATT-TGAAATGAAATATTTCG
AAGGAGGACTTGAAAGTAATTTTGATTATATAAATAGAATGAATAAGGCTCTGAAACGTG
CAGAATTTTAATGGTCGAACAGACCAACCCTTAAAGACTTCTGCATCTTTAGGATATTCT
GGTCCAACATCGAGGTCACAAACCTTTTTTTCGATATGGGCTCTTGAAAAAGATAATGCT
GTTATCCCTACGGTAACTAGTTCCTTTGATCAAAATCTT-TGGATCAAC-ACAAGTTTGA
CTTA-AAAGGAGGCTTTATTTGCTCCTCGGTTGCCCCAACCAAAGT--ATTTAATAGCTT
T-TCTTCTA-TTTAATTG--ATCAAGCCTATTAATTTCTCTAAAGCTCGATAGGGTCTTC
TTGTCTTTTAATAGTAACTGGACTTTTTCATCCAAAAATAAAATTCTAAACAATCTAAAA
GAGACAGCTGTACTCTTGTCAAACCATTCATTCCAGCCTTCAATTATAAGGCAAATGATT
ATGCTACCTTTGCACGGTCAGAGTACCGCGGCCGTT-TAAAA-CACTGGGCAGGTCCGAC
TTCGCATCTAGT-CATAACACGACATTGTATATTTTATTTGGAATGTGATCTGGGTTAGT
TGGAACTGCCCTAAGGTTGTTAATTCGTGCAGAGCTAGGGCAGCCTGGTGCCTTACTTGG
GGATGATCAGCTATATAATGTAATTGTAACGGCACATGCCTTTGTTATAATTTTTTTCTT
GGTAATGCCCATGATAATTGGGGGTTTCGGAAATTGGTTAGTACCCCTAATGTTAGGGGC
TCCAGATATGGTATTTCCTCGGCTAAATAATATAAGTTTTTGACTTCTTCCTCCTGCACT
CTTGCTTTTGCTATCATCAGCTGCTGTTGAAAGAGGGGTTGGAACGGGATGAACTGTTTA
TCCGCCTTTGGCAGGAAACTTAGCACATGCTGGTGGTTCTGTAGATCTTGCAATTTTTTC
TTTACATCTTGCTGGTGTATCCTCTATTCTAGGAGCGGTAAATTTTATTACCACAATTAT
TAATATGCGATGACAAGGAATGAAATTCGAACGTCTTTCGCTATTTGTGTGGTCAGTAAA
AATTACAGCTATTTTGCTCCTTTTATCTCTACCGGTATTAGCGGGTGCAATTACTATACT

TTTAACTGATCGAAATTTTAATACTGCCTTCTTTGATCCAGCCGGAGGTGGTGATCCTAT
TTTATATCAACATCTTTTC
>pulicarius
AACCAAGAAATTTAGTCATTCTTGTACGGTAAAAAGACAGTTAAGCATA-AAGGACCATT
AGATCTTCAATAAAGGAGTAAAATCTATATATTGAAAGTTATGCTAGACGTGGTTT----
TACGCTGAATCTGTGAAATCTTTAAGGGAAACTGGGATTGGATACCCCATTATTTTTAGT
CGTAAATTTAGCT--TGAGCTTACCAGAGTACTACGAATGTTTAAAACTCAAAGGGCTTG
GCGGTGTTTTAGACCTCTCAGGGGAACCTGTCTCGTAATCGACAATCCACGTTAGACCTG
ACCCTTTATTGTAAAACAGCCTGTATACCGTCGTCGTCAGGTAACTTTTTAAAATATAGA
AGTTAGCTAGAAAATT--AC-ATTAATTAAAACGTCAGATCAAGGTGCAGCTAATAAGAG
GGAGAGGATGGGTTACAATTATAGATTTATAAATACGATATT-TGAAATGAAATATTTCG
AAGGAGGACTTGAAAGTAATTTTGATTATATAAATAGAATGAATAAGGCTCTGAAACGTG
CAGAATTTTAATGGTCGAACAGACCAACCCTTAAAGACTTCTGCATCTTTAGGATATTCT
GGTCCAACATCGAGGTCACAAACCTTTTTTTCGATATGGGCTCTTGAAAAAGATAATGCT
GTTATCCCTACGGTAACTAATTCCTTTGATCAAAATCTT-TGGATCAAC-ACAAGTTTGA
CTTA-AAAGGAGGCTTTATTTGCTCCTCGGTTGCCCCAACCAAAGT--ATTTAATAGCTT
T-TCTTTTA-TTTAATTG--ATCAAGCCTACTAATTTCTCTAAAGCTCGATAGGGTCTTC
TTGTCTTTTAATAGTAACTGGACTTTTTCATCCAAAAATAAAATTCTAAACAATCTAAAA
GAGACAGCCGTATTCTTGTCAAACCATTCATTCCAGCCTTCAATTATAAGGCAAATGATT
ATGCTACCTTTGCACGGTCAGAGTACCGCGGCCGTT-TAAAA-CACTGGGCAGGTCCGAC
TTCGCATCTAGT-CGTAACACGACATTGTATATTTTATTTGGAATGTGATCTGGGTTAGT
TGGAACTGCCCTAAGGTTGTTAATTCGTGCAGAGCTAGGGCAGCCTGGTGCCTTACTTGG
GGATGATCAGCTATATAATGTAATTGTAACGGCACATGCCTTTGTTATAATTTTTTTCTT
GGTAATGCCCATGATAATTGGGGGTTTCGGAAATTGGTTAGTACCCCTAATGTTAGGGGC
TCCAGATATGGTATTTCCTCGGCTAAATAATATAAGTTTTTGACTTCTTCCTCCTGCACT
CTTGCTTTTACTATCATCAGCTGCTGTTGAAAGAGGGGTTGGAACGGGATGAACTGTTTA
TCCGCCTTTGGCAGGAAACTTAGCACATGCTGGTGGTTCTGTAGATCTTGCAATTTTTTC
TTTACATCTTGCTGGTGTATCCTCTATTCTAGGAGCGGTAAATTTTATTACCACAATTAT
TAATATGCGATGACAAGGAATGAAATTCGAACGTCTTTCGCTATTTGTGTGGTCAGTAAA
AATTACAGCTATTTTGCTCCTTTTATCTCTACCGGTATTAGCGGGTGCAATTACTATACT
TTTAACTGATCGAAATTTTAATACTGCCTTCTTTGATCCAGCCGGAGGTGGTGATCCTAT
TTTATATCAACATCTTTTC
>caracteristicus
AACCAAGAAATTTAGTCATTCTTGTACGGTAAAAAGACAGTTAAGCGCA-AAGGACCATC
GGACGTTTAGTAGAGGAGTAAAATCTAAATACTGGAAATTATACCGGGTATGGTTT----
TATGCTGAATCTGTGAAATCTTTAAGGGAAACTGGGATTGGATACCCCATTATTTTTAGT
TGTAAACTTAGTT--TAAACTTACCAGAGTACTACGAATGTTTAAAACTCAAAGGGCTTG
GCGGTGTTTTAGACCTCTTAGGGGAACCTGTCTCATAATCGACAATCCACGTTAGACCTG
ACCCTTTATGGTAAAACAGCCTGTATACCGTCGTCGTCAGGTAACTTTTTAAAATACATA
AGTTAGCCCGAGAATT--AT-ATTAATTAAAACGTCAGATCAAGGTGCAGCTAATGAAAG
GGGGAGGATGGGTTACAATTACATATTTGTAAATACGGCATT-TGAAATAAAATGTTTTA
AAGGAGGACTTGAAAGTAATTTTGATTATATAAACAGAATGAATAAGGCTCTGAAACGTG
CAGAATTTTAATGGTCGAACAGACCAACCCTTAAAGACTTCTGCATCTTTAGGATATTCT
GGTCCAACATCGAGGTCACAAACCTTTTTTTCGATATGGGCTCTTGAAAAAGATAATGCT
GTTATCCCTACGGTAACTAATTCCTTTGATCAAAATTTT-TGGATCAAC-ACAAGTACGA
TTCA-AAAGGAGGCTTCATTTACTCCTCGGTTGCCCCAACCAAAGT--ATTTAGTAGTTT
T-TCTTTTA-CTTAATTG--GTTAAACCTACTAATTTCTCTAAAGCTCGATAGGGTCTTC
TTGTCTTTTAATTACACCTGGACTTTTTCATCCAAAGATAAAATTCTAAATAATCTAAAA

GAGACAGCTGTATTCTTGTCAAACCATTCATTCCAGCCTTCAATTATAAGGCAAATGATT
ATGCTACCTTTGCACGGTCAGAGTACCGCGGCCGTT-TAAAA-CACTGGGCAGGTCCGAC
TTCGTATCTAAC-ACTAACACGACATTGTATATTCTATTTGGTATATGGTCTGGGCTAGT
TGGAACTGCTTTAAGGCTGTTAATTCGTGCGGAACTTGGGCAGCCAGGTGCTTTACTCGG
AGACGATCAGCTATATAATGTAATTGTAACAGCGCATGCTTTTGTTATAATTTTTTTCTT
GGTAATGCCTATAATAATTGGAGGCTTTGGAAACTGATTGGTGCCATTAATGCTAGGGGC
TCCAGATATGGTATTTCCTCGTCTAAATAATATAAGTTTTTGGCTTCTTCCTCCTGCACT
TTTACTTTTGTTATCTTCAGCTGCTGTTGAAAGTGGGGTTGGGACGGGATGAACTGTTTA
TCCGCCTTTAGCAGGAAATTTGGCTCACGCCGGTGGTTCTGTAGATCTTGCGATTTTCTC
TTTACATCTTGCCGGTGTATCTTCTATTTTAGGAGCTGTAAATTTTATTACGACAATTAT
TAATATACGGTGACAAGGAATAAAATTTGAACGTCTTTCATTGTTTGTGTGATCAGTAAA
AATTACGGCTATTCTACTTCTTTTATCTCTACCGGTATTAGCAGGTGCAATTACTATGCT
TTTAACTGATCGAAATTTTAATACTGCTTTCTTTGACCCTGCCGGAGGAGGTGATCCAAT
TTTGTATCAGCATCTTTTT
>bullatus
AACCAAGAAATTTAGTCATT-TTTTACGGTAAAAAGACAGTTAGGCAGA-AGGAATTATT
AGATCTTTAGTAAAGGAGTAAAATCTAAATACTAAAAGTTATACCGAATATAATT--ATT
TGTGTTGAATCTGTGACATCTTTAAGGGAAACTGGGATTGGATACCCCACTATTTTTAGT
TATAAATTTAGTT--TATGCTTACCAGAGTACTACGAATGTTTAAAACTCAAAGGGCTTG
GCGGTGTTTTAGACCTCTCAGGGGAACCTGTCTCGTAATCGACAATCCACGTTAGACCTA
ACCTTTTATGGTAATTCAGCCTGTATACCGTCGTCGCCAGGTAACTTCTTAAAATATAGT
AGTTAGCTTGATAATT--TA-ATTAATTAGAACGTCAGATCAAGGTGCAGCTAATAAAAA
GGTGAGGATGGGTTACAATTATAGATTTGTAAATACGGTGCT-TGAAATGGAGCATTTCA
AAGGAGGACTTGAAAGTAATTTTGATTATATAAGCAAAATGAATAGGGCTCTGAAACGTG
CNNNNNNNNNNTGGTCGAACAGACCAACCCTT-AAGACTTCTGCATCTTTAGGATATTCT
GGTCCAACATCGAGGTCACAAACCTTTTTTTCGATATGAGCTCTTAAAAAAGATAATGCT
GTTATCCCTACGGTAACTAATTCCTTTAATCAAAATTCT-TGGATCAAA-ACAAGCAAGA
CTTA-AAAGGAGGCTCTATATGCTCCTCGGTTGCCCCAACCAAAGT--ATTTAATAGTTT
TATCTTTTA-CTTCATTG--AT-AAATCTATCAATTTTTCTGAAGCTCGATAGGGTCTTC
TTGTCTTTTATTAGAATTTGGACTTTTTCATCCAGAAATAAAATTCTAATTAATCTAAAA
GAGACAGCTGTATTCTTGTCAAACCATTCATTCCAGCCTTCAATTATAAGGCAAATGATT
ATGCTACCTTTGCACGGTCAGAGTACCGCGGCCGTT-TAAAA-CACTGGGCAGGTCCGAC
TTCGTATTTAGA-ACCTACACGACATTGTATATTTTATTTGGAATATGATCAGGTCTGGT
TGGAACGGCCTTAAGGCTCTTGATTCGTGCGGAATTAGGACAACCTGGTGCTTTGCTCGG
AGATGATCAGTTATATAATGTGATTGTAACGGCTCATGCCTTTGTTATAATTTTTTTCTT
AGTAATACCTATAATGATTGGCGGTTTTGGAAACTGGCTGGTACCTTTAATATTGGGAGC
TCCAGATATGGTATTTCCTCGATTGAATAATATGAGTTTTTGGCTCCTTCCTCCTGCGCT
TTTACTTCTTCTGTCTTCGGCTGCAGTAGAAAGCGGGGTAGGTACAGGTTGAACTGTATA
TCCTCCCTTGGCAGGAAACCTGGCACATGCTGGTGGTTCTGTAGATCTGGCAATTTTTTC
TTTACATCTTGCTGGTGTTTCTTCTATTTTAGGTGCAGTAAATTTTATTACTACAATTAT
TAACATACGATGACAAGGGATGAAATTTGAGCGTCTTTCTTTGTTTGTATGATCGGTAAA
GATTACAGCAATTTTGCTTCTTTTATCTCTACCTGTCTTAGCGGGTGCTATTACTATGCT
TTTGACTGATCGGAACTTTAATACTGCTTTCTTTGATCCAGCAGGAGGTGGTGATCCTAT
TTTGTATCAGCATCTATTT
>sponsalis
AACCAAGAAATTTAGTCATTCTTTCACGGTAAAAAGACAGTTAGACAGA-AAAAATCGTT
GGACCTTTGGTATAGGAGTAAAATCTAAATACCGAAAGTTATATCAGGTGCGATTT-AGT
CATGTTGAATCTGTGATAGCTTTAAGGGAAACTGGGATTGGATACCCCATTATTTTTAGT

CATAAATATAGTA--TAGACTTACCGGAGTACTACGAATGTTTAAAACTCAAAGGGCTTG
GCGGTGTTTTAGACCTCTCAGGGGAACCTGTCTCGTAATCGACAATCCACGCTAGACCTG
ACCTTTTATGGTAATACAGTCTGTATACCGTCGTCGTCAGGTAACTTCTTAAAATATAGT
AGTTAGCATGAAAATT--TTGATTAATTAAGACGTCAGATCAAGGTGCAGCCAATAAAAA
GGTGAGGATGGGTTACAATTATATAATTATAACTACGGTACT-TGAAAAGAAGTGTTTCA
AAGGAGGACTTGAAAGTAATTTTAATTATATAAATAAAATGAATTAGGCTCTGAAACGTG
CAGAATTTTAATGGTCGAACAGACCAACCCTTAAAGACTTCTGCATCTTCAGGATATTCT
GGTCCAACATCGAGGTCACAAACCTTTTTTTCGATATGGGCTCTTGAAAAAGATTATGCT
GTTATCCCTACGGTAACTAATTCCTTTGATCAAAATTAC-TGGATCAAC-ACAAGTAAGA
TTTG-AAAGGAGGCTTTGTCTGCTCCTCGGTTGCCCCAACCAAAGT--GTTTAGTAACCT
T-TCTTTTA-CTTAATTG--ATAGAGTTTACTAATTTCTCTAAAGCTCGATAGGGTCTTC
TTGTCTATTAATTATATCTGGACTTTTTCATCCAAAAATAAAGTTCTAAATAATCTAAAG
GAGACAGGGGTATTCTTGTCAAACCATTCATTCCAGCCTTCAATTATAAGGCAAATGATT
ATGCTACCTTTGCACGGTCAGAGTACCGCGGCCGTT-TAAAA-CACTGGGCAGGTCCGAC
TTCGTATTTATT-ATCTACACGACATTATACATTTTGTTTGGAATATGATCGGGACTGGT
TGGAACTGCTCTAAGGTTGTTAATTCGTGCAGAGCTAGGGCAACCTGGTGCTCTTCTTGG
AGATGATCAATTGTACAACGTAATTGTAACAGCCCATGCTTTTGTTATAATTTTTTTCTT
AGTAATACCTATGATGATTGGGGGATTTGGAAATTGATTAGTACCTTTAATGTTGGGAGC
TCCCGATATGGTGTTTCCTCGTTTAAATAATATGAGTTTTTGACTTCTCCCTCCTGCACT
TTTGCTTCTCTTGTCGTCAGCTGCAGTGGAAAGTGGCGTTGGTACAGGATGAACAGTATA
TCCACCTTTGTCAGGAAACCTGGCCCATGCTGGTGGGTCTGTAGATTTAGCAATTTTTTC
TCTACATCTTGCTGGTGTGTCGTCTATTTTAGGTGCAGTAAATTTTATTACTACAATTAT
TAACATACGATGACAAGGAATAAAGTTTGAACGTCTTTCATTGTTTGTCTGATCGGTAAA
AATTACAGCTATTTTACTTCTTTTATCTCTACCTGTGTTGGCCGGTGCTATTACTATGCT
TTTAACAGATCGGAATTTTAATACTGCCTTCTTTGATCCAGCAGGAGGTGGTGATCCAAT
TTTATATCAACACTTGTTT
>nux
AACCAAGAAATTTAGTCATTCTTTCACGGTAAAAAGACAGTTAGACAGA-AAAAATCGTT
GGACCTTTGGTATAGGAGTAAAATCTAAATACCAAAAGTTATATCAGGTGCGATTT-AGT
CATGTTGAATCTGTGATAGCTTTAAGGGAAACTGGGATTGGATACCCCACTATTTTTAGT
CATAAATATAGTA--TAGACTTACCGGAGTACTACGAATGTTTAAAACTCAAAGGGCTTG
GCGGTGTTTTAGACCTCTCAGGGGAACCTGTCTCATAATCGACAATCCACGCTAGACCTG
ACCTTTTATGGTAAAACAGTCTGTATACCGTCGTCGTCAGGTAACTTCTTAAAATATAGT
AGTTAGCATGAAAATT--TTGATTAATTAAGACGTCAGATCAAGGTGCAGCCAATAAAAA
GGTGAGGATGGGTTACAATTATATAGTTATAAATACGGTACT-TGAAACGAAGTGTTTCA
AAGGAGGACTTGAAAGTAATTTTAATTATATAAATAAAATGAATTAGGCTCTGAAACGTG
CAGAATTTTAATGGTCGAACAGACCAACCCTTAAAGACTTCTGCATCTTCAGGATATTCT
GGTCCAACATCGAGGTCACAAACCTTTTTTTCGATATGGGCTCTTGAAAAAGATTATGCT
GTTATCCCTACGGTAACTAATTCCTTTGATCAAAATTAT-TGGATCAAC-ACAAGTAAGA
TTTG-AAAGGAGGCTTTGCCTGCTCCTCGGTTGCCCCAACCAAAGT--GTTTAGTAACCT
T-TCTTTTA-CTTAATTG--ACAGAGTTTACTAATTTCTCTAAAGCTCGATAGGGTCTTC
TTGTCTATTAATTATATCTGGACTTTTTCATCCAAAAATAAAGTTCTAAATAATCTAAAG
GAGACAGGGGTATTCTTGTCAAACCATTCATTCCAGCCTTCAATTATAAGGCAAATGATT
ATGCTACCTTTGCACGGTCAGAGTACCGCGGCCGTT-TAAAA-CACTGGGCAGGTCCGAC
TTCGTATTTATT-ATCTGCACNNNNNNATACATTTTGTTTGGAATATGATCGGGACTGGT
TGGAACTGCTCTAAGGCTGTTGATTCGTGCAGAGCTAGGGCAACCTGGTGCTCTTCTTGG
AGATGATCAGTTGTATAACGTAATTGTAACAGCCCATGCTTTTGTTATAATTTTTTTCTT
AGTAATACCTATGATGATTGGGGGATTTGGAAATTGATTAGTACCCTTAATGTTGGGAGC

TCCCGATATGGTGTTTCCTCGTTTAAATAATATGAGTTTTTGACTTCTCCCTCCTGCACT
TTTGCTTCTCTTGTCGTCAGCTGCAGTGGAAAGTGGCGTTGGTACAGGATGAACAGTATA
TCCACCTTTGTCAGGAAACCTGGCTCATGCTGGTGGATCTGTAGATTTAGCAATTTTTTC
TCTACATCTTGCTGGTGTATCATCTATTTTAGGTGCAGTAAATTTTATTACTACAATTAT
CAACATACGATGACAAGGAATAAAGTTTGAACGTCTTTCACTGTTTGTCTGATCGGTAAA
AATTACAGCTATTTTACTTCTTTTATCTTTACCTGTGTTGGCTGGTGCTATTACTATGCT
CTTGACAGATCGGAATTTTAATACTGCTTTCTTTGATCCAGCGGGAGGTGGTGATCCAAT
TTTATATCAACACTTGTTT
>musicus
AACCAAGAAATTTAGTCATTCTTTCACGGTAAAAAGACAGTTAGACAGA-AAAAATCGTT
GGACCTTTGGTATAGGAGTAAAATCTAAATACCAAAAGCTATATCAGATGCGGTTT-GGT
TATGTTGAATCTGTGATAACTCTAAGGGAAACTGGGATTGGATACCCCACTATTTTTAGT
CATAAATTTAGTA--TGGACTTACCAGAGTACTACGAATGTTTAAAACTCAAAGGGCTTG
GCGGTGTTTTAGACCTCTCAGGGGAACCTGTCTCATAATCGACAATCCACGTTAGACCTA
ACCTTTTATGGTAATACAGTCTGTATACCGTCGTCGTCAGGTAACTTCTTAAAATATAGT
AGTTAGCATGAAAATC--TTGATTAATTAAGACGTCAGATCAAGGTGCAGCCAATAAAAA
GGTGAGGATGGGTTACAATTATATAATTATAAATACGGCACT-TGAAATGAAGTGTCTCA
AAGGAGGACTTGAAAGTAATTTTAATTATATAAATAAAATGAATTAGGCTCTGAAACGTG
CAGAATTTTAATGGTCGAACAGACCAACCCTTAAAGACTTCTGCATCTTTAGGATATTCT
GGTCCAACATCGAGGTCACAAACCTTTTTTTCGATATGGGCTCTTGAAAAAGATTATGCT
GTTATCCCTACGGTAACTAATTCCTTTGATCAAAATTAT-TGGATCGAC-ACAAGTAAGA
TTTA-AAAGGAGGCTTTGTTTGCTCCTCGGTTGCCCCAACCAAAGT--GTTTAGCAATCT
T-TCTTTTA-CTTAATTG--GTAAAGTTTACTAATTTCTCTAAAGCTCGATAGGGTCTTC
TTGTCTATTAATTGTATCTGGACTTTTTCATCCAAAAATAAAGTTCTAAATAATCTAAAA
GAGACAGGGGTATTCTTGTCAAACCATTCATTCCAGCCTTCAATTATAAGGCAAATGATT
ATGCTACCTTTGCACGGTCAGAGTACCGCGGCCGTT-TAAAA-CACTGGGCAGGTCCGAC
TTCGTATTTATT-ATTTGCACGACATTATACATTTTGTTTGGAATATGATCAGGATTGGT
TGGAACTGCTCTAAGGTTGTTAATTCGTGCAGAGTTAGGGCAGCCTGGTGCTCTTCTCGG
AGATGATCAGCTATATAACGTAATTGTAACAGCCCATGCCTTTGTTATAATTTTTTTCTT
AGTAATACCTATGATGATTGGGGGATTTGGAAATTGATTAGTGCCTCTGATGTTGGGAGC
TCCTGATATGGTGTTTCCTCGTTTAAATAATATAAGTTTTTGACTTCTTCCTCCTGCACT
CTTGCTTCTTCTGTCGTCAGCTGCAGTGGAAAGTGGTGTTGGTACAGGATGGACGGTATA
TCCACCTTTGTCGGGGAATCTGGCTCATGCTGGTGGATCTGTAGATTTAGCAATTTTTTC
CTTGCATCTTGCTGGTGTGTCATCTATTTTAGGTGCAGTAAATTTTATTACTACAATCAT
CAATATACGATGGCAAGGAATAAAATTTGAACGTCTTTCATTATTTGTTTGATCGGTAAA
AATTACAGCTATCTTACTTCTTTTATCATTACCCGTGTTAGCCGGTGCAATCACTATGCT
TTTAACAGATCGGAATTTTAATACTGCCTTCTTTGATCCAGCAGGAGGANNNNNNNNNNN
NNNNNNNNNNNNNNNNNNN
>thalassiarchus
AACCAAGAAATTTAGTCATTCTTTTATGGTAAAAAGACAGTTAGGCAGA-GAAGGCCATA
AGATCTCTAATATAGGGGTAAAATCTAAATACTAGAAGTTATCTCAAGCATGGCTT-ATT
TGTGTTGAGTCTGTGATAGCTTTAAGGGAAACTGGGATTGGATACCCCATTATTTTTAGT
TGTAAATTTAGTT--TGAACTTACCAGAGCACTACGAACGTTTAAAACTCAAAGGGCTTG
GCGGTGTTTTAGACCTCTCAGGGGAACCTGTCTCGTAATCGATAGTCCACGTTAAACCTG
ATCTCTTGTAGTAATGCAGCCTGTATACCGTCGTCGTCAGGTAACTTCTTAAAATATAGG
AGTTAGCAGGATAATC--AG-ATTAGTTAAGACGTCAGATCAAGGTGCAGCTAATAAAGA
GGTGAAGATGGGTTACACTTATACATTTATAAATACGATACT-TGAAATAAAGTACTTTG
AAGGAGGACTTGAAAGTAATCTTAATTATATAAATAAGATGAATATGGCTCTGAAACGTG

CAGAATTTTAATGGTCGAACAGACCAACCCTTGAAGACTTCTGCATCTTCAGGACATTCT
GGTCCAACATCGAGGTCACAAACCTTTTTTTCGATATGGGCTCTTAAAAAAGATAATGCT
GTTATCCCTACGGTAACTAATTCCTTTGCTCAAAAATTT-TGGATCAAC-ATAAGTAAAA
CTTA-AAAGGAGGCTTTCTTTACTCCTCGGTTGCCCCAACCAAAGT--ATTTAGTAGCTT
--CATTTTA-TTTAACTA--TTAAAGTCTACTAGTTTCTCTAAAGCTCGATAGGGTCTTC
TTGTCTTTTAATTCTATCTGGACTTTTTCATCCAAAAATGAAATTCTAAATAATCTAAAA
GAGACAGATGTATTCTTGTCAAACCATTCATTCCAGCCTTCAATTATAAGGCAAATGATT
ATGCTACCTTTGCACGGTCAGAGTACCGCGGCCGTT-TAAAA-CACTGGGCAGGTCCGAC
TTCGTATTTAAT-ATTGACATGACATTGTATATTTTATTTGGTATATGATCTGGGTTAGT
TGGAACGGCCTTAAGGTTATTAATTCGTGCAGAACTAGGGCAACCCGGCGCTTTACTTGG
AGATGATCAGTTGTATAATGTAATTGTAACAGCTCATGCTTTTGTTATAATTTTTTTCTT
AGTAATACCTATAATAATTGGTGGCTTTGGAAACTGATTGGTACCGTTGATACTAGGGGC
TCCAGACATAGTTTTTCCACGTTTAAATAACATAAGTTTTTGGCTTTTGCCTCCTGCTCT
TTTACTTCTTTTGTCATCAGCTGCTGTAGAAAGTGGAGTAGGAACAGGATGGACTGTATA
TCCACCTTTAGCTGGAAACTTAGCACATGCTGGTGGTTCTGTAGATCTAGCAATTTTCTC
TTTGCATCTTGCTGGGGCTTCCTCTATTTTAGGAGCAGTAAATTTTATTACCACAATTAT
TAATATACGATGGCAAGGAATGAAATTTGAACGTCTTTCATTGTTTGTATGGTCTGTGAA
AATTACAGCTATTTTACTCCTTTTATCTTTGCCTGTTTTGGCTGGAGCTATTACTATGCT
TTTAACTGATCGGAATTTTAATACTGCCTTCTTTGATCCTGCTGGAGGCGGGGATCCTAT
TTTGTACCAACACTTATTT
>betulinus
AACCAAGAGATTTAGTCATTCTTTTATGGTAAAAAGACAGTTAGGCAAG-AAGGACCATT
AGATCTTTAGTATAGGGGTAAAATCTATATACTAAAAGTTATACCGGGCGTGGTTT-ATT
TGTGCTGAATCTGTGATAGCTTTAAGGGAAACTGGGATTGGATACCCCATTATTTCTAGT
TGTAAATTTAGTT--TAAGCTTACCAGAGTACTACGAATGTTTAAAACTCAAAGGGCTTG
GCGGTGTTTTAGACCTCTTAGGGGAACCTGTCTCGTAATCGACAATCCACGTTAGACCTA
ACCTTTTGTGGCATGACAGCCTGTATACCGTCGTCGTCAGGTAACTTCTTAAAATATAGT
AGTTAGCCCGAGAACT--TT-ATTAGTTAAAACGTCAGATCAAGGTGCAGCTAATAAAAG
GGTGAGGATGGGTTACAATTATAGATTTATAAGTACGGGATT-TGAAATGGAGTGTTTGA
AAGGAGGACTTAAAAGTAATTTTGATTATATAAGCAGAATGAATATGGCTCTGAAACGTG
CAGAATTTTAATGGTCGAACAGACCAACCCTTAAAGACGTCTGCATCTTTAGGACATTCT
GGTCCAACATCGAGGTCACAAACCTTTTTTTCGATATGGGCTCTTGAAAAAGATAATGCT
GTTATCCCTACGGTAACTAATTCCTTTGATCAGAAACTC-TGGATCAAC-ACAAGTAAGG
CTAT-AAAGGAGGCTTTTTATGCTCCTCGGTTGCCCCAACCAAAGT--TTTTAGTAGACT
TGTCTTTTA-CTAAATTA--ACAAAATCTACTAGCTCCTCTAAAGCTCGATAGGGTCTTC
TTGTCTTTTAATTATATCTGAACTTTTTCATTCAGAGATAAAATTCTAAGTAATCTAAAA
GAGACAGCTGTATTCTTGTCAAACCATTCATTCCAGCCTTCAATTATAAGGCAAATGATT
ATGCTACCTTTGCACGGTCAGAGTACCGCGGCCGTT-TAAAA-CACTGGGCAGGCCCGAC
TTCGTATCTAAC-CTCCACACGACATTGTATATTTTATTCGGGATATGGTCTGGTTTAGT
CGGAACTGCATTAAGATTGTTGATTCGTGCAGAATTAGGGCAGCCTGGTGCTTTACTGGG
GGACGATCAGCTATATAATGTAATTGTAACAGCTCATGCTTTTGTTATAATCTTTTTCTT
GGTAATGCCTATAATGATTGGGGGTTTCGGAAATTGATTGGTGCCTTTGATATTAGGGGC
TCCAGATATAGTGTTTCCCCGCTTAAATAATATAAGTTTTTGACTTCTTCCTCCTGCACT
TTTACTTTTGTTATCGTCGGCCGCGGTAGAGAGTGGAGTGGGTACGGGGTGAACTGTGTA
TCCGCCTTTGGCAGGAAACTTGGCACATGCTGGTGGATCTGTAGATCTAGCAATTTTCTC
TTTACATCTTGCAGGAGTATCTTCTATCTTAGGGGCAGTAAATTTTATTACGACAATTAT
TAATATACGATGACAGGGAATAAAATTTGAGCGTCTTTCTTTGTTTGTCTGATCGGTGAA
GATTACGGCTATTTTACTTCTTTTGTCTTTACCTGTATTGGCTGGGGCAATTACTATGCT

TTTGACTGATCGAAACTTTAATACTGCATTCTTTGATCCAGCAGGAGGTGGTGACCCTAT
TTTGTACCAGCATTTGTTT
>figulinus
AACCAAGAAATTTAGTCATTCTTTTACGGTAAAAAGACAGTTAGGCAAA-AGGAATCATT
GGATCTTTAGTATAGGAGTAAAATCTAGATACTAAAAGTTATACCTGGCATGATTT-GTT
TGGGCTGAATCTGTGATAGCTTTAAGGGAAACTGGGATTGGATACCCCATTATTTCTAGT
TGTAAATTTAGTT--TGGGCTTACCGGAGTACTACGAATGTTTAAAACTCAAAGGGCTTG
GCGGTGTTTTAGACCTCTTAGGGGAACCTGTCTCGTAATCGACAATCCACGTTAAACCTA
ACCCTTTGTGGCATGACAGCCTGTATACCGTCGTCGTCAGGTAACTTCTTAAAATATAGT
AGTTAGCTCGAGAACA--TT-GTTGGTTAAAACGTCAGATCAAGGTGCAGCTAATAAAAG
GGTGAGGATGGGTTACAATTATATATTTATAAGTATGACATT-TGAAATGAAATGTCTTG
AAGGAGGACTTAAAAGTAATTTTGATTATATAAATAAAATGAATATGGCTCTGAAACGTG
CAGAATTTTAATGGTCGAACAGACCAACCCTCAAAGACGTCTGCATCTTTAGGACATTCT
GGTCCAACATCGAGGTCACAAACCTTTTTTTCGATATGGGCTCTTGAAAAAGATAATGCT
GTTATCCCTACGGTAACTAATTCCTTTGATCAAAAACTC-TGGATCAAC-ACGAGTAAGA
CTAT-AAAGGAGGCTTTATCTACTCCTTGGTTGCCCCAACCAAAAT--TTTTAATAGACT
CGTCTTTTA-CTTAATTG--ACAAAATCTACTAATTCTTCTAAAGCTCGATAGGGTCTTC
TTGTCTTTTAATTATATCTGAACTTTTTCATTCAGAAATAAAATTCTAAACAATCTAAAA
GAGACAGCTGTATTCTTGTCAAACCATTCATTCCAGCCTTCAATTATAAGGCAAATGATT
ATGCTACCTTTGCACGGTCAGAGTACCGCGGCCGTT-TAAAA-CACTGGGCAGGCCCGAC
TTCGTATCTAGC-ATCCACACGACATTGTATATTTTATTTGGGATGTGGTCTGGCTTAGT
TGGAACTGCATTGAGGTTGCTAATTCGTGCAGAGTTAGGGCAGCCTGGTGCTTTACTTGG
AGACGATCAATTGTATAATGTGATCGTAACGGCGCATGCTTTTGTTATAATTTTTTTCTT
AGTGATGCCTATGATGATTGGGGGCTTTGGGAATTGATTGGTACCATTGATGTTAGGGGC
TCCAGATATAGTATTTCCTCGGTTAAATAACATAAGTTTTTGGCTTCTTCCTCCTGCACT
TTTGCTTCTCTTGTCGTCGGCCGCGGTGGAGAGCGGAGTGGGTACAGGGTGAACTGTATA
TCCACCTTTGGCTGGTAATTTGGCGCATGCTGGTGGGTCTGTAGATCTTGCGATTTTTTC
TTTACATCTTGCTGGAGTATCTTCTATTTTGGGGGCGGTAAATTTTATTACTACGATTAT
TAATATACGATGACAGGGAATAAAGTTTGAACGTCTTTCGTTGTTTGTCTGATCAGTGAA
GATTACGGCTATTTTGCTTTTATTGTCTTTACCTGTATTGGCGGGAGCAATTACTATGCT
TTTGACTGRTCGAAATTTTAATACTGCTTTTTTTGATCCAGCAGGAGGTGGTGATCCTAT
CTTGTATCAGCACTTATTT
>cordigera
AACCAAGAAATCTAGTCATTCTTTTACGGTAAAAAGACAGTTAAACAAATAGGAGCCATT
GGACCTTTAGTGTAGGAGTAAAATCCGAATACTAAAAGTTACACCCGGTATGGTTT-GTT
TTCGTTGAATCTGTGATAGCTTTAAGGGAAACTGGGATTGGATACCCCATTATTTTTAGC
TGTAAATTTAGTT--TAAGCTTACCCGAGTACTACGAATGTTTAAAACTCAAAGGACTTG
GCGGTGTTTTAGACCTCTTAGGGGAACCTGTCTCGTAATCGACAATCCGCGTTAAACCTG
ACCTTTTTTAGTAG-ACAGCCTGTATACCGTCGTCGTCAGGTAACTTCTTAAAATATAAT
GGTTAGCTTGAGAATT--AG-ATGAATTAAAACGTCAGATCAAGGTGCAGCTGATAAAAA
GGTGAGGATGGGTTACAATTATATATTTGTAATTACGGTGCT-TGAAATAAAGTATATTG
AAGGAGGACTTAAAAGTAATTCTAATTATATAGATAGAATGAATAGGGCTCTGAAACGTG
TAGAATTTTAATGGTCGAACAGACCAACCCTTAAAGACTTCTGCATCTTTAGGATATTCT
GGTCCAACATCGAGGTCACAAACTTTTTTTTCGATATGAGCTCTTAAAAAAAATAATGCT
GTTATCCCTACGGTAACTAATTTCTTTGATCAAAAACTT-TGGATCAAC-ACGAGAAGGT
TTTA-AAAGGAGGTTTTGCTTACTCCTCGGTTGCCCCAACCAAAGT--ATTTAATAGTTT
T-ATTTTT--CTTAATTG--ATAAGGCCTATTAACTCTTCTAAAGCTCGATAGGGTCTTC
TTGTCTTTTAATAATATCTAGACTTTTTCATCCAAAGATAAAATTCTAAGCAGTCTAAAA

GAGACAGACGTATTCTTGTCAAACCATTCATTCCAGCCTTCAATTATAAGGCAAGTGATT
ATGCTACCTTTGCACGGTCAGAGTACCGCGGCCGTT-TAAAA-CACTGGGCAGGTCCGAC
TTCGTATCTGGT-ATTAACACGACATTATATATTCTATTTGGAATGTGATCTGGGTTGGT
TGGAACTGCTCTTAGACTATTAATTCGTGCAGAGTTGGGGCAGCCAGGTGCTTTGCTTGG
GGATGATCAGCTATATAATGTGATTGTGACGGCTCATGCTTTTGTTATAATTTTCTTTCT
GGTGATGCCTATAATAATTGGGGGCTTCGGGAACTGATTAGTGCCTTTGATACTAGGGGC
TCCGGATATGGTGTTTCCGCGTTTAAATAATATAAGTTTCTGGCTTTTACCGCCTGCACT
TTTACTTTTATTGTCGTCGGCCGCAGTGGAGAGAGGAGTGGGTACTGGATGAACTGTGTA
TCCACCTTTAGCAGGAAATTTAGCTCATGCTGGAGGATCTGTAGATCTGGCAATTTTTTC
TTTGCATCTTGCTGGTGTTTCTTCTATTTTGGGGGCAGTAAATTTTATTACTACCATTAT
TAATATACGGTGACAAGGAATGAAGTTTGAACGTCTTTCATTGTTTGTGTGGTCTGTAAA
AATTACAGCTATTTTGCTTCTTTTATCTTTACCTGTATTAGCTGGGGCAATTACTATGCT
TTTGACTGATCGAAATTTTAATACTGCTTTTTTTGACCCAGCAGGAGGTGGTGATCCAAT
TTTATATCAGCACTTGTTT
>nobilis
AACCAAGAAATTTAGTCATTCTTTCACGGTAAAAAGACAGTTAGGCGAA-AGGAATCATT
GGACCTTTAGTACAGGAGTAAAATCTGAATACTAAAAGTTATACCTAACATGGTTC-ATT
TTTGTTGAATCTGTGATAGCTTTAAGGGAAACTGGGATTGGATACCCCATTATTTCTAGT
CGTAAATTTAGTT--TAAATTTACCGGAGTACTACGAATGTTTAAAACTCAAAGGACTTG
GCGGTGTTTTAGACCTCTCAGGGGAACCTGTCTCGTAATCGACAATCCGCGTTAAACCTA
ACCTTTTTTGGTAATACAGCCTGTATACCGTCGTCGCCAGGTAACTTCTTAAAGTATAGT
AGTTAGCTTGAGAATT--TA-GTAAATTAAAACGTCAGATCAAGGTGCAGCTAATAAAAA
GGTGAGGATGGGTTACAATTATATATTTATAAATACGATACT-TGAAATAAAGCGTTTTG
AAGGAGGACTTGAAAGTAATTTTAATTATATAAATAAAATGAATAAGGCTCTGAAACGTG
CAGAATTTTAATGGTCGAACAGACCAACCTTTAAAGACTTCTGCATCTTTGGGGTATTCT
GGTCCAACATCGAGGTCACAAACTTTTTTTTCGATATGAGCTCTTGAAAAAAATGATGCT
GTTATCCCTACGGTAACTAATTTCTTTGATCAAAAATTT-TGGATCAGC-ACAAGCAAGA
TTTA-AAAGGAAGCTTTTTCTGCTCCTCGGTTGCCCCAACCAAAGT--ATTTAATAGTTT
T-ACTTTTA-CTTAATTA--GTAAAATCTATTAACTTCTCTAAAGCTCGATAGGGTCTTC
TTGTCTTTTAATCATATCTGGACTTTTTCATCCAAAAATAAAATTCTAAGCAATCTAAAA
GAGACAGGCGTATTCTTGTCAAACCATTCATTCCAGCCTTCAATTATAAGGCAAATGATT
ATGCTACCTTTGCACGGTCAGAGTACCGCGGCCGTT-TAAAA-CACTGGGCAGGTCCGAC
TTCGTATTTGAT-ATCAACACGACATTGTATATTTTATTTGGGATGTGATCTGGGTTAGT
TGGGACTGCCCTCAGATTACTGATTCGTGCAGAGTTAGGACAGCCAGGTGCCTTGCTTGG
AGATGATCAGTTGTATAATGTAATTGTAACAGCACACGCTTTTGTTATGATTTTTTTTCT
AGTAATACCTATAATGATTGGGGGCTTTGGGAATTGATTGGTACCTTTAATATTAGGGGC
CCCGGATATGGTGTTTCCGCGTTTAAATAATATAAGTTTTTGACTTTTACCGCCTGCGCT
CTTACTTTTGTTGTCCTCAGCTGCGGTAGAAAGCGGGGTGGGTACAGGATGGACTGTGTA
TCCGCCTCTAGCAGGGAATTTAGCGCATGCTGGAGGATCTGTAGATCTGGCAATTTTTTC
TTTGCATCTTGCTGGGGTTTCTTCTATTTTAGGAGCAGTGAATTTCATTACTACAATTAT
TAATATACGATGACAAGGAATGAAGTTTGAGCGTCTTTCATTATTTGTATGATCTGTAAA
AATTACGGCTATTTTACTTCTTTTGTCTTTACCTGTATTAGCTGGAGCAATTACTATACT
TTTAACTGATCGAAATTTTAATACCGCTTTCTTTGATCCAGCAGGGGGTGGTGATCCAAT
TTTATACCAACATTTATTT
>cuvieri
AACCAAGAAATTTAGTCATTCTTTTACGGTAAAAAGACAGTTAGGCAAA-AGCGATTATT
AGATCTTTAGTAGAGGGGTAAAATCTATATACTAAAAGTTATTCCTGAAATGATTT-GCT
-AAGCTGAATCTGTGACAACTTTAGGGGAAACTGGGATTGGATACCCCATTATTTTTAGT

CGTAAATTTAGTT--TA-ACTTACCTGAGTACTACGAATGTTTAAAACTCAAAGGGCTTG
GCGGTGTTTTAAACCTTTCAGGGGAACCTGTCTCGTAATCGACAATCCACGTTATACCTA
ACCTCTTATAGTGAAACAGCCTGTATACCGTCGTCGCCAGGTAACTTCTAAAAATAGAGT
AGTTAGCTTGAAAATC--TA-ATTGATTAGGACGTCAGATCAAGGTGCAGCTAATGAAGA
GGTGAGGATAGGTTACAATTACATATTTGTAAATACGGCACT-TGAAATAAG--TGCTTA
AAGGAGGACTTGAAAGTAATCGTAATTATATAAATAAAATGAATAAGGCTCTGAAACGTG
CAGAATTTTAATGGTCGAACAGACCAACCCTTAAAGACTTCTGCATCTTTAGGACATTCT
GGTCCAACATCGAGGTCACAAACCTTCTTTTCGATATGATCTCTTAAAAAAGATAATGCT
GTTATCCCTACGGTAACTAATTCCTTCAATCAAAAATTT-TGGATCAAT-ACAAGTCAGA
TTTT-AAAGGAGGCTTTAAATGCTCCTCGGTTGCCCCAACCAAAGT--CTTTAATAGTTT
T-TCTTTTA-GTTAATTG--ACCAAATCTATTAATTTTCCTAAAGCTCGATAGGGTCTTC
TTGTCTTTTAATATGATCTGGACTTTTTCATCCAGAAATAAAATTCTAGATAGTCTAAAA
GAGACAGGTATATTCTTGTCAAACCATTCATTCTAGCCTTCAATTATAAGGCAAATGATT
ATGCTACCTTTGCACGGTCAGAGTACCGCGGCCGTT-TAAAA-CACTGGGCAGGTCCGAC
TTCGTATCTTAT-ACGAACACTACATTGTATATTTTGTTTGGGATATGATCTGGATTGGT
TGGTACTGCTTTAAGATTGTTAATCCGTGCAGAGTTAGGTCAACCAGGTGCTTTACTTGG
GGATGACCAATTATACAATGTTATTGTAACAGCCCATGCTTTTGTTATGATCTTTTTCTT
GGTAATACCTATAATAATTGGGGGTTTTGGGAATTGACTTGTACCCTTAATGCTAGGCGC
TCCAGATATAGTATTTCCTCGTCTAAATAATATAAGTTTTTGGCTTCTTCCTCCTGCTCT
TTTACTTCTTTTATCTTCAGCTGCTGTGGAAAGTGGAGTGGGTACGGGGTGGACTGTATA
TCCTCCTTTAGCTGGAAATTTAGCTCATGCTGGGGGGTCAGTAGATCTAGCGATTTTTTC
TTTACATTTAGCTGGGGTATCTTCTATTTTGGGGGCAGTAAATTTTATTACCACAATTAT
TAACATACGATGACAGGGAATAAAATTTGAACGTCTTTCATTATTTGTGTGGTCAGTGAA
GGTTACGGCTATTTTACTTCTTTTATCTCTACCTGTATTGGCTGGTGCAATTACTATATT
ATTAACTGATCGGAATTTTAATACTGCTTTCTTTGACCCAGCAGGGGGTGGGGATCCTAT
TTTATATCAGCACCTTTTC
>geographus
AACCAAGAAATTTAGTCATTCTTTTGTGGTAAAAAGACAGTTAGATAGA-CATAATTATT
AGATCCTTAATAGAGGAGTAAAATCTATATATTAAAAGTTATTCCTGAAATGATTT-GCT
TATATCGAATCTGTGACAACTTTAAGGGAAACTGGGATTGGATACCCCATTATTTTCAGT
CGTAAATTTAGTA--TAAACTTACCAGAGTACTACGAATGTTTAAAACTCAAAGGGCTTG
GCGGTGTTTTAGACCTCTCAGGGGAACCTGTCTCATAATCGACAGTCCACGTTAAACCTA
ACCTTTTATAGTAATACAGCTTGTATACCGTCGTCGTCAGGTAACTTCTAAAAATAAAGT
AGTTAGCTTGAGAATT--TT-ATTAATTAGAACGTCAGATCAAGGTGCAGCTAATGAAGA
GGTGAGGATGGGTTACAATTATATATTTGTAAATACGGTGCT-TGAAATTAAGCAC-CTA
AAGGAGGACTTGAAAGTAATTTTAATTATATAAGTAAAATGAATAAGGCTCTGAAACGTG
CAGAATTTTAATGGTCGAACAGACCAACCCTCAAAGACTTCTGCATCTTTAGGATATTCT
GGTCCAACATCGAGGTCACAAACCTTTTTTTCGATATGGGCTCTTAAAAAAGATAATGCT
GTTATCCCTACGGTAACTAATTCCTTTAATCAAAAATTT-TGGATCAAC-ACAAGTCAGA
TTTT-AAAGGAGGCTTCAAGTGCTCCTCGGTTGCCCCAACCAAAGT--ATTTAATAGTTT
T-TCTTTTA-CTTAATTG--ATAAGATCTATTAATTTTCCTAAAGCTCGATAGGGTCTTC
TTGTCTTTTAATACAATCTGAACTTTTTCATCCAAAAATAAAATTCTAAATAATTTAAAA
GAGACAGGTATATTCTTGTCAAACCATTCATTCCAGCCTTCAATTATAAGGCAAATGATT
ATGCTACCTTTGCACGGTCAGAGTACCGCGGCCGTT-TAAAA-CACTGGGCAGGTCCGAC
TTCGTATCTAAT-ATTAACAC--CATTATATATCTTATTTGGAATGTGATCTGGATTGGT
TGGTACTGCTTTAAGGTTGTTAATTCGTGCAGAATTAGGACAACCAGGTGCCTTACTTGG
AGACGATCAATTATATAATGTTATTGTAACAGCTCATGCTTTTGTTATAATTTTTTTCTT
AGTAATACCAATGATGATTGGAGGGTTTGGAAATTGACTTGTGCCTTTAATGTTAGGGGC

CCCAGATATGGTATTTCCTCGCTTAAATAATATAAGTTTTTGACTTCTCCCTCCTGCCCT
TTTACTTCTATTATCGTCAGCTGCTGTAGAAAGTGGAGTGGGTACAGGATGAACTGTATA
CCCACCATTGGCTGGTAATTTAGCTCATGCTGGTGGGTCTGTAGATCTAGCAATCTTTTC
TTTACACCTAGCTGGGGTATCATCTATTTTAGGGGCAGTAAATTTTATTACTACAATTAT
TAATATACGATGACAAGGAATAAAATTTGAACGTCTTTCACTTTTTGTATGGTCGGTAAA
GATTACGGCTATTTTACTTCTTTTATCTTTACCTGTGCTAGCTGGTGCAATTACTATATT
ACTAACTGACCGAAATTTTAATACTGCTTTTTTTGATCCAGCAGGAGGTGGTGATCCTAT
TTTGTATCAACATCTTTTT

>tulipa

NNNNNNNNNNNNNNNNNNNTTCTTTTATGGTAAAAAGACAGTTAGGCAAT-CAGGATTATT
AGATCTTTAGTAGAGGAGTAAAACCTATATACTGAAAGTTATTCCTAAAATAATTT-GTT
TATGTTGAATCTGTGATAGCTTTAAGGGAAACTGGGATTGGATACCCCATTATTTTTAGT
CGTAAATTTAGTT--TAGGCTTACCGGAGTACTACGAATGTTTAAAACTCAAAGGGCTTG
GCGGTGTTTTAGACCCCTCAGGGGAACCTGTCTCATAATCGACAATCCACGTTAAACCTA
ACCTTTTATAGTAATACAGCTTGTATACCGTCGTCGTCAGGTAACTTCTTAAAATAAAAT
AGTTAGCTTGAGAATT--TG-ATTAATTAGAACGTCAGATCAAGGTGCAGCTAATGAAAG
GGTGAGGATGGGTTACAATTACATATTTGTAAATACGGCACT-TGAAATTAAGTGC-TTA
AAGGAGGACTTGAAAGTAATTCTATTTATATAAATAGAATGAATAAGGCTCTGAACCGTG
CAGAATTTTAATGGTCGAACAGACCAACCCTCAAAGACTTCTGCATCTTTAGGATATTCT
GGTCCAACATCGAGGTCACAAACCTTTTTTTCGATATGGGCTCTTAAAAAAGATAATGCT
GTTATCCCTACGGTAACTAATTCCTTTGATCAAAAAATT-TGGATCAAC-ACAAGTCAGA
CTTT-AAAGGAGGCTTTAGATGCTCCTCGGTTGCCCCAACCAAAAT--ATTTAATAGTTT
T-TCTTTTA-CTTAATCG--ACGAGATCTATTAATTTTTCTAAAGCTCGATAGGGTCTTC
TTGTCTTTTAATATTATCTGGACTTTTTCATCCAAAAATAAAGTTCTAAATAATTTAAAA
GAGACAGCTATACTCTTGTCAAACCATTCATTCCAGCCTTCAATTATAAGGCAAATGATT
ATGCTACCTTTGCACGGTCAGAGTACCGCGGCCGTT-TAAAA-CACTGGGCAGGTCCGAC
TTCGTATCTAAC-ATTAACACGACGTTATATATCTTATTTGGGATGTGATCTGGGTTAGT
TGGTACTGCTTTAAGTTTATTAATTCGTGCAGAATTAGGACAACCAGGTGCTTTGCTTGG
AGACGATCAATTGTATAATGTTATTGTGACGGCTCATGCTTTTGTTATAATTTTTTTCTT
GGTGATGCCTATAATGATTGGAGGTTTTGGGAATTGGCTTGTACCTCTAATACTAGGGGC
TCCAGATATAGTATTCCCTCGATTAAATAATATGAGTTTCTGACTTCTACCTCCTGCTCT
TTTACTTCTTTTATCATCGGCTGCTGTAGAAAGTGGGGTAGGTACAGGATGAACTGTATA
TCCTCCTTTGGCTGGTAACTTAGCTCATGCTGGGGGGTCTGTAGATCTAGCAATTTTTTC
TTTACATTTAGCTGGGGTATCTTCTATTTTAGGGGCGGTAAATTTTATTACCACAATTAT
TAATATACGATGACAGGGAATAAAATTTGAGCGCCTTTCTCTTTTTGTGTGATCAGTGAA
GATTACGGCTATCTTGCTTCTTTTATCCTTGCCTGTATTAGCTGGTGCTATTACTATGCT
GTTAACTGATCGAAATTTTAATACTGCCTTCTTTGATCCGGCAGGAGGTGGTGATCCTAT
TTTATATCAGCACCTTTTT

>obscurus

AACCAAGAAATTTAGTCATTCTTTTACGGTAAAAAGACAGTTAGACGGG-TAGAATCGTT
AGATCTTTAGTATAGGAGTAAAATCTAAATACTAAAAGTTATTCCCAAAACGATTT-ATT
AGCGTTGAATCTGTGACAACTTTAAGGGAAACTGGGATTGGATACCCCATTATTTTTAGT
CGTAAATATAGTT--AGGATTTACCAGAGTACTACGAATGTTTAAAACTCAAAGGGCTTG
GCGGTGTTTTAGACCTCTCAGGGGAACCTGTCTCGTAATCGACAATCCACGTCAAACCTA
ACCTCTTATTGTAATACAGCTTGTATACCGTCGTCGTCAGGTAACTTCTTAAAATTGAGT
AGTTAGCTTGAGAATT--TA-ATTGATTAGAACGTCAGATCAAGGTGCAGCTAATGAAGA
GGTGAGGATGGGTTACAATTACATATTCGTAAATACGGCACT-TGAAATGAAGTGC-TTA
AAGGAGGACTTGAAAGTAATTCTAATTATATAAATAGAATGAATGTGGCTCTGAAACGTG

CAGAATTTTAATGGTCGAACAGACCAACCCTTAAAGACTTCTGCATCTTTAGGACATTCT
GGTCCAACATCGAGGTCACAAACCTTTTTTTCGATATGGGCTCTCAAAAAAGATAATGCT
GTTATCCCTACGGTAACTAATTCCTTTGATCAAAAAACT-TGGATCAAC-ACAAGTCAGA
CTTA-AAAGGAGGCTTTAGATACTCCTTGGTTGCCCCAACCAAAGT--ATTTAATAGTTT
T-TCTTTTA-GCTAATTG--AT-AGATCTATTAATTCTTCTAAAGCTCGATAGGGTCTTC
TTGTCTTTTAATAAAATCTGAACTTTTTCATTCAGAAATAAAATTCTAAGTAATCTAAAA
GAGACAGGCGTATTCTTGTCAAACCATTCATTCCAGCCTTCAATTATAAGGCAAATGATT
ATGCTACCTTTGCACGGTCAGAGTACCGCGGCCGTT-TAAAA-CACTGGGCAGGTCCGAC
TTCGCATTTTGT-TCTAACGCAACATTGTATATTTTGTTTGGAATATGATCGGGATTGGT
TGGAACTGCTTTAAGGTTGTTAATTCGTGCAGAATTAGGGCAGCCAGGTGCTTTACTTGG
TGACGATCAGCTGTATAATGTTATTGTAACAGCCCATGCTTTTGTTATAATTTTTTTCTT
AGTAATGCCTATAATGATTGGAGGTTTTGGAAACTGGCTTGTACCTTTGATGTTAGGGGC
TCCAGATATGGTATTTCCTCGTCTAAATAATATAAGTTTTTGACTTCTTCCTCCTGCTCT
TTTGCTTCTCCTATCATCAGCCGCCGTAGAGAGTGGTGTAGGTACAGGGTGAACTGTATA
TCCTCCTCTTTCTGGGAATTTAGCCCATGCTGGGGGGTCTGTAGATCTAGCAATTTTTTC
TTTACATCTAGCTGGTGTATCCTCTATTTTGGGGGCAGTAAACTTTATTACTACAATCAT
TAATATACGATGACAAGGAATGAAGTTTGAGCGTCTCTCTTTGTTTGTTTGATCGGTAAA
AATTACAGCTATTTTACTTTTACTATCTTTGCCTGTCTTAGCTGGTGCAATTACAATGTT
GTTAACTGATCGAAATTTTAATACTGCTTTCTTTGATCCAGCGGGTGGTGGGGATCCAAT
CTTGTATCAGCACCTCTTC
>spurius
AACCAAGAAATTTAGTCATTCATTTACGGTAAAAAGACAGTTAGGTAAA-TAGGTCATTA
AGATCCTTAGTATAGGAGTAAAATCTAAATACTAAAAGTTATACCGAATATGGCTT-ACC
TATACTGAATCTGTGACAACTTTAAGGGAAACTGGGATTGGATACCCCATTATTTTTAGT
TGTAAATCTAGTT--TAAGCTTACCGGAGTACTACGAATGTTTAAAACTCAAAGGGCTTG
GCGGTGTTTTAGACTTCTCAGGGGAACCTGTCTCGTAATCGACAATCCACGTTAAACCTA
ACCTTTTATGGTAATACAGTTTGTATACCGTCGTCGTCAGGTAACTTCTAAAAATATGGT
AGTTAGCTCGAGAGTC--TT-ATTAGTTAAAACGTCAGATCAAGGTGCAGCTAATAAAAA
GGTGAGGATGGGTTACAATTATATATTTATAAATACGGCACTATGAAATGGAGTGTCTTA
AAGGAGGACTTGAAAGTAATTTTAATTATGTAAATAAAATGAATTTGGCTCTGAAACGTG
CAGAATTTTAATGGTCGAACAGACCAACCCTCAAAGACTTCTGCATCTTTAGGACATTCT
GGTCCAACATCGAGGTCACAAACCTTTTTTTCGATATGGGCTCTTGAAAAAGATAATGCT
GTTATCCCTACGGTAACTAATTCCTTTAATCAATAATGTTTGGATCAAT-GCAAGTAAGA
TTTT-AAAGGAGGCTTCACCTACTCCTCGGTTGCCCCAACCAAAAT--ATTTAATAGCTC
T-TCTTTTA-TTTAATTG--ATAGAATCTATTAATTTTTTTAAAGCTCGATAGGGTCTTC
TTGTCTTTTAATTTCATCTGGACTTTTTCATCCAAAAATAAAATTCTAGATAATTTAAAA
GAGACAGGTGTATTCTTGTCAAACCATTCATTCCAGCCTTCAATTATAAGGCAAATGATT
ATGCTACCTTTGCACGGTCAGAGTACCGCGGCCGTT-TAAAA-CACTGGGCAGGTCCGAC
TTCATATTTAGT-ACTAACATGACATTATATATTCTGTTTGGAATATGATCTGGGTTGGT
TGGAACTGCTTTGAGATTGTTAATTCGGGCAGAATTAGGGCAGCCAGGTGCTTTACTTGG
AGATGATCAGTTATATAATGTGATTGTAACAGCACACGCTTTTGTTATAATCTTTTTTTT
AGTAATGCCTATAATAATTGGTGGTTTTGGAAACTGATTAGTGCCCTTAATGCTGGGTGC
TCCTGATATGGTGTTTCCTCGACTAAATAATATGAGTTTTTGGCTTCTTCCTCCTGCGCT
TTTGCTTCTTTTATCATCGGCTGCGGTAGAAAGTGGTGTGGGTACTGGATGAACTGTTTA
TCCACCTTTAGCAGGAAATCTAGCTCATGCTGGTGGATCTGTGGATCTGGCGATTTTTTC
TTTACATCTAGCTGGTGCTTCTTCTATTTTAGGGGCAGTAAATTTCATTACTACAATTAT
TAATATGCGGTGGCAGGGGATGAAATTTGAACGTCTGTCACTTTTTGTATGGTCGGTAAA
AATCACAGCTATTTTGCTTCTTTTATCTTTACCAGTATTAGCTGGTGCAATTACGATACT

```
TTTAACTGATCGAAACTTTAATACTGCTTTTTTTGATCCTGCGGGAGGCGGTGATCCTAT
TTTGTATCAGCATCTGTTT
>atlanticus
AACCAAAAAATTTAGTCATTCATTTACGGTAAAAAGACAGTTAGGTAAA-GAGGTCATTA
AGATCTTTAGTATAGGAGTAAAATCTAAATACTAAAAGTTATACCAGATATGGCTT-GCC
TATACTGAATCTGTGACAACTTTAAGGGAAACTGGGATTGGATACCCCATTATTTTTAGC
TGTAAATCTAGTT--TAAGCTTACCGGAGTACTACGAATATTTAAAACTCAAAGGGCTTG
GCGGTGTTTTAGACTTCTCAGGGGAACCTGTCTCGTAATCGACAATCCACGTTAGACCTA
ACCTTTTATGGTAATACAGTCTGTATACCGTCGTCGTCAGGTAACTTCTAAAAATATGGT
GGTTAGCTCGACAGTC--TT-ATTAGCTAAAACGTCAGATCAAGGTGCAGCTAATAAAAA
GGTGAGGATGGGTTACAATTATATATTTATAAATATGGCACTATGAAATGGAGTGTCTTA
AAGGAGGACTTGAAAGTAATTTTAATTATATAAATGAGATGAATTTGGCTCTGAAACGTG
CAGAATTTTAATGGTCGAACAGACCAACCCTCAAAGACTTCTGCATCTTTAGGACATTCT
GGTCCAACATCGAGGTCACAAACCTTTTTTTCGATATGGGCTCTTGAAAAAGATAATGCT
GTTATCCCTACGGTAACTAATTCCTTTGATCAATATTATTTGGATCAAT-GCAAGTAAGA
TTTT-AAAGGAGGCTTCATCTACTCCTCGGTTGCCCCAACCAAAAT--ATTTAATAGTTC
T-TCTTTTA-CTTAATTG--ATAGAATCTATTAATTTTTTTAAAGCTCGATAGGGTCTTC
TTGTCTTTTAATTTCATCTGGACTTTTTCATCCAAAAATAAAATTCTAGATAATTTAAAA
GAGACAGGTGTATTCTTGTCAAACCATTCATTCCAGCCTTCAATTATAAGGCAAATGATT
ATGCTACCTTTGCACGGTCAGAGTACCGCGGCCGTT-TAAAA-CACTGGGCAGGTCCGAC
TTCATATTTAGT-ACTAACATGACATTATATATCCTTTTTGGAATATGATCTGGGTTGGT
TGGAACTGCTTTGAGGTTGTTAATTCGTGCAGAATTAGGGCAGCCAGGTGCTTTACTTGG
AGATGATCAGTTATATAATGTAATTGTAACAGCACACGCTTTTGTTATAATCTTTTTTTT
AGTAATGCCTATAATAATTGGTGGTTTTGGAAACTGATTAGTGCCCTTGATGCTGGGTGC
TCCTGATATAGTGTTTCCTCGGCTAAATAATATAAGTTTCTGGCTTCTTCCTCCTGCGCT
CTTGCTTCTTTTATCATCGGCTGCGGTAGAGAGTGGCGTGGGTACTGGATGAACTGTTTA
TCCGCCTTTAGCAGGAAATCTGGCCCATGCTGGTGGGTCTGTGGATCTGGCAATTTTTTC
TTTACATCTAGCCGGTGCTTCTTCTATTTTAGGAGCGGTAAATTTCATTACTACAATTAT
TAATATGCGGTGGCAGGGAATGAAATTTGAGCGTCTGTCACTTTTTGTGTGGTCGGTAAA
AATCACAGCTATTTTACTTCTTTTATCTTTACCAGTATTAGCTGGTGCAATTACGATACT
TTTAACTGATCGAAACTTTAATACTGCTTTTTTTGATCCTGCAGGAGGTGGTGATCCTAT
TTTGTATCAGCATCTGTTT
>balteatus
AACCAAGAAATTTAGTCATTCTTTTTCGGTAAAAAGACAGTTAGGCAAA-AAAGGCCATT
GGACCTTTAGTACAGGGGTAAAATCTAAATACTAAAAGTTATGCTAGATATGGCCA-TTT
TGTGCTGAATCTGTGACGACTTTAAGGGAAACTGGGATTGGATACCCCATTATTTTTAGT
AGTAAA-TTAACT--TAAGTTTACCAGAGTACTACGAATATTTAAAACTCAAAGAGCTTG
GCGGTGTTTTAGACCTCTCAGGGGAACCTGTCTCATAATCGACAATCCACGATATACCTG
ACCCTTCGTAGCAATTCAGCTTGTATACCGTCGTCGTCAGGTAACTTCTTAAAATATAGT
AGTTAGCTTGAAAATT--TT-ATTGATTAATACGTCAGATCAAGGTGCAGCTAATAAAAG
GGAGAGGATGGGTTACAATTACATATTTGTAATTACGGTATT-TTAAAAGAAATATTCTG
AAGGAGGACTTGAAAGTAATCTTAGTTATATAAATAAGATGAATAAGGCTCTGAAACGTG
CAGAATTTTAATGGTCGAACAGACCAACCCTTAAAGACTTCTGCATCTTTAGGACATTCT
GGTCCAACATCGAGGTCACAAACCTTTTTTTCGATATGGGCTCTTGAAAAAGATAATGCT
GTTATCCCTACGGTAACTAATTCCTTTGATCAAAATCTT-TGGATCAAT-ACGAGTGTGA
TTTA-AAAGGAGGCTTTATCTACTCCTCGGTTGCCCCAACCAAAGTACACTTAATGGCTT
T-TCTTTTA-CTTGATTG--ATAAAGTCCATTAATTCTTCTAAAGCTCGATAGGGTCTTC
TTGTCTTTTAATTATATCTGGACTTTTTCATCCAAAGATAAAATTCTAGAAAATCAAAAA
```

GAGACAGATGTATTCTTGTCAAACCATTCATTCCAGCCTTCAATTATAAGGCAAATTATT
ATGCTACCTTTGCACGGTCAGAGTACCGCGGCCGTT-TAAAA-CACTGGGCAGGTCCGAC
TTCGTATTTAGA-ACTGACACGACATTGTATATTTTATTTGGAATATGGTCTGGATTAGT
TGGGACTGCTTTAAGTTTATTAATTCGTGCAGAATTAGGGCAGCCTGGTGCTTTGCTCGG
AGATGATCAACTATATAATGTAATTGTAACAGCCCATGCTTTTGTTATAATTTTCTTTTT
AGTAATGCCTATAATAATTGGGGGATTTGGAAACTGGTTGGTACCATTGATATTAGGGGC
TCCAGATATAGTTTTTCCTCGTTTGAATAATATAAGTTTTTGGCTTCTTCCTCCTGCATT
ATTACTTCTTTTATCATCGGCTGCAGTAGAAAGAGGAGTAGGTACAGGATGAACAGTGTA
TCCACCCTTAGCGGGAAATTTAGCCCATGCTGGAGGTTCTGTGGACCTAGCAATTTTTTC
TCTTCATCTTGCCGGGGTTTCTTCTATTTTAGGGGCAGTAAACTTTATTACCACAATTAT
TAATATACGATGACAAGGAATAAAATTTGAGCGTCTTTCGTTGTTTGTATGATCTGTAAA
AATTACTGCTATTTTACTTTTATTATCTCTACCTGTATTAGCTGGGGCTATTACTATGCT
TTTAACTGATCGAAATTTTAATACTGCTTTTTTTGATCCAGCAGGAGGTGGTGACCCAAT
CTTGTATCAACATTTATTT
>californiconus
TACCAAAAAATTAAGTCATACCACTCCGGTAAAAAGACAGTTAGACATG-AATAGTCTAT
AAAATATTGATATAAAGGTAAAATTTGTATATCAGTTAGTAGTTTAATAATGACCT----
-ATGTTGAATCTGTGACAGCTTTGAGGGAAACTGGGATTGGATACCCCACTATTCTTAGC
TGTAAACTTGATTGCTTGATTTACCAGAGCACTACGAACATTTAAAACTCAAAGGGCTTG
GCGGTGTTTTAGACCATTCAGGGGAACCTGTCTCATAATCGACAATCCACGTTAAACCTG
ACCTTTTTTCGTAAT-CAGCCCGTATACCGTCGTCGTCAGGTAACTTCTAAAAATTTAAA
AGTTAGCTAGAAAACTACTA-ATAAGTTAAAACGTCAGATCAGGGTGCAGCCTATAAAAA
GGGGAGGATGGGTTACAATTAAATATTCTTAACTACGGTTAT-TCAGA-AAGATAACTTG
AAGGAGGACTTGAAAGTAATTTTTTTTACATAAATTATATGAATTAGGCTCTGAAACGTG
CAGAATTTTAATGGTCGAACAGACCAACCCTTGAAGACTTCTGCATCTTCAGGATATTCT
GGTCCAACATCGAGGTCACAAACCTTTTTTTCGATAGGAACTCTCAAAAAAGATAATGCT
GTTATCCCTACGGTAACTATTTTCTTTGATCAAAAAGTT-TGGATCAGT-ACTTGTTAGT
TTAT-AAAGGAGGCTTTTTTTACTCCTTAGTTGCCCCAACCAAAAT--GTTTAACAACAC
A-ATTTTTT-CGTCAATA--AATTCTTTTGTTAATTTATTTCAAGCTCGATAGGGTCTTC
TTGTCTTTTAATTATATTAGGACTTTTTCATCCCAAAATAAAATTCTATTGAATCATAAA
GAGACAGTTGCACTCTTGTCAAACCATTCATTCCAGCCTTCAATTATAAGGCAAATGATT
ATGCTACCTTTGCACGGTCAGAGTACCGCGGCCGTTGAAAAA-CACTGGGCAGGTCCGAC
TTCATATC-AAA-AATGTCATAACATTATATATTTTATTTGGTATATGATCTGGGTTAGT
TGGTACAGCTTTAAGTCTACTTATTCGGGCTGAATTAGGTCAACCTGGAGCCTTATTAGG
AGATGATCAGCTGTATAATGTTATTGTTACGGCTCATGCTTTCGTTATAATTTTTTTCTT
GGTTATGCCTATGATAATTGGTGGTTTTGGTAACTGGTTGGTACCTTTAATACTTGGAGC
ACCTGATATGGTATTTCCACGACTGAATAATATAAGCTTTTGACTTTTACCCCCTGCTTT
GTTATTACTTCTATCATCAGCTGCAGTGGAAAGAGGTGTGGGGACTGGATGAACGGTCTA
TCCCCCATTATCTGGAAACCTAGCTCATGCAGGAGGTTCGGTAGACTTGGCCATTTTTTC
ATTGCACCTTGCTGGTGTTTCATCAATTTTAGGGGCAGTAAATTTTATTACAACAATTAT
CAACATACGATGACAAGGAATGCAATTTGAGCGTCTTCCTTTGTTTGTATGATCGGTGAA
AATTACTGCTATTCTTTTACTTCTGTCGCTACCTGTGTTAGCAGGAGCCATTACTATGCT
TCTAACAGATCGAAACTTTAATACAGCTTTCTTTGATCCAGCAGGAGGTGGTGATCCGAT
CCTATATCAGCATTTGTTT
